# Synthesis and Application of New Chiral Phosphoric Acids Based on *oxa*-Spirocyclic Diphenol

**DOI:** 10.3390/molecules31213858

**Published:** 2026-10-26

**Authors:** Jia-Jie Chen, Zhuo-Ting Chen, Peng Wang, Wen-Jing Wang, Li-Guo Shen, Zhi-Yuan Bai, Si-Hai Zhou, Zehua Yang, Ye-Hui Chen

**Affiliations:** School of Pharmaceutical Science, Hengyang Medical School, University of South China, Hengyang 421001, China; 20252012111034@stu.usc.edu.cn (J.-J.C.); 20242012211047@stu.usc.edu.cn (Z.-T.C.); 20242012111027@stu.usc.edu.cn (P.W.); 17797512194@163.com (W.-J.W.); clgsr830@163.com (L.-G.S.); 19174679631@163.com (Z.-Y.B.); 13155709589@163.com (S.-H.Z.); yangzehua@usc.edu.cn (Z.Y.)

**Keywords:** *oxa*-spirocyclic diphenol, chiral phosphoric acid, synthesis, application

## Abstract

Chiral phosphoric acids (CPAs) are a highly versatile class of Brønsted acid catalysts. They have been employed in various challenging organic transformations. However, previously reported chiral phosphoric acids are still limited in variety. In this work, a new type of CPAs with different substitutions (27 examples) derived from the *O*-SPINOL scaffold was synthesized through 8 steps. The new type of CPAs were applied to the electrophilic amination of indoles bearing bulky substituents and exhibited high catalytic efficiency. Preliminary comparative experiments revealed that the chiral phosphoric acid derived from *O*-SPINOL gave better catalytic performance in terms of enantioselectivity than that derived from SPINOL in most examples. This new type of chiral phosphoric acids adds to and complements the existing pool of chiral phosphoric acid catalysts.

## 1. Introduction

**Scheme 1:**
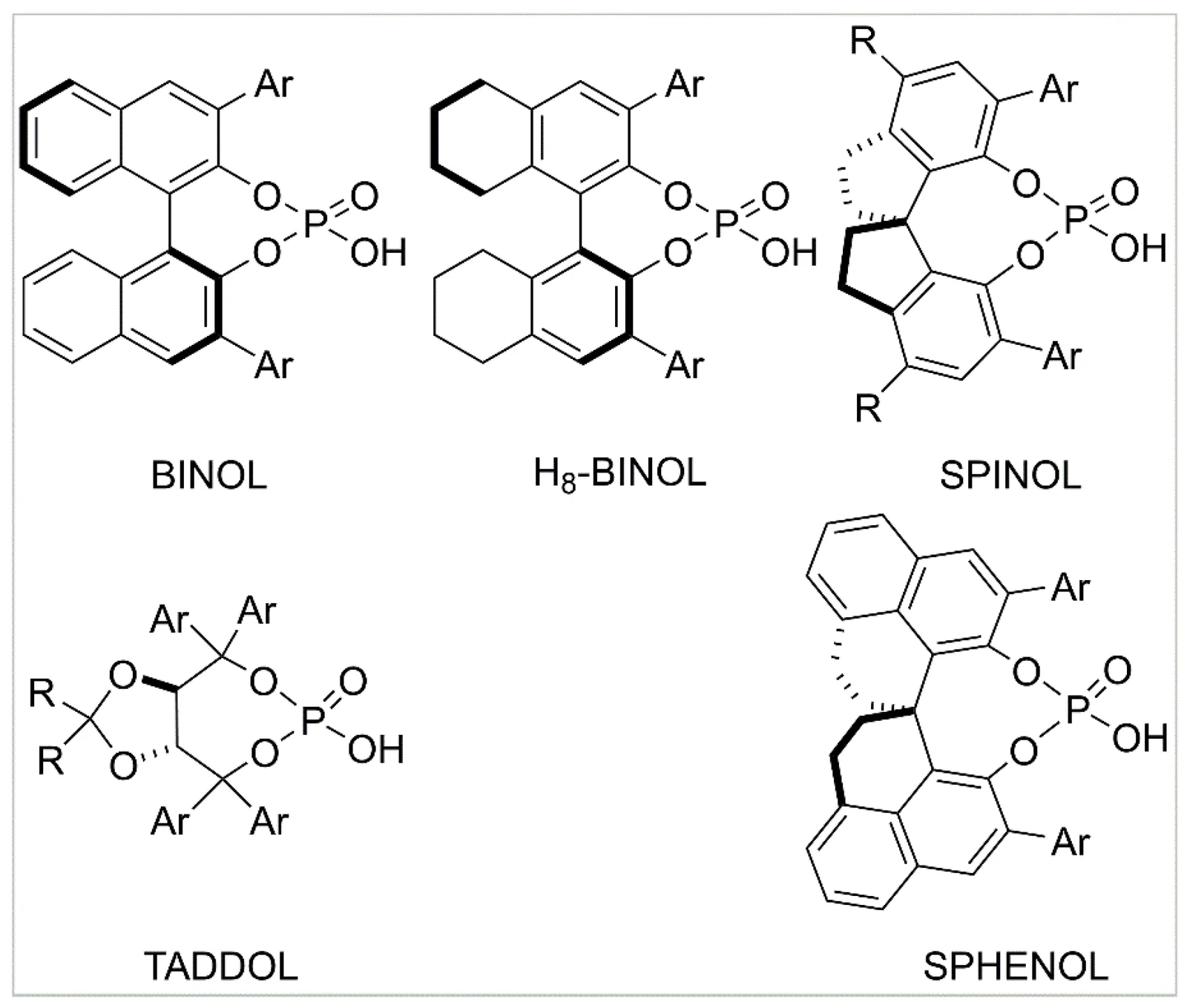
The main chiral phosphoric acids (CPAs) reported.

Asymmetric catalysis [1,2,3,4,5] represents the most direct and efficient approach to access chiral compounds, and it has been widely applied [1,2,3,4,5] in pharmaceutical synthesis, materials science and energy-related fields. The development of chiral catalysts has emerged as a key research area in asymmetric catalysis [1,2,3,4,5]. As a very important class of organocatalysts, chiral phosphoric acids [6,7,8,9,10,11,12,13,14,15] have attracted extensive attention in recent years. Up to now, the types of existing chiral phosphoric acids have been relatively scarce, with only a limited number of scaffolds, which are derived from BINOL, H_8_-BINOL, SPINOL, TADDOL [16], and SPHENOL [17], respectively (Scheme 1). In addition, some examples of chiral bisphosphoric acids [18] have also been reported.

**Scheme 2:**
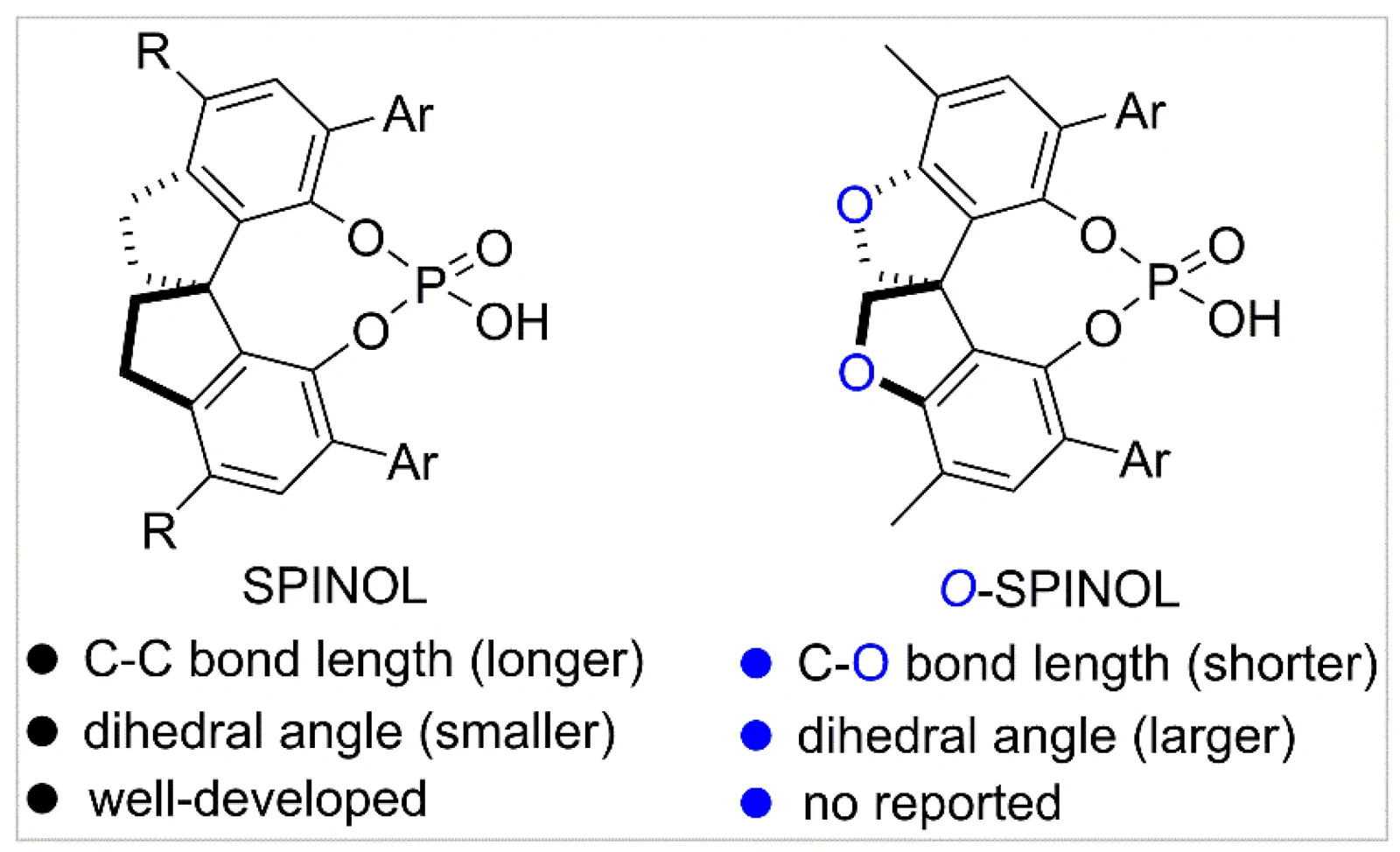
The characteristic of *O*-SPINOL-derived CPAs.

Accordingly, developing chiral phosphoric acids based on new scaffolds is of great significance. Such efforts can not only enrich the existing chiral phosphoric acid library but also expand their application scope, thereby advancing the development of related fields. The *oxa*-spirocyclic backbone has been extensively employed in asymmetric catalysis [19,20,21,22,23,24,25,26,27,28,29,30,31]. This paper describes the synthesis of a new type of chiral phosphoric acid derived from the *oxa*-spirocyclic diphenol (*O*-SPINOL) framework. Compared with the SPINOL skeleton, the *O*-SPINOL scaffold has the following characteristics: the *O*-SPINOL framework exhibits greater planar rigidity and a bigger dihedral angle because of the shorter C–O bond relative to the C−C bond [27,28,29]. Catalysts derived from the *O*-SPINOL scaffold perhaps exhibit distinct properties compared with those derived from the SPINOL scaffold, serving as an enrichment and complement to the existing SPINOL scaffold [28,29] (Scheme 2).

## 2. Results and Discussion

### 2.1. Synthesis of O-SPINOL-Derived CPAs (S)-***9***

**Scheme 3:**
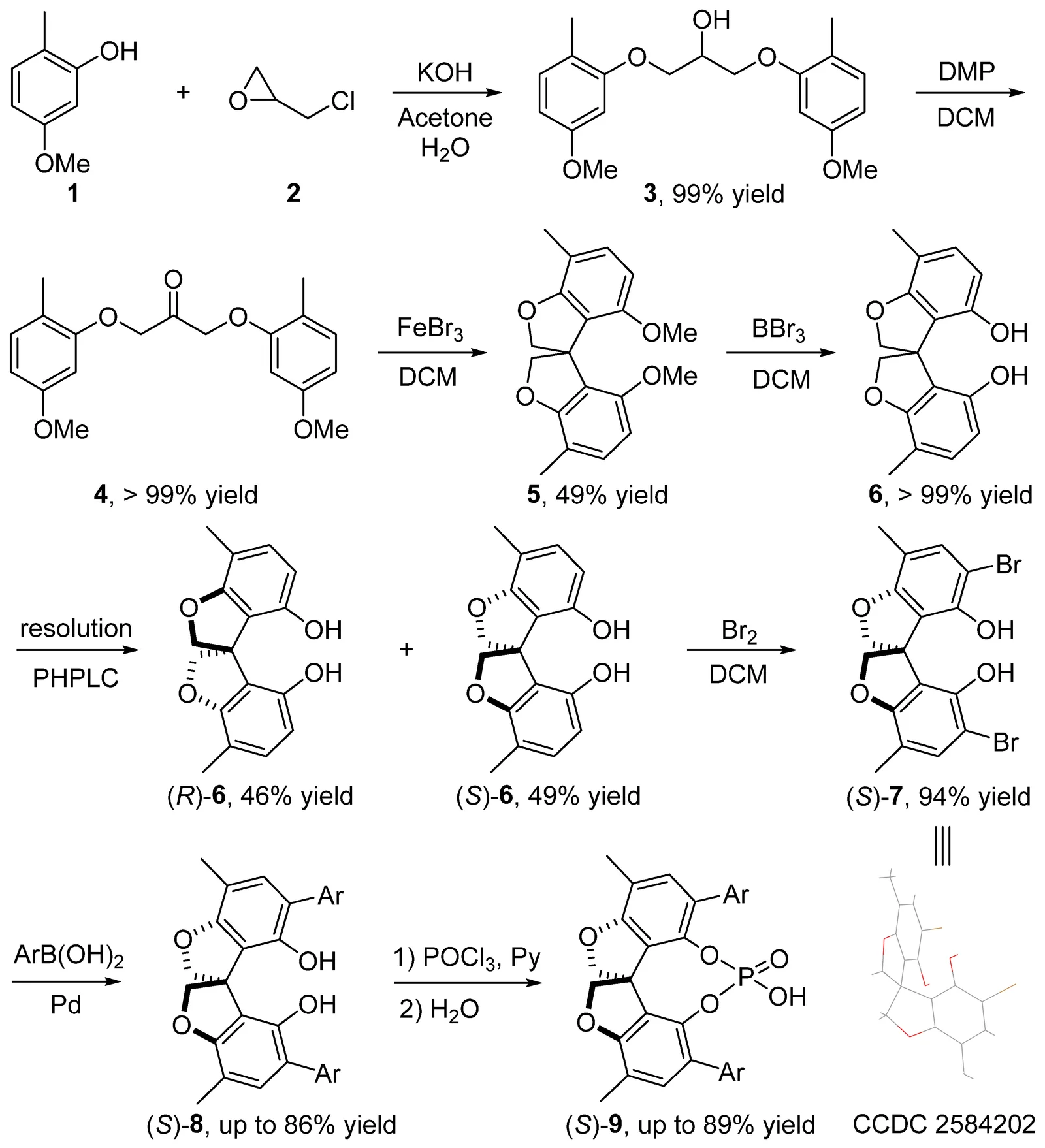
Synthetic route to CPAs (*S*)-**9**.

As illustrated in Scheme 3, a series of chiral phosphoric acids bearing different substituents can be conveniently prepared in eight steps. Our investigation began with 5-methoxy-2-methylphenol **1** and racemic 2-(chloromethyl)oxirane **2** which are commercially available. It was found that secondary alcohol **3** could be obtained quantitatively employing potassium hydroxide (KOH) as the base in acetone. When Dess–Martin periodinane (DMP) was used as the oxidant in dichloromethane (DCM), the desired ketone **4** was obtained in high yield. The *oxa*-spirocyclic compound **5** can be readily obtained in the presence of iron(III) bromide (FeBr_3_) using DCM as the solvent. Demethylation was carried out using boron tribromide (BBr_3_) to afford the *oxa*-spirocyclic diphenol **6** efficiently. Resolution of the racemate **6** was achieved via preparative high-performance liquid chromatography (PHPLC) [32]. Both enantiomers can be obtained in greater than 99% enantiomeric excess (ee). The first five steps were carried out according to the procedures described in [32]. Bromination of (*S*)-**6** was carried out using bromine in DCM to afford (*S*)-**7**. The absolute configuration of (*S*)-**7** was confirmed by single-crystal X-ray diffraction analysis. A series of aryl-substituted *oxa*-spirobisphenols (*S*)-**8** were obtained through the Suzuki coupling of (*S*)-**7** with arylboronic acids, catalyzed by a palladium/phosphine ligand. Treatment of (*S*)-**8** with phosphorus oxychloride (POCl_3_) under basic condition, followed by hydrolysis, afforded the chiral phosphoric acids (*S*)-**9**. A total of 27 chiral phosphoric acids bearing different substituents were synthesized. Single-crystal X-ray diffraction analysis of (*S*)-**9-21** confirmed the absolute configuration of the chiral phosphoric acids. All products were characterized by ^1^H NMR, ^13^C NMR, and HRMS.

### 2.2. Application of O-SPINOL-Derived CPAs (S)-***9*** in Electrophilic C_3_-Amination of Indoles—Reaction Condition Optimization

Axially chiral 3-arylaminoindoles are an important class of compounds due to their significant anticancer activity [33]. Electrophilic *C*_3_-amination of indoles with quinone diimine (QDI) is the most straightforward and efficient approach to access 3-arylaminoindoles [33]. Therefore, the aforementioned CPAs were applied to the electrophilic amination of sterically hindered indoles with QDI to evaluate their catalytic activity. Our investigation began by employing QDI **10** and 2-(tert-butyl)-4-methyl-1H-indole (**11-1**) as substrates, with chiral phosphoric acid (*S*)-**9** as the catalyst, in DCM at room temperature (Table 1, entries 1–6). To our delight, among the catalysts screened, (*S*)-**9-22** gave the best results with respect to enantioselectivity and chemical yield (84% ee, 96% yield, entry 5). Screening of solvents (Table 1, entries 5, 7–8) revealed DCM to be the most suitable (entry 5). Further optimization of the reaction concentration, catalyst loading, and temperature established the optimal conditions as follows: treatment of **10** (0.05 mmol) with **11-1** (0.10 mmol) in the presence of (*S*)-**9-22** (0.2 mol%) in DCM at −20 °C for 12 h furnished **12-1** in 98% yield with 97% ee (Table 1, entry 11). The reaction time was determined by TLC analysis, which indicated that the starting material **10** was completely consumed. Catalyst loading below 0.2 mol% was attempted and lower temperatures than −20 °C were also examined. Apart from the need to extend the reaction time to ensure complete consumption of starting material **10**, the yield and enantioselectivity could almost be maintained. The optimized reaction conditions were almost identical to those in [33], except that the solvent was changed from DCE to DCM.

### 2.3. Substrate Scope

**Scheme 4:**
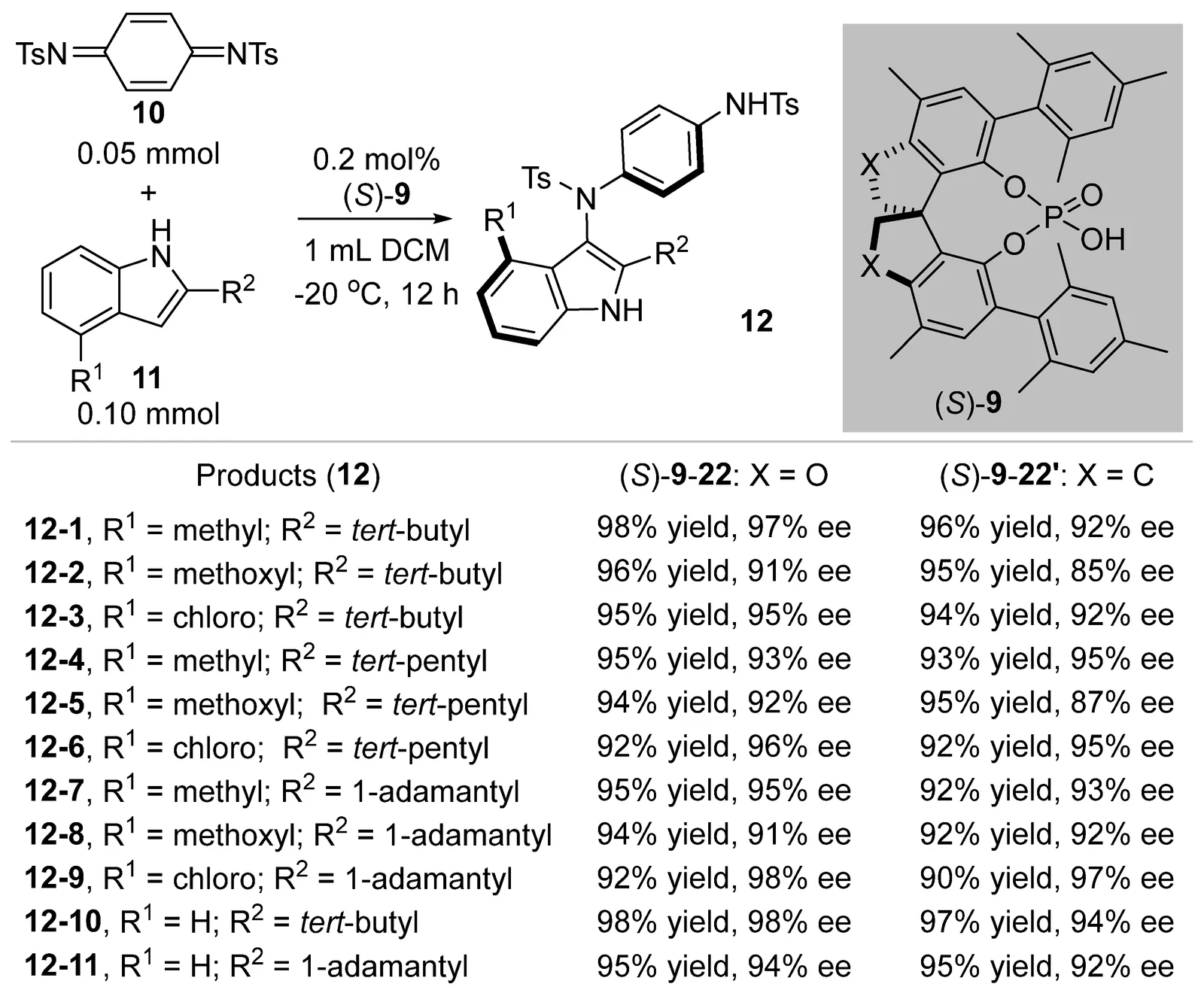
Electrophilic amination of indoles catalyzed by (*S*)-**9-22** and (*S*)-**9-22’**.

Armed with the optimized conditions, we explored the substrate scope using a variety of indole derivatives. As shown in Scheme 4, all of the tested indoles **11** reacted smoothly with **10** to exclusively afford the desired *N*-sulfonyl-3-arylaminoindoles **12** in high yields (92–98% yield) with excellent enantioselectivities (91–98% ee). With respect to yield and enantioselectivity, (*S*)-**9-22** exhibited catalytic performance comparable to that of the catalyst reported in [33]. Meanwhile, the utility of *O*-SPINOL-derived CPA (*S*)-**9-22** in catalyzing electrophilic *C*_3_-amination of indoles was showcased by a large-scale synthesis of **12-10**, which provided 0.58 g of the product in 99% yield and 98% ee, with essentially no change in either yield or enantioselectivity. The absolute configurations of the products were assigned by analogy with known compound [33] (for details, see the Supporting Information, pages 61 and 62).

### 2.4. Application of SPINOL-Derived CPA (S)-***9***-***22’*** in Electrophilic C_3_-Amination of Indoles—Comparison of Catalytic Performance

To further illustrate the potential utility of this type of scaffold, we employed the SPINOL-derived chiral phosphoric acid (*S*)-**9-22′** to catalyze the reaction under the optimized conditions for *O*-SPINOL-derived chiral phosphoric acid (*S*)-**9-22** (Scheme 4). As shown in Scheme 4, the results indicated that the chiral phosphoric acid (*S*)-**9-22** derived from *O*-SPINOL gave better catalytic performance in terms of enantioselectivity in most examples. This improved catalytic effect may be attributable to the greater planar rigidity and larger dihedral angle of the *O*-SPINOL framework [29].

## 3. Materials and Methods

### 3.1. General Information

NMR spectra were recorded in CDCl_3_ or DMSO-d_6_ on a Bruker DPX 400 or 500 NMR spectrometer. Chemical shifts (δ) are reported in ppm relative to the residual solvent peak. Coupling constants (J) are given in Hertz (Hz), and multiplicities are denoted as s (singlet), d (doublet), t (triplet), q (quartet), dd (double of doublet) and m (multiplet). High-resolution mass spectra (HRMSs) were acquired using an Agilent 1260 Series time-of-flight (TOF) electrospray ionization (ESI) mass spectrometer. Enantioselectivities were recorded on Shimadzu HPLC using chiral columns from Guangzhou Starchrom Technologies Co., Ltd. (Guangzhou, China).

### 3.2. Application of CPAs (S)-***9*** in Asymmetric Synthesis of ***12***

#### 3.2.1. General Procedure for Asymmetric Synthesis of Axially Chiral 3-Aryl-Aminoindoles **12**

Into an oven-dried Schlenk tube chiral phosphoric acid, (*S*)-**9-22** (0.2 mol%, 60 μg/mL in DCM, 1 mL) and **11** (0.10 mmol) were added. The reaction mixture was stirred at −20 °C for 10 min, then **10** (0.05 mmol) was added. The reaction was stirred at −20 °C for 12 h. Solvent was removed under reduced pressure and the crude residue was purified by column chromatography on silica gel (EA/DCM = 1/30) to give pure product **12**.

#### 3.2.2. N-(2-(tert-butyl)-4-methyl-1H-indol-3-yl)-4-methyl-N-(4-((4-methylphenyl)sulfonamido)phenyl)benzenesulfonamide (**12-1**)

White solid, 29.49 mg, 98% yield; 97% ee. ^1^H NMR (400 MHz, CDCl3) δ 8.40 (s, 1H), 7.67 (d, *J* = 8.1 Hz, 2H), 7.53 (d, *J* = 8.1 Hz, 2H), 7.23–7.11 (m, 7H), 7.01 (t, *J* = 7.6 Hz, 1H), 6.91–6.88 (m, 3H), 6.64 (d, *J* = 7.2 Hz, 1H), 2.41 (s, 3H), 2.33 (s, 3H), 1.38 (s, 9H), 1.33 (s, 3H). ^13^C NMR (100 MHz, CDCl3) δ 145.87, 144.14, 143.82, 141.98, 137.21, 135.81, 134.20, 131.06, 129.63, 129.59, 129.20, 128.36, 127.32, 124.11, 123.30, 122.40, 122.30, 119.97, 109.56, 108.60, 33.55, 30.13, 21.66, 21.60, 18.54. HRMS (ESI) *m*/*z* [M + H]^+^ calcd for C_33_H_36_N_3_O_4_S_2_^+^ 602.2142; found: 602.2144. HPLC conditions: Cel-Z (Guangzhou Starchrom Technologies Co., Ltd.); hexane = 0.65 mL/min, isopropanol = 0.35 mL/min; T = 40 °C; λ = 254 nm; t_R_ (major) = 10.2 min, t_R_ (minor) = 13.3 min.

#### 3.2.3. N-(2-(tert-butyl)-4-methoxy-1H-indol-3-yl)-4-methyl-N-(4-((4-methylphenyl)sulfonamido)phenyl)benzenesulfonamide (**12-2**)

White solid, 29.65 mg, 96% yield; 91% ee. ^1^H NMR (400 MHz, CDCl_3_) δ 8.17 (s, 1H), 7.55–7.52 (m, 4H), 7.34 (d, *J* = 9.0 Hz, 2H), 7.14 (d, *J* = 7.9 Hz, 4H), 7.03 (t, *J* = 7.9 Hz, 1H), 6.91 (d, *J* = 8.1 Hz, 1H), 6.85 (d, *J* = 8.9 Hz, 2H), 6.55 (s, 1H), 6.23 (d, *J* = 7.8 Hz, 1H), 2.84 (s, 3H), 2.38 (s, 3H), 2.35 (s, 3H), 1.46 (s, 9H). ^13^C NMR (100 MHz, CDCl_3_) δ 152.03, 144.48, 143.78, 142.97, 141.74, 137.56, 136.04, 134.82, 131.50, 129.57, 128.70, 128.59, 127.40, 123.01, 122.72, 122.37, 115.83, 110.06, 104.05, 99.93, 53.64, 33.29, 30.27, 21.65, 21.60. HRMS (ESI) *m*/*z* [M + Na]^+^ calcd for C_33_H_35_N_3_O_5_S_2_Na^+^ 640.1910; found: 640.1911. HPLC conditions: Cel-D (Guangzhou Starchrom Technologies Co., Ltd.); hexane = 0.80 mL/min, isopropanol = 0.20 mL/min; T = 40 °C; λ = 254 nm; t_R_ (major) = 19.8 min, t_R_ (minor) = 25.8 min.

#### 3.2.4. N-(2-(tert-butyl)-4-chloro-1H-indol-3-yl)-4-methyl-N-(4-((4-methylphenyl)sulfonamido)phenyl)benzenesulfonamide (**12-3**)

White solid, 29.55 mg, 95% yield; 95% ee. ^1^H NMR (400 MHz, CDCl_3_) δ 8.46 (s, 1H), 7.64 (d, *J* = 8.1 Hz, 2H), 7.48 (d, *J* = 8.1 Hz, 2H), 7.24–7.18 (m, 5H), 7.11 (d, *J* = 8.0 Hz, 2H), 7.02 (t, *J* = 7.9 Hz, 1H), 6.86 (t, *J* = 7.5 Hz, 3H), 6.48 (s, 1H), 2.40 (s, 3H), 2.33 (s, 3H), 1.45 (s, 9H). ^13^C NMR (100 MHz, CDCl_3_) δ 147.66, 143.91, 143.80, 142.17, 136.86, 135.84, 135.14, 130.98, 129.61, 129.39, 128.63, 127.37, 124.10, 123.48, 122.88, 122.45, 121.78, 120.33, 109.66, 109.22, 33.71, 30.13, 21.72, 21.65. HRMS (ESI) *m*/*z* [M + H]^+^ calcd for C_32_H_33_ClN_3_O_4_S_2_^+^ 622.1596; found: 622.1598. HPLC conditions: Cel-X (Guangzhou Starchrom Technologies Co., Ltd., Guangzhou, China); hexane = 0.70 mL/min, isopropanol = 0.30 mL/min; T = 40 °C; λ = 254 nm; t_R_ (major) = 25.0 min, t_R_ (minor) = 39.5 min.

#### 3.2.5. 4-Methyl-N-(4-methyl-2-(tert-pentyl)-1H-indol-3-yl)-N-(4-((4-methyl phenyl)sulfonamido)phenyl)benzenesulfonamide (**12-4**)

White solid, 29.25 mg, 95% yield; 93% ee. ^1^H NMR (400 MHz, CDCl3) δ 8.18 (s, 1H), 7.67 (d, *J* = 8.3 Hz, 2H), 7.51 (d, *J* = 8.3 Hz, 2H), 7.24–7.21 (m, 3H), 7.17–7.12 (m, 3H), 7.05–7.01 (m, 1H), 6.88 (d, *J* = 9.2 Hz, 2H), 6.66 (d, *J* = 7.2 Hz, 1H), 6.53 (s, 1H), 2.41 (s, 3H), 2.34 (s, 3H), 1.84–1.58 (m, 2H), 1.41 (s, 3H), 1.36 (s, 3H), 1.27 (s, 3H), 0.65 (t, *J* = 7.4 Hz, 3H). ^13^C NMR (100 MHz, CDCl_3_) δ 144.72, 144.15, 143.86, 142.14, 137.20, 135.88, 134.19, 130.99, 129.64, 129.61, 129.24, 128.45, 127.36, 124.44, 123.40, 122.55, 122.36, 120.06, 111.21, 108.55, 37.62, 37.13, 28.50, 25.07, 21.70, 21.65, 18.60, 9.48. HRMS (ESI) *m*/*z* [M + H]^+^ calcd for C_34_H_38_N_3_O_4_S_2_^+^ 616.2298; found: 616.2301. HPLC conditions: Cel-X (Guangzhou Starchrom Technologies Co., Ltd.); hexane = 0.70 mL/min, isopropanol = 0.30 mL/min; T = 40 °C; λ = 254 nm; t_R_ (major) = 21.2 min, t_R_ (minor) = 30.0 min.

#### 3.2.6. N-(4-methoxy-2-(tert-pentyl)-1H-indol-3-yl)-4-methyl-N-(4-((4-methylphenyl)sulfonamido)phenyl)benzenesulfonamide (**12-5**)

White solid, 29.69 mg, 94% yield; 92% ee. ^1^H NMR (400 MHz, CDCl_3_) δ 8.18 (s, 1H), 7.53 (dd, *J* = 8.4, 2.3 Hz, 4H), 7.40–7.36 (m, 2H), 7.13 (d, *J* = 8.3 Hz, 4H), 7.03 (t, *J* = 8.0 Hz, 1H), 6.91 (d, *J* = 8.0 Hz, 1H), 6.88–6.84 (m, 2H), 6.72 (s, 1H), 6.25 (d, *J* = 7.7 Hz, 1H), 2.85 (s, 3H), 2.38 (s, 3H), 2.34 (s, 3H), 1.77–1.65 (m, 2H), 1.46 (s, 3H), 1.44 (s, 3H), 0.57 (t, *J* = 7.4 Hz, 3H). ^13^C NMR (100 MHz, CDCl_3_) δ 151.94, 143.75, 143.33, 142.91, 141.64, 137.47, 135.95, 134.84, 131.60, 129.54, 128.65, 128.58, 127.39, 122.88, 122.63, 122.57, 116.09, 111.13, 104.08, 99.90, 53.67, 36.92, 36.63, 28.16, 26.43, 21.63, 21.58, 9.34. HRMS (ESI) *m*/*z* [M + Na]^+^ calcd for C_34_H_37_N_3_O_5_S_2_Na^+^ 654.2067; found: 654.2067. HPLC conditions: Cel-D (Guangzhou Starchrom Technologies Co., Ltd.); hexane = 0.80 mL/min, isopropanol = 0.20 mL/min; T = 40 °C; λ = 254 nm; t_R_ (major) = 18.3 min, t_R_ (minor) = 23.0 min.

#### 3.2.7. N-(4-chloro-2-(tert-pentyl)-1H-indol-3-yl)-4-methyl-N-(4-((4-methylphenyl)sulfonamido)phenyl)benzenesulfonamide (**12-6**)

White solid, 29.27 mg, 92% yield; 96% ee. ^1^H NMR (400 MHz, CDCl_3_) δ 8.48 (s, 1H), 7.64 (d, *J* = 8.2 Hz, 2H), 7.49 (d, *J* = 8.2 Hz, 2H), 7.27–7.18 (m, 5H), 7.11 (d, *J* = 8.1 Hz, 2H), 7.02 (t, *J* = 7.9 Hz, 1H), 6.89–6.86 (m, 3H), 6.61 (s, 1H), 2.39 (s, 3H), 2.33 (s, 3H), 1.84–1.59 (m, 2H), 1.44 (s, 3H), 1.36 (s, 3H), 0.63 (t, *J* = 7.4 Hz, 3H). ^13^C NMR (100 MHz, CDCl_3_) δ 146.56, 143.90, 143.79, 142.11, 136.76, 135.77, 135.13, 131.06, 129.59, 129.34, 128.67, 127.36, 123.96, 123.33, 122.81, 122.75, 121.80, 120.49, 110.50, 109.69, 37.28, 37.19, 28.30, 25.39, 21.70, 21.64, 9.43. HRMS (ESI) *m*/*z* [M + Na]^+^ calcd for C_33_H_34_ClN_3_O_4_S_2_Na^+^ 658.1571; found: 658.1575. HPLC conditions: Cel-X (Guangzhou Starchrom Technologies Co., Ltd.); hexane = 0.70 mL/min, isopropanol = 0.30 mL/min; T = 40 °C; λ = 254 nm; t_R_ (major) = 27.7 min, t_R_ (minor) = 41.4 min.

#### 3.2.8. N-(2-(adamantan-1-yl)-4-methyl-1H-indol-3-yl)-4-methyl-N-(4-((4-methylphenyl) sulfonamido)phenyl)benzenesulfonamide (**12-7**)

White solid, 32.30 mg, 95% yield; 95% ee. ^1^H NMR (400 MHz, CDCl3) δ 8.28 (s, 1H), 7.68 (d, *J* = 8.3 Hz, 2H), 7.52 (d, *J* = 8.3 Hz, 2H), 7.24–7.20 (m, 4H), 7.15–7.11 (m, 3H), 7.04–7.01 (m, 1H), 6.90–6.87 (m, 2H), 6.66 (d, *J* = 7.2 Hz, 1H), 6.57 (s, 1H), 2.41 (s, 3H), 2.34 (s, 3H), 2.01–1.96 (m, 9H), 1.68–1.61 (m, 9H), 1.45 (s, 3H). ^13^C NMR (100 MHz, CDCl_3_) δ 145.64, 144.13, 143.86, 142.39, 137.24, 135.92, 134.01, 130.94, 129.65, 129.59, 129.45, 128.49, 127.36, 124.57, 123.42, 122.50, 122.33, 120.14, 109.62, 108.56, 41.15, 36.55, 36.04, 28.42, 21.68, 21.64, 18.70. HRMS (ESI) *m*/*z* [M + Na]^+^ calcd for C_39_H_41_N_3_O_4_S_2_Na^+^ 702.2431; found: 702.2432. HPLC conditions: IMB (Guangzhou Starchrom Technologies Co., Ltd.); hexane = 0.70 mL/min, isopropanol = 0.30 mL/min; T = 40 °C; λ = 254 nm; t_R_ (major) = 9.9 min, t_R_ (minor) = 16.5 min.

#### 3.2.9. N-(2-(adamantan-1-yl)-4-methoxy-1H-indol-3-yl)-4-methyl-N-(4-((4-methylphenyl) sulfonamido)phenyl)benzenesulfonamide (**12-8**)

White solid, 32.71 mg, 94% yield; 91% ee. ^1^H NMR (400 MHz, CDCl_3_) δ 8.32 (s, 1H), 7.54 (dd, *J* = 8.3, 1.7 Hz, 4H), 7.34 (t, *J* = 6.1 Hz, 2H), 7.14–7.11 (m, 4H), 7.01 (t, *J* = 7.9 Hz, 1H), 6.91–6.85 (m, 3H), 6.79 (s, 1H), 6.22 (d, *J* = 7.8 Hz, 1H), 2.85 (s, 3H), 2.38 (s, 3H), 2.34 (s, 3H), 2.17–2.02 (m, 8H), 1.73–1.71 (m, 7H). ^13^C NMR (100 MHz, CDCl_3_) δ 152.03, 144.54, 143.74, 142.90, 141.81, 137.58, 135.98, 134.72, 131.53, 129.51, 128.68, 128.57, 127.38, 122.82, 122.61, 122.53, 115.92, 109.87, 104.12, 99.80, 53.64, 41.23, 36.63, 35.70, 28.49, 21.61, 21.57. HRMS (ESI) *m*/*z* [M + Na]^+^ calcd for C_39_H_41_N_3_O_5_S_2_Na^+^ 718.2380; found: 718.2382. HPLC conditions: IMB (Guangzhou Starchrom Technologies Co., Ltd.); hexane = 0.70 mL/min, isopropanol = 0.30 mL/min; T = 40 °C; λ = 254 nm; t_R_ (major) = 11.1 min, t_R_ (minor) = 15.5 min.

#### 3.2.10. N-(2-(adamantan-1-yl)-4-chloro-1H-indol-3-yl)-4-methyl-N-(4-((4-methylphenyl) sulfonamido)phenyl)benzenesulfonamide (**12-9**)

White solid, 32.21 mg, 92% yield; 98% ee. ^1^H NMR (400 MHz, CDCl3) δ 8.62 (s, 1H), 7.65 (d, *J* = 8.3 Hz, 2H), 7.49 (d, *J* = 8.2 Hz, 2H), 7.23–7.19 (m, 5H), 7.10 (d, *J* = 8.1 Hz, 2H), 7.00 (t, *J* = 7.9 Hz, 1H), 6.89–6.85 (m, 3H), 6.66 (s, 1H), 2.40 (s, 3H), 2.32 (s, 3H), 2.12–1.96 (m, 9H), 1.72–1.66 (m, 6H). ^13^C NMR (100 MHz, CDCl_3_) δ 147.51, 143.88, 143.78, 142.34, 136.89, 135.79, 135.01, 131.01, 129.56, 129.38, 128.66, 127.34, 124.09, 123.33, 122.72, 121.70, 120.54, 109.71, 109.02, 41.02, 36.49, 36.15, 28.38, 21.69, 21.64. HRMS (ESI) *m*/*z* [M + Na]^+^ calcd for C_38_H_38_ClN_3_O_4_S_2_Na^+^ 722.1884; found: 722.1887. HPLC conditions: IMB (Guangzhou Starchrom Technologies Co., Ltd.); hexane = 0.70 mL/min, isopropanol = 0.30 mL/min; T = 40 °C; λ = 254 nm; t_R_ (major) = 10.1 min, t_R_ (minor) = 17.9 min.

#### 3.2.11. N-(2-(tert-butyl)-1H-indol-3-yl)-4-methyl-N-(4-((4-methylphenyl)sulfonamido) phenyl)benzenesulfonamide (**12-10**)

White solid, 28.80 mg, 98% yield; 98% ee. ^1^H NMR (400 MHz, DMSO-*d*_6_) δ 11.04 (s, 1H), 10.16 (s, 1H), 7.58 (d, *J* = 8.2 Hz, 2H), 7.38–7.25 (m, 9H), 7.00 (t, *J* = 8.1 Hz, 3H), 6.72 (t, *J* = 7.5 Hz, 1H), 6.31 (d, *J* = 8.0 Hz, 1H), 2.38 (s, 3H), 2.31 (s, 3H), 1.34 (s, 9H). ^13^C NMR (100 MHz, DMSO-*d*_6_) δ 145.30, 143.89, 143.19, 139.03, 137.21, 136.49, 134.82, 133.24, 129.55, 129.52, 127.51, 126.77, 125.64, 124.08, 120.98, 120.86, 119.13, 118.04, 111.53, 110.80, 32.86, 29.84, 21.05, 20.97. HRMS (ESI) Calcd for C_32_H_34_N_3_O_4_S_2_^+^ [M + H]^+^ 588.1985; Found: 588.1992. HPLC conditions: IMB (Guangzhou Starchrom Technologies Co., Ltd.); hexane = 0.80 mL/min, isopropanol = 0.20 mL/min; T = 40 °C; λ = 254 nm; t_R_ (major) = 19.2 min, t_R_ (minor) = 21.7 min.

#### 3.2.12. N-(2-((3r,5r,7r)-adamantan-1-yl)-1H-indol-3-yl)-4-methyl-N-(4-((4-methylphenyl) sulfonamido)phenyl)benzenesulfonamide (**12-11**)

White solid, 31.63 mg, 95% yield; 94% ee. ^1^H NMR (400 MHz, DMSO-*d*_6_) δ 10.99 (s, 1H), 10.16 (s, 1H), 7.57 (d, *J* = 8.1 Hz, 2H), 7.40–7.24 (m, 9H), 6.99 (t, *J* = 8.6 Hz, 3H), 6.73 (t, *J* = 7.5 Hz, 1H), 6.37 (d, *J* = 8.0 Hz, 1H), 2.39 (s, 3H), 2.31 (s, 3H), 1.98–1.96 (m, 9H), 1.71–1.62 (m, 6H). ^13^C NMR (100 MHz, DMSO-*d*_6_) δ 145.18, 143.90, 143.20, 139.25, 137.17, 136.44, 134.79, 133.19, 129.52, 127.57, 126.78, 125.85, 124.25, 120.92, 120.82, 119.11, 118.11, 111.58, 110.82, 40.49, 36.12, 35.09, 27.83, 21.06, 20.97. HRMS (ESI) Calcd for C_38_H_40_N_3_O_4_S_2_^+^ [M + H]^+^ 666.2455; Found: 666.2455. HPLC conditions: IMB (Guangzhou Starchrom Technologies Co., Ltd.); hexane = 0.70 mL/min, isopropanol = 0.30 mL/min; T = 40 °C; λ = 254 nm; t_R_ (major) = 9.5 min, tR (minor) = 12.8 min.

## 4. Conclusions

In summary, a new type of CPAs derived from the *O*-SPINOL scaffold has been synthesized. This synthetic route is concise and operationally efficient. We preliminarily applied the CPAs to the asymmetric construction of atropochiral *N*-sulfonyl-3-arylaminoindoles, in which they showed outstanding catalytic activity. This new type of CPAs serves as an enrichment and complement to the existing library of chiral phosphoric acids. Ongoing efforts in our laboratory are directed toward exploring the applications of the above CPAs in asymmetric synthesis and medicinal chemistry.

## Figures and Tables

**Table 1:** Optimization of the reaction conditions ^a^.

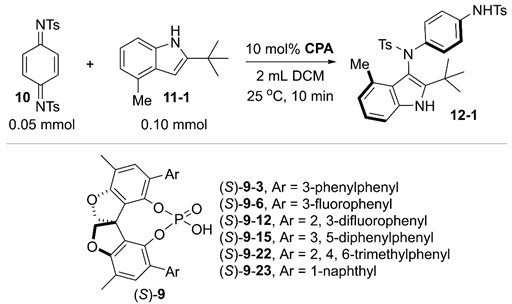			
Entry	**CPA**	Ee/% ^b^	Yield/% ^c^
1	(*S*)-**9-3**	79	95
2	(*S*)-**9-6**	71	95
3	(*S*)-**9-12**	74	90
4	(*S*)-**9-15**	75	95
5	(*S*)-**9-22**	84	96
6	(*S*)-**9-23**	79	92
7 ^d^	(*S*)-**9-22**	83	96
8 ^e^	(*S*)-**9-22**	79	95
9 ^f^	(*S*)-**9-22**	84	97
10 ^g^	(*S*)-**9-22**	97	96
11 ^h^	(*S*)-**9-22**	97	98

^a^ Reactions were performed with **10** (0.05 mmol), **11-1** (0.10 mmol), and **CPA** (10 mol%) in DCM (2.0 mL) at 25 °C for 10 min. ^b^ Determined by HPLC analysis on a chiral stationary phase. ^c^ Isolated yield; ^d^ 2 mL DCE was used; ^e^ 2 mL CHCl_3_ was used; ^f^ 1 mL DCM was used; ^g^ 1 mL DCM was used. The reaction was conducted at −20 °C for 4 h. ^h^ (S)-**9-22** (0.2 mol%) and 1 mL DCM was used. The reaction was conducted at −20 °C for 12 h.

## Data Availability

The data supporting this work is in the article and Supplementary Materials.

## Supplementary Materials

The following supporting information can be downloaded at: https://www.mdpi.com/article/10.3390/molecules31213858/s1. Experimental procedures for synthesis of (*S*)-**9**; reaction condition screening for the application of the CPAs in the asymmetric construction of atropochiral N-sulfonyl-3-arylaminoindoles; X-ray single-crystal diffraction data; copies of HPLC and NMR spectra.
