# Supplementary material for: Synthesis and Application of New Chiral Phosphoric Acids Based on oxa-Spirocyclic Diphenol
Source: Molecules. 2026 Oct 26;31(21):3858. doi: 10.3390/molecules31213858 (PMC13765076; doi:10.3390/molecules31213858)
Supplement: Supplementary file 1 [file molecules-31-03858-s001.zip › molecules-4607971-supplementary.pdf]

Supporting Information

Synthesis and Application of New Chiral Phosphoric Acids Based on *oxa*-Spirocyclic Diphenol

Jia-Jie Chen,[1†] Zhuo-Ting Chen,[1†] Peng Wang,[1] Wen-Jing Wang,[1] Li-Guo Shen,[1] Zhi-Yuan Bai,[1] Si-Hai Zhou,[1] Zehua Yang,[1] and Ye-Hui Chen[1]*

[1]School of Pharmaceutical Science, Hengyang Medical School, University of South China, Hengyang 421001, China

[†]These authors contributed equally.
*Corresponding author, E-mail: chenyh@usc.edu.cn.

List of Content

1. General information..3

2. Synthesis of chiral phosphoric acids (S)-**9**..4

3. Synthesis of QDI **10**...37

4. Synthesis of indoles **11**...37

5. Screening of conditions for the electrophilic amination of indoles................42

6. General procedure for asymmetric synthesis of axially chiral 3-arylamino-indoles **12**..45

7. Determination of the absolute configuration of the products.......................61

8. Scale-up experiment..63

9. Single-crystal X-ray diffraction data..64

10. Copies of NMR spectra..68

1. General information

All commercial reagents and solvents were purchased from suppliers and used without further purification. Analytical thin layer chromatography (TLC) was performed on precoated silica gel 60 F254 plates. Visualization on TLC was achieved by use of UV light (254 nm). Purification by flash column chromatography was performed on Tsingdao silica gel (300−400 mesh). NMR spectra were recorded in $CDCl_3$ or DMSO-d_6 on a Bruker DPX 400 or 500 NMR spectrometer. Chemical shifts (δ) are reported in ppm relative to the residual solvent peak. Coupling constants (*J*) are given in Hertz (Hz), and multiplicities are denoted as s (singlet), d (doublet), t (triplet), q (quartet), dd (double of doublet) and m (multiplet). High-resolution mass spectra (HRMS) were acquired using an Agilent 1260 Series time-of-flight (TOF) electrospray ionization (ESI) mass spectrometer. Enantioselectivities were recorded on Shimadzu HPLC using chiral columns from Guangzhou Starchrom Technologies Co., Ltd.

2. Synthesis of chiral phosphoric acids (*S*)-9

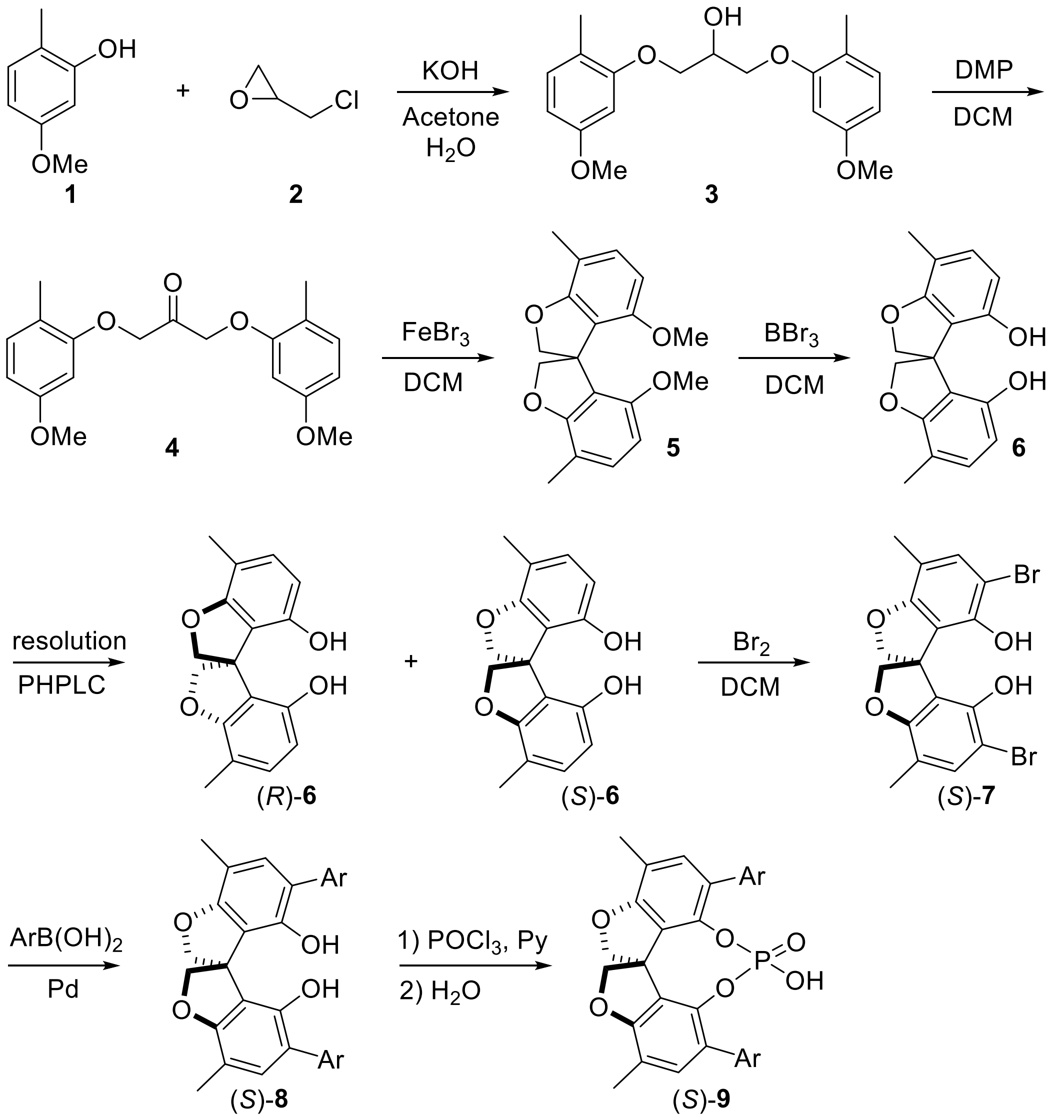

Synthesis of (*S*)-6

(*S*)-**6** was synthesized according to the reference (*Synthesis*, **2026**, *58*, 2114-2116.).

Synthesis of (*S*)-7

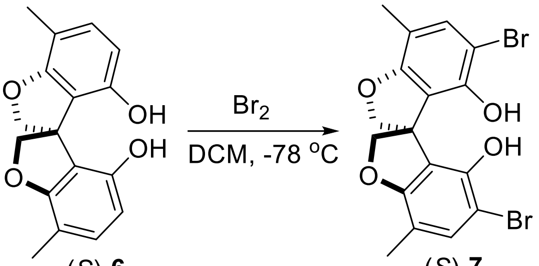

The solution of (*S*)-**6** (35.17 mmol, 10.00 g) in dichloromethane (150 mL) was stirred at −78 °C for 10 min, and then Br_2 (77.38 mmol, 3.96 mL) was slowly added. The reaction mixture was stirred at −78 °C for 40 min. Upon complete consumption of the starting material monitored by thin-layer chromatographic (TLC), the reaction was quenched with saturated aqueous sodium thiosulfate solution (50 mL). The organic layer was separated and washed with H_2O (50 mL × 3), dried over anhydrous sodium sulfate, and concentrated under reduced pressure to afford product (*S*)-**7**.

White solid, 14.6 g, 94% yield.

^{1}H NMR (500 MHz, $CDCl_3$) δ 7.12 (s, 2H), 5.33 (s, 2H), 4.85 (d, *J* = 9.2 Hz, 2H), 4.58 (d, *J* = 9.2 Hz, 2H), 2.17 (s, 6H).

^{13}C NMR (125 MHz, $CDCl_3$) δ 159.07, 146.95, 133.08, 114.52, 113.97, 100.84, 81.54, 55.81, 14.30.

HRMS (ESI): m/z [M-H]$^-$ calcd for $C_{17}H_{13}Br_2O_4^-$: 438.9186; found: 438.9192.

HPLC conditions: IMB (Guangzhou Starchrom Technologies Co., Ltd); hexane = 0.95 mL/min, isopropanol = 0.05 mL/min; T = 40 °C; λ = 254 nm; t_R (major) = 7.2 min, t_R (minor) = 8.1 min.

HPLC chromatogram of racemic **7**

<Chromatogram>

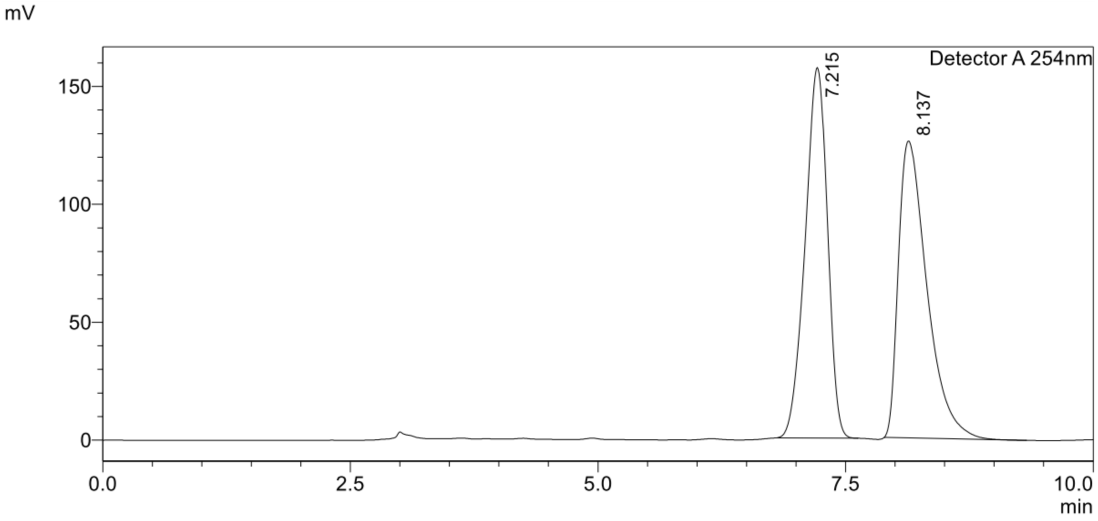

<Peak Table>

Detector A 254nm

Peak#	Ret. Time	Area	Height	Conc.	Unit	Mark	Name
1	7.215	2462430	157124	49.457		M	
2	8.137	2516464	125901	50.543		M	
Total		4978894	283024				

HPLC chromatogram of (*S*)-**7**

<Chromatogram>

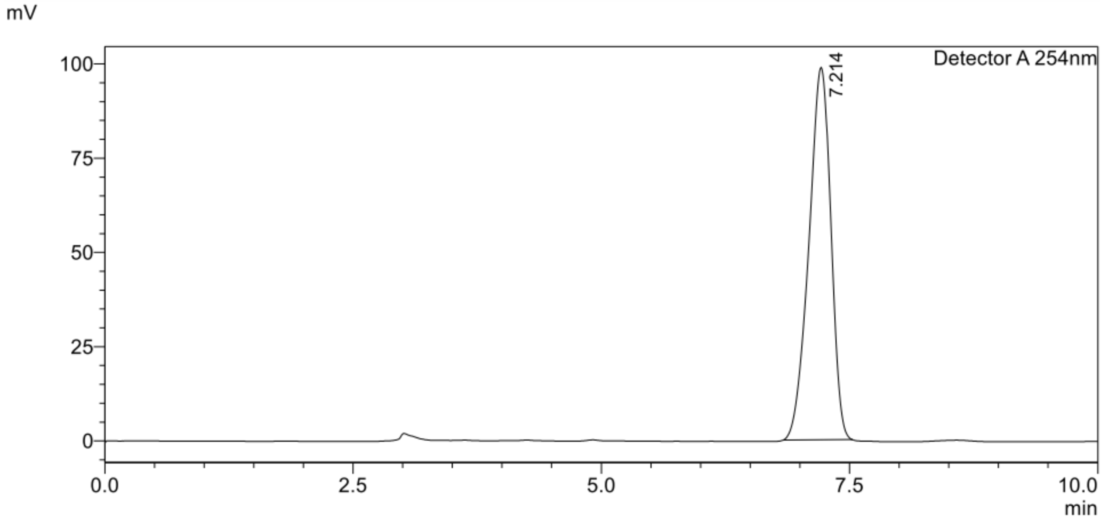

<Peak Table>

Detector A 254nm

Peak#	Ret. Time	Area	Height	Conc.	Unit	Mark	Name
1	7.214	1516041	98729	100.000		M	
Total		1516041	98729				

Synthesis of (*S*)-8

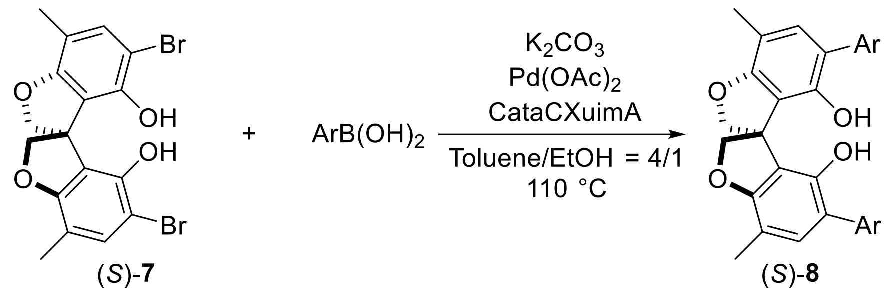

(*S*)-**7** (*S*)-**8**

At 25 °C, to the mixture of (*S*)-**7** (2.0 mmol, 0.88 g), arylboronic acid (8.0 mmol), potassium carbonate (8.0 mmol, 1.11 g), $Pd(OAc)_2$ (0.2 mmol, 0.04 g) and CataCXium A (0.8 mmol, 0.29 g) were added degassed toluene/ethanol (12.0 mL/3.0 mL) under N_2 atmosphere. The reaction mixture was stirred at 110 °C for 12 h. TLC analysis indicated full consumption of (*S*)-**7**, the reaction mixture was cooled to room temperature and then 3 M HCl (25 mL) was added. The mixture was extracted with dichloromethane (20 mL × 3). The combined organic extracts were washed with H_2O (20 mL × 3) and dried over anhydrous sodium sulfate. Solvent was evaporated under reduced pressure and the residue was purified by column chromatography on silica gel (PE/EA = 20/1, V/V) to give the product (*S*)-**8**.

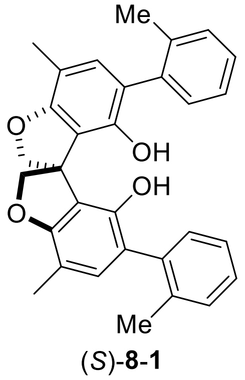

(*S*)-**8**-**1**

Yellow solid, 0.59 g, 64% yield.

^{1}H NMR (500 MHz, $CDCl_3$) δ 7.26 – 7.22 (m, 8H), 6.79 (s, 2H), 4.90 – 4.89 (m, 2H), 4.67 – 4.66 (m, 4H), 2.19 (s, 6H), 2.08 (s, 6H).

^{13}C NMR (125 MHz, $CDCl_3$) δ 158.67, 147.66, 137.56, 135.74, 132.20, 130.89, 130.37, 128.07, 126.23 (2C), 121.37, 111.78, 81.29, 55.24, 29.70, 14.37.

HRMS (ESI): m/z $[M-H]^-$ calcd for $C_{31}H_{27}O_4^-$: 463.1915; found: 463.1913.

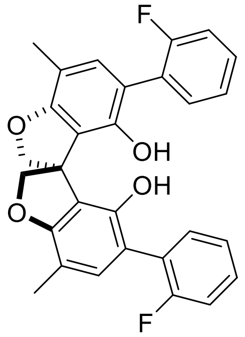

(S)-**8-2**

Yellow solid, 0.65 g, 69% yield.

^{1}H NMR (500 MHz, $CDCl_3$) δ 7.34 – 7.30 (m, 4H), 7.19 – 7.12 (m, 4H), 6.95 (s, 2H), 5.02 (s, 2H), 4.95 (d, J = 9.2 Hz, 2H), 4.65 (d, J = 9.2 Hz, 2H), 2.21 (s, 6H).

^{13}C NMR (125 MHz, $CDCl_3$) δ 159.90, 158.03, 157.94, 147.11, 132.38, 131.04, 131.01, 128.40, 128.34, 123.45, 123.42, 123.37, 123.24, 115.10, 114.96, 114.92, 112.99, 111.58, 79.67, 54.08, 13.34.

^{19}F NMR (471 MHz, $CDCl_3$) δ -114.20.

HRMS (ESI): m/z [M-H]$^-$ calcd for $C_{29}H_{21}F_2O_4^-$: 471.1413; found: 471.1400.

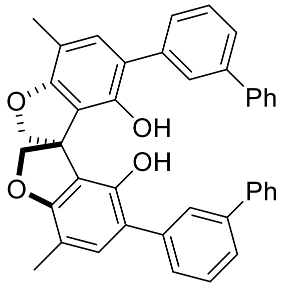

(S)-**8-3**

Yellow solid, 0.65 g, 55% yield.

^{1}H NMR (500 MHz, $CDCl_3$) δ 7.63 – 7.35 (m, 18H), 7.02 (s, 2H), 5.30 (s, 2H), 5.00 (d, J = 9.2 Hz, 2H), 4.66 (d, J = 9.2 Hz, 2H), 2.23 (s, 6H).

^{13}C NMR (125 MHz, $CDCl_3$) δ 158.77, 147.71, 142.11, 140.85, 137.45, 132.67, 129.37, 128.80, 128.05, 127.95, 127.48, 127.23, 126.07, 121.89, 114.05, 112.56, 80.93, 55.20, 14.39.

HRMS (ESI): m/z [M-H]$^-$ calcd for $C_{41}H_{31}O_4^-$: 587.2228; found: 587.2219.

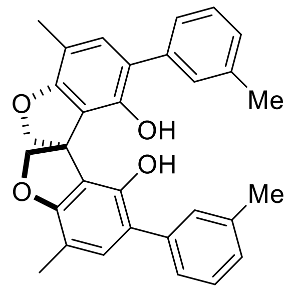

(*S*)-**8-4**

Yellow solid, 0.67 g, 72% yield.

^{1}H NMR (500 MHz, $CDCl_3$) δ 7.31 – 7.28 (m, 2H), 7.22 – 7.19 (m, 4H), 7.13 – 7.12 (m, 2H), 6.95 (s, 2H), 5.28 (s, 2H), 4.97 (d, *J* = 9.2 Hz, 2H), 4.64 (d, *J* = 9.2 Hz, 2H), 2.36 (s, 6H), 2.21 (s, 6H).

^{13}C NMR (125 MHz, $CDCl_3$) δ 158.73, 147.63, 138.78, 136.84, 132.47, 129.75, 128.96, 128.02, 126.13, 121.91, 113.92, 112.27, 81.14, 55.21, 21.45, 14.38.

HRMS (ESI): m/z $[M-H]^-$ calcd for $C_{31}H_{27}O_4^-$: 463.1915; found: 463.1910.

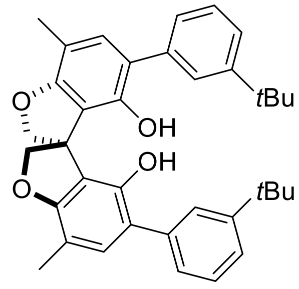

(*S*)-**8-5**

Yellow solid, 0.85 g, 78% yield.

^{1}H NMR (500 MHz, $CDCl_3$) δ 7.40 (s, 2H), 7.35 – 7.34 (m, 4H), 7.22 – 7.21 (m, 2H), 6.96 (s, 2H), 5.27 (s, 2H), 4.99 (d, *J* = 9.2 Hz, 2H), 4.65(d, *J* = 9.2 Hz, 2H), 2.23 (s, 6H), 1.33 (s, 18H).

^{13}C NMR (125 MHz, $CDCl_3$) δ 158.74, 152.14, 147.69, 136.50, 132.53, 128.71, 126.18, 124.36, 122.32, 113.91, 112.22, 81.08, 55.26, 34.82, 31.39, 14.40.

HRMS (ESI): m/z $[M-H]^-$ calcd for $C_{37}H_{39}O_4^-$: 547.2854; found: 547.2851.

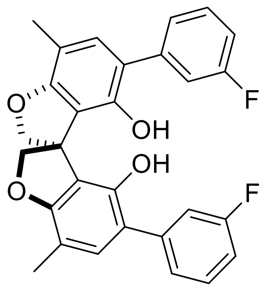

(S)-**8-6**

Light yellow solid, 0.58 g, 61% yield.

^{1}H NMR (500 MHz, $CDCl_3$) δ 7.38 – 7.34 (m, 2H), 7.21 (d, J = 9.9 Hz, 2H), 7.15 (d, J = 9.9 Hz, 2H), 7.02 – 6.98 (m, 2H), 6.97 (s, 2H), 5.22 (s, 2H), 4.95 (d, J = 9.2 Hz, 2H), 4.64 (d, J = 9.2 Hz, 2H), 2.22 (s, 6H).

^{13}C NMR (125 MHz, $CDCl_3$) δ 164.08, 162.12, 158.84, 147.58, 139.27, 139.21, 132.72, 130.38, 130.32, 124.64, 124.62, 120.96, 120.94, 116.25, 116.08, 114.15, 114.05, 113.99, 112.86, 80.63, 55.08, 14.34.

^{19}F NMR (471 MHz, $CDCl_3$) δ -112.30.

HRMS (ESI): m/z $[M-H]^-$ calcd for $C_{29}H_{21}F_2O_4^-$: 471.1413; found: 471.1416.

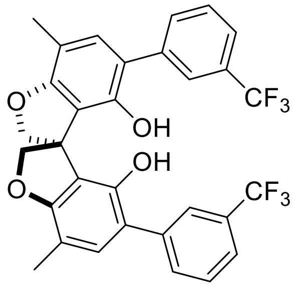

(S)-**8-7**

Light yellow solid, 0.69 g, 60% yield.

^{1}H NMR (500 MHz, $CDCl_3$) δ 7.70 (s, 2H), 7.63 (d, J = 7.6 Hz, 2H), 7.57 (d, J = 7.8 Hz, 2H), 7.53 – 7.49 (m, 2H), 6.99 (s, 2H), 5.15 (s, 2H), 4.96 (d, J = 9.2 Hz, 2H), 4.65 (d, J = 9.2 Hz, 2H), 2.23 (s, 6H).

^{13}C NMR (125 MHz, $CDCl_3$) δ 158.75, 147.60, 137.94, 133.07, 132.47, 131.31, 131.06, 129.10, 126.02, 125.99, 125.14, 123.81, 123.78, 122.98, 121.10, 114.08, 113.29, 80.21, 55.03, 14.29.

^{19}F NMR (471 MHz, $CDCl_3$) δ -62.61.

HRMS (ESI): m/z $[M-H]^-$ calcd for $C_{31}H_{21}F_6O_4^-$: 571.1350; found: 571.1350.

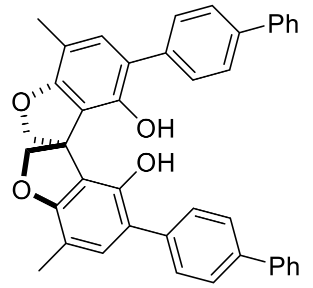

(S)-**8-8**

Light yellow solid, 0.97 g, 82% yield.

^{1}H NMR (500 MHz, $CDCl_3$) δ 7.65 (d, J = 8.0 Hz, 4H), 7.62 (d, J = 7.6 Hz, 4H), 7.50 (d, J = 8.0 Hz, 4H), 7.45 (t, J = 7.6 Hz, 4H), 7.36 (t, J = 7.4 Hz, 2H), 7.03 (s, 2H), 5.30 (s, 2H), 4.98 (d, J = 9.2 Hz, 2H), 4.66 (d, J = 9.2 Hz, 2H), 2.25 (s, 6H).

^{13}C NMR (125 MHz, $CDCl_3$) δ 158.72, 147.74, 140.65, 140.07, 135.95, 132.66, 129.51, 128.84, 127.66, 127.40, 127.06, 121.66, 114.03, 112.63, 80.81, 55.20, 14.41.

HRMS (ESI): m/z $[M-H]^-$ calcd for $C_{41}H_{31}O_4^-$: 587.2228; found: 587.2220.

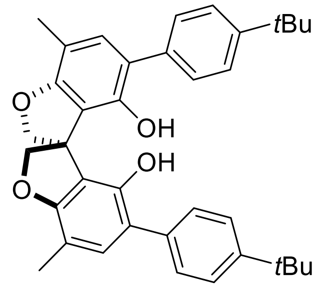

(S)-**8-9**

White solid, 0.82 g, 75% yield.

^{1}H NMR (500 MHz, $CDCl_3$) δ 7.42 (d, J = 8.4 Hz, 4H), 7.32 (d, J = 8.3 Hz, 4H), 6.95 (s, 2H), 5.25 (s, 2H), 4.95 (d, J = 9.1 Hz, 2H), 4.62 (d, J = 9.2 Hz, 2H), 2.20 (s, 6H), 1.32 (s, 18H).

^{13}C NMR (125 MHz, $CDCl_3$) δ 158.49, 150.17, 147.71, 133.90, 132.46, 128.77, 125.90, 121.85, 113.95, 112.23, 80.85, 55.18, 34.55, 31.32, 14.39.

HRMS (ESI): m/z $[M-H]^-$ calcd for $C_{37}H_{39}O_4^-$: 547.2854; found: 547.2857.

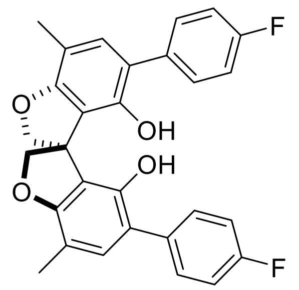

(S)-**8-10**

White solid, 0.80 g, 85% yield.

^{1}H NMR (500 MHz, $CDCl_3$) δ 7.39 – 7.36 (m, 4H), 7.11 – 7.07 (m, 4H), 6.93 (s, 2H), 5.13 (d, J = 2.4 Hz, 2H), 4.95 (d, J = 9.2 Hz, 2H), 4.63 (d, J = 9.3 Hz, 2H), 2.21 (s, 6H).

^{13}C NMR (125 MHz, $CDCl_3$) δ 163.07, 161.11, 158.51, 147.53, 132.88, 132.85, 132.78, 130.88, 130.82, 121.20, 115.87, 115.70, 114.01, 112.69, 80.55, 55.08, 14.35.

^{19}F NMR (471 MHz, $CDCl_3$) δ -115.04.

HRMS (ESI): m/z $[M-H]^-$ calcd for $C_{29}H_{21}F_2O_4^-$: 471.1413; found: 471.1417.

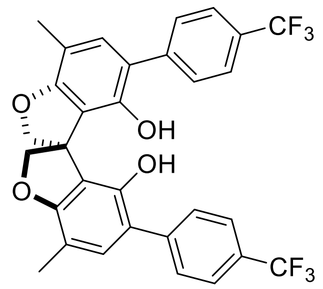

(S)-**8-11**

Light yellow solid, 0.87 g, 76% yield.

^{1}H NMR (500 MHz, $CDCl_3$) δ 7.65 (d, J = 8.1 Hz, 4H), 7.56 (d, J = 8.1 Hz, 4H), 6.99 (s, 2H), 5.19 (s, 2H), 4.95 (d, J = 9.3 Hz, 2H), 4.65 (d, J = 9.4 Hz, 2H), 2.22 (s, 6H).

^{13}C NMR (125 MHz, $CDCl_3$) δ 158.82, 147.62, 140.83, 129.44, 125.71, 125.68, 125.65, 125.62, 121.10, 114.16, 113.29, 80.14, 54.99, 14.35.

^{19}F NMR (471 MHz, $CDCl_3$) δ -62.51.

HRMS (ESI): m/z $[M-H]^-$ calcd for $C_{31}H_{21}F_6O_4^-$: 571.1350; found: 571.1352.

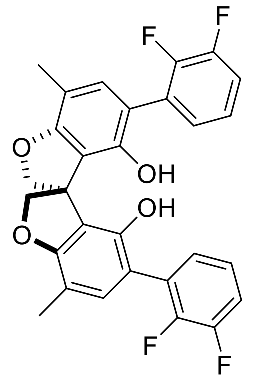

(*S*)-**8**-**12**

Light yellow solid, 0.54 g, 53% yield.

^{1}H NMR (500 MHz, $CDCl_3$) δ 7.15 – 7.08 (m, 6H), 6.95 (s, 2H), 5.04 (s, 2H), 4.94 (d, *J* = 9.3 Hz, 2H), 4.65 (d, *J* = 9.3 Hz, 2H), 2.20 (s, 6H).

^{13}C NMR (125 MHz, $CDCl_3$) δ 159.14, 158.43, 152.15, 152.04, 151.98, 150.17, 150.06, 149.18, 149.08, 148.07, 147.11, 146.07, 133.52, 126.90, 126.80, 126.54, 126.51, 126.49, 124.21, 124.18, 124.16, 124.12, 116.50, 116.36, 115.10, 115.08, 114.04, 112.97, 80.32, 55.02, 14.33.

^{19}F NMR (471 MHz, $CDCl_3$) δ -137.23 (d, *J* = 21.5 Hz), -139.18 (d, *J* = 21.2 Hz).

HRMS (ESI): m/z $[M-H]^-$ calcd for $C_{29}H_{19}F_4O_4^-$: 507.1225; found: 507.1228.

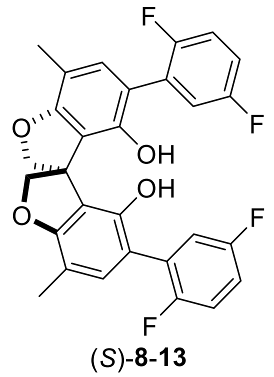

(*S*)-**8**-**13**

Light yellow solid, 0.50 g, 49% yield.

^{1}H NMR (500 MHz, $CDCl_3$) δ 7.11 – 7.03 (m, 4H), 7.01 – 6.97 (m, 2H), 6.94 (s, 2H), 5.04 (s, 2H), 4.93 (d, *J* = 9.3 Hz, 2H), 4.65 (d, *J* = 9.3 Hz, 2H), 2.21 (s, 6H).

^{13}C NMR (125 MHz, $CDCl_3$) δ 159.60, 159.18, 157.65, 156.86, 154.92, 148.06, 133.49, 126.01, 125.87, 118.42, 118.39, 118.23, 118.20, 117.06, 116.99, 116.85, 116.78, 115.72, 115.66, 115.53, 115.47, 115.38, 114.11, 113.04, 80.37, 55.02, 14.33.

^{19}F NMR (471 MHz, $CDCl_3$) δ -118.66 (d, *J* = 17.4 Hz), -120.39 (d, *J* = 17.4 Hz).

HRMS (ESI): m/z $[M-H]^-$ calcd for $C_{29}H_{19}F_4O_4^-$: 507.1225; found: 507.1226.

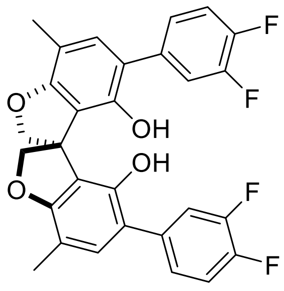

(*S*)-**8-14**

Light yellow solid, 0.55 g, 54% yield.

^{1}H NMR (500 MHz, $CDCl_3$) δ 7.28 – 7.24 (m, 2H), 7.19 – 7.14 (m, 4H), 6.94 (s, 2H), 5.16 (s, 2H), 4.93 (d, *J* = 9.3 Hz, 2H), 4.63 (d, *J* = 9.3 Hz, 2H), 2.21 (s, 6H).

^{13}C NMR (125 MHz, $CDCl_3$) δ 158.56, 151.38, 151.28, 150.62, 150.52, 149.40, 149.30, 148.64, 148.54, 147.47, 134.02, 133.99, 133.97, 133.94, 132.88, 125.17, 125.14, 125.12, 125.09, 120.46, 118.36, 118.22, 117.55, 117.42, 114.09, 113.12, 80.14, 54.98, 14.31.

^{19}F NMR (471 MHz, $CDCl_3$) δ -137.03 (d, *J* = 21.6 Hz), -139.72 (d, *J* = 21.5 Hz).

HRMS (ESI): m/z $[M-H]^-$ calcd for $C_{29}H_{19}F_4O_4^-$: 507.1225; found: 507.1228.

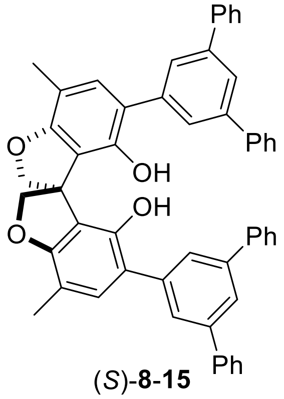

(*S*)-**8-15**

White solid, 1.27 g, 86% yield.

^{1}H NMR (500 MHz, $CDCl_3$) δ 7.75 (s, 2H), 7.65 – 7.63 (m, 12H), 7.45 (t, *J* = 7.6 Hz, 8H), 7.37 (t, *J* = 7.3 Hz, 4H), 7.09 (s, 2H), 5.35 (s, 2H), 5.04 (d, *J* = 9.2 Hz, 2H), 4.69 (d, *J* = 9.2 Hz, 2H), 2.25 (s, 6H).

^{13}C NMR (125 MHz, $CDCl_3$) δ 158.87, 147.80, 142.61, 140.85, 137.98, 132.74, 128.84, 127.60, 127.33, 126.92, 125.06, 121.84, 114.13, 112.69, 80.96, 55.24, 14.39.

HRMS (ESI): m/z $[M-H]^-$ calcd for $C_{53}H_{39}O_4^-$: 739.2854; found: 739.2858.

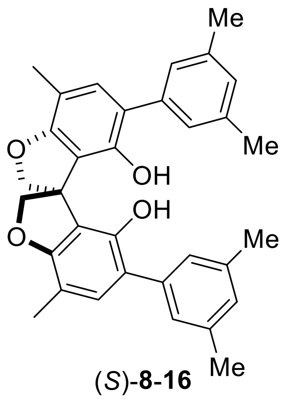

(*S*)-**8-16**

Light yellow solid, 0.76 g, 77% yield.

^{1}H NMR (500 MHz, $CDCl_3$) δ 7.02 (s, 4H), 6.95 – 6.94 (m, 4H), 5.31 (s, 2H), 4.95 (d, *J* = 9.1 Hz, 2H), 4.64 (d, *J* = 9.1 Hz, 2H), 2.32 (s, 12H), 2.21 (s, 6H).

^{13}C NMR (125 MHz, $CDCl_3$) δ 158.74, 147.64, 138.76, 136.76, 132.32, 128.93, 121.86, 113.88, 112.12, 81.31, 55.23, 21.33, 14.40.

HRMS (ESI): m/z $[M-H]^-$ calcd for $C_{33}H_{31}O_4^-$: 491.2228; found: 491.2228.

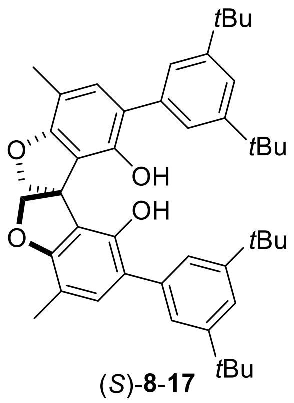

(*S*)-**8-17**

Light yellow solid, 0.98 g, 74% yield.

^{1}H NMR (500 MHz, $CDCl_3$) δ 7.38 (t, *J* = 1.8 Hz, 2H), 7.21 (d, *J* = 1.8 Hz, 4H), 6.95 (s, 2H), 5.29 (s, 2H), 4.99 (d, *J* = 9.1 Hz, 2H), 4.65 (d, *J* = 9.1 Hz, 2H), 2.23 (s, 6H), 1.32 (s, 36H).

^{13}C NMR (125 MHz, $CDCl_3$) δ 158.75, 151.65, 147.75, 135.96, 132.45, 123.31, 122.72, 121.46, 113.83, 111.99, 81.13, 55.34, 34.96, 31.49, 14.41.

HRMS (ESI): m/z $[M-H]^-$ calcd for $C_{45}H_{55}O_4^-$: 659.4106; found: 659.4106.

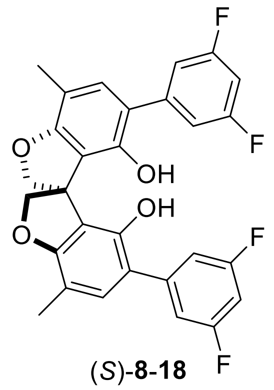

(*S*)-**8-18**

Light yellow solid, 0.54 g, 53% yield.

^{1}H NMR (500 MHz, $CDCl_3$) δ 7.00 – 6.97 (m, 6H), 6.77 – 6.73 (m, 2H), 5.21 (s, 2H), 4.94 (d, *J* = 9.3 Hz, 2H), 4.63 (d, *J* = 9.3 Hz, 2H), 2.22 (s, 6H).

^{13}C NMR (125 MHz, $CDCl_3$) δ 164.23, 164.13, 162.25, 162.15, 158.91, 147.54, 140.32, 132.80, 120.25, 114.16, 113.30, 112.05, 112.00, 111.90, 111.85, 102.67, 102.47, 102.27, 80.20, 54.95, 14.33.

^{19}F NMR (471 MHz, $CDCl_3$) δ -109.32.

HRMS (ESI): m/z $[M-H]^-$ calcd for $C_{29}H_{19}F_4O_4^-$: 507.1225; found: 507.1228.

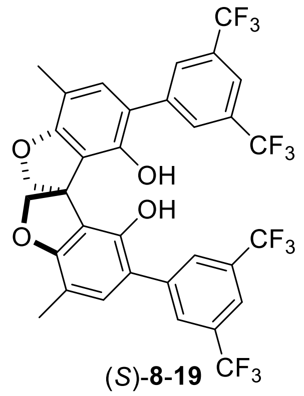

(*S*)-**8-19**

Light yellow solid, 1.13 g, 80% yield.

^{1}H NMR (500 MHz, $CDCl_3$) δ 7.93 (s, 4H), 7.80 (s, 2H), 7.05 (s, 2H), 5.27 (s, 2H), 4.96 (d, *J* = 9.5 Hz, 2H), 4.66 (d, *J* = 9.5 Hz, 2H), 2.26 (s, 6H).

^{13}C NMR (125 MHz, $CDCl_3$) δ 158.60, 147.58, 139.36, 133.48, 132.09, 131.82, 131.56, 131.30, 129.34, 126.60, 124.43, 122.26, 120.57, 120.34, 120.09, 114.29, 114.15, 79.03, 54.78, 14.32.

^{19}F NMR (471 MHz, $CDCl_3$) δ -62.79.

HRMS (ESI): m/z $[M-H]^-$ calcd for $C_{33}H_{19}F_{12}O_4^-$: 707.1097; found: 707.1093.

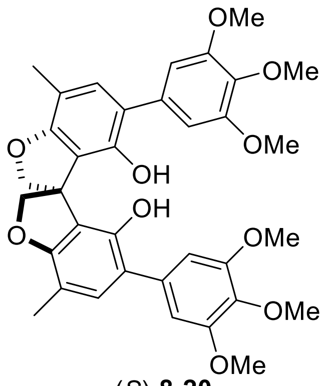

(*S*)-**8-20**

Light yellow solid, 0.86 g, 70% yield.

^{1}H NMR (500 MHz, $CDCl_3$) δ 6.95 (s, 2H), 6.58 (s, 4H), 5.33 (s, 2H), 5.01 (d, *J* = 9.2 Hz, 2H), 4.64 (d, *J* = 9.2 Hz, 2H), 3.85 (s, 18H), 2.22 (s, 6H).

^{13}C NMR (125 MHz, $CDCl_3$) δ 158.76, 153.70, 147.57, 137.31, 132.40, 132.26, 121.81, 114.05, 112.30, 106.20, 81.05, 60.92, 56.24, 55.19, 14.39.

HRMS (ESI): m/z $[M-H]^-$ calcd for $C_{35}H_{35}O_{10}^-$: 615.2236; found: 615.2228.

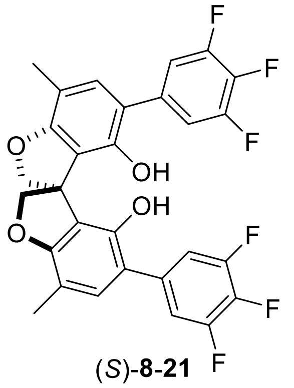

(*S*)-**8-21**

Yellow solid, 0.72 g, 66% yield.

^{1}H NMR (500 MHz, $CDCl_3$) δ 7.08 (t, *J* = 7.4 Hz, 4H), 6.94 (s, 2H), 5.19 (s, 2H), 4.92 (d, *J* = 9.3 Hz, 2H), 4.62 (d, *J* = 9.3 Hz, 2H), 2.22 (s, 6H).

^{13}C NMR (125 MHz, $CDCl_3$) δ 157.54, 151.06, 149.11, 146.38, 138.81, 136.81, 131.89, 118.81, 113.16, 112.51, 112.29, 112.25, 112.16, 112.12, 78.57, 53.81, 13.28.

^{19}F NMR (471 MHz, $CDCl_3$) δ -134.18 (d, *J* = 20.6 Hz), -162.45 (t, *J* = 20.7 Hz).

HRMS (ESI): m/z $[M-H]^-$ calcd for $C_{29}H_{17}F_6O_4^-$: 543.1037; found: 543.1040.

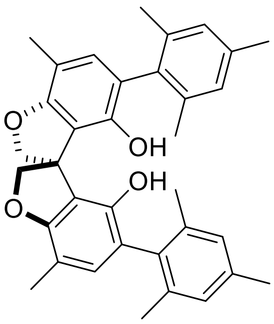

(*S*)-**8-22**

Yellow solid, 0.60 g, 58% yield.

^{1}H NMR (500 MHz, $CDCl_3$) δ 6.94 (s, 2H), 6.90 (s, 2H), 6.63 (s, 2H), 4.91 (d, *J* = 9.0 Hz, 2H), 4.68 (d, *J* = 9.1 Hz, 2H), 4.48 (s, 2H), 2.29 (s, 6H), 2.17 (s, 6H), 2.04 (s, 6H), 1.87 (s, 6H).

^{13}C NMR (125 MHz, $CDCl_3$) δ 157.45, 146.52, 137.02, 136.96, 136.39, 130.71, 130.55, 127.48, 127.38, 118.60, 112.78, 110.68, 80.37, 54.21, 19.98, 19.37, 18.95, 13.41.

HRMS (ESI): m/z $[M-H]^-$ calcd for $C_{35}H_{35}O_4^-$: 519.2541; found: 519.2535.

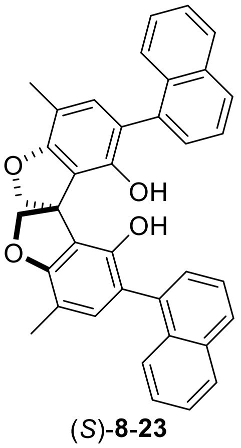

(*S*)-**8-23**

Yellow solid, 0.69 g, 64% yield.

^{1}H NMR (500 MHz, $CDCl_3$) δ 7.93 – 7.64 (m, 6H), 7.55 – 7.33 (m, 8H), 6.95 – 6.93 (m, 2H), 5.06 – 4.73 (m, 6H), 2.21 (s, 6H).

^{13}C NMR (125 MHz, $CDCl_3$) δ 159.30, 159.23, 159.07, 148.40, 148.37, 148.35, 148.27, 134.13, 134.12, 134.05, 134.03, 134.01, 133.92, 133.89, 133.38, 133.31, 133.19, 133.08, 132.28, 132.23, 132.14, 128.53, 128.48, 128.46, 128.44, 128.40, 128.38, 128.36, 128.25, 126.82, 126.60, 126.55, 126.38, 126.22, 126.13, 126.06, 125.92, 125.86, 125.83, 125.72, 125.69, 125.65, 119.77, 119.67, 119.59, 119.58, 114.01, 113.89, 113.82, 113.80, 111.99, 111.97, 81.80, 81.52, 81.50, 55.37, 14.43.

HRMS (ESI): m/z [M-H]$^-$ calcd for $C_{37}H_{27}O_4^-$: 535.1915; found: 535.1915.

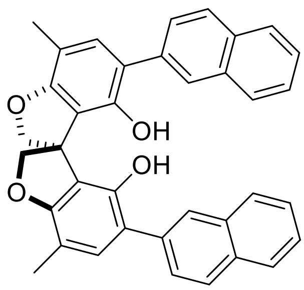

(*S*)-**8**-**24**

Yellow solid, 0.63 g, 59% yield.

^{1}H NMR (500 MHz, $CDCl_3$) δ 7.90 – 7.88 (m, 4H), 7.85 – 7.81 (m, 4H), 7.56 (dd, *J* = 8.5, 2.3 Hz, 2H), 7.49 – 7.47 (m, 4H), 7.08 (s, 2H), 5.40 (s, 2H), 5.02 (d, *J* = 9.2 Hz, 2H), 4.69 (d, *J* = 9.2 Hz, 2H), 2.25 (s, 6H).

^{13}C NMR (125 MHz, $CDCl_3$) δ 158.87, 147.85, 134.40, 133.60, 132.89, 132.39, 128.78, 127.88, 127.70, 127.52, 127.47, 126.45, 126.07, 121.82, 114.05, 112.64, 81.10, 55.25, 14.44.

HRMS (ESI): m/z [M-H]$^-$ calcd for $C_{37}H_{27}O_4^-$: 535.1915; found: 535.1917.

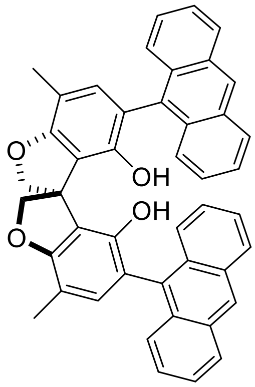

(*S*)-**8**-**25**

Yellow solid, 0.76 g, 60% yield.

^{1}H NMR (500 MHz, $CDCl_3$) δ 8.53 (s, 2H), 8.06 (t, *J* = 9.3 Hz, 4H), 7.84 (d, *J* = 8.6 Hz, 2H), 7.53 – 7.41 (m, 8H), 7.00 – 6.97 (m, 2H), 6.89 (s, 2H), 5.10 (d, *J* = 9.3 Hz, 2H), 4.86 (d, *J* = 9.3 Hz, 2H), 4.53 (s, 2H), 2.20 (s, 6H).

^{13}C NMR (125 MHz, $CDCl_3$) δ 159.46, 149.04, 133.84, 131.61, 131.53, 131.33, 131.23, 130.01, 128.61, 128.47, 127.60, 126.38, 126.14, 126.11, 125.41, 125.30, 117.05, 114.25, 112.12, 82.05, 55.44, 14.45.

HRMS (ESI): m/z [M-H]$^-$ calcd for $C_{45}H_{31}O_4^-$: 635.2228; found: 635.2231.

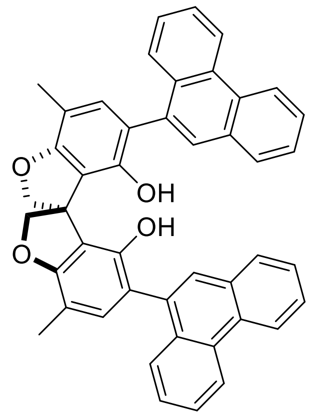

(*S*)-**8-26**

Yellow solid, 0.76 g, 60% yield.

^{1}H NMR (500 MHz, $CDCl_3$) δ 8.79 – 8.71 (m, 4H), 7.91 – 7.57 (m, 14H), 7.01 – 6.99 (m, 2H), 5.09 – 4.76 (m, 6H), 2.22 (s, 6H).

^{13}C NMR (125 MHz, $CDCl_3$) δ 158.28, 155.68, 147.56, 147.45, 138.11, 132.36, 132.27, 132.15, 132.05, 131.65, 130.48, 130.16, 129.83, 129.68, 129.25, 128.33, 128.28, 128.08, 127.63, 126.25, 125.97, 125.93, 125.82, 125.73, 125.45, 121.99, 121.89, 121.61, 121.56, 120.61, 118.71, 118.58, 113.01, 112.79, 111.08, 80.79, 80.47, 54.36, 28.67, 13.39.

HRMS (ESI): m/z $[M-H]^-$ calcd for $C_{45}H_{31}O_4^-$: 635.2228; found: 635.2234.

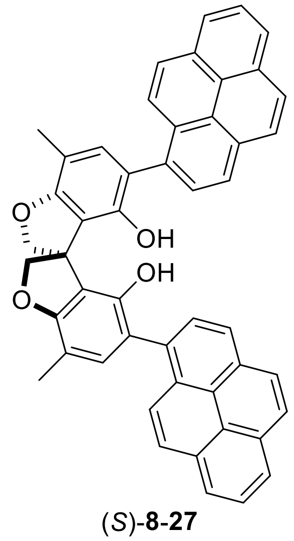

(*S*)-**8-27**

Yellow solid, 1.16 g, 85% yield.

^{1}H NMR (500 MHz, $CDCl_3$) δ 8.29 – 7.79 (m, 18H), 7.08 – 7.04 (m, 2H), 5.14 – 4.81 (m, 6H), 2.25 (s, 6H).

^{13}C NMR (125 MHz, $CDCl_3$) δ 159.58, 159.39, 148.57, 148.51, 148.39, 133.85,

133.72, 133.54, 131.40, 131.36, 131.26, 131.18, 131.02, 130.84, 129.72, 128.71, 128.48, 128.17, 128.08, 127.94, 127.80, 127.70, 127.40, 127.34, 126.24, 126.16, 125.40, 125.26, 125.17, 125.08, 125.05, 124.96, 124.82, 124.72, 120.37, 120.26, 120.22, 113.79, 113.50, 112.32, 81.51, 55.49, 14.49.
HRMS (ESI): m/z $[M-H]^-$ calcd for $C_{49}H_{31}O_4^-$: 683.2228; found: 683.2221.

Synthesis of (*S*)-9

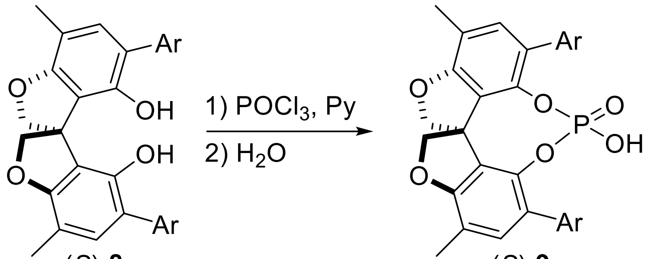

The solution of (*S*)-**8** (1.0 mmol) in pyridine (10 mL) was stirred at 0 °C for 10 min, then $POCl_3$ (6.0 mmol, 0.92 g) was added. The reaction mixture was stirred at 110 °C for 12 h and cooled to 0 °C, then 10 mL H_2O was slowly added. The mixture was stirred at 0 °C for 10 min and at 110 °C for 7 h. After cooling to room temperature, 1 M HCl (20 mL) was added. The mixture was extracted with dichloromethane (20 mL × 3). The combined organic layers were washed with H_2O (20 mL × 3) and dried over anhydrous sodium sulfate. Solvent was evaporated under reduced pressure and the residue was purified by column chromatography on silica gel (PE/EA = 1/1, V/V) to afford the product (*S*)-**9**.

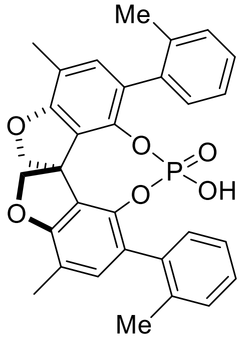

(*S*)-**9**-**1**

Light yellow solid, 0.13 g, 25% yield.

^{1}H NMR (500 MHz, DMSO-*d*$_6$) δ 7.18 – 7.07 (m, 8H), 6.75 (s, 2H), 4.67 (d, *J* = 8.2 Hz, 2H), 4.11 (d, *J* = 7.7 Hz, 2H), 2.15 (s, 6H), 2.10 (s, 6H).

^{13}C NMR (125 MHz, DMSO-*d*$_6$) δ 158.83, 145.36, 145.30, 139.28, 136.72, 133.32, 131.89, 129.77, 129.75, 129.44, 126.77, 125.22, 120.00, 114.29, 80.11, 55.06, 27.63, 14.52.

^{31}P NMR (202 MHz, DMSO-*d*$_6$) δ -12.38.

HRMS (ESI): m/z $[M-H]^-$ calcd for $C_{31}H_{26}O_6P^-$: 525.1472; found: 525.1474.

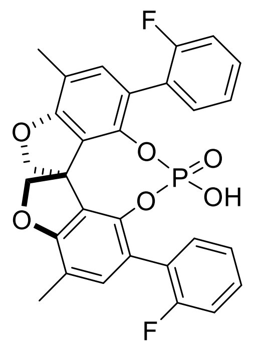

(S)-**9-2**

Pink solid, 0.45 g, 85% yield.

^{1}H NMR (500 MHz, DMSO-d_6) δ 7.54 – 7.50 (m, 2H), 7.27 – 7.22 (m, 2H), 7.11 – 7.07 (m, 4H), 6.93 (s, 2H), 4.68 (d, J = 7.9 Hz, 2H), 4.11 (d, J = 7.9 Hz, 2H), 2.17 (s, 6H).

^{13}C NMR (125 MHz, DMSO-d_6) δ 160.79, 159.56, 158.84, 145.87, 133.52, 128.74, 126.88, 123.90, 123.68, 120.05, 115.18, 114.51, 80.15, 60.23, 55.02, 21.23, 14.50.

^{19}F NMR (471 MHz, DMSO-d_6) δ -114.86.

^{31}P NMR (202 MHz, DMSO-d_6) δ -12.65.

HRMS (ESI): m/z [M-H]$^-$ calcd for $C_{29}H_{20}F_2O_6P^-$: 533.0971; found: 533.0971.

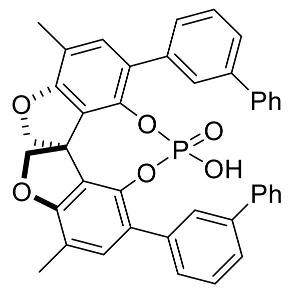

(S)-**9-3**

Light yellow solid, 0.58 g, 89% yield.

^{1}H NMR (500 MHz, DMSO-d_6) δ 8.09 (s, 2H), 7.71 (d, J = 7.6 Hz, 4H), 7.58 (d, J = 7.7 Hz, 2H), 7.51 (d, J = 7.6 Hz, 2H), 7.45 – 7.39 (m, 6H), 7.33 (t, J = 7.4 Hz, 2H), 7.17 (s, 2H), 4.65 (d, J = 8.1 Hz, 2H), 4.15 (d, J = 8.0 Hz, 2H), 2.21 (s, 6H).

^{13}C NMR (125 MHz, DMSO-d_6) δ 159.24, 145.29, 145.22, 141.15, 139.95, 139.71, 133.02, 129.25, 128.86, 128.77, 128.61, 127.62, 127.36, 124.80, 120.29, 114.97, 79.91, 55.18, 14.58.

^{31}P NMR (202 MHz, DMSO-d_6) δ -12.39.

HRMS (ESI): m/z [M-H]$^-$ calcd for $C_{41}H_{30}O_6P^-$: 649.1785; found: 649.1786.

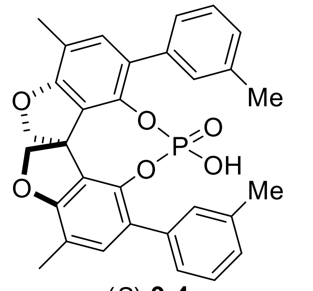

(*S*)-**9-4**

Light yellow solid, 0.44 g, 84% yield.

^{1}H NMR (500 MHz, DMSO-*d*$_6$) δ 7.46 – 7.43 (m, 4H), 7.17 (t, *J* = 7.6 Hz, 2H), 7.02 – 7.01 (m, 4H), 4.62 (d, *J* = 8.0 Hz, 2H), 4.10 (d, *J* = 7.9 Hz, 2H), 2.29 (s, 6H), 2.18 (s, 6H).

^{13}C NMR (125 MHz, DMSO-*d*$_6$) δ 158.94, 145.10, 139.15, 136.78, 132.97, 130.62, 129.59, 127.94, 127.12, 127.05, 120.22, 114.72, 79.89, 55.14, 21.64, 14.55.

^{31}P NMR (202 MHz, DMSO-*d*$_6$) δ -12.73.

HRMS (ESI): m/z [M-H]$^-$ calcd for $C_{31}H_{26}O_6P^-$: 525.1472; found: 525.1472.

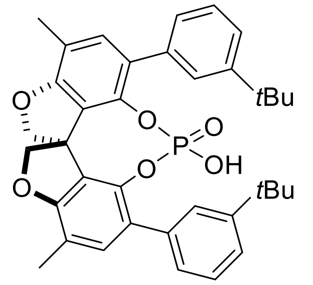

(*S*)-**9-5**

Light yellow solid, 0.35 g, 57% yield.

^{1}H NMR (500 MHz, DMSO-*d*$_6$) δ 7.77 (s, 2H), 7.53 (d, *J* = 8.5 Hz, 2H), 7.22 – 7.19 (m, 4H), 7.04 (s, 2H), 4.59 (d, *J* = 8.0 Hz, 2H), 4.08 (d, *J* = 7.9 Hz, 2H), 2.19 (s, 6H), 1.28 (s, 18H).

^{13}C NMR (125 MHz, DMSO-*d*$_6$) δ 158.89, 150.09, 145.20, 138.71, 133.00, 129.69, 127.67, 127.16, 127.09, 123.10, 120.09, 114.66, 79.73, 55.20, 34.88, 31.80, 14.58.

^{31}P NMR (202 MHz, DMSO-*d*$_6$) δ -12.27.

HRMS (ESI): m/z [M-H]$^-$ calcd for $C_{37}H_{38}O_6P^-$: 609.2411; found: 609.2415.

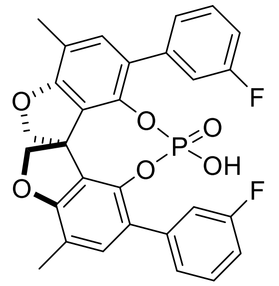

(*S*)-**9**-**6**

Light yellow solid, 0.38 g, 71% yield.

^{1}H NMR (500 MHz, DMSO-d_6) δ 7.69 (d, *J* = 11.1 Hz, 2H), 7.38 – 7.30 (m, 4H), 7.09 (s, 2H), 7.03 (t, *J* = 8.0 Hz, 2H), 4.633 (d, *J* = 8.1 Hz, 2H), 4.11 (d, *J* = 8.0 Hz, 2H), 2.18 (s, 6H).

^{13}C NMR (125 MHz, DMSO-d_6) δ 172.48, 163.18, 161.27, 159.50, 145.14, 145.07, 141.52, 141.45, 133.01, 129.76, 129.69, 128.01, 125.93, 125.91, 120.18, 120.16, 116.84, 116.66, 115.11, 113.21, 113.04, 79.90, 55.04, 14.52.

^{19}F NMR (471 MHz, DMSO-d_6) δ -114.45.

^{31}P NMR (202 MHz, DMSO-d_6) δ -12.23.

HRMS (ESI): m/z [M-H]$^-$ calcd for $C_{29}H_{20}F_2O_6P^-$: 533.0971; found: 533.0974.

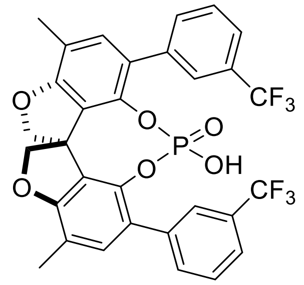

(*S*)-**9**-**7**

Light yellow solid, 0.43 g, 68% yield.

^{1}H NMR (500 MHz, DMSO-d_6) δ 7.99 (s, 2H), 7.87 (d, *J* = 7.1 Hz, 2H), 7.56 – 7.50 (m, 4H), 7.12 (s, 2H), 4.66 (d, *J* = 8.1 Hz, 2H), 4.15 (d, *J* = 8.0 Hz, 2H), 2.20 (s, 6H).

^{13}C NMR (125 MHz, DMSO-d_6) δ 172.46, 159.76, 145.46, 145.40, 140.15, 133.73, 132.85, 129.00, 128.75, 127.91, 126.32, 126.04, 123.87, 122.99, 122.96, 120.38, 115.19, 114.48, 80.04, 55.01, 14.50.

^{19}F NMR (471 MHz, DMSO-d_6) δ -60.77.

^{31}P NMR (202 MHz, DMSO-d_6) δ -12.23.

HRMS (ESI): m/z [M-H]$^-$ calcd for $C_{31}H_{20}F_6O_6P^-$: 633.0907; found: 633.0909.

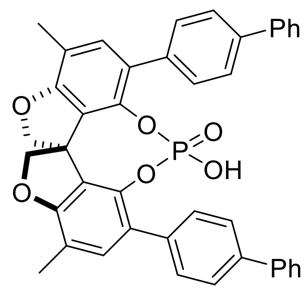

(*S*)-**9-8**

Light yellow solid, 0.55 g, 85% yield.

^{1}H NMR (500 MHz, DMSO-*d*$_6$) δ 7.76 (d, *J* = 7.9 Hz, 4H), 7.70 (d, *J* = 7.6 Hz, 4H), 7.60 (d, *J* = 7.9 Hz, 4H), 7.45 (t, *J* = 7.5 Hz, 4H), 7.34 (t, *J* = 7.2 Hz, 2H), 7.11 (s, 2H), 4.65 (d, *J* = 8.0 Hz, 2H), 4.14 (d, *J* = 7.9 Hz, 2H), 2.21 (s, 6H).

^{13}C NMR (125 MHz, DMSO-*d*$_6$) δ 159.21, 145.17, 140.55, 138.43, 137.99, 132.86, 130.40, 129.37, 128.91, 127.62, 126.91, 126.33, 120.33, 114.99, 79.93, 55.16, 14.61.

^{31}P NMR (202 MHz, DMSO-*d*$_6$) δ -12.37.

HRMS (ESI): m/z [M-H]$^-$ calcd for $C_{41}H_{30}O_6P^-$: 649.1785; found: 649.1784.

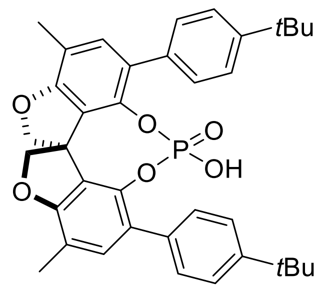

(*S*)-**9-9**

White solid, 0.31 g, 51% yield.

^{1}H NMR (500 MHz, DMSO-*d*$_6$) δ 7.62 (d, *J* = 8.5 Hz, 4H), 7.31 (d, *J* = 8.5 Hz, 4H), 7.01 (s, 2H), 4.59 (d, *J* = 7.9 Hz, 2H), 4.07 (d, *J* = 7.8 Hz, 2H), 2.17 (s, 6H), 1.29 (s, 18H).

^{13}C NMR (125 MHz, DMSO-*d*$_6$) δ 158.83, 148.34, 145.28, 136.37, 132.94, 129.59, 129.19, 124.77, 120.12, 114.58, 79.74, 55.16, 34.55, 31.69, 14.59.

^{31}P NMR (202 MHz, DMSO-*d*$_6$) δ -12.02.

HRMS (ESI): m/z [M-H]$^-$ calcd for $C_{37}H_{38}O_6P^-$: 609.2411; found: 609.2413.

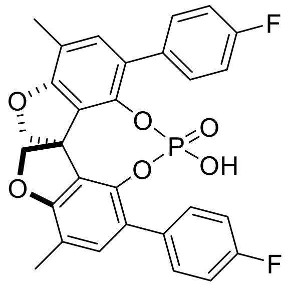

(*S*)-**9-10**

White solid, 0.45 g, 84% yield.

^{1}H NMR (500 MHz, DMSO-d_6) δ 7.70 – 7.67 (m, 4H), 7.10 (t, *J* = 9.0 Hz, 4H), 7.02 (s, 2H), 4.62 (d, *J* = 7.9 Hz, 2H), 4.09 (d, *J* = 7.9 Hz, 2H), 2.17 (s, 6H).

^{13}C NMR (125 MHz, DMSO-d_6) δ 162.36, 160.43, 159.12, 145.08, 145.01, 135.50, 135.48, 132.93, 131.88, 131.82, 128.39, 128.36, 120.13, 120.11, 114.87, 114.77, 114.61, 79.84, 55.11, 14.52.

^{19}F NMR (471 MHz, DMSO-d_6) δ -117.39.

^{31}P NMR (202 MHz, DMSO-d_6) δ -12.42.

HRMS (ESI): m/z [M-H]$^-$ calcd for $C_{29}H_{20}F_2O_6P^-$: 533.0971; found: 533.0969.

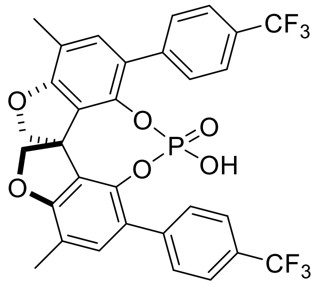

(*S*)-**9-11**

Light yellow solid, 0.30 g, 47% yield.

^{1}H NMR (500 MHz, DMSO-d_6) δ 7.81 (d, *J* = 8.1 Hz, 4H), 7.63 (d, *J* = 8.3 Hz, 4H), 7.12 (s, 2H), 4.67 (d, *J* = 8.1 Hz, 2H), 4.15 (d, *J* = 8.0 Hz, 2H), 2.20 (s, 6H).

^{13}C NMR (125 MHz, DMSO-d_6) δ 159.91, 145.36, 143.38, 132.95, 130.43, 128.34, 128.02, 128.00, 127.10, 126.87, 126.62, 126.36, 126.18, 124.87, 124.84, 124.81, 124.78, 124.02, 120.41, 115.33, 80.07, 55.01, 14.52.

^{19}F NMR (471 MHz, DMSO-d_6) δ -60.64.

^{31}P NMR (202 MHz, DMSO-d_6) δ -12.79.

HRMS (ESI): m/z [M-H]$^-$ calcd for $C_{31}H_{20}F_6O_6P^-$: 633.0907; found: 633.0910.

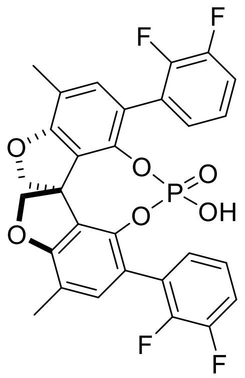

(*S*)-**9-12**

Light green solid, 0.13 g, 23% yield.

^{1}H NMR (500 MHz, DMSO-d_6) δ 7.27 – 7.22 (m, 4H), 7.11 – 7.07 (m, 2H), 6.99 (s, 2H), 4.72 (d, *J* = 8.1 Hz, 2H), 4.13 (d, *J* = 8.1 Hz, 2H), 2.18 (s, 6H).

^{13}C NMR (125 MHz, DMSO-d_6) δ 160.04, 151.27, 151.13, 149.30, 149.15, 148.68, 148.61, 146.76, 146.69, 146.01, 133.22, 129.23, 129.14, 128.45, 123.86, 122.62, 120.23, 120.21, 115.67, 115.53, 114.86, 80.28, 54.94, 14.48.

^{19}F NMR (471 MHz, DMSO-d_6) δ -140.38 (d, *J* = 22.6 Hz), -140.67 (d, *J* = 22.5 Hz).

^{31}P NMR (202 MHz, DMSO-d_6) δ -12.88.

HRMS (ESI): m/z [M-H]$^-$ calcd for $C_{29}H_{18}F_4O_6P^-$: 569.0783; found: 569.0784.

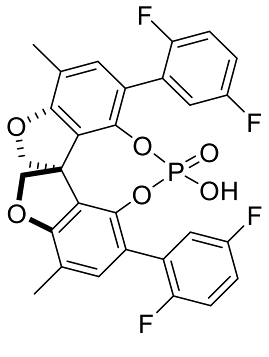

(*S*)-**9-13**

Light yellow solid, 0.43 g, 75% yield.

^{1}H NMR (500 MHz, DMSO-d_6) δ 7.53 – 7.49 (m, 2H), 7.18 – 7.07 (m, 4H), 6.99 (s, 2H), 4.70 (d, *J* = 8.1 Hz, 2H), 4.12 (d, *J* = 8.0 Hz, 2H), 2.17 (s, 6H).

^{13}C NMR (125 MHz, DMSO-d_6) δ 159.91, 158.83, 157.01, 156.96, 155.10, 145.75, 145.69, 133.43, 128.32, 128.24, 128.17, 128.10, 122.40, 120.05, 120.02, 119.77, 119.58, 116.64, 116.57, 116.44, 116.36, 115.18, 115.11, 114.99, 114.92, 114.80, 80.18, 54.95, 14.47.

^{19}F NMR (471 MHz, DMSO-d_6) δ -120.33 (d, *J* = 17.3 Hz), -120.72 (d, *J* = 17.5

Hz).

^{31}P NMR (202 MHz, DMSO-d_6) δ -12.61.

HRMS (ESI): m/z [M-H]$^-$ calcd for $C_{29}H_{18}F_4O_6P^-$: 569.0783; found: 569.0784.

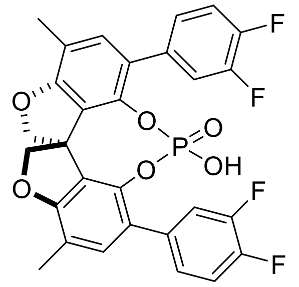

(*S*)-**9-14**

Light pink solid, 0.44 g, 77% yield.

^{1}H NMR (500 MHz, DMSO-d_6) δ 7.97 – 7.93 (m, 2H), 7.37 – 7.31 (m, 2H), 7.08 (s, 2H), 4.63 (d, *J* = 8.1 Hz, 2H), 4.10 (d, *J* = 8.0 Hz, 2H), 2.18 (s, 6H).

^{13}C NMR (125 MHz, DMSO-d_6) δ 159.55, 150.14, 150.04, 149.66, 149.56, 148.21, 148.11, 147.72, 147.62, 144.92, 136.51, 132.99, 127.19, 126.60, 120.08, 119.05, 118.90, 116.94, 116.81, 115.27, 79.87, 55.03, 14.47.

^{19}F NMR (471 MHz, DMSO-d_6) δ -139.99 (d, *J* = 22.9 Hz), -142.69 (d, *J* = 22.6 Hz).

^{31}P NMR (202 MHz, DMSO-d_6) δ -12.34.

HRMS (ESI): m/z [M-H]$^-$ calcd for $C_{29}H_{18}F_4O_6P^-$: 569.0783; found: 569.0783.

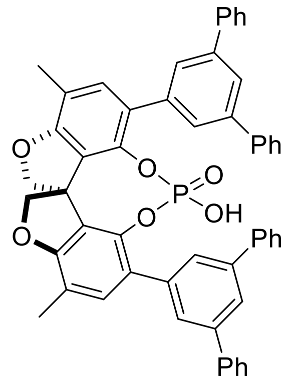

(*S*)-**9-15**

White solid, 0.51 g, 64% yield.

^{1}H NMR (500 MHz, DMSO-d_6) δ 7.99 (d, *J* = 1.6 Hz, 4H), 7.81 – 7.79 (m, 8H), 7.74 (t, *J* = 1.6 Hz, 2H), 7.46 (t, *J* = 7.7 Hz, 8H), 7.36 (d, *J* = 7.4 Hz, 4H), 7.32 (s, 2H), 4.67 (d, *J* = 7.9 Hz, 2H), 4.19 (d, *J* = 7.8 Hz, 2H), 2.24 (s, 6H).

^{13}C NMR (125 MHz, DMSO-d_6) δ 158.95, 145.08, 145.03, 140.68, 140.31, 139.83, 132.53, 128.79, 128.48, 127.30, 127.11, 122.87, 119.91, 114.53, 79.48, 54.73, 14.11.

^{31}P NMR (202 MHz, DMSO-d_6) δ -12.33.

HRMS (ESI): m/z [M-H]$^-$ calcd for $C_{53}H_{38}O_6P^-$: 801.2411; found: 801.2415.

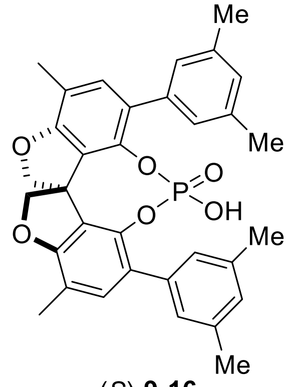

(*S*)-**9-16**

White solid, 0.32 g, 58% yield.

^{1}H NMR (500 MHz, DMSO-d_6) δ 7.22 (s, 4H), 6.99 (s, 2H), 6.83 (s, 2H), 4.61 (d, *J* = 7.8 Hz, 2H), 4.11 (d, *J* = 7.8 Hz, 2H), 2.24 (s, 12H), 2.17 (s, 6H).

^{13}C NMR (125 MHz, DMSO-d_6) δ 158.81, 145.23, 139.18, 136.52, 132.80, 129.78, 127.84, 127.79, 120.33, 114.45, 79.92, 55.11, 21.55, 14.53.

^{31}P NMR (202 MHz, DMSO-d_6) δ -12.62.

HRMS (ESI): m/z [M-H]$^-$ calcd for $C_{33}H_{30}O_6P^-$: 553.1785; found: 553.1779.

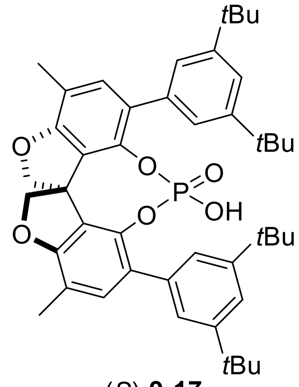

(*S*)-**9-17**

White solid, 0.34 g, 47% yield.

^{1}H NMR (500 MHz, DMSO-d_6) δ 7.57 (d, *J* = 1.6 Hz, 4H), 7.22 (s, 2H), 7.06 (s, 2H), 4.58 (d, *J* = 7.9 Hz, 2H), 4.07 (d, *J* = 7.8 Hz, 2H), 2.19 (s, 6H), 1.29 (s,

36H).

^{13}C NMR (125 MHz, DMSO-d_6) δ 158.75, 149.42, 145.51, 138.20, 132.81, 130.04, 124.15, 120.25, 119.73, 114.39, 79.68, 55.22, 34.99, 31.94, 14.60.

^{31}P NMR (202 MHz, DMSO-d_6) δ -12.21.

HRMS (ESI): m/z [M-H]$^-$ calcd for $C_{45}H_{54}O_6P^-$: 721.3663; found: 721.3661.

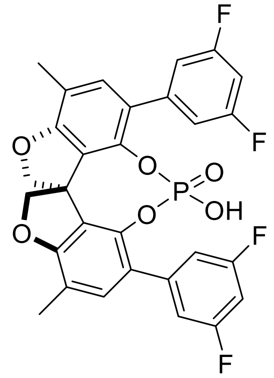

(*S*)-**9-18**

White solid, 0.51 g, 89% yield.

^1H NMR (500 MHz, DMSO-d_6) δ 7.43 (d, *J* = 7.5 Hz, 4H), 7.16 (s, 2H), 7.05 (t, *J* = 9.3 Hz, 2H), 4.65 (d, *J* = 8.0 Hz, 2H), 4.12 (d, *J* = 8.0 Hz, 2H), 2.19 (s, 6H).

^{13}C NMR (125 MHz, DMSO-d_6) δ 163.31, 163.20, 161.38, 161.27, 159.93, 145.27, 142.99, 142.64, 132.97, 126.90, 120.24, 115.30, 113.01, 112.80, 101.93, 101.72, 101.51, 79.95, 54.96, 14.48.

^{19}F NMR (471 MHz, DMSO-d_6) δ -111.60.

^{31}P NMR (202 MHz, DMSO-d_6) δ -12.10.

HRMS (ESI): m/z [M-H]$^-$ calcd for $C_{29}H_{18}F_4O_6P^-$: 569.0783; found: 569.0782.

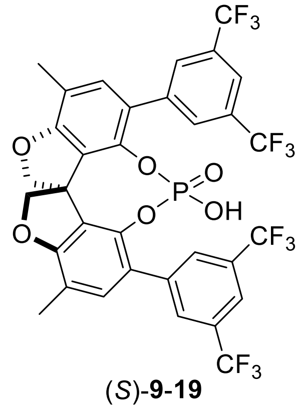

(*S*)-**9-19**

White solid, 0.64 g, 83% yield.

^1H NMR (500 MHz, DMSO-d_6) δ 8.21 (s, 4H), 7.89 (s, 2H), 7.28 (s, 2H), 4.71

(d, J = 7.9 Hz, 2H), 4.21 (d, J = 7.6 Hz, 2H), 2.22 (s, 6H).

^{13}C NMR (125 MHz, DMSO-d_6) δ 160.57, 145.66, 145.62, 141.67, 139.99, 132.87, 130.20, 130.11, 129.94, 126.38, 125.11, 122.94, 120.56, 119.78, 115.74, 80.22, 54.90, 14.43.

^{19}F NMR (471 MHz, DMSO-d_6) δ -61.14.

^{31}P NMR (202 MHz, DMSO-d_6) δ -12.55.

HRMS (ESI): m/z $[M-H]^-$ calcd for $C_{33}H_{18}F_{12}O_6P^-$: 769.0655; found: 769.0658.

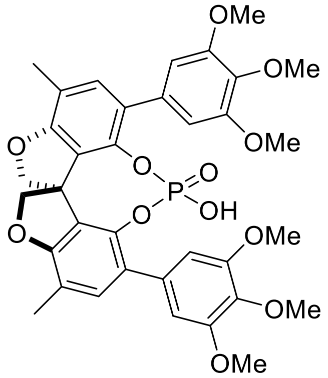

(*S*)-**9**-**20**

White solid, 0.46 g, 68% yield.

^{1}H NMR (500 MHz, DMSO-d_6) δ 7.14 (s, 4H), 7.10 (s, 2H), 4.57 (d, J = 8.0 Hz, 2H), 4.05 (d, J = 7.9 Hz, 2H), 3.76 (s, 12H), 3.67 (s, 6H), 2.18 (s, 6H).

^{13}C NMR (125 MHz, DMSO-d_6) δ 158.90, 152.52, 145.02, 136.38, 134.47, 132.90, 129.08, 119.87, 114.71, 107.76, 79.61, 60.36, 56.33, 55.19, 14.57.

^{31}P NMR (202 MHz, DMSO-d_6) δ -11.81.

HRMS (ESI): m/z $[M-H]^-$ calcd for $C_{35}H_{34}O_{12}P^-$: 677.1793; found: 677.1788.

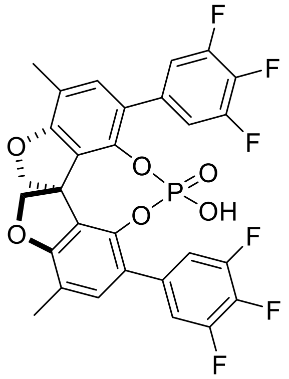

(*S*)-**9**-**21**

Light yellow solid, 0.42 g, 69% yield.

^{1}H NMR (500 MHz, DMSO-d_6) δ 7.70 – 7.67 (m, 4H), 7.15 (s, 2H), 4.64 (d, J =

8.1 Hz, 2H), 4.10 (d, *J* = 8.1 Hz, 2H), 2.18 (s, 6H).

^{13}C NMR (125 MHz, DMSO-d_6) δ 159.98, 150.97, 149.14, 149.06, 145.06, 144.99, 133.05, 126.11, 120.12, 120.10, 115.48, 114.50, 114.46, 114.36, 114.33, 79.92, 54.95, 14.47.

^{19}F NMR (471 MHz, DMSO-d_6) δ -137.18 (d, *J* = 21.9 Hz), -165.76 (t, *J* = 22.0 Hz).

^{31}P NMR (202 MHz, DMSO-d_6) δ -11.79.

HRMS (ESI): m/z [M-H]$^-$ calcd for $C_{29}H_{16}F_6O_6P^-$: 605.0594; found: 605.0597.

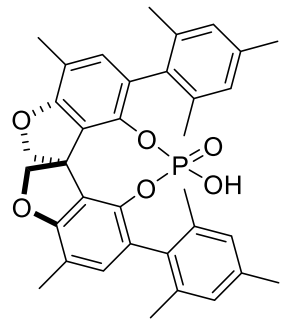

(*S*)-**9-22**

Light yellow solid, 0.35 g, 60% yield.

^{1}H NMR (500 MHz, DMSO-d_6) δ 6.77 (s, 2H), 6.73 (s, 2H), 6.63 (s, 2H), 4.62 (d, *J* = 8.0 Hz, 2H), 4.04 (d, *J* = 7.9 Hz, 2H), 2.20 (s, 6H), 2.16 (s, 6H), 2.13 (s, 6H), 1.89 (s, 6H).

^{13}C NMR (125 MHz, DMSO-d_6) δ 158.45, 146.23, 137.97, 136.61, 135.99, 135.16, 133.05, 128.55, 128.05, 127.29, 119.80, 114.00, 79.75, 55.06, 21.73, 21.12, 20.54, 14.55.

^{31}P NMR (202 MHz, DMSO-d_6) δ -11.29.

HRMS (ESI): m/z [M-H]$^-$ calcd for $C_{35}H_{34}O_6P^-$: 581.2098; found: 581.2100.

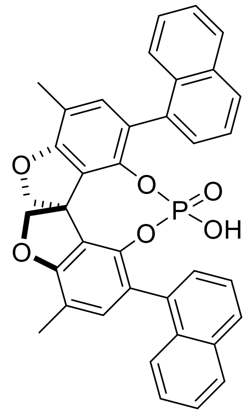

(*S*)-**9-23**

Light yellow solid, 0.44 g, 74% yield.

^{1}H NMR (500 MHz, DMSO-d_6) δ 7.88 – 7.74 (m, 4H), 7.61 – 7.27 (m, 10H), 6.92– 6.85 (m, 2H), 4.82 – 4.76 (m, 2H), 4.32 – 4.29 (m, 2H), 2.21 – 2.19 (m, 6H).

^{13}C NMR (125 MHz, DMSO-d_6) δ 159.33, 145.94, 145.88, 137.24, 134.14, 133.13, 132.39, 129.47, 128.28, 128.09, 127.16, 126.54, 125.98, 125.84, 125.49, 125.22, 119.87, 114.56, 80.32, 55.19, 55.11, 14.52.

^{31}P NMR (202 MHz, DMSO) δ -12.68, -12.85.

HRMS (ESI): m/z [M-H]$^-$ calcd for $C_{37}H_{26}O_6P^-$: 597.1472; found: 597.1473.

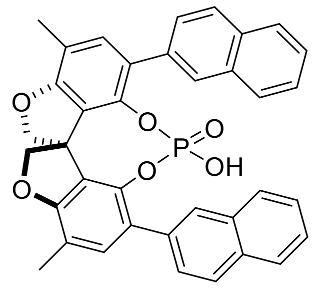

(*S*)-**9-24**

Light yellow solid, 0.39 g, 65% yield.

^{1}H NMR (500 MHz, DMSO-d_6) δ 8.11 (s, 2H), 7.86 – 7.78 (m, 8H), 7.45 – 7.43 (m, 4H), 7.19 (s, 2H), 4.69 (d, *J* = 8.1 Hz, 2H), 4.19 (d, *J* = 8.0 Hz, 2H), 2.23 (s, 6H).

^{13}C NMR (125 MHz, DMSO-d_6) δ 159.30, 145.41, 136.89, 133.48, 133.16, 132.07, 129.37, 128.92, 128.40, 128.04, 127.70, 127.07, 126.05, 125.80, 120.46, 114.98, 80.05, 55.18, 14.61.

^{31}P NMR (202 MHz, DMSO-d_6) δ -12.44.

HRMS (ESI): m/z [M-H]$^-$ calcd for $C_{37}H_{26}O_6P^-$: 597.1472; found: 597.1474.

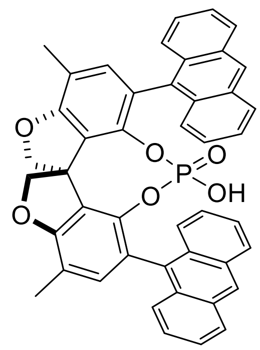

(*S*)-**9-25**

Light yellow solid, 0.42 g, 60% yield.

^{1}H NMR (500 MHz, DMSO-d_6) δ 8.42 (s, 2H), 8.01 (d, *J* = 8.4 Hz, 2H), 7.91 (d, *J* = 8.4 Hz, 2H), 7.83 (d, *J* = 8.9 Hz, 2H), 7.63 (d, *J* = 8.7 Hz, 2H), 7.43 – 7.30 (m, 6H), 7.14 – 7.11 (m, 2H), 6.84 (s, 2H), 4.93 (d, *J* = 8.1 Hz, 2H), 4.47 (d, *J* = 8.1 Hz, 2H), 2.23 (s, 6H).

^{13}C NMR (125 MHz, DMSO-d_6) δ 159.57, 135.25, 134.67, 131.81, 131.17, 131.06, 130.11, 129.82, 128.60, 127.57, 127.46, 126.11, 125.73, 125.55, 125.21, 125.06, 124.02, 120.22, 114.48, 80.44, 55.23, 14.61.

^{31}P NMR (202 MHz, DMSO-d_6) δ -11.84.

HRMS (ESI): m/z $[M-H]^-$ calcd for $C_{45}H_{30}O_6P^-$: 697.1785; found: 697.1779.

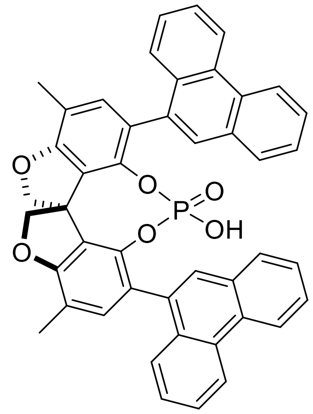

(*S*)-**9-26**

White solid, 0.34 g, 49% yield.

^{1}H NMR (500 MHz, DMSO-d_6) δ 8.82 – 8.72 (m, 4H), 7.96 – 7.79 (m, 4H), 7.63 – 7.37 (m, 10H), 7.03 – 6.99 (m, 2H), 4.87 – 4.82 (m, 2H), 4.40 – 4.34 (m, 2H), 2.24 – 2.21 (m, 6H).

^{13}C NMR (125 MHz, DMSO-d_6) δ 159.54, 135.41, 134.08, 131.91, 131.82, 129.95, 129.81, 129.16, 128.01, 127.99, 127.16, 126.90, 126.76, 126.34,

124.53, 123.23, 123.00, 119.92, 114.80, 80.43, 55.27, 55.17, 14.64, 14.55.
^{31}P NMR (202 MHz, DMSO) δ -12.94, -13.09.
HRMS (ESI): m/z [M-H]$^-$ calcd for $C_{45}H_{30}O_6P^-$: 697.1785; found: 697.1784.

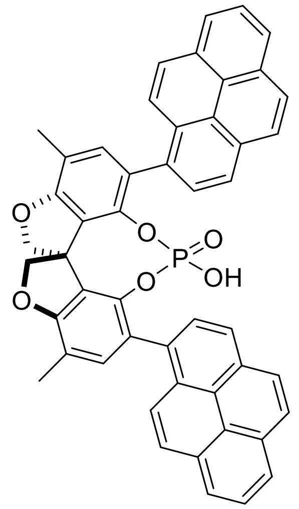

(*S*)-**9-27**

White solid, 0.56 g, 75% yield.
^{1}H NMR (500 MHz, DMSO-*d*$_6$) δ 8.24 – 7.82 (m, 18H), 7.06 – 7.03 (m, 2H), 4.95 – 4.88 (m, 2H), 4.51 – 4.42 (m, 2H), 2.28 (d, J = 7.3 Hz, 6H).
^{13}C NMR (125 MHz, DMSO-*d*$_6$) δ 159.65, 145.95, 135.39, 135.34, 134.63, 131.28, 131.25, 131.01, 130.87, 130.47, 130.07, 130.01, 129.78, 129.37, 128.87, 128.78, 128.59, 127.85, 127.74, 127.21, 126.52, 126.40, 125.28, 125.07, 124.94, 124.85, 124.49, 124.42, 123.89, 120.10, 120.08, 115.09, 80.56, 55.30, 55.26, 14.63.
^{31}P NMR (202 MHz, DMSO) δ -12.66, -12.68.
HRMS (ESI): m/z [M-H]$^-$ calcd for $C_{49}H_{30}O_6P^-$: 745.1785; found: 745.1785.

3. Synthesis of QDI 10

10 was synthesized according to the reference (*Chin. J. Chem.* **2024**, *42,* 731.).

4. Synthesis of indoles 11

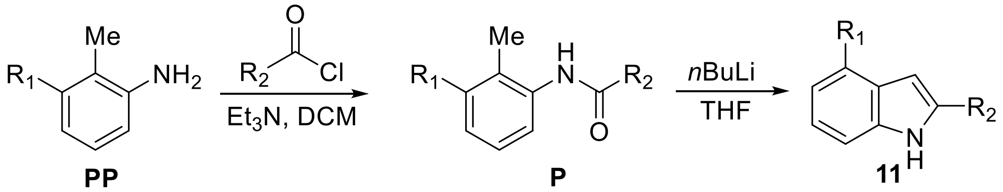

To a solution of **PP** (20 mmol) and Et_3N (25 mmol) in CH_2Cl_2 (DCM, 20 mL) was added a solution of acyl chloride (21 mol) in DCM (10 mL) at 0 °C. After addition, the mixture was stirred overnight at room temperature. The mixture was washed with 2 N HCl (20 mL × 4) and brine. The organic layer was dried over Na_2SO_4 and concentrated under vacuum to give product **P** which was used without further purification.

To a solution of **P** in dry THF (40 mL) as added dropwise *n*BuLi (2.4 M in hexane, 48 mmol) at 0 °C. After addition, the mixture was stirred overnight at 25 °C. The mixture was cooled in an ice-water bath and treated with saturated NH_4Cl. The organic layer was separated and the aqueous layer was extracted with ethyl acetate. The combined organic layers were dried over Na_2SO_4, filtered, and concentrated in vacuum. The residue was purified by column chromatography on silica gel (DCM/PE = 1/2) to give product **11**.

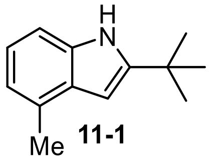

11-1

Light yellow solid, 3.37 g, 90% yield.

^{1}H NMR (400 MHz, $CDCl_3$) δ 7.97 (s, 1H), 7.22 (d, *J* = 8.0 Hz, 1H), 7.10 (t, *J* = 8.0 Hz, 1H), 6.95 (d, *J* = 8.0 Hz, 1H), 6.33 – 6.32 (m, 1H), 2.60 (s, 3H), 1.45 (s, 9H).

^{13}C NMR (100 MHz, $CDCl_3$) δ 148.24, 135.50, 129.59, 128.33, 121.32, 119.87, 108.09, 95.49, 31.94, 30.46, 18.88.

HRMS (ESI) m/z $[M+H]^+$ calcd for $C_{13}H_{18}N^+$ 188.1434; found: 188.1433.

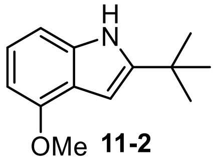

11-2

White solid, 3.58 g, 88% yield.

^{1}H NMR (400 MHz, $CDCl_3$) δ 8.03 (s, 1H), 7.13 – 7.10 (m, 1H), 7.02 (d, *J* = 8.0 Hz, 1H), 6.58 (d, *J* = 8.0 Hz, 1H), 6.43 (s, 1H), 4.00 (s, 3H), 1.42 (s, 9H).

^{13}C NMR (100 MHz, $CDCl_3$) δ 152.88, 147.47, 137.21, 121.89, 118.86, 104.14, 99.65, 94.04, 55.40, 31.87, 30.39.

HRMS (ESI) m/z $[M+H]^+$ calcd for $C_{13}H_{18}NO^+$ 204.1383; found: 204.1383.

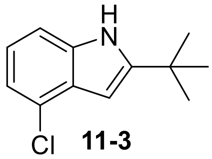

11-3

White solid, 3.82 g, 92% yield.

^{1}H NMR (400 MHz, $CDCl_3$) δ 8.07 (s, 1H), 7.24 (d, *J* = 8.0 Hz, 1H), 7.13 – 7.04 (m, 2H), 6.41-6.40 (m, 1H), 1.43 (s, 9H).

^{13}C NMR (100 MHz, $CDCl_3$) δ 149.68, 136.51, 127.34, 125.28, 121.77, 119.41, 109.16, 95.78, 32.03, 30.31.

HRMS (ESI) m/z $[M+H]^+$ calcd for $C_{12}H_{15}ClN^+$ 208.0888; found: 208.0889.

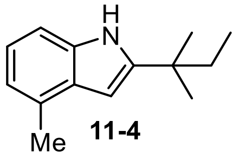

11-4

Yellowish-brown solid, 3.54 g, 88% yield.

^{1}H NMR (400 MHz, $CDCl_3$) δ 7.94 (s, 1H), 7.20 (d, *J* = 8.0 Hz, 1H), 7.07 (t, *J* = 8.0 Hz, 1H), 6.93 (d, *J* = 8.0 Hz, 1H), 6.29 – 6.28 (m, 1H), 2.58 (s, 3H), 1.72 (q, *J* = 8.0 Hz, 2H), 1.39 (s, 6H), 0.82 (t, *J* = 8.0 Hz, 3H).

^{13}C NMR (100 MHz, $CDCl_3$) δ 146.73, 135.55, 129.51, 128.38, 121.20, 119.79, 108.05, 96.93, 36.08, 35.43, 27.70, 18.90, 9.32.

HRMS (ESI) m/z $[M+H]^+$ calcd for $C_{14}H_{20}N^+$ 202.1590; found: 202.1590.

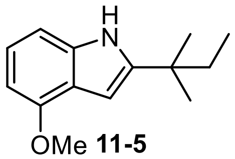

OMe **11-5**

Brown solid, 3.74 g, 86% yield.

^{1}H NMR (400 MHz, $CDCl_3$) δ 7.95 (s, 1H), 7.08 (t, *J* = 7.9 Hz, 1H), 6.98 (d, *J* = 8.1 Hz, 1H), 6.54 (d, *J* = 7.7 Hz, 1H), 6.40 – 6.38 (m, 1H), 3.98 (s, 3H), 1.67 (q, *J* = 7.4 Hz, 2H), 1.36 (s, 6H), 0.80 (t, *J* = 7.5 Hz, 3H).

^{13}C NMR (100 MHz, $CDCl_3$) δ 152.83, 145.91, 137.28, 121.82, 118.94, 104.10, 99.60, 95.52, 55.40, 36.08, 35.41, 27.67, 9.29.

HRMS (ESI) m/z $[M+H]^+$ calcd for $C_{14}H_{20}NO^+$ 218.1539; found: 218.1540.

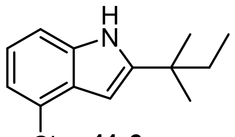

Cl **11-6**

White solid, 3.99 g, 90% yield.

^{1}H NMR (400 MHz, $CDCl_3$) δ 8.11 (s, 1H), 7.32-7.26 (m, 2H), 7.18 (t, *J* = 8.0 Hz, 1H), 6.55 – 6.54 (m, 1H), 1.75 (q, *J* = 8.0 Hz, 2H), 1.44 (s, 6H), 0.89 (t, *J* = 8.0 Hz, 3H).

^{13}C NMR (100 MHz, $CDCl_3$) δ 148.29, 136.56, 127.29, 124.98, 121.56, 119.21, 109.23, 96.97, 35.77, 35.36, 27.35, 9.15.

HRMS (ESI) m/z $[M+H]^+$ calcd for $C_{13}H_{17}ClN^+$ 222.1044; found: 222.1046.

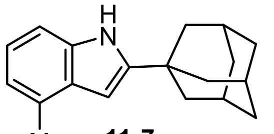

Me **11-7**

Light yellow solid, 4.51 g, 85% yield.

^{1}H NMR (400 MHz, $CDCl_3$) δ 8.01 (s, 1H), 7.19 (d, *J* = 8.0 Hz, 1H), 7.05 (t, *J* = 8.0 Hz, 1H), 6.90 (d, *J* = 8.0 Hz, 1H), 6.27 – 6.25 (m, 1H), 2.55 (s, 3H), 2.14-2.13 (m, 3H), 2.03 – 2.01 (m, 6H), 1.87 – 1.79 (m, 6H).

^{13}C NMR (100 MHz, $CDCl_3$) δ 148.74, 135.17, 129.61, 128.31, 121.22, 119.79, 108.12, 94.91, 42.79, 36.91, 33.85, 28.63, 18.89.

HRMS (ESI) m/z $[M+H]^+$ calcd for $C_{19}H_{24}N^+$ 266.1903; found: 266.1902.

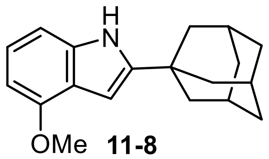

OMe **11-8**

Light yellow solid, 4.84 g, 86% yield.

^{1}H NMR (400 MHz, $CDCl_3$) δ 8.02 (s, 1H), 7.07 (t, *J* = 7.9 Hz, 1H), 6.98 (d, *J* = 8.1 Hz, 1H), 6.52 (d, *J* = 7.7 Hz, 1H), 6.36 (s, 1H), 3.97 (s, 3H), 2.13 – 2.10 (m, 3H), 2.03 – 2.00 (m, 6H), 1.83 – 1.80 (m, 6H).

^{13}C NMR (100 MHz, $CDCl_3$) δ 153.02, 147.95, 136.91, 121.82, 118.94, 104.16, 99.64, 93.53, 55.45, 42.76, 36.90, 33.81, 28.62.

HRMS (ESI) m/z $[M+H]^+$ calcd for $C_{19}H_{24}NO^+$ 282.1852; found: 282.1853.

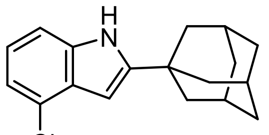

Cl **11-9**

White solid, 4.86 g, 85% yield.

^{1}H NMR (400 MHz, $CDCl_3$) δ 8.11 (s, 1H), 7.23 – 7.21 (m, 1H), 7.09 – 7.02 (m, 2H), 6.35 – 6.34 (m, 1H), 2.13 – 2.12 (m, 3H), 2.01 – 2.00 (m, 6H), 1.85 – 1.77 (m, 6H).

^{13}C NMR (100 MHz, $CDCl_3$) δ 150.06, 136.21, 127.36, 125.40, 121.66, 119.35, 109.15, 95.26, 42.62, 36.81, 33.94, 28.53.

HRMS (ESI) m/z $[M+H]^+$ calcd for $C_{18}H_{21}ClN^+$ 286.1357; found: 286.1358.

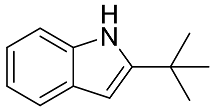

11-10

Compound **11-10** was synthesized following a literature procedure (*Angew. Chem. Int. Ed.* **2022**, *61*, e202205159).

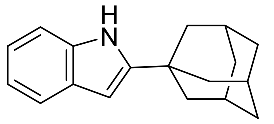

11-11

Compound **11-11** was synthesized according to a literature method (*Angew. Chem. Int. Ed.* **2022**, *61*, e202205159).

5. Screening of conditions for the electrophilic amination of indoles

Structure and numbering of chiral phosphoric acids

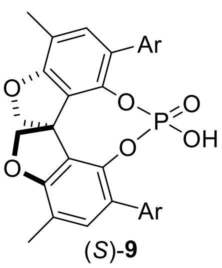

(*S*)-**9**

(*S*)-**9-1**, Ar = 2-methylphenyl
(*S*)-**9-2**, Ar = 2-fluorophenyl
(*S*)-**9-3**, Ar = 3-phenylphenyl
(*S*)-**9-4**, Ar = 3-methylphenyl
(*S*)-**9-5**, Ar = 3-*tert*-butylphenyl
(*S*)-**9-6**, Ar = 3-fluorophenyl
(*S*)-**9-7**, Ar = 3-trifluoromethylphenyl
(*S*)-**9-8**, Ar = 4-phenylphenyl
(*S*)-**9-11**, Ar = 4-trifluoromethylphenyl
(*S*)-**9-12**, Ar = 2, 3-difluorophenyl
(*S*)-**9-13**, Ar = 2, 5-difluorophenyl
(*S*)-**9-14**, Ar = 3, 4-difluorophenyl
(*S*)-**9-15**, Ar = 3, 5-diphenylphenyl
(*S*)-**9-17**, Ar = 3, 5-di-*tert*-butylphenyl
(*S*)-**9-18**, Ar = 3, 5-difluorophenyl
(*S*)-**9-19**, Ar = 3, 5-ditrifluoromethylphenyl
(*S*)-**9-20**, Ar = 3, 4, 5-trimethoxylphenyl
(*S*)-**9-21**, Ar = 3, 4, 5-trifluorophenyl
(*S*)-**9-22**, Ar = 2, 4, 6-trimethylphenyl
(*S*)-**9-23**, Ar = 1-naphthyl
(*S*)-**9-24**, Ar = 2-naphthyl
(*S*)-**9-26**, Ar = 9-phenanthryl
(*S*)-**9-27**, Ar = 1-pyrenyl

Screening of chiral phosphoric acids[a]

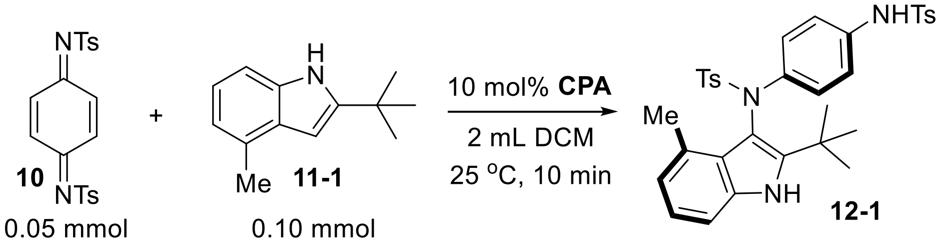

Entry	CPA	Ee/%[b]	Yield/%[c]
01	(*S*)-**9-1**	79%	94
02	(*S*)-**9-2**	75%	92
03	(*S*)-**9-3**	79%	95
04	(*S*)-**9-4**	70%	90
05	(*S*)-**9-5**	55%	92
06	(*S*)-**9-6**	71%	95
07	(*S*)-**9-7**	65%	93
08	(*S*)-**9-8**	79%	94
09	(*S*)-**9-11**	64%	92
10	(*S*)-**9-12**	74%	90
11	(*S*)-**9-13**	73%	95
12	(*S*)-**9-14**	65%	94
13	(*S*)-**9-15**	75%	95
14	(*S*)-**9-17**	31%	93
15	(*S*)-**9-18**	70%	93
16	(*S*)-**9-19**	45%	94

17	(*S*)-**9-20**	53%	91
18	(*S*)-**9-21**	63%	95
19	(*S*)-**9-22**	84%	96
20	(*S*)-**9-23**	79%	92
21	(*S*)-**9-24**	67%	94
22	(*S*)-**9-26**	81%	95
23	(*S*)-**9-27**	77%	93

[a] Reactions were performed with **10** (0.05 mmol), **11-1** (0.10 mmol), and **CPA** in DCM (2.0 mL) at 25 °C for 10 min. [b] Determined by HPLC analysis on a chiral stationary phase. [c] Isolated yield.

Screening of solvents and other conditions[a]

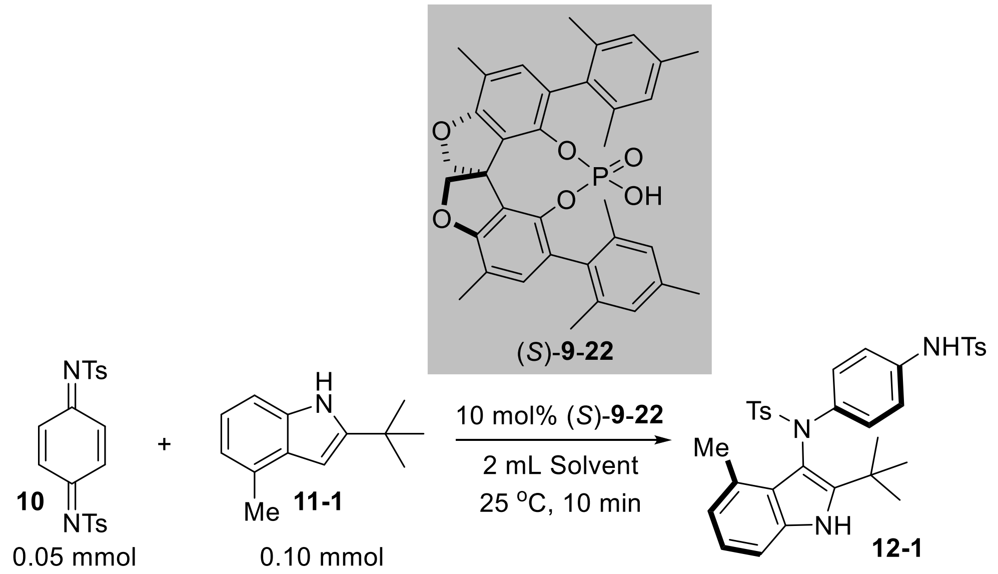

Entry	Solvent	Ee/%[b]	Yield/%[c]
1	DCM	84	96
2	DCE	83	96
3	$CHCl_3$	79	95
4	Toluene	77	92
5[d]	DCM	84	97
6[e]	DCM	97	96
7[f]	DCM	97	98

[a] Reactions were performed with **10** (0.05 mmol), **11-1** (0.10 mmol), and (*S*)-**9-22** (10 mol%) in solvent (2.0 mL) at 25 °C for 10 min. [b] Determined by HPLC analysis on a chiral stationary phase. [c] Isolated yield. [d] 1 mL DCM was used. [e] 1 mL DCM was used. The

reaction was conducted at -20 ^{o}C for 4 h. [f] (*S*)-**9-22** (0.2 mol%) and 1 mL DCM was used. The reaction was conducted at -20 ^{o}C for 12 h.

6. General procedure for asymmetric synthesis of axially chiral 3-aryl-aminoindoles 12

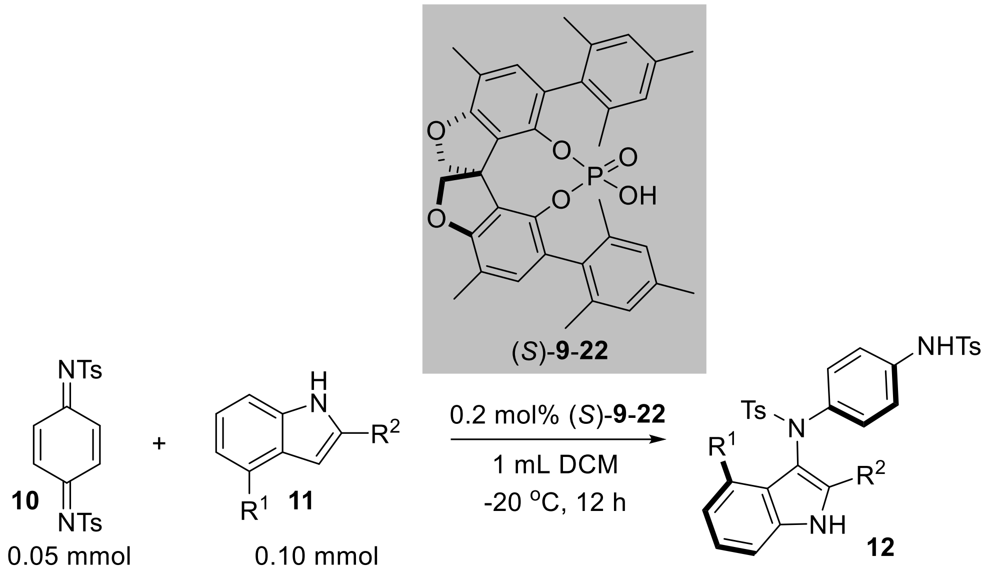

Into an oven dried Schlenk tube chiral phosphoric acid (*S*)-**9-22** (0.2 mol%, 60 μg/mL in DCM, 1 mL) and **11** (0.10 mmol) were added. The reaction mixture was stirred at -20 °C for 10 min, then **10** (0.05 mmol) was added. The reaction was stirred at -20 °C for 12 h. TLC analysis indicated that the starting material **10** was completely consumed. Solvent was removed under reduced pressure and the crude residue was purified by column chromatography on silica gel (EA/DCM = 1/30) to give pure product **12**.

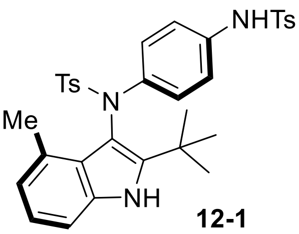

12-1

HPLC conditions: Cel-Z (Guangzhou Starchrom Technologies Co., Ltd); hexane = 0.65 mL/min, isopropanol = 0.35 mL/min; T = 40 °C; λ = 254 nm; t_R (major) = 10.2 min, t_R (minor) = 13.3 min.

HPLC chromatogram of racemic **12-1**

<Chromatogram>

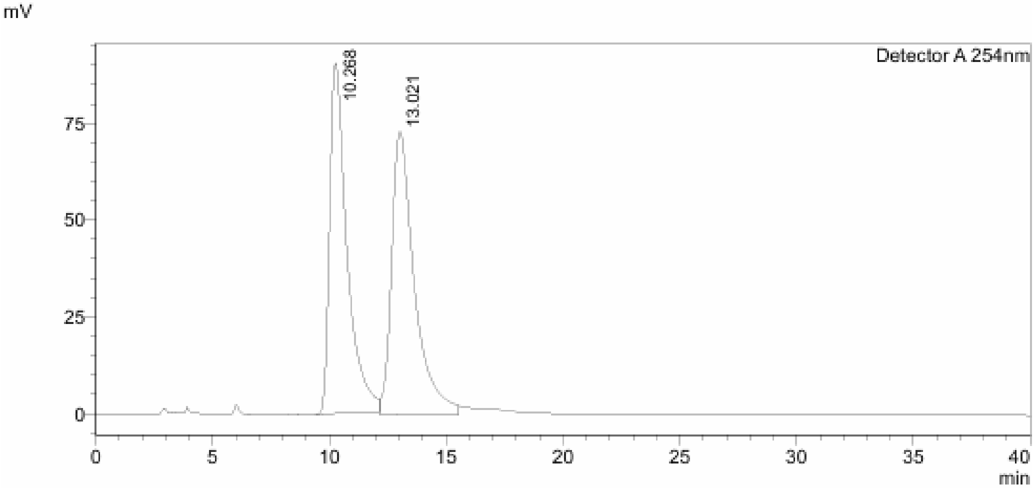

<Peak Table>

Detector A 254nm

Peak#	Ret. Time	Area	Height	Conc.	Unit	Mark	Name
1	10.268	4660158	90454	48.534			
2	13.021	4941674	72863	51.466		M	
Total		9601833	163317				

HPLC chromatogram of **12-1**

<Chromatogram>

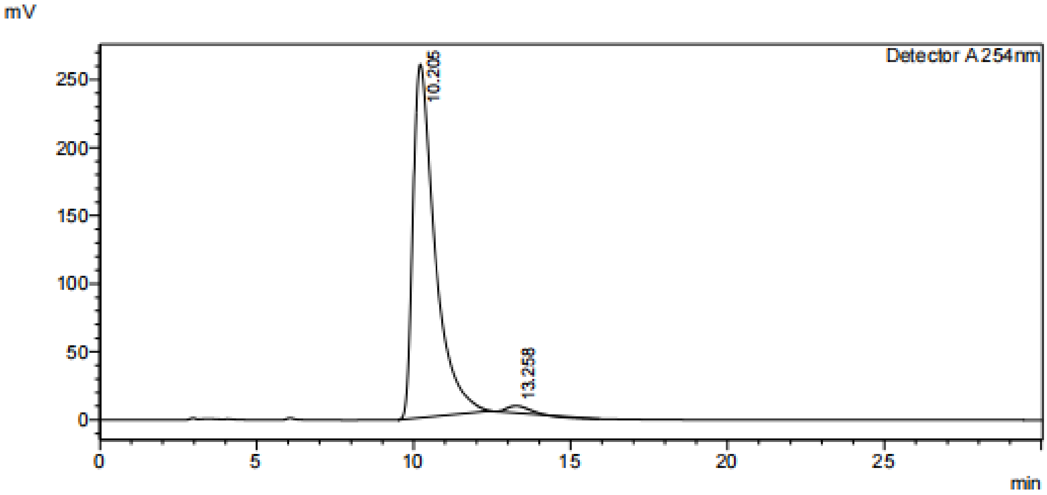

<Peak Table>

Detector A 254nm

Peak#	Ret. Time	Area	Height	Conc.	Unit	Mark	Name
1	10.205	12623501	259780	98.325		M	
2	13.258	215039	5199	1.675		M	
Total		12838540	264979				

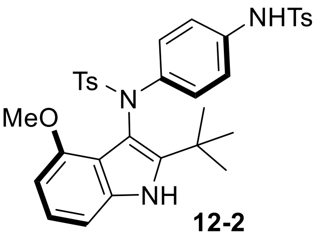

12-2

HPLC conditions: Cel-D (Guangzhou Starchrom Technologies Co., Ltd); hexane = 0.80 mL/min, isopropanol = 0.20 mL/min; T = 40 ^{o}C; λ = 254 nm; t_R (major) = 19.8 min, t_R (minor) = 25.8 min.

HPLC chromatogram of racemic **12-2**

<Chromatogram>

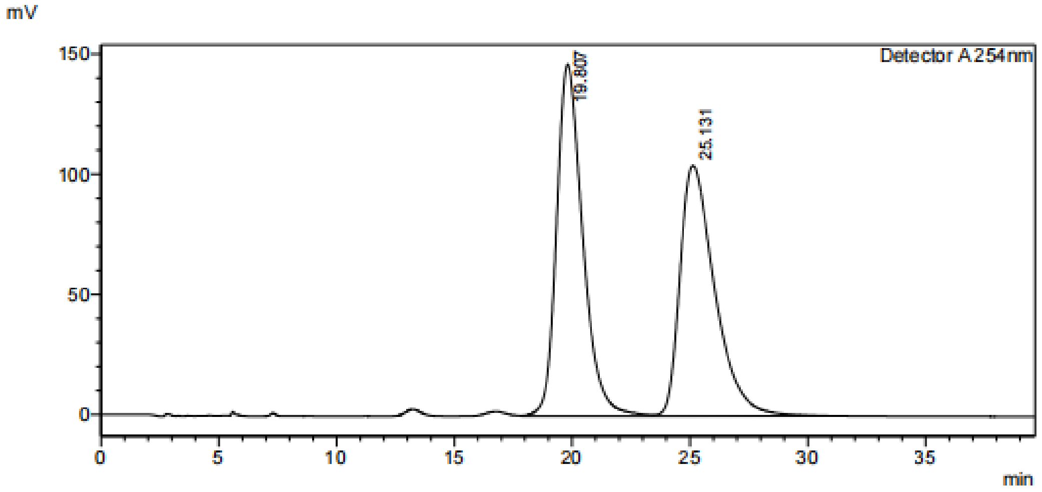

<Peak Table>

Detector A 254nm

Peak#	Ret. Time	Area	Height	Conc.	Unit	Mark	Name
1	19.807	11343832	146395	51.388			
2	25.131	10730891	104213	48.612		V	
Total		22074722	250608				

HPLC chromatogram of **12-2**

<Chromatogram>

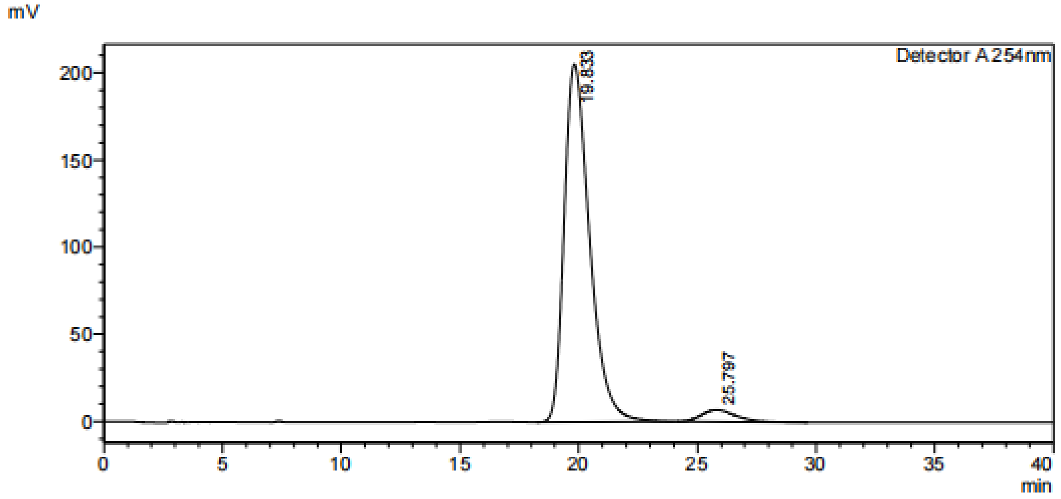

<Peak Table>

Detector A 254nm

Peak#	Ret. Time	Area	Height	Conc.	Unit	Mark	Name
1	19.833	15228666	205185	95.669			
2	25.797	689462	6796	4.331			
Total		15918128	211981				

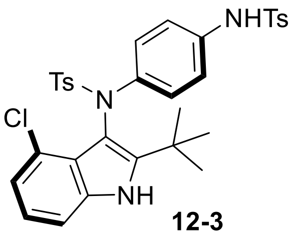

12-3

HPLC conditions: Cel-X (Guangzhou Starchrom Technologies Co., Ltd); hexane = 0.70 mL/min, isopropanol = 0.30 mL/min; T = 40 °C; λ = 254 nm; t_R (major) = 25.0 min, t_R (minor) = 39.5 min.

HPLC chromatogram of racemic **12-3**

<Chromatogram>

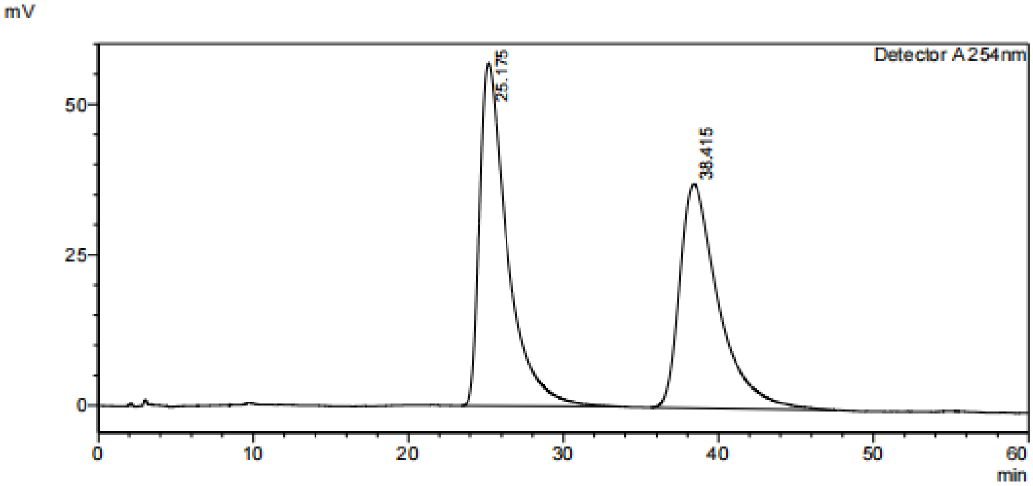

<Peak Table>

Detector A 254nm

Peak#	Ret. Time	Area	Height	Conc.	Unit	Mark	Name
1	25.175	7040817	56870	52.101			
2	38.415	6472872	37160	47.899			
Total		13513689	94030				

HPLC chromatogram of **12-3**

<Chromatogram>

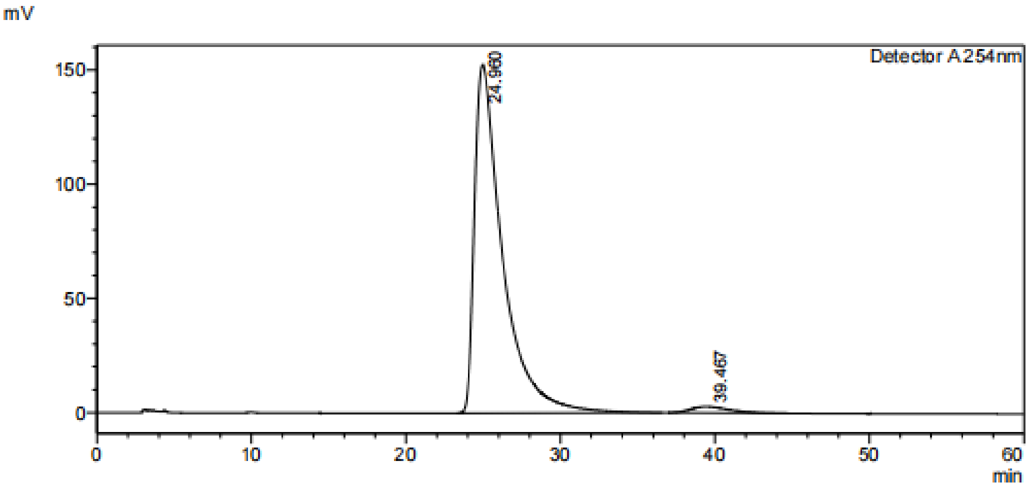

<Peak Table>

Detector A 254nm

Peak#	Ret. Time	Area	Height	Conc.	Unit	Mark	Name
1	24.960	19234949	152477	97.652		S	
2	39.467	462442	2675	2.348			
Total		19697390	155153				

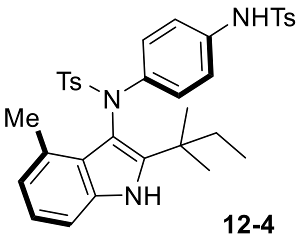

12-4

HPLC conditions: Cel-X (Guangzhou Starchrom Technologies Co., Ltd); hexane = 0.70 mL/min, isopropanol = 0.30 mL/min; T = 40 °C; λ = 254 nm; t_R (major) = 21.2 min, t_R (minor) = 30.0 min.

HPLC chromatogram of racemic **12-4**

<Chromatogram>

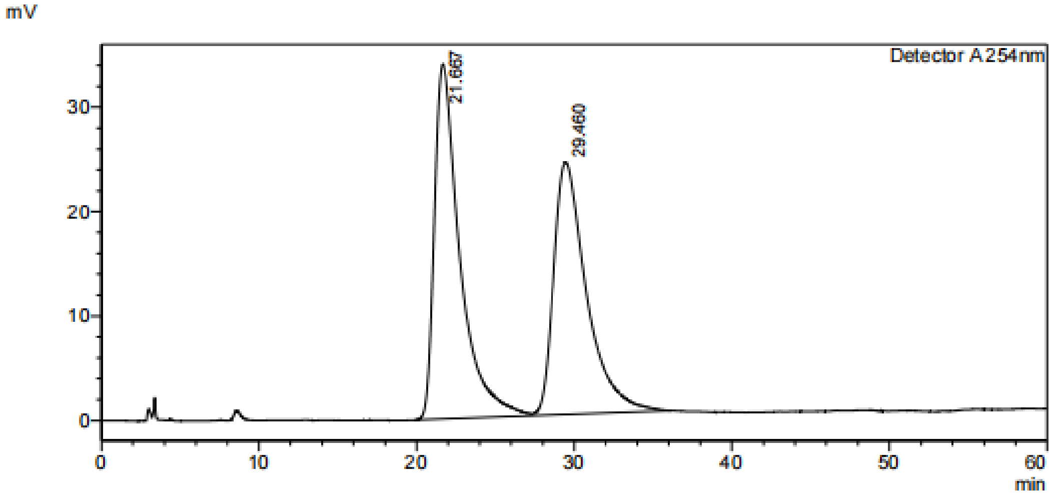

<Peak Table>

Detector A 254nm

Peak#	Ret. Time	Area	Height	Conc.	Unit	Mark	Name
1	21.667	3836498	33943	52.341			
2	29.460	3493276	24151	47.659		V	
Total		7329775	58094				

HPLC chromatogram of **12-4**

<Chromatogram>

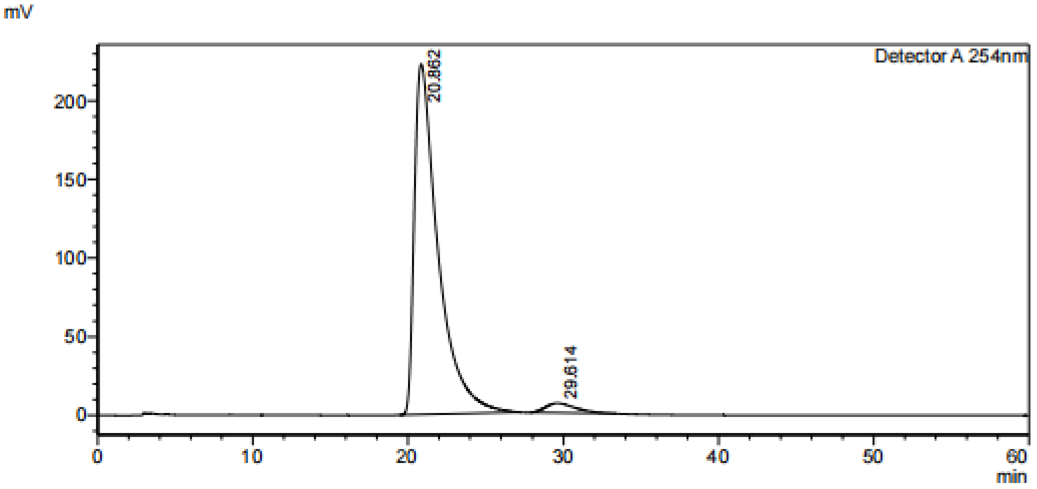

<Peak Table>

Detector A 254nm

Peak#	Ret. Time	Area	Height	Conc.	Unit	Mark	Name
1	20.862	23714056	222858	96.593		M	
2	29.614	836474	6154	3.407		M	
Total		24550530	229012				

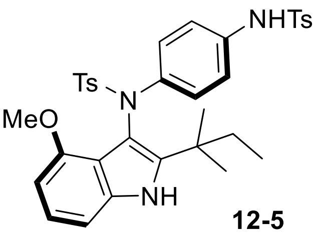

12-5

HPLC conditions: Cel-D (Guangzhou Starchrom Technologies Co., Ltd); hexane = 0.80 mL/min, isopropanol = 0.20 mL/min; T = 40 °C; λ = 254 nm; t_R (major) = 18.3 min, t_R (minor) = 23.0 min.

HPLC chromatogram of racemic **12-5**

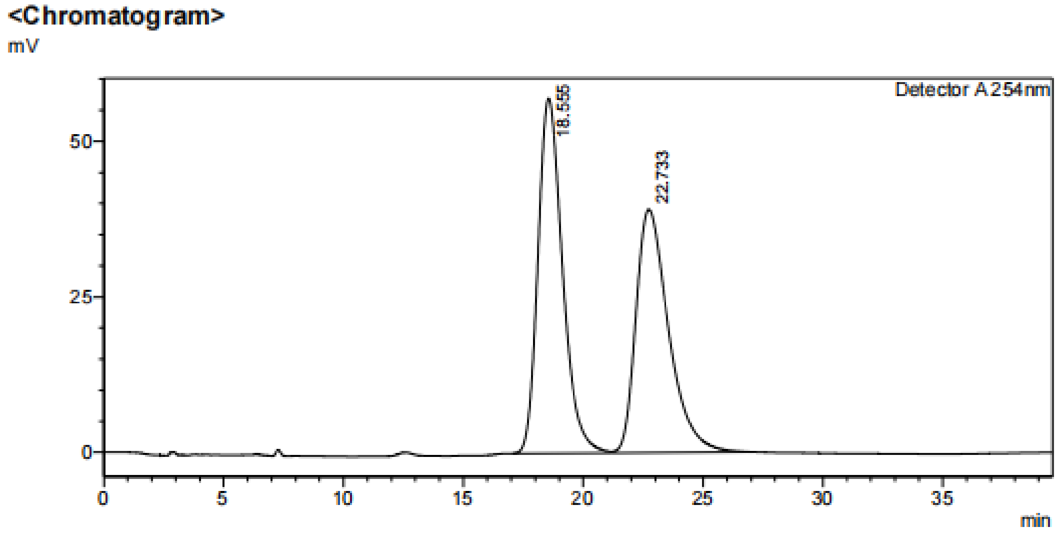

<Peak Table>

Detector A 254nm

Peak#	Ret. Time	Area	Height	Conc.	Unit	Mark	Name
1	18.555	4059924	57056	52.191			
2	22.733	3719018	39100	47.809		V	
Total		7778942	96157				

HPLC chromatogram of **12-5**

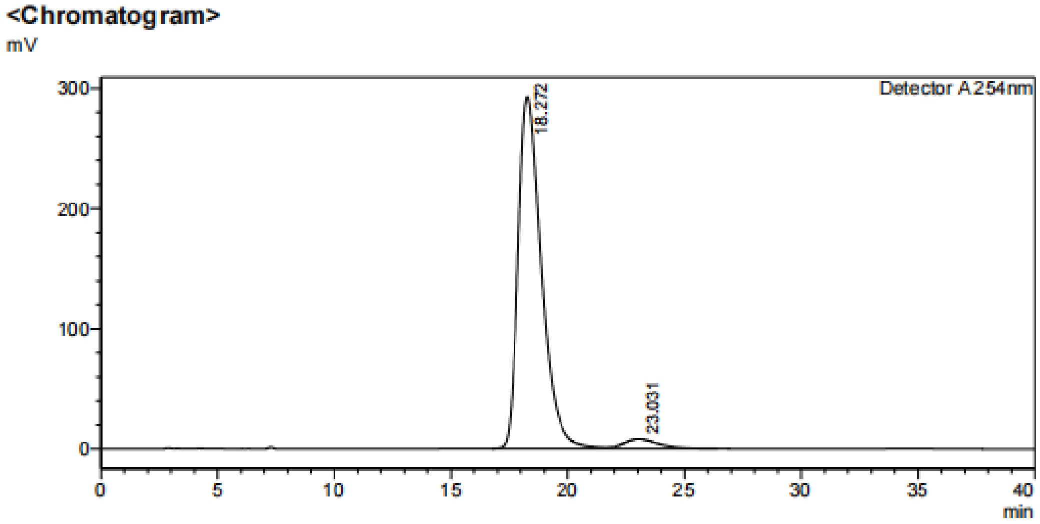

<Peak Table>

Detector A 254nm

Peak#	Ret. Time	Area	Height	Conc.	Unit	Mark	Name
1	18.272	20170878	292632	96.001			
2	23.031	840172	8403	3.999		V	
Total		21011049	301035				

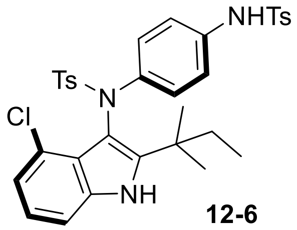

12-6

HPLC conditions: Cel-X (Guangzhou Starchrom Technologies Co., Ltd); hexane = 0.70 mL/min, isopropanol = 0.30 mL/min; T = 40 ^{o}C; λ = 254 nm; t_R (major) = 27.7 min, t_R (minor) = 41.4 min.

HPLC chromatogram of racemic **12-6**

<Chromatogram>

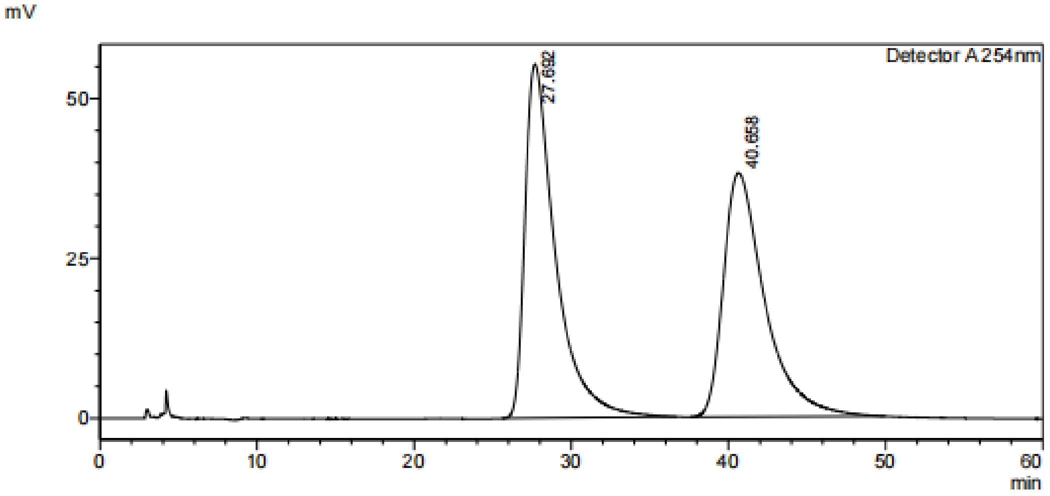

<Peak Table>

Detector A 254nm

Peak#	Ret. Time	Area	Height	Conc.	Unit	Mark	Name
1	27.692	7492267	55330	51.754			
2	40.658	6984407	38113	48.246			
Total		14476674	93443				

HPLC chromatogram of **12-6**

<Chromatogram>

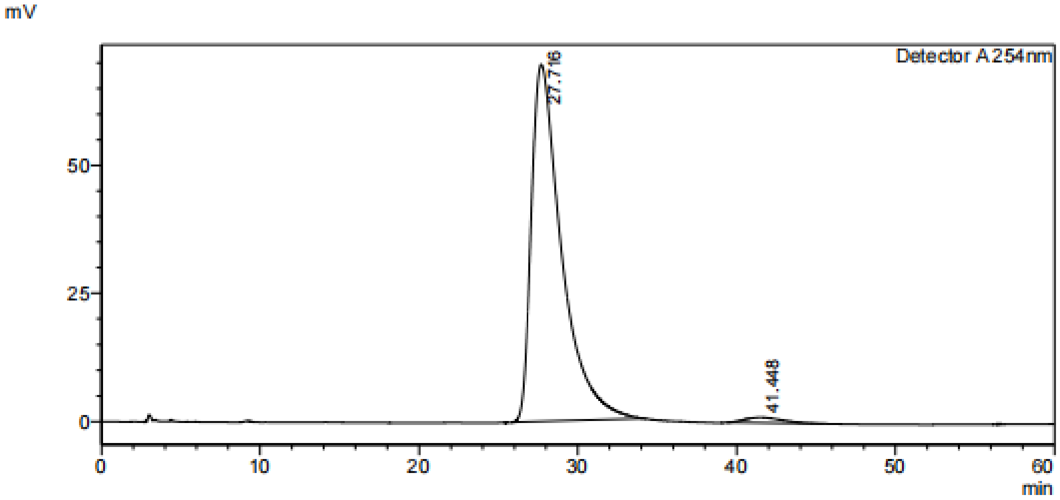

<Peak Table>

Detector A 254nm

Peak#	Ret. Time	Area	Height	Conc.	Unit	Mark	Name
1	27.716	9243565	69596	98.133		M	
2	41.448	175846	1077	1.867		M	
Total		9419411	70672				

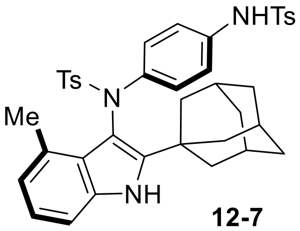

12-7

HPLC conditions: IMB (Guangzhou Starchrom Technologies Co., Ltd); hexane = 0.70 mL/min, isopropanol = 0.30 mL/min; T = 40 °C; λ = 254 nm; t_R (major) = 9.9 min, t_R (minor) = 16.5 min.

HPLC chromatogram of racemic **12-7**

<Chromatogram>

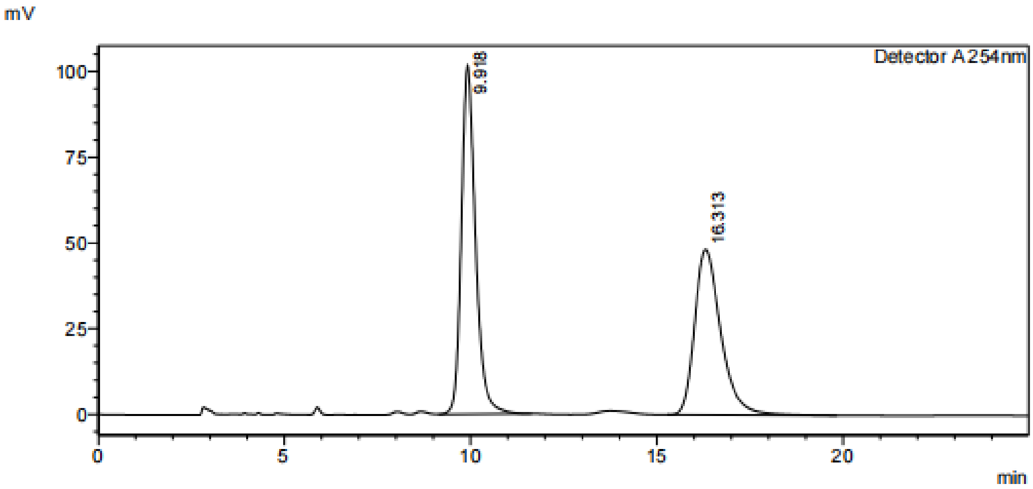

<Peak Table>

Detector A 254nm

Peak#	Ret. Time	Area	Height	Conc.	Unit	Mark	Name
1	9.918	2707541	101774	53.383			
2	16.313	2364337	48180	46.617			
Total		5071878	149955				

HPLC chromatogram of **12-7**

<Chromatogram>

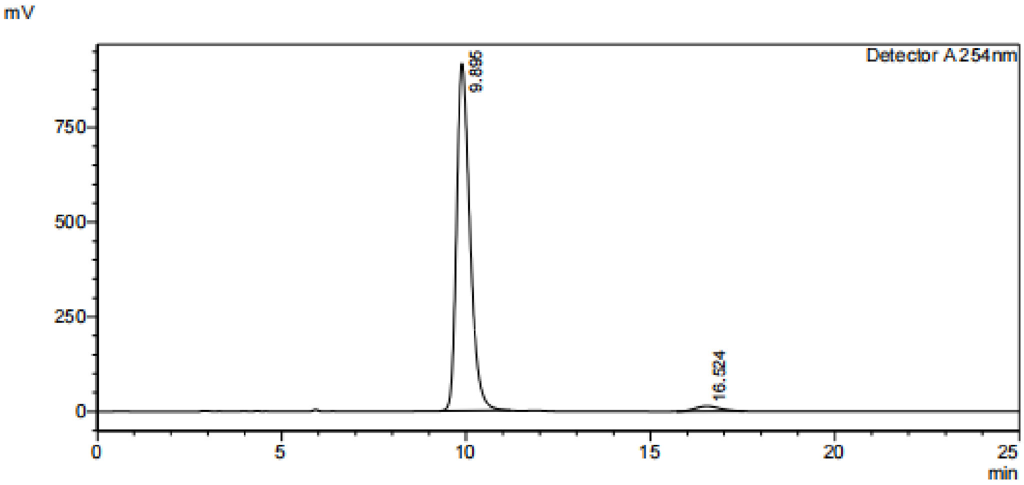

<Peak Table>

Detector A 254nm

Peak#	Ret. Time	Area	Height	Conc.	Unit	Mark	Name
1	9.895	23049164	915426	97.396		M	
2	16.524	616300	13395	2.604		M	
Total		23665464	928822				

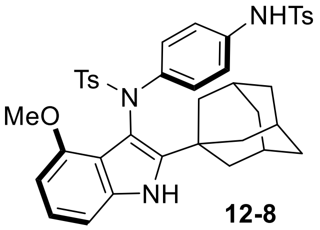

12-8

HPLC conditions: IMB (Guangzhou Starchrom Technologies Co., Ltd); hexane = 0.70 mL/min, isopropanol = 0.30 mL/min; T = 40 °C; λ = 254 nm; t_R (major) = 11.1 min, t_R (minor) = 15.5 min.

HPLC chromatogram of racemic **12-8**

<Chromatogram>

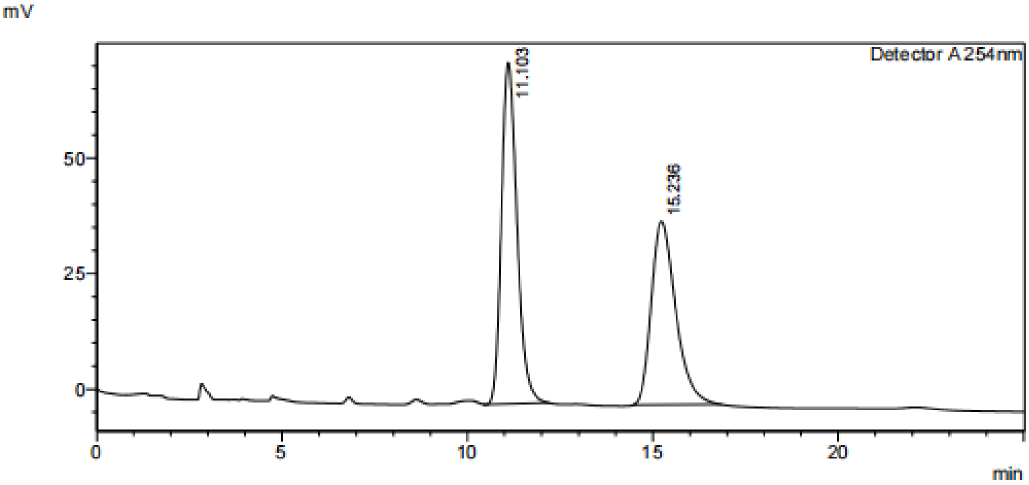

<Peak Table>

Detector A 254nm

Peak#	Ret. Time	Area	Height	Conc.	Unit	Mark	Name
1	11.103	2151275	73788	54.212		M	
2	15.236	1816971	39675	45.788		M	
Total		3968246	113463				

HPLC chromatogram of **12-8**

<Chromatogram>

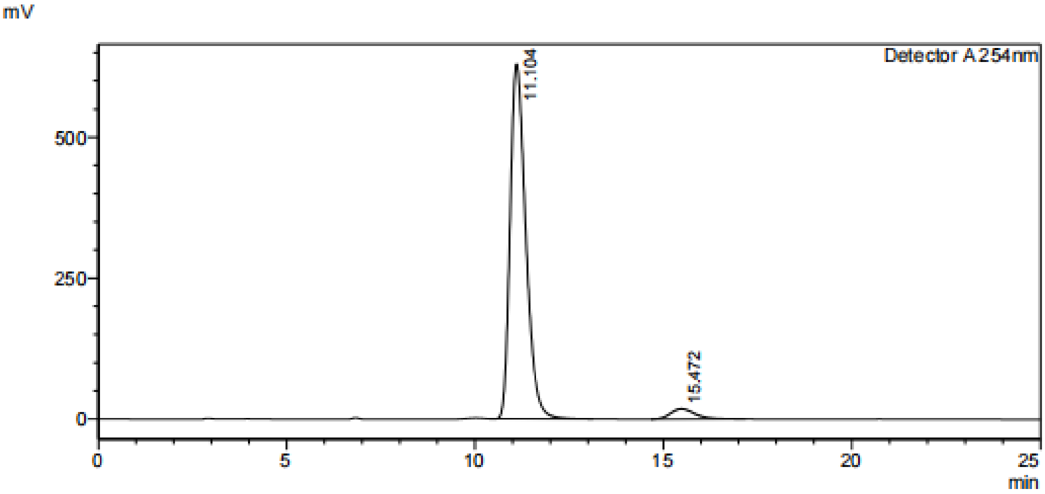

<Peak Table>

Detector A 254nm

Peak#	Ret. Time	Area	Height	Conc.	Unit	Mark	Name
1	11.104	18057983	629063	95.476		M	
2	15.472	855657	18486	4.524		M	
Total		18913639	647550				

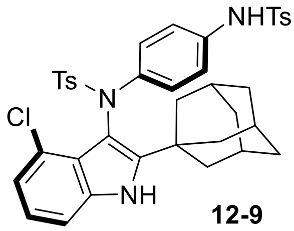

12-9

HPLC conditions: IMB (Guangzhou Starchrom Technologies Co., Ltd); hexane = 0.70 mL/min, isopropanol = 0.30 mL/min; T = 40 °C; λ = 254 nm; t_R (major) = 10.1 min, t_R (minor) = 17.9 min.

HPLC chromatogram of racemic **12-9**

<Chromatogram>

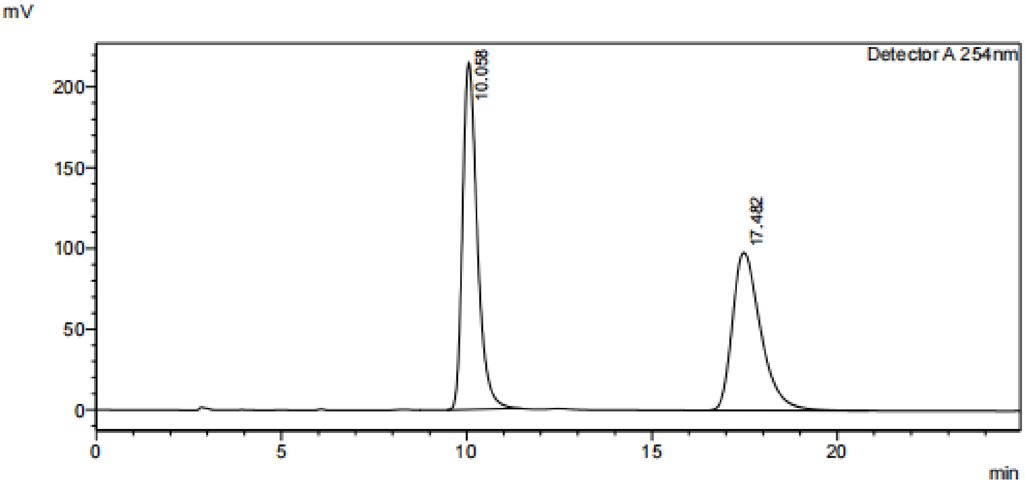

<Peak Table>

Detector A 254nm

Peak#	Ret. Time	Area	Height	Conc.	Unit	Mark	Name
1	10.058	5914344	214925	53.008		M	
2	17.482	5243062	98070	46.992			
Total		11157406	312995				

HPLC chromatogram of **12-9**

<Chromatogram>

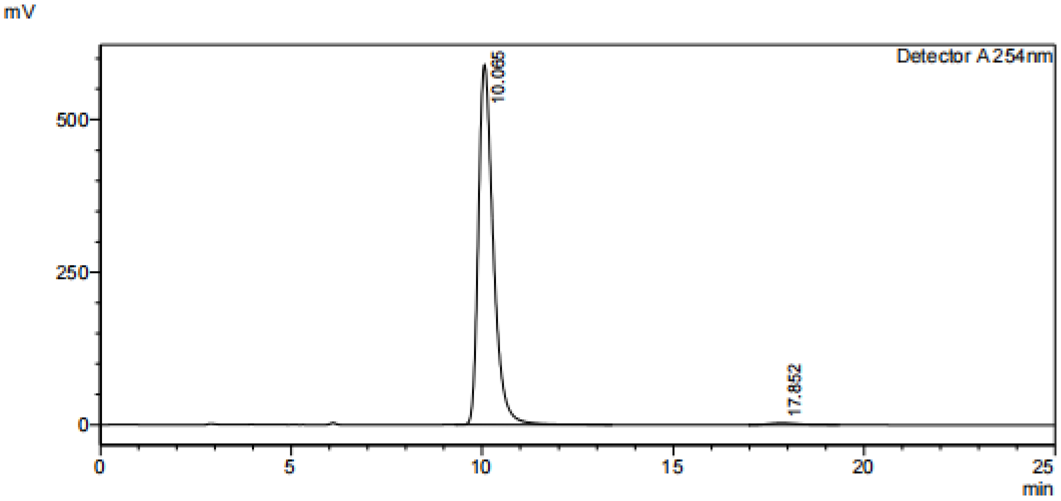

<Peak Table>

Detector A 254nm

Peak#	Ret. Time	Area	Height	Conc.	Unit	Mark	Name
1	10.065	16005866	589082	98.971		M	
2	17.852	166332	3082	1.029		M	
Total		16172197	592165				

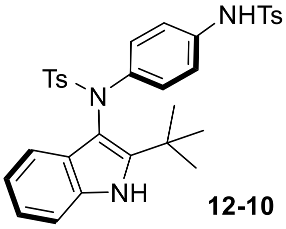

12-10

HPLC conditions: IMB (Guangzhou Starchrom Technologies Co., Ltd); hexane = 0.80 mL/min, isopropanol = 0.20 mL/min; T = 40 °C; λ = 254 nm; t_R (major) = 19.2 min, t_R (minor) = 21.7 min.

HPLC chromatogram of racemic **12-10**

<Chromatogram>

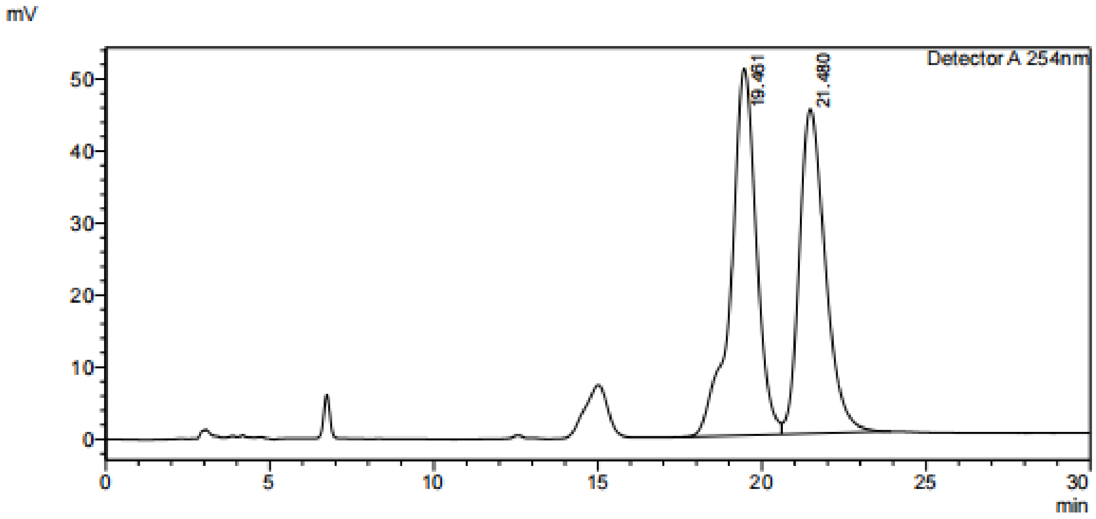

<Peak Table>

Detector A 254nm

Peak#	Ret. Time	Area	Height	Conc.	Unit	Mark	Name
1	19.461	2742219	50938	52.454			
2	21.480	2485623	45039	47.546		V	
Total		5227842	95977				

HPLC chromatogram of **12-10**

<Chromatogram>

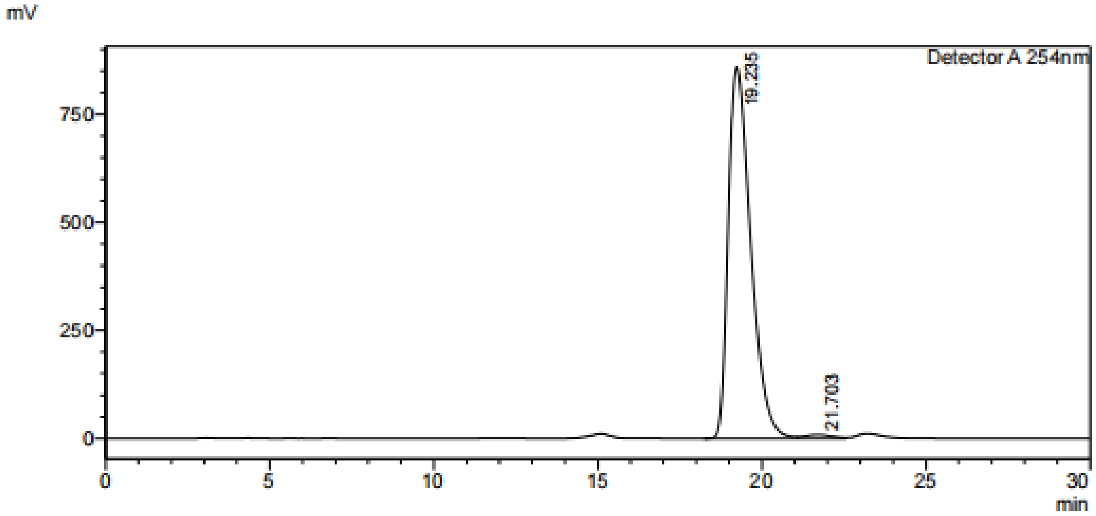

<Peak Table>

Detector A 254nm

Peak#	Ret. Time	Area	Height	Conc.	Unit	Mark	Name
1	19.235	40919185	858781	98.766			
2	21.703	511288	8793	1.234		V	
Total		41430474	867575				

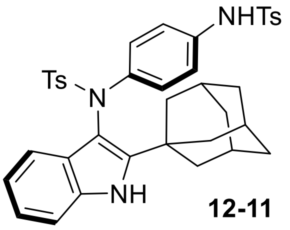

12-11

HPLC conditions: IMB (Guangzhou Starchrom Technologies Co., Ltd); hexane = 0.70 mL/min, isopropanol = 0.30 mL/min; T = 40 °C; λ = 254 nm; t_R (major) = 9.5 min, t_R (minor) = 12.8 min.

HPLC chromatogram of racemic **12-11**

<Chromatogram>

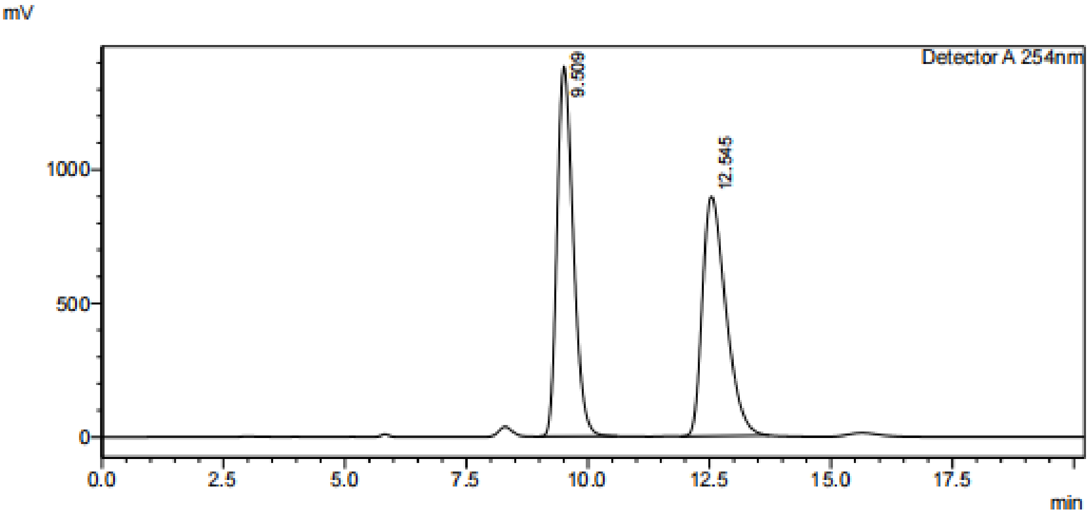

<Peak Table>

Detector A 254nm

Peak#	Ret. Time	Area	Height	Conc.	Unit	Mark	Name
1	9.509	32165316	1381083	51.649		M	
2	12.545	30111672	894996	48.351		M	
Total		62276988	2276080				

HPLC chromatogram of **12-11**

<Chromatogram>

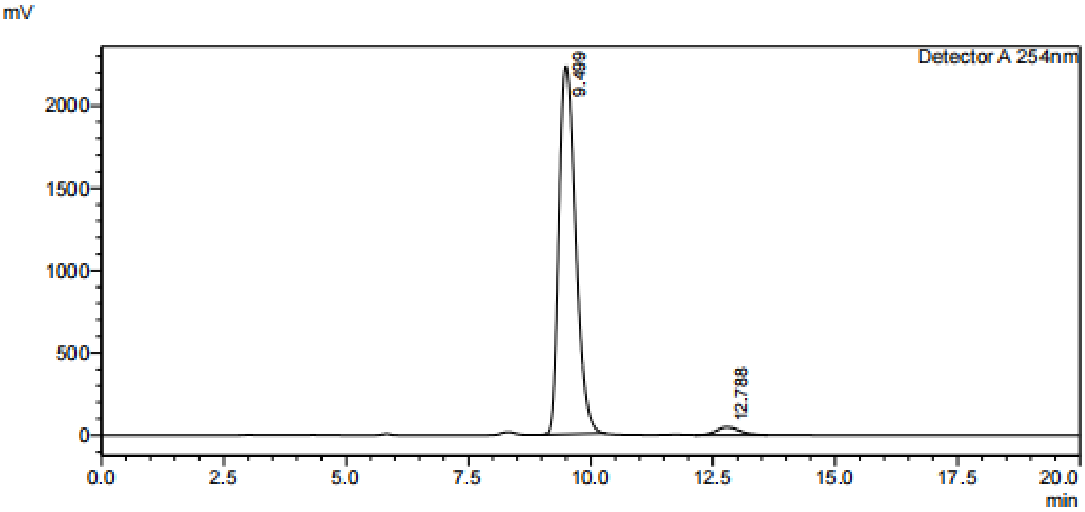

<Peak Table>

Detector A 254nm

Peak#	Ret. Time	Area	Height	Conc.	Unit	Mark	Name
1	9.499	52950547	2230185	97.172		M	
2	12.788	1541098	47138	2.828		M	
Total		54491645	2277323				

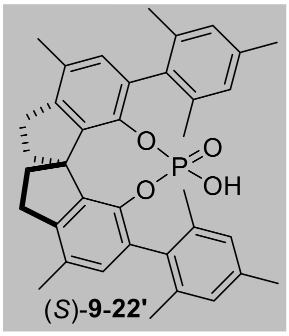
(*S*)-**9-22'**

Chiral phosphoric acid (*S*)-**9-22′** is commercially available.

7. Determination of the absolute configuration of the products

Compound **12-1** has been reported in the reference (*Angew. Chem. Int. Ed.* **2022**, *61*, e202205159). Chiral resolution of **12-1** prepared by the literature method and by our method under the same conditions showed that they correspond to the same configuration. So the absolute configuration of the products prepared by our method is consistent with that reported in the literature.

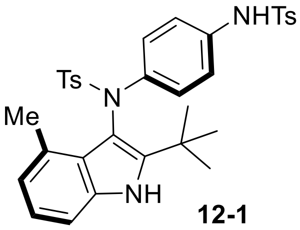

12-1

HPLC conditions: Cel-Z (Guangzhou Starchrom Technologies Co., Ltd); hexane = 0.65 mL/min, isopropanol = 0.35 mL/min; T = 40 °C; λ = 254 nm.

HPLC chromatogram of racemic **12-1**

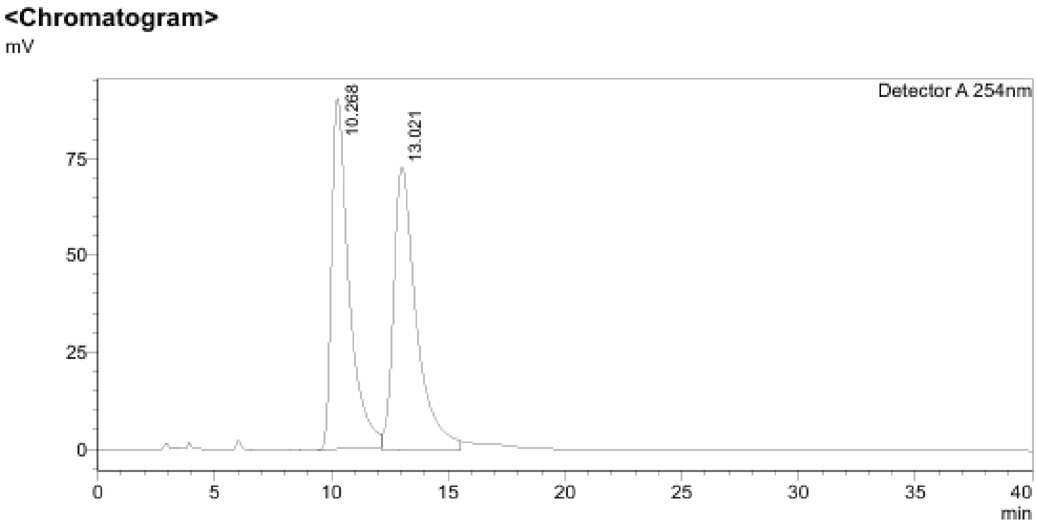

<Peak Table>

Detector A 254nm

Peak#	Ret. Time	Area	Height	Conc.	Unit	Mark	Name
1	10.268	4660158	90454	48.534			
2	13.021	4941674	72863	51.466		M	
Total		9601833	163317				

HPLC chromatogram of **12-1** (Synthesized according to the literature method)

<Chromatogram>

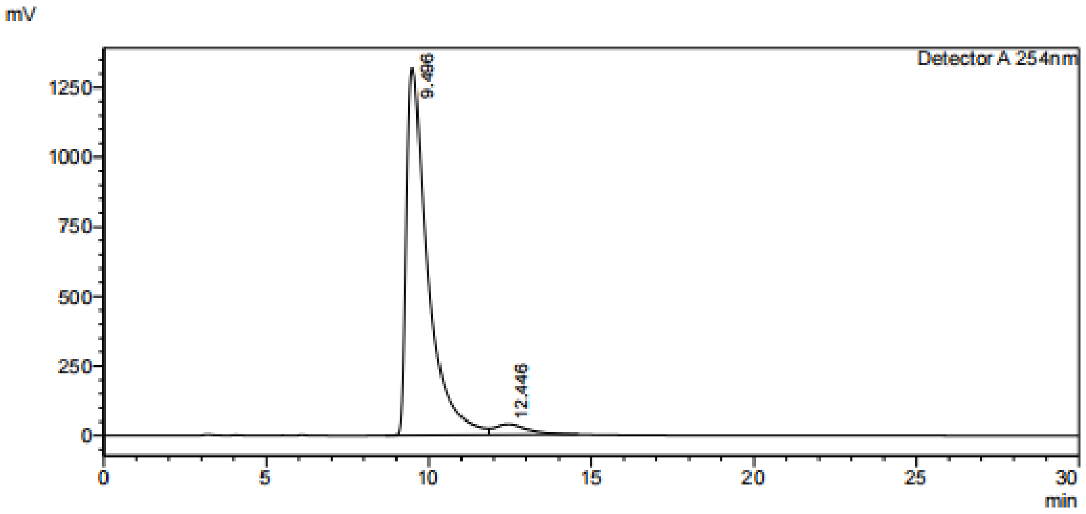

<Peak Table>

Detector A 254nm

Peak#	Ret. Time	Area	Height	Conc.	Unit	Mark	Name
1	9.496	60121485	1318729	95.651		M	
2	12.446	2733827	38851	4.349		V M	
Total		62855313	1357580				

HPLC chromatogram of **12-1** (Synthesized according to our method)

<Chromatogram>

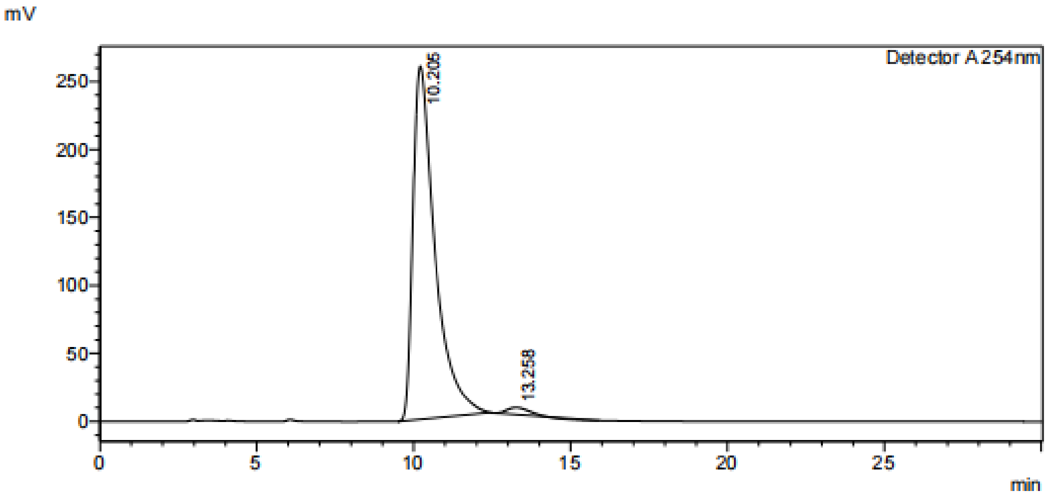

<Peak Table>

Detector A 254nm

Peak#	Ret. Time	Area	Height	Conc.	Unit	Mark	Name
1	10.205	12623501	259780	98.325		M	
2	13.258	215039	5199	1.675		M	
Total		12838540	264979				

8. Scale-up experiment

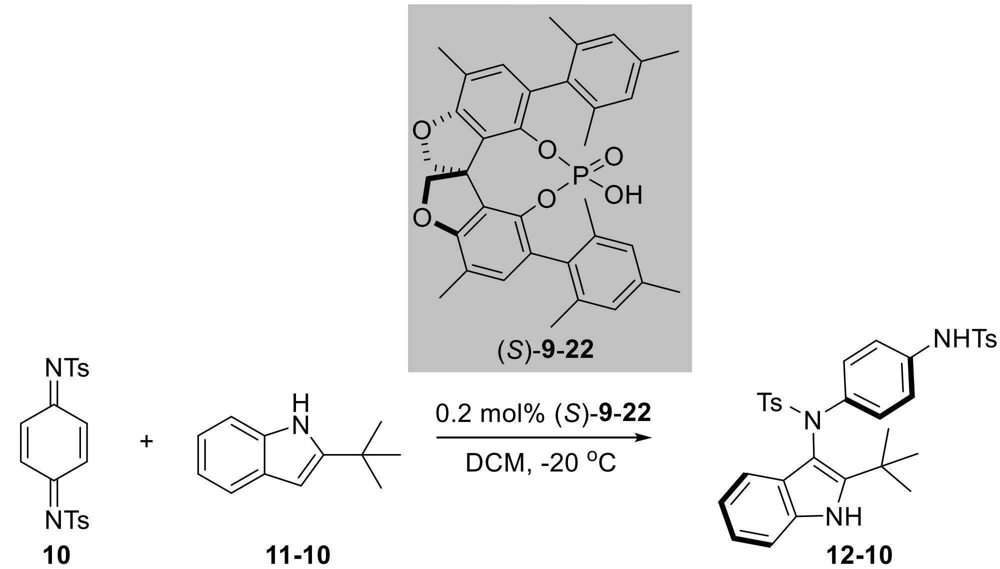

10 **11-10** **12-10**

Into an oven dried Schlenk tube chiral phosphoric acid (*S*)-**9-22** (0.2 mol%, 1.17 mg), **11-10** (2.0 mmol, 0.35 g) and DCM (10 mL) were added. The reaction mixture was stirred at -20 °C for 10 min, then **10** (1.0 mmol, 0.42 g) was added. The reaction was stirred at -20 °C when TLC analysis indicated that the starting material **10** was completely consumed (about 1h). Solvent was removed under reduced pressure and the crude residue was purified by column chromatography on silica gel (PE/EA = 1/1) to give pure product **12-10**. 0.58 g, 99% yield, 98% ee.

9. Single-crystal X-ray diffraction data

Single-crystal X-ray crystallography of (*S*)-7

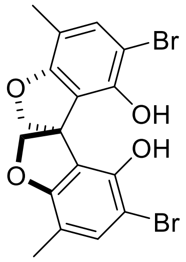

(*S*)-**7**

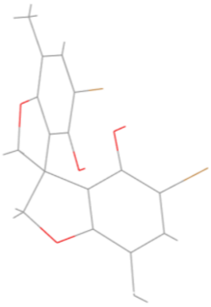

CCDC 2584202

Table 1 Crystal data and structure refinement for 260705_CJJ_329_2.

Identification code	260705_CJJ_329_2
Empirical formula	$C_{17}H_{14}Br_2O_4$
Formula weight	442.10
Temperature/K	170.00
Crystal system	monoclinic
Space group	$P2_1$
a/Å	9.3900(5)
b/Å	15.2433(8)
c/Å	17.4616(10)
α/°	90
β/°	97.700(2)
γ/°	90
Volume/Å^3	2476.8(2)
Z	6
ρ_{calc}g/cm^3	1.778
μ/mm^{-1}	4.357
F(000)	1308.0
Crystal size/mm^3	0.4 × 0.13 × 0.1
Radiation	GaKα (λ = 1.34139)
2Θ range for data collection/°	4.442 to 121.432
Index ranges	-12 ≤ h ≤ 12, -18 ≤ k ≤ 19, -22 ≤ l ≤ 22
Reflections collected	56101
Independent reflections	11189 [R_{int} = 0.0382, R_{sigma} = 0.0357]
Data/restraints/parameters	11189/1/634
Goodness-of-fit on F^2	1.045
Final R indexes [I>=2σ (I)]	R_1 = 0.0256, wR_2 = 0.0625

Final R indexes [all data]	R_1 = 0.0264, wR_2 = 0.0629
Largest diff. peak/hole / e Å^{-3}	0.43/-0.92
Flack parameter	-0.002(5)

Single-crystal X-ray crystallography of (*S*)-9-21

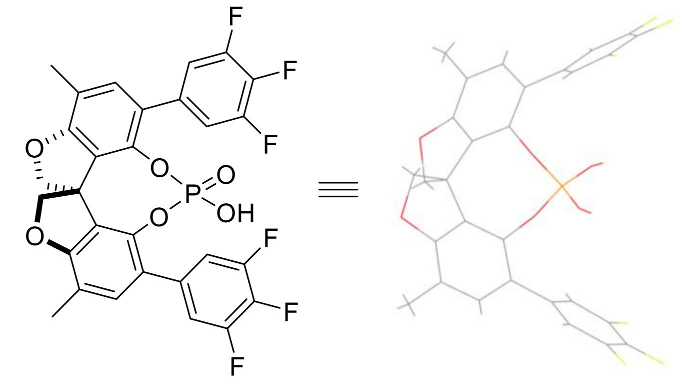

(*S*)-**9-21** CCDC 2586984

Table 2 Crystal data and structure refinement for 260907_CJJ_402_1.

Identification code	260907_CJJ_402_1
Empirical formula	$C_{89}H_{55}Cl_4F_{18}O_{18}P_3$
Formula weight	1989.04
Temperature/K	170.00
Crystal system	monoclinic
Space group	C2
a/Å	16.9621(10)
b/Å	18.4210(10)
c/Å	15.5727(9)
α/°	90
β/°	120.021(2)
γ/°	90
Volume/Å^3	4213.0(4)
Z	2
ρ_{calc}g/cm^3	1.568
μ/mm^{-1}	1.876
F(000)	2016.0
Crystal size/mm^3	0.16 × 0.09 × 0.08
Radiation	GaKα (λ = 1.34139)
2Θ range for data collection/°	5.702 to 121.63
Index ranges	-22 ≤ h ≤ 22, -23 ≤ k ≤ 23, -20 ≤ l ≤ 20
Reflections collected	64015
Independent reflections	9622 [R_{int} = 0.0353, R_{sigma} = 0.0273]
Data/restraints/parameters	9622/16/620
Goodness-of-fit on F^2	1.082
Final R indexes [I>=2σ (I)]	R_1 = 0.0393, wR_2 = 0.1079
Final R indexes [all data]	R_1 = 0.0410, wR_2 = 0.1093
Largest diff. peak/hole / e Å^{-3}	0.41/-0.81

Flack parameter	0.052(4)

10. Copies of NMR spectra

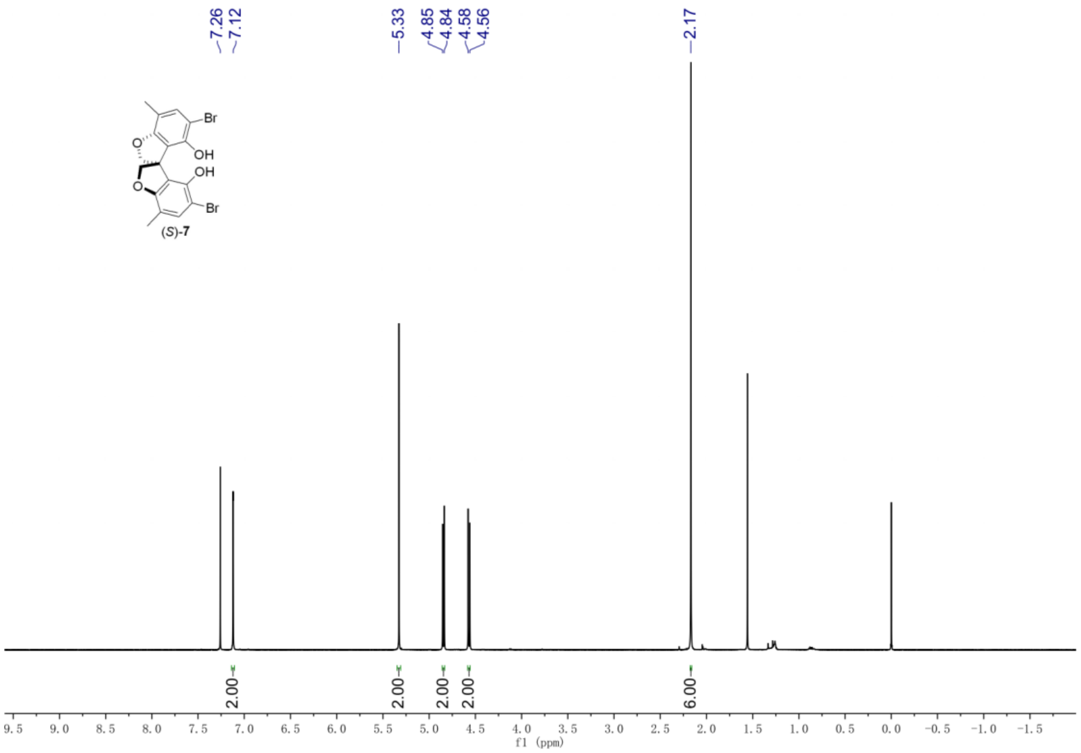

^{1}H NMR of (*S*)-**7** ($CDCl_3$)

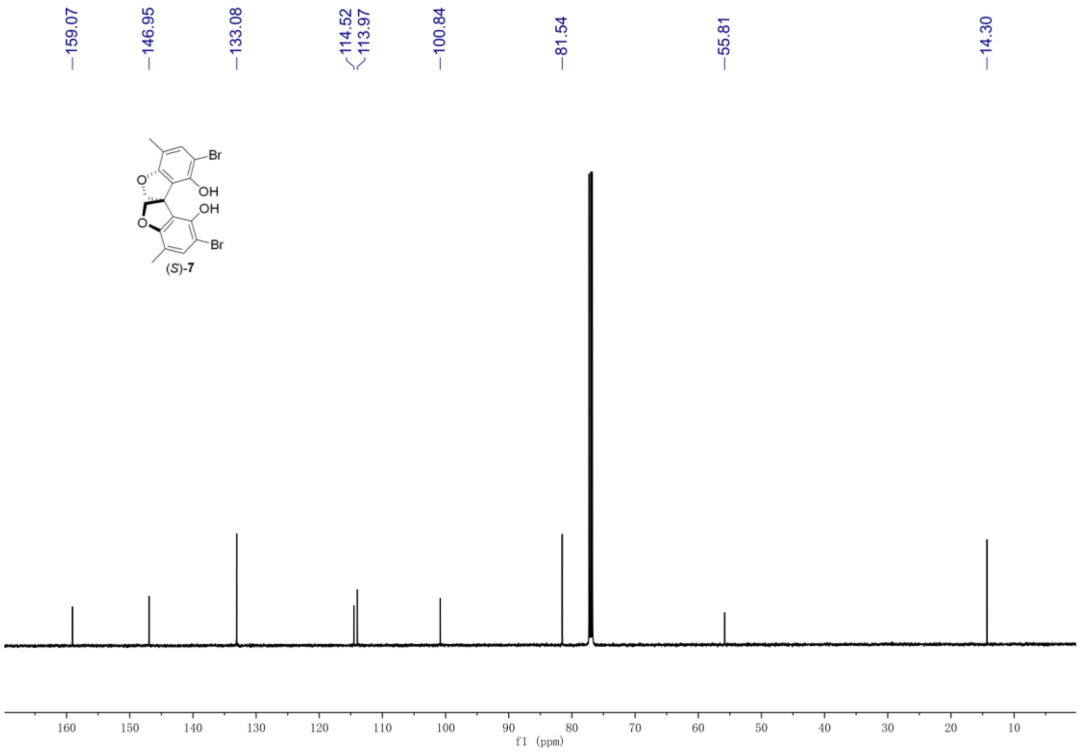

^{13}C NMR of (*S*)-**7** ($CDCl_3$)

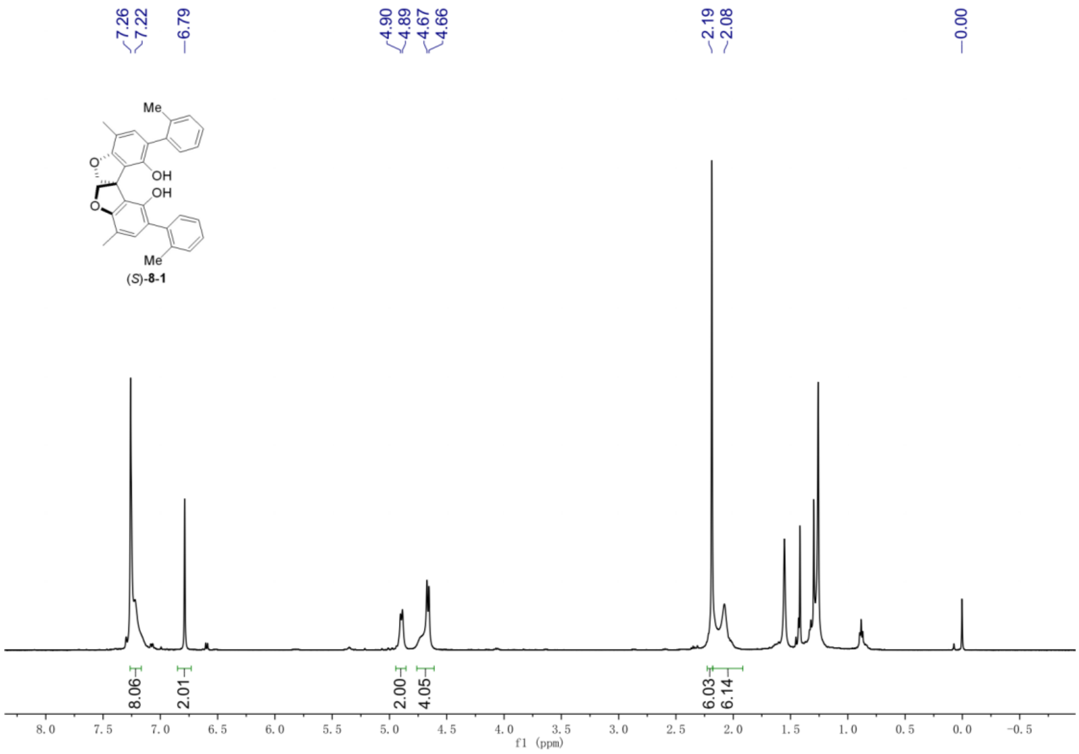

^{1}H NMR of (*S*)-**8-1** ($CDCl_3$)

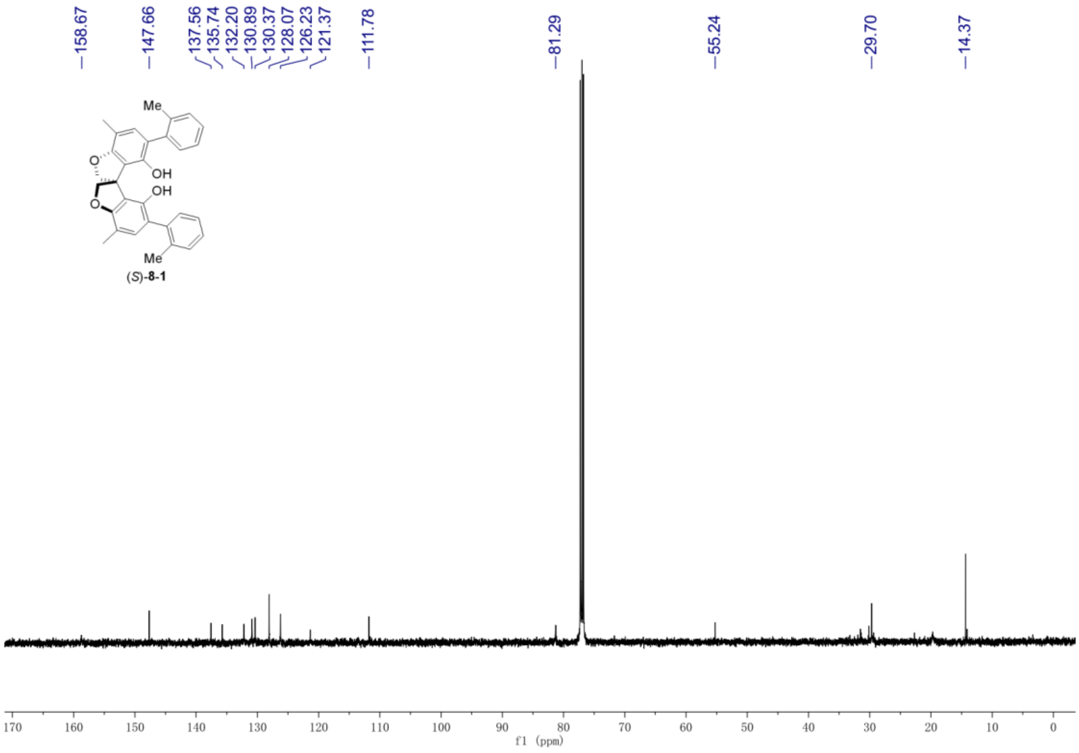

^{13}C NMR of (*S*)-**8-1** ($CDCl_3$)

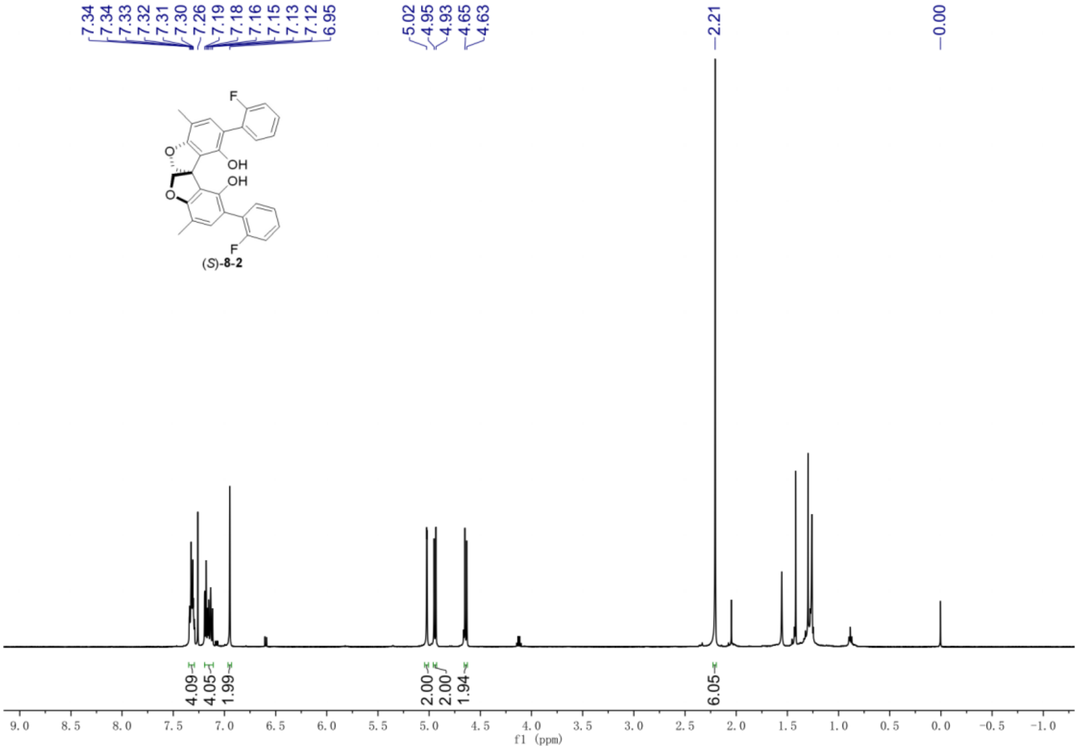

^{1}H NMR of (*S*)-**8-2** ($CDCl_3$)

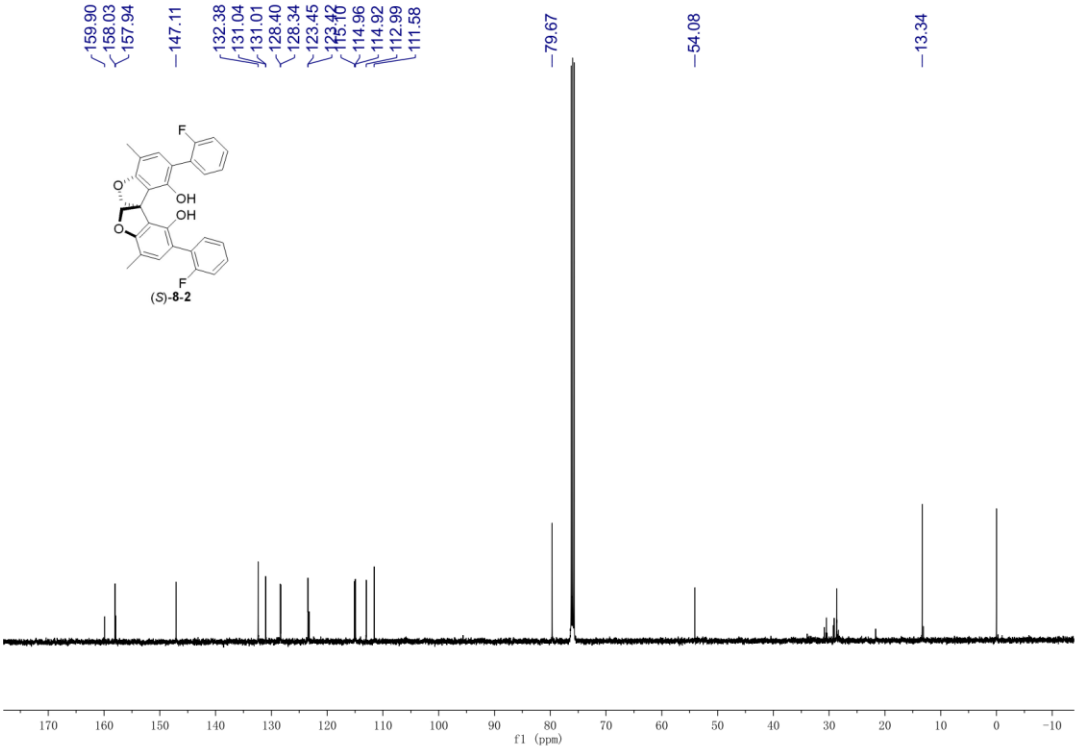

^{13}C NMR of (*S*)-**8-2** ($CDCl_3$)

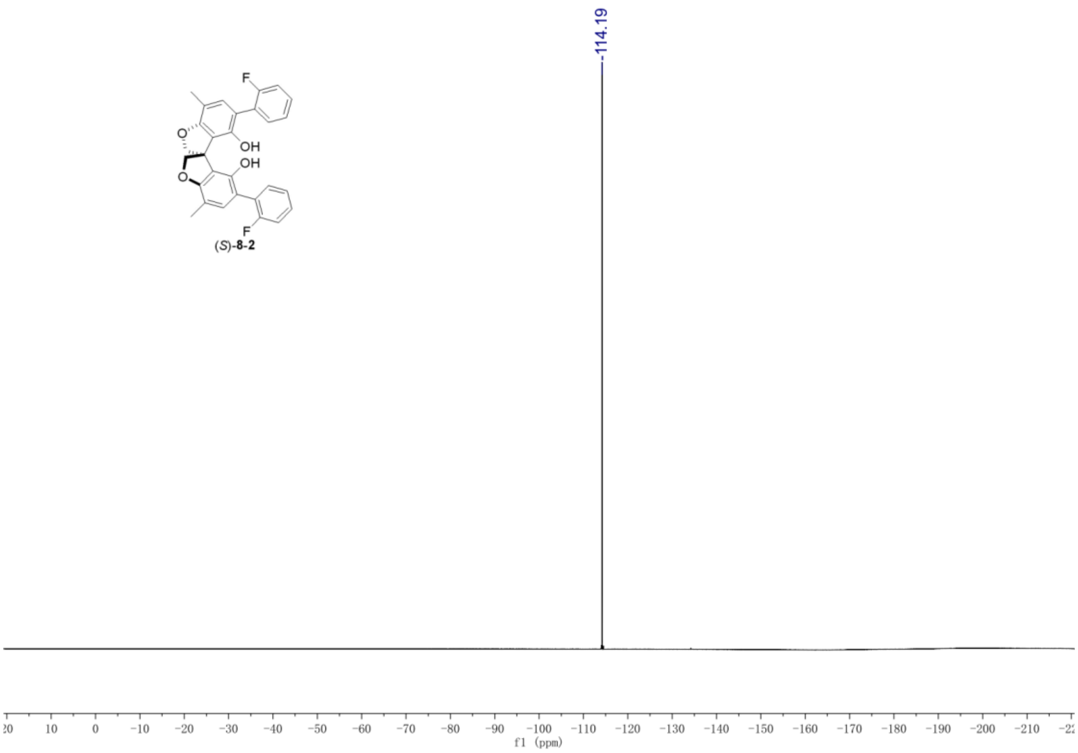

^{19}F NMR of (*S*)-**8-2** ($CDCl_3$)

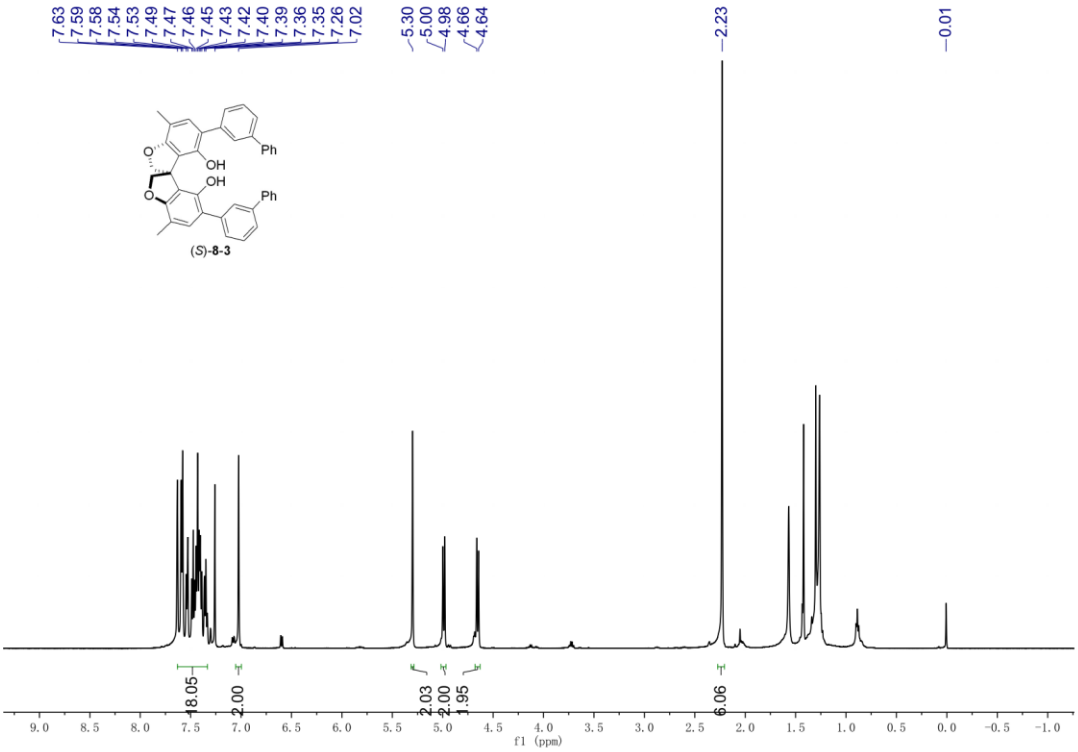

^{1}H NMR of (*S*)-**8-3** ($CDCl_3$)

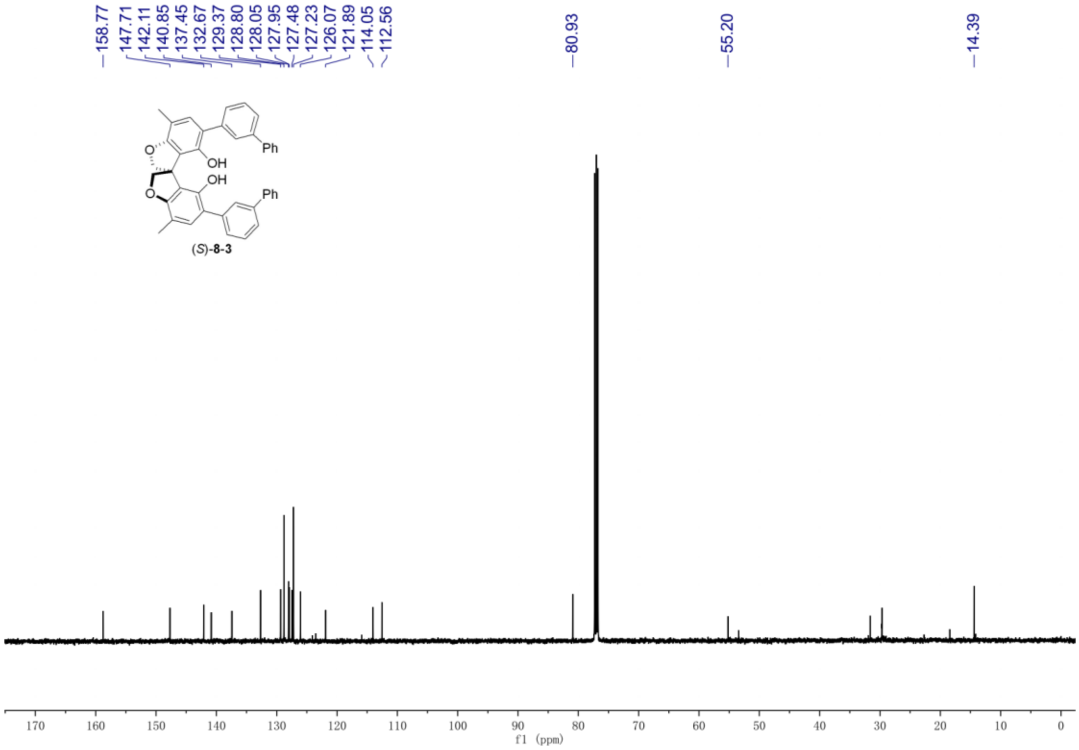

^{13}C NMR of (*S*)-**8-3** ($CDCl_3$)

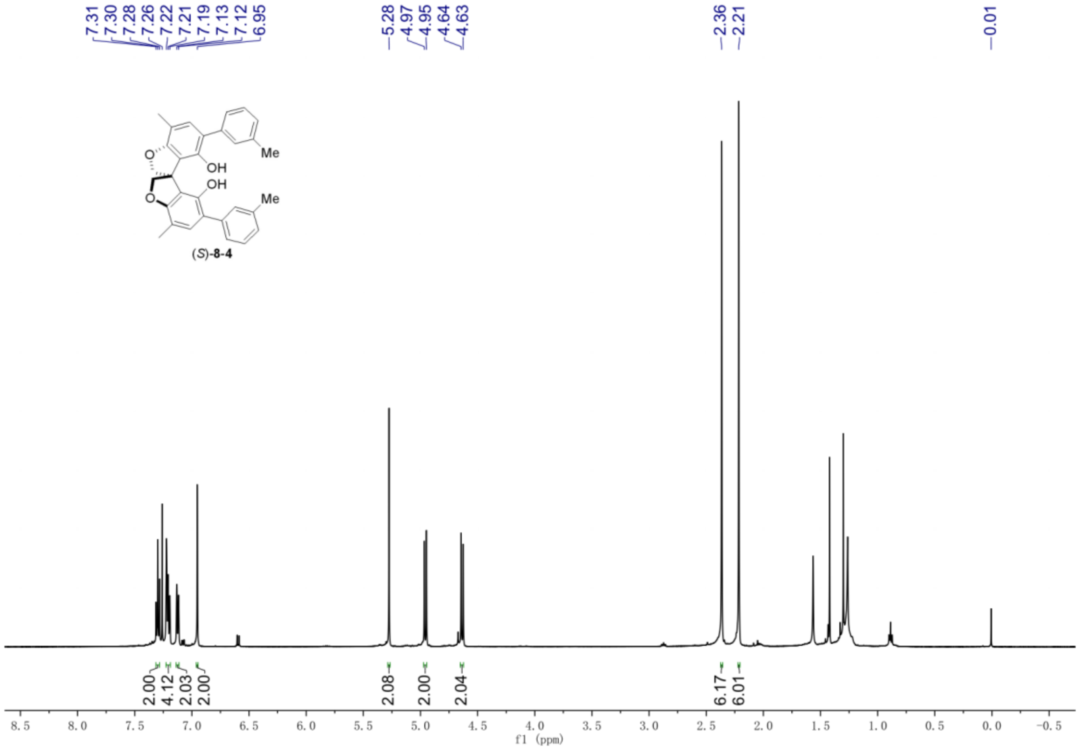

^{1}H NMR of (*S*)-**8-4** ($CDCl_3$)

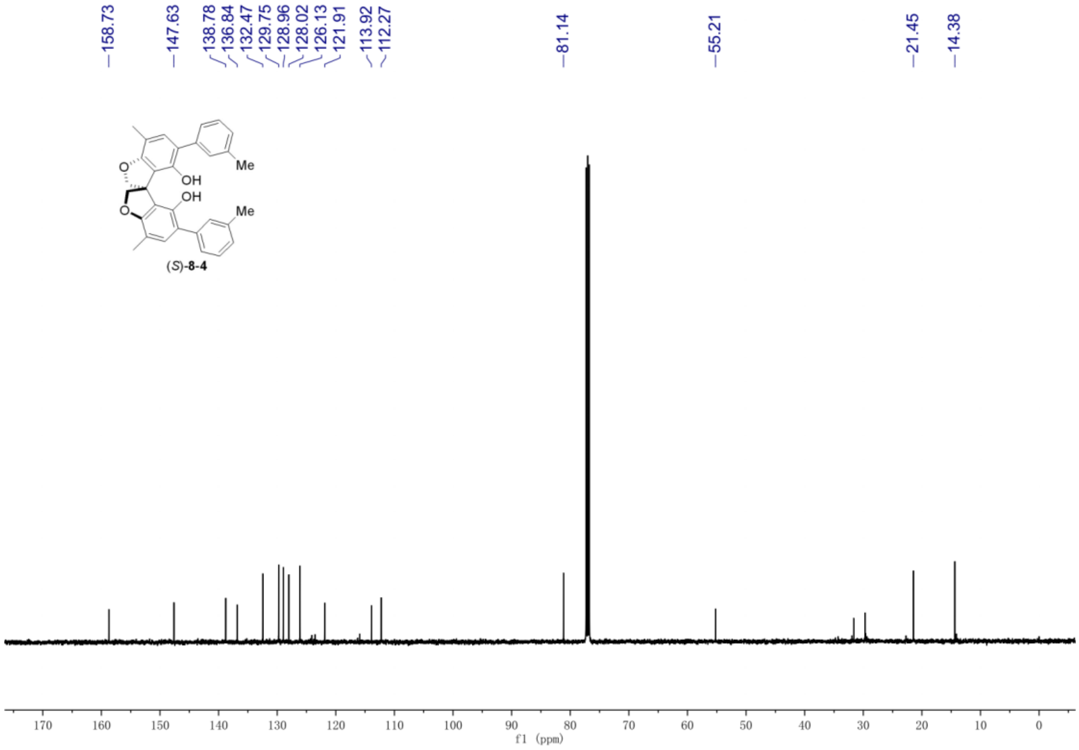

^{13}C NMR of (*S*)-**8-4** ($CDCl_3$)

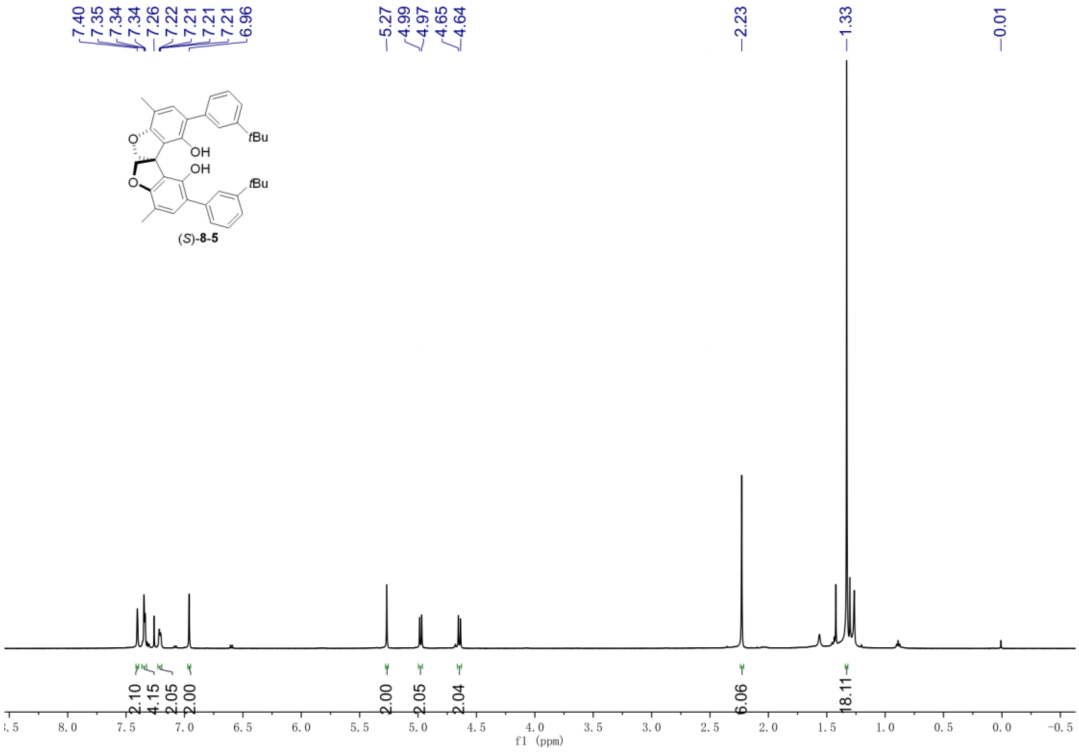

^{1}H NMR of (*S*)-**8-5** ($CDCl_3$)

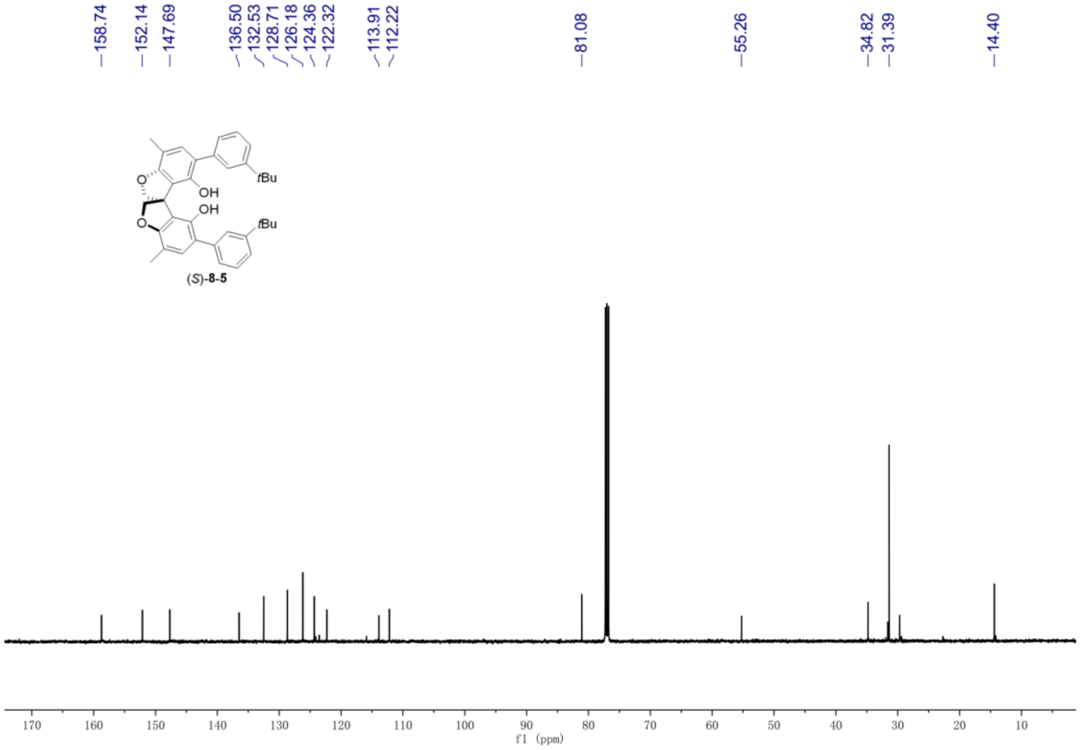

^{13}C NMR of (*S*)-**8-5** ($CDCl_3$)

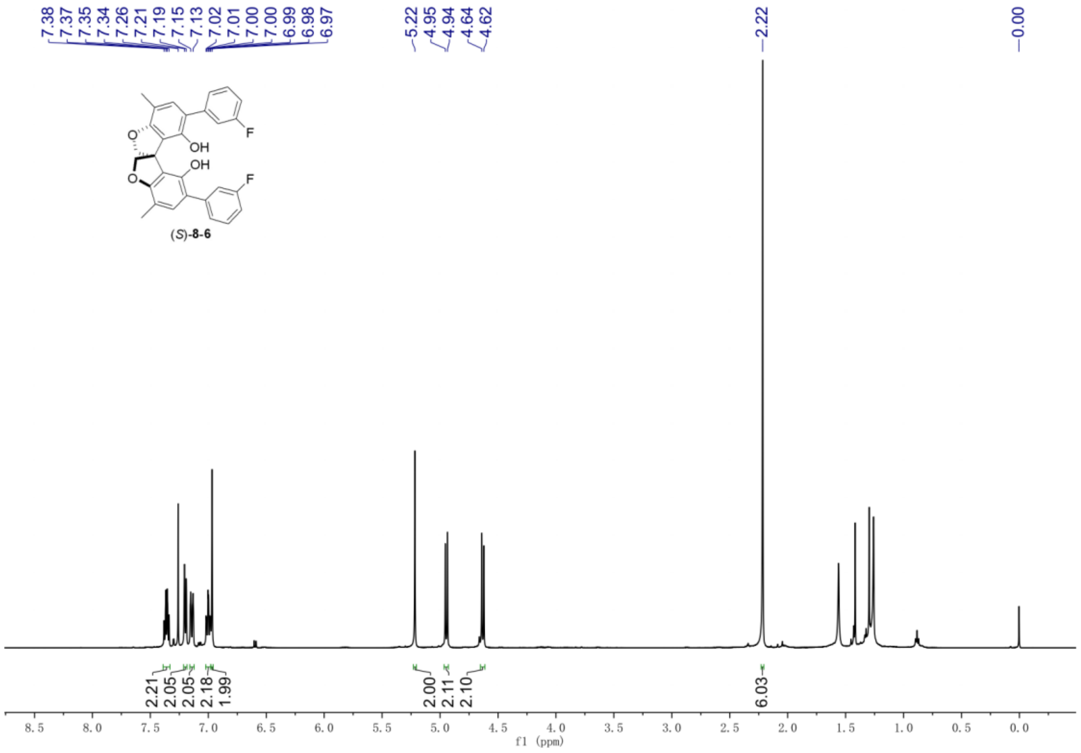

^{1}H NMR of (*S*)-**8-6** ($CDCl_3$)

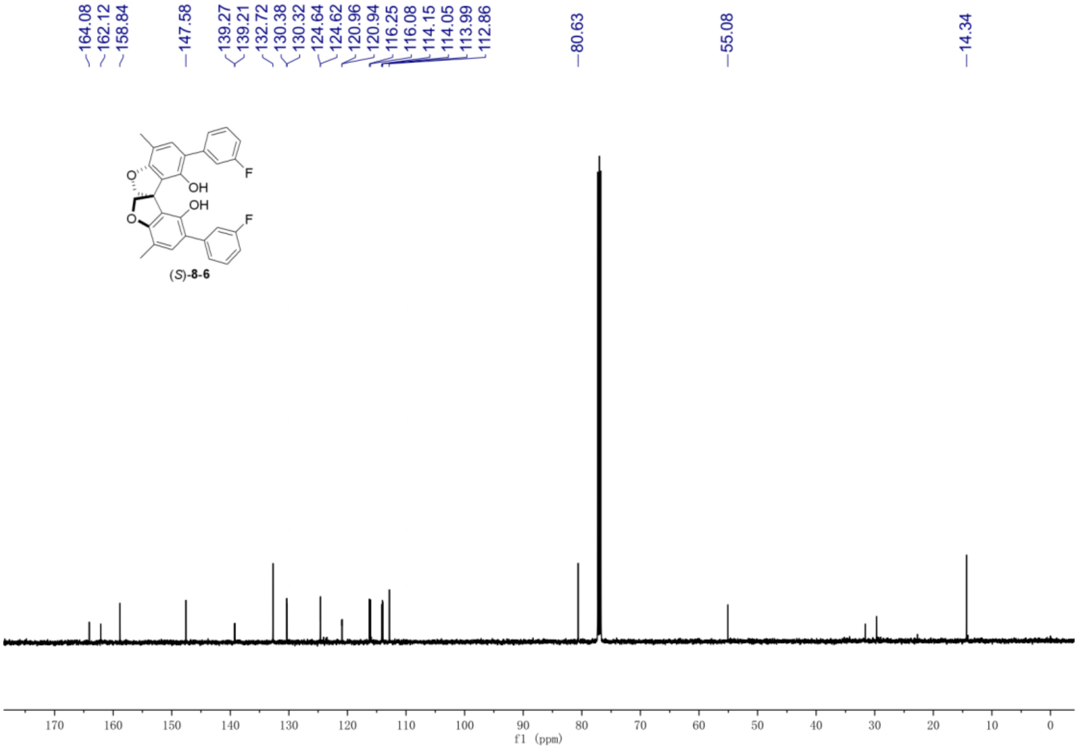

^{13}C NMR of (*S*)-**8-6** ($CDCl_3$)

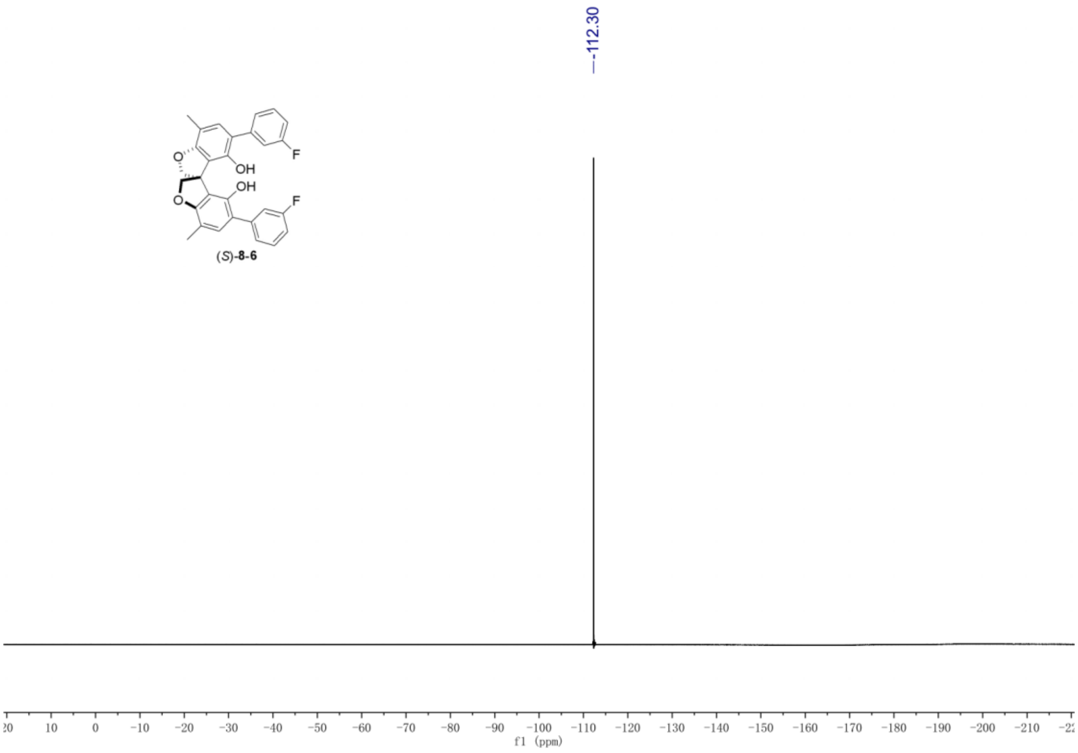

^{19}F NMR of (*S*)-**8-6** ($CDCl_3$)

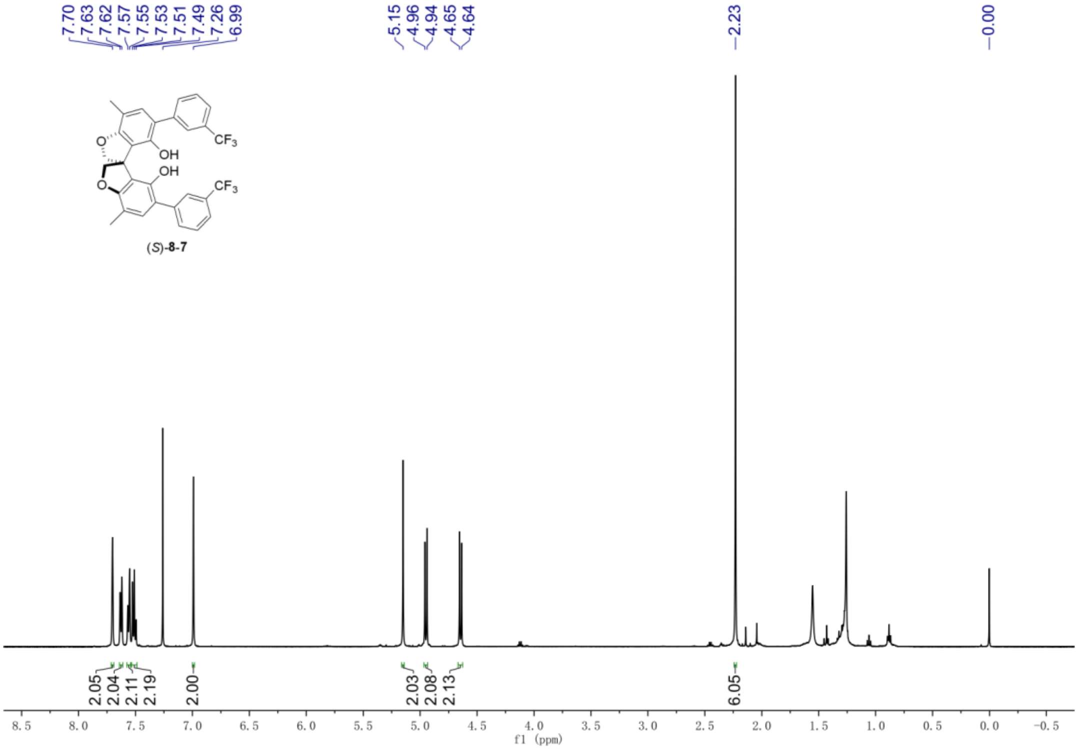

^1H NMR of (*S*)-**8-7** ($CDCl_3$)

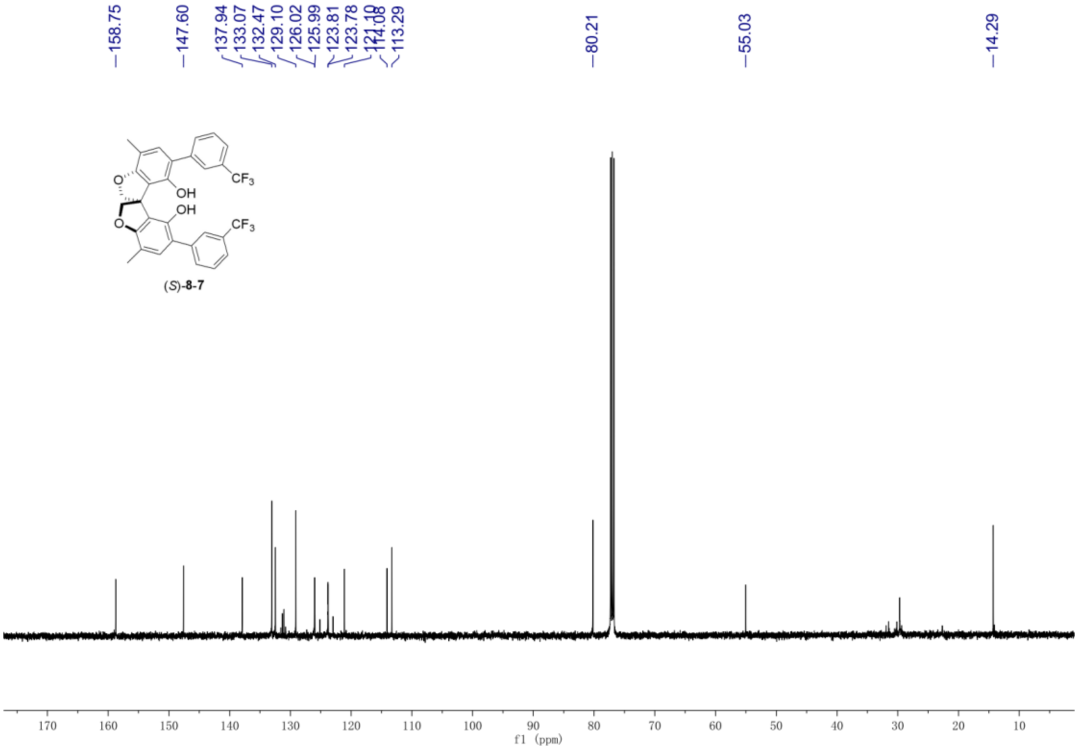

^{13}C NMR of (*S*)-**8-7** ($CDCl_3$)

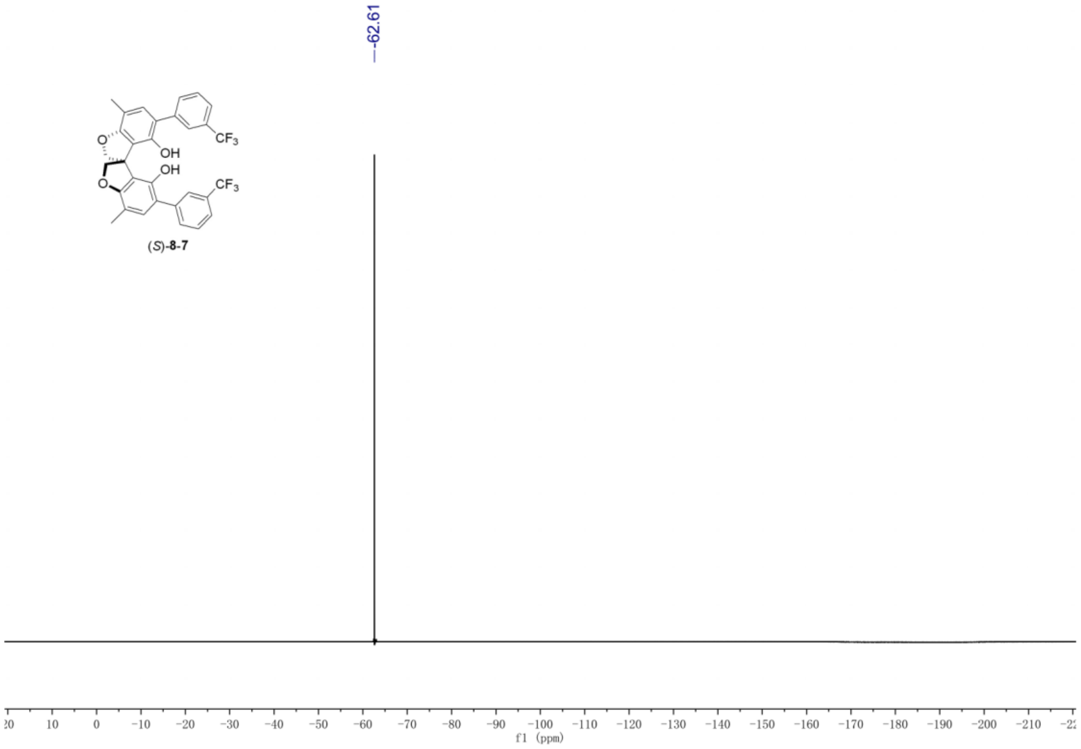

^{19}F NMR of (*S*)-**8-7** ($CDCl_3$)

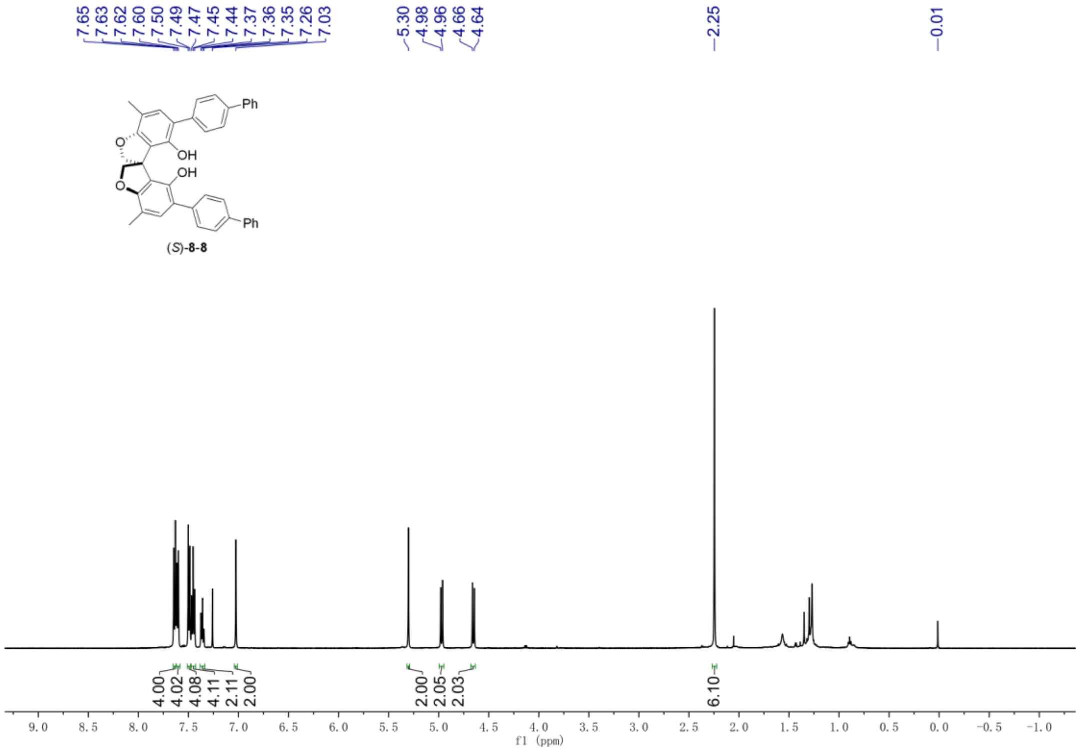

^{1}H NMR of (*S*)-**8-8** ($CDCl_3$)

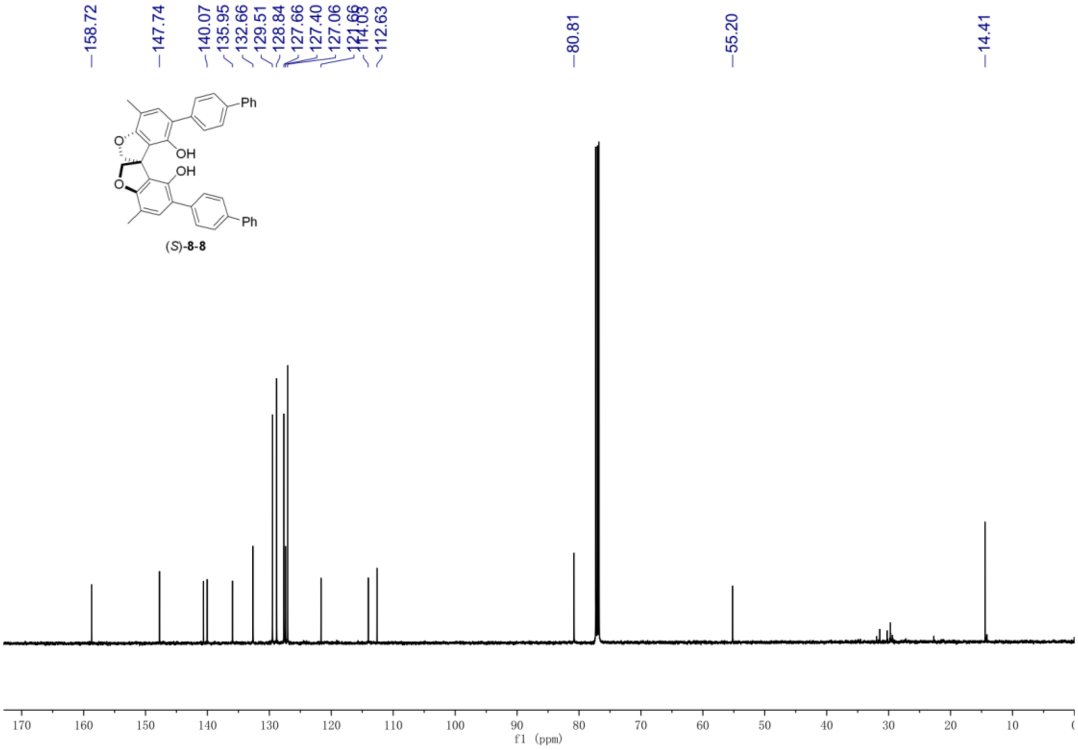

^{13}C NMR of (*S*)-**8-8** ($CDCl_3$)

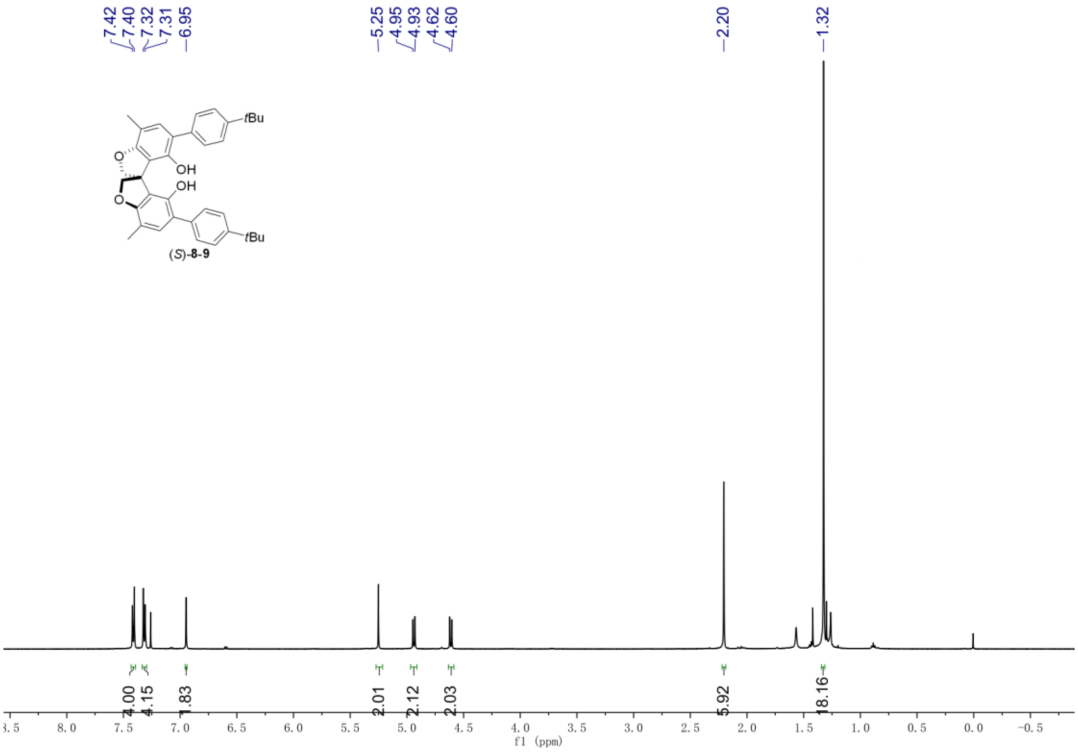

^{1}H NMR of (*S*)-**8-9** ($CDCl_3$)

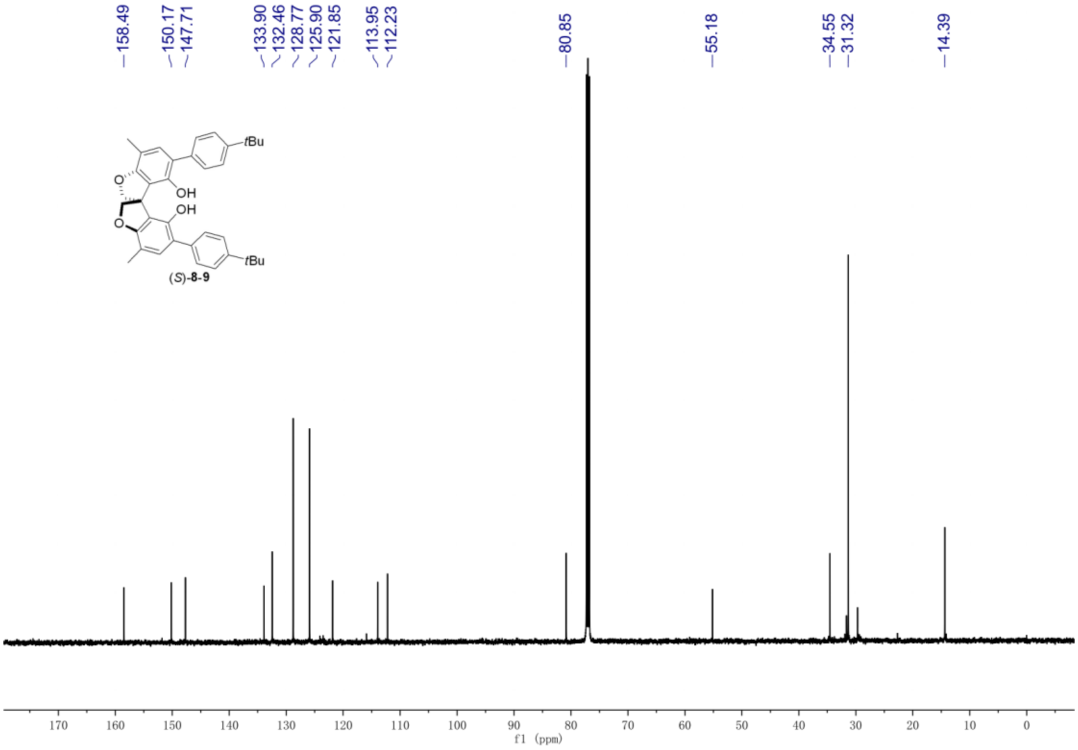

^{13}C NMR of (*S*)-**8-9** ($CDCl_3$)

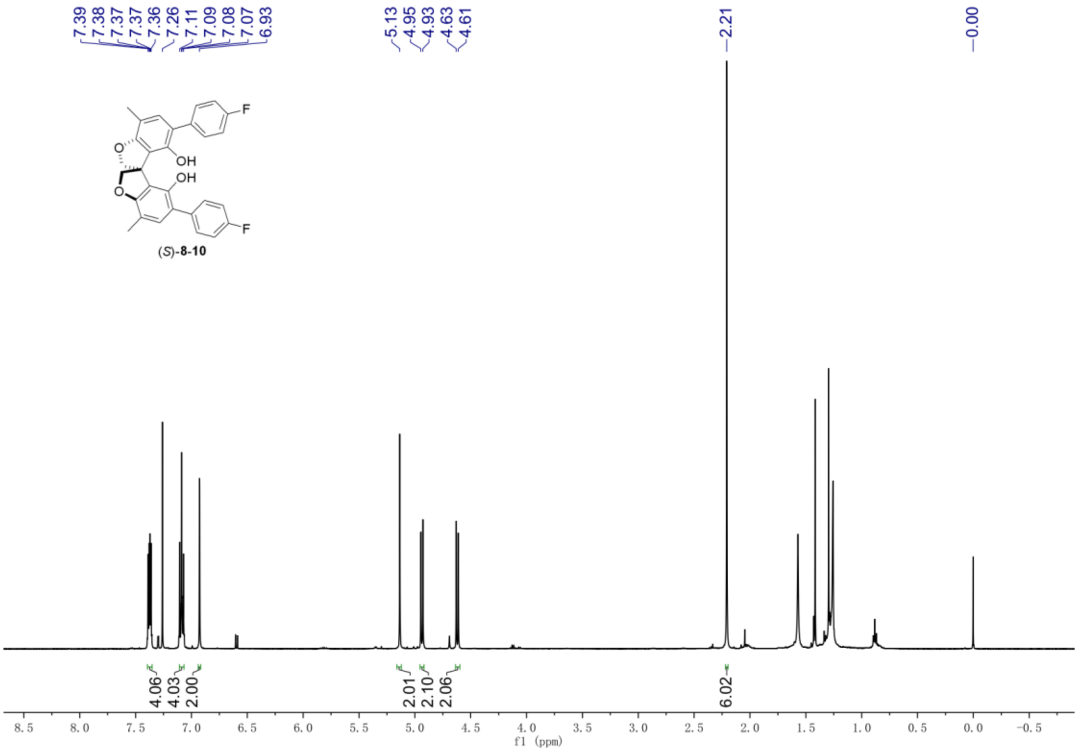

^{1}H NMR of (*S*)-**8-10** ($CDCl_3$)

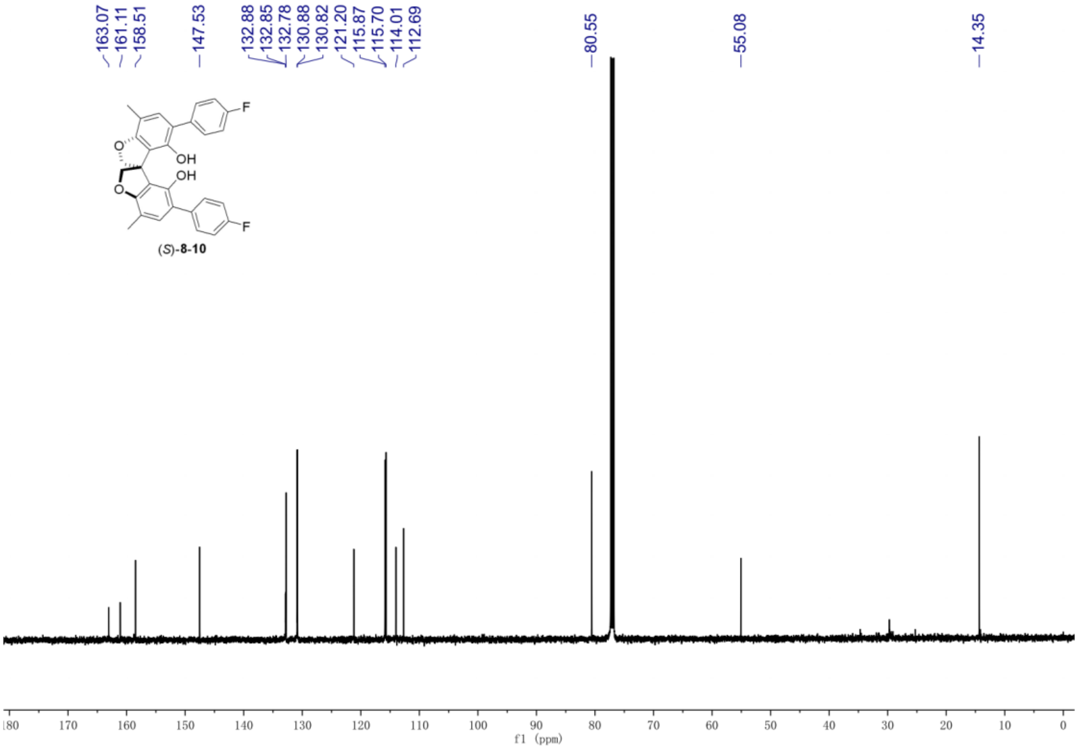

^{13}C NMR of (*S*)-**8-10** ($CDCl_3$)

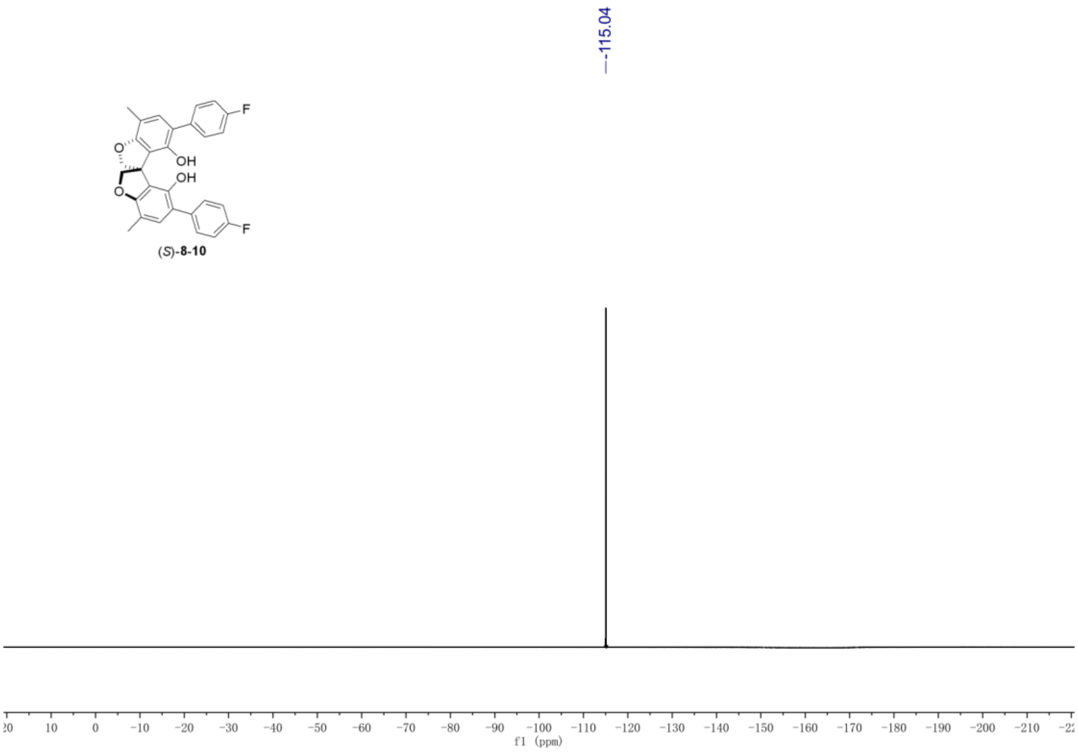

^{19}F NMR of (*S*)-**8-10** ($CDCl_3$)

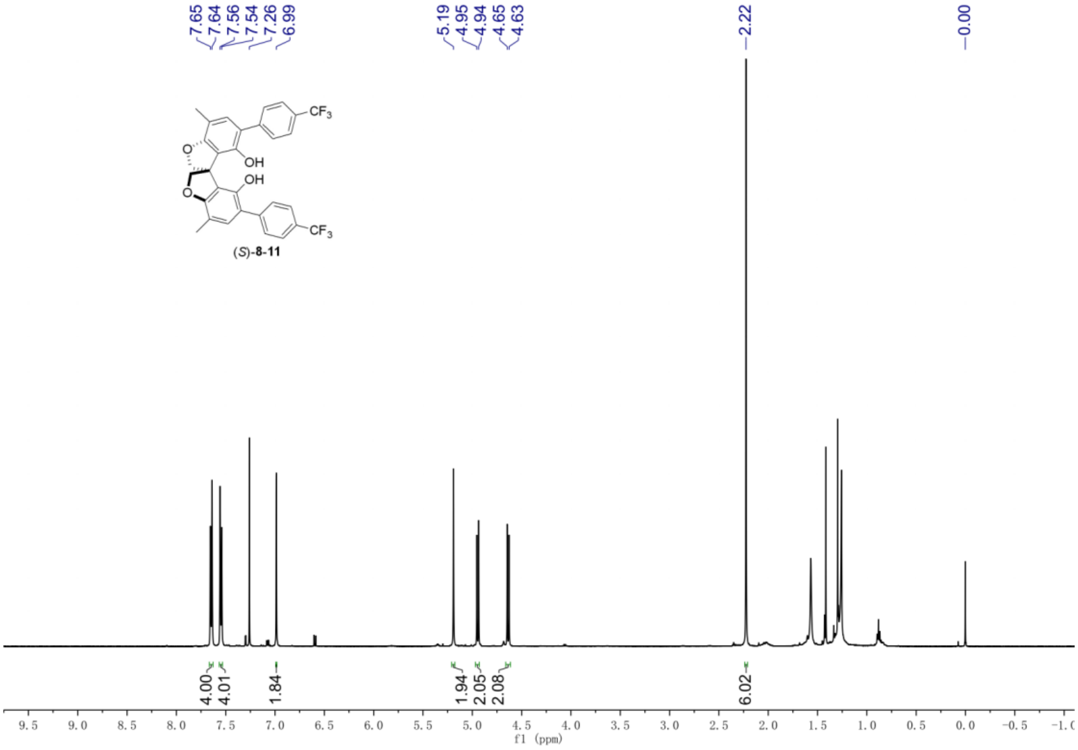

^{1}H NMR of (*S*)-**8-11** ($CDCl_3$)

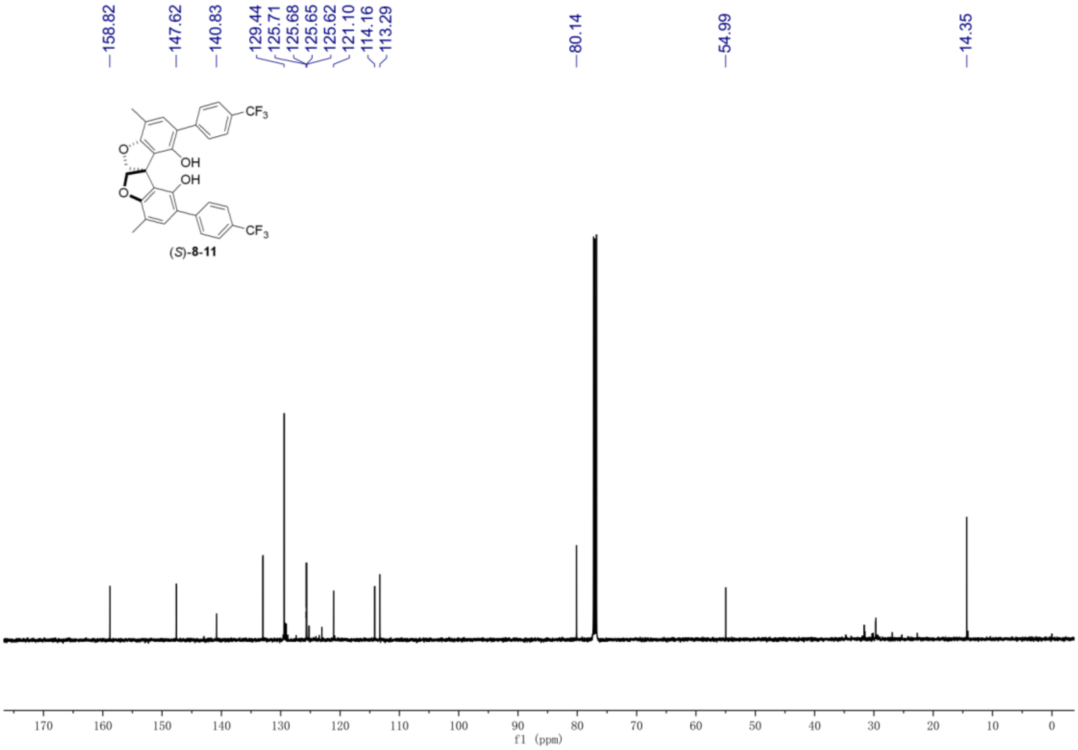

^{13}C NMR of (*S*)-**8-11** ($CDCl_3$)

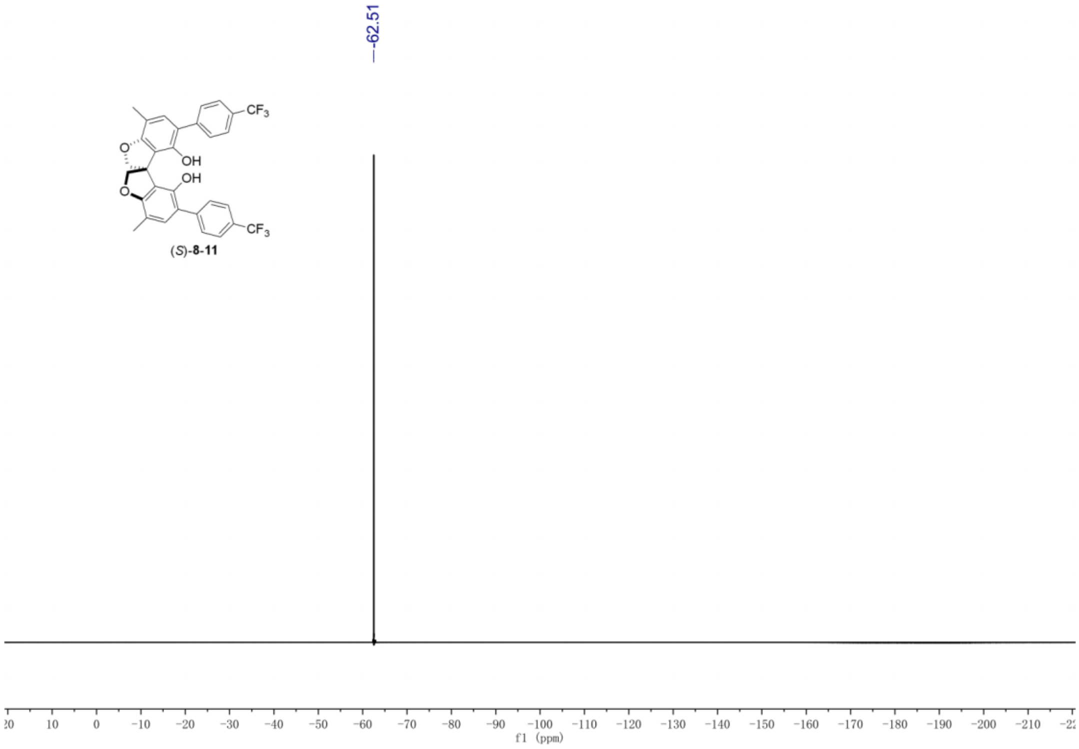

^{19}F NMR of (*S*)-**8-11** ($CDCl_3$)

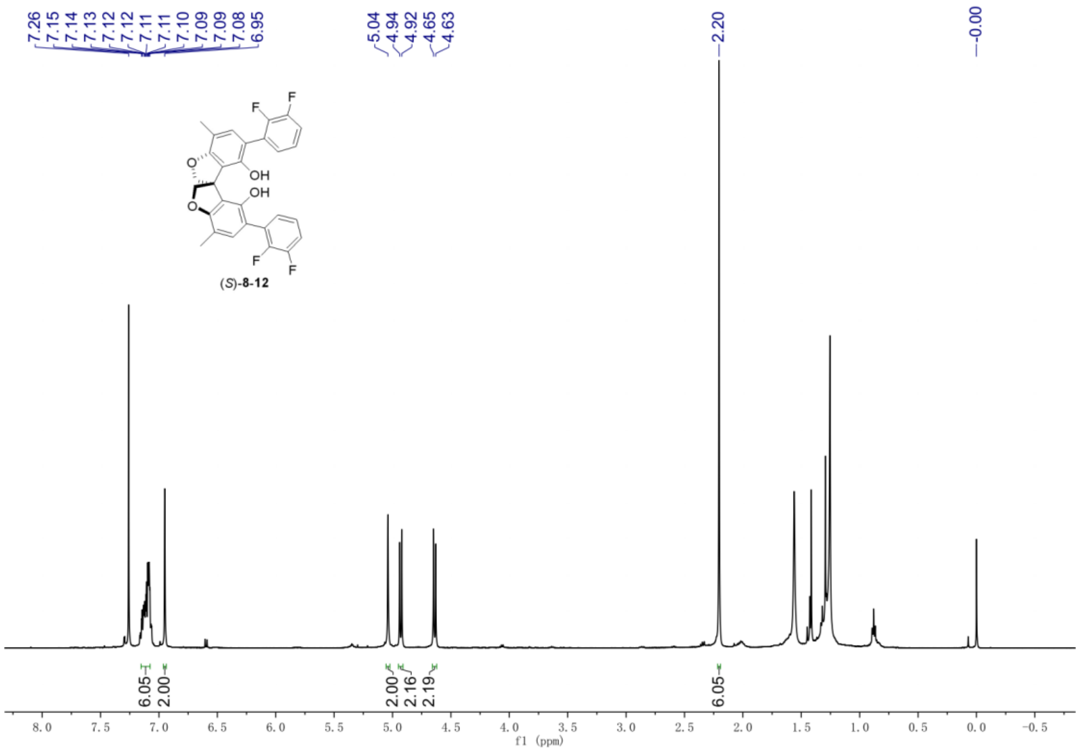

^{1}H NMR of (*S*)-**8-12** ($CDCl_3$)

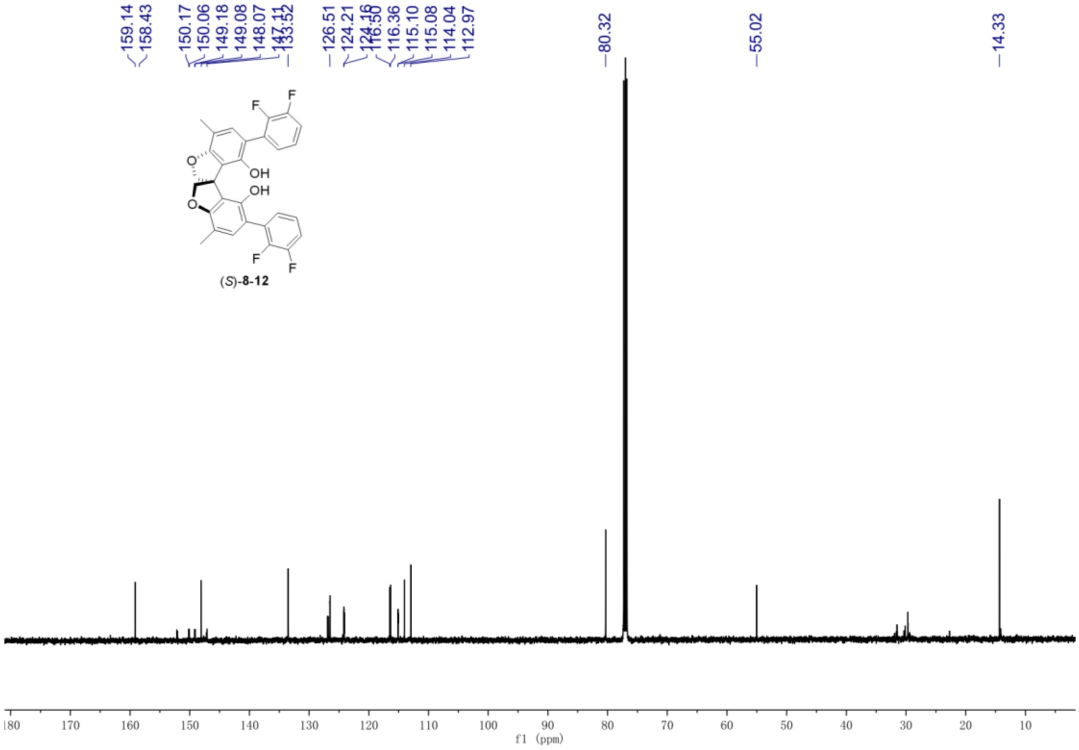

^{13}C NMR of (*S*)-**8-12** ($CDCl_3$)

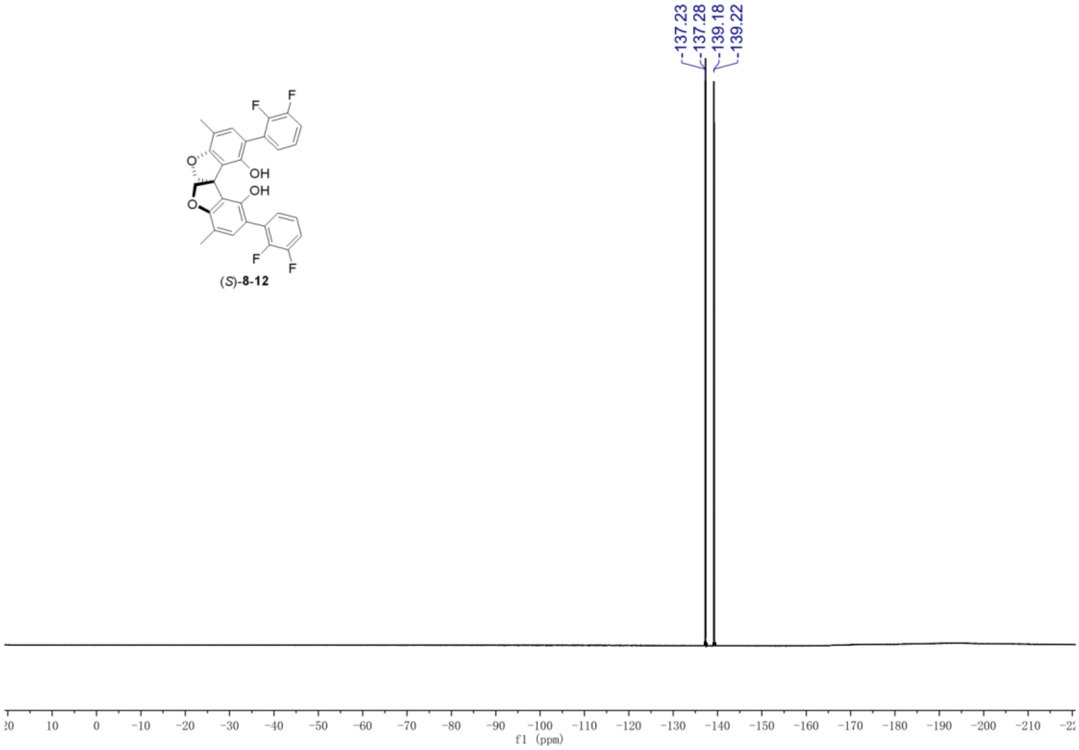

^{19}F NMR of (*S*)-**8-12** ($CDCl_3$)

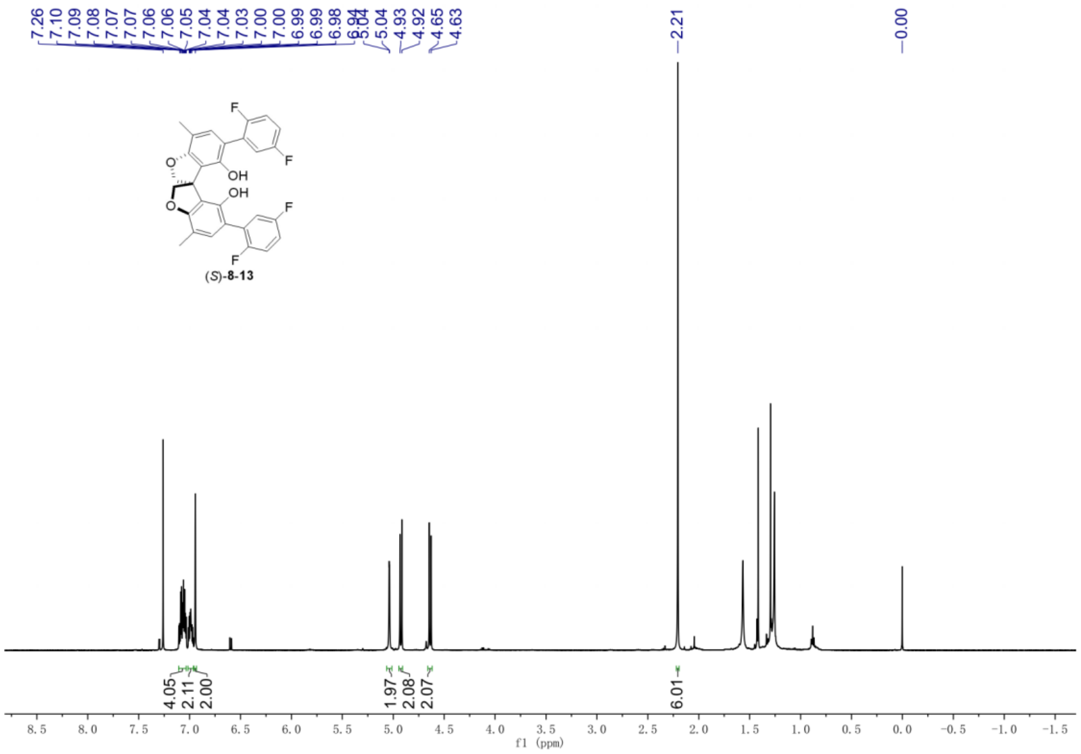

^{1}H NMR of (*S*)-**8-13** ($CDCl_3$)

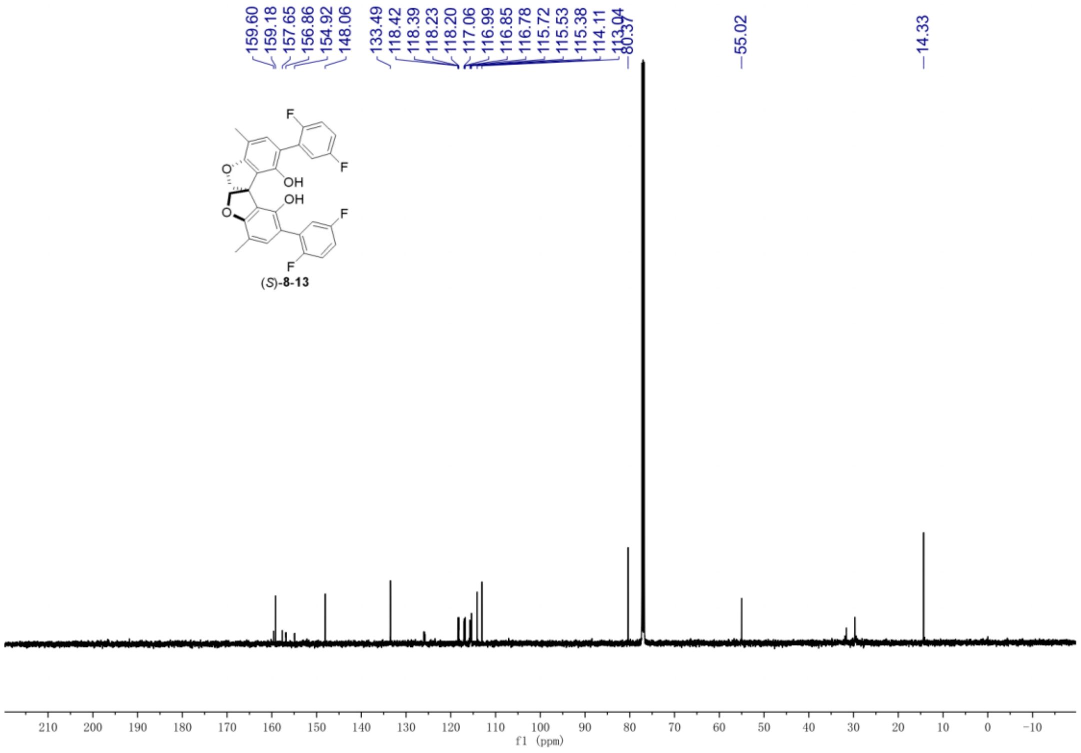

^{13}C NMR of (*S*)-**8-13** ($CDCl_3$)

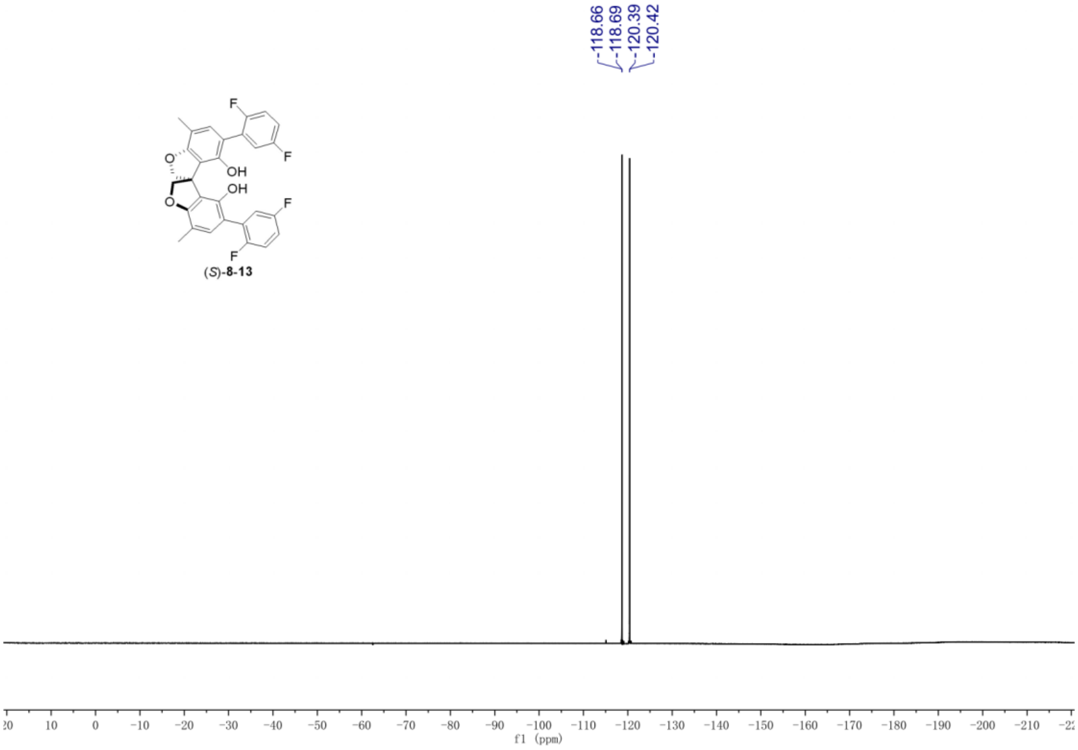

^{19}F NMR of (*S*)-**8-13** ($CDCl_3$)

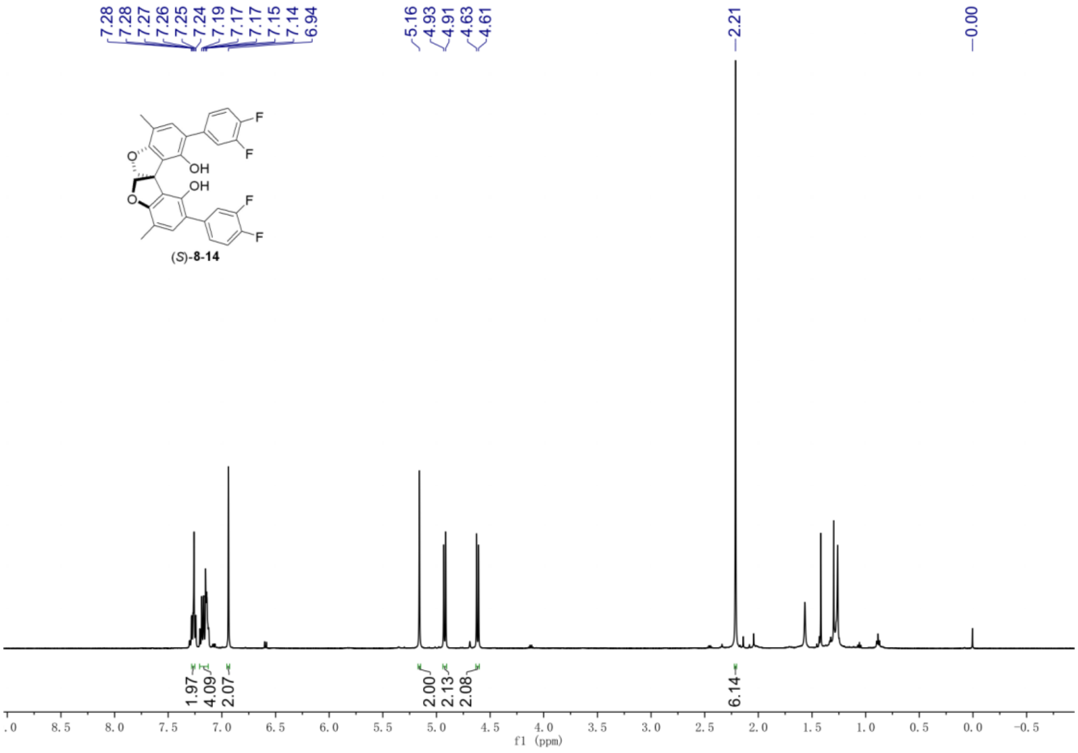

^{1}H NMR of (*S*)-**8-14** ($CDCl_3$)

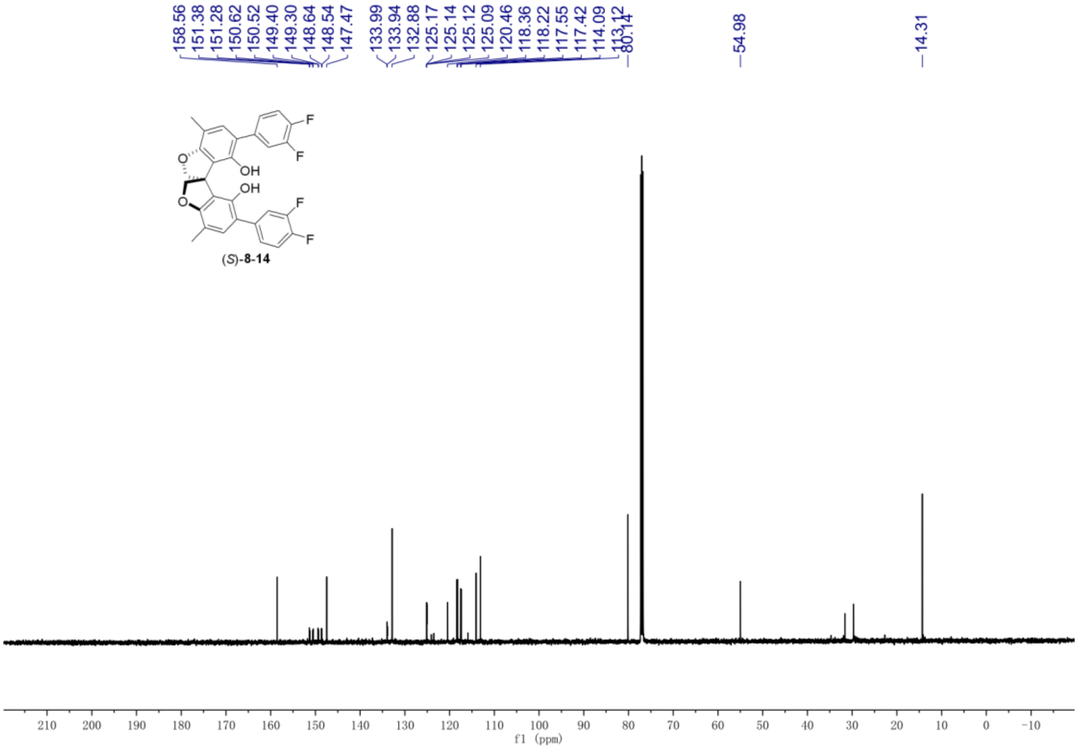

^{13}C NMR of (*S*)-**8-14** ($CDCl_3$)

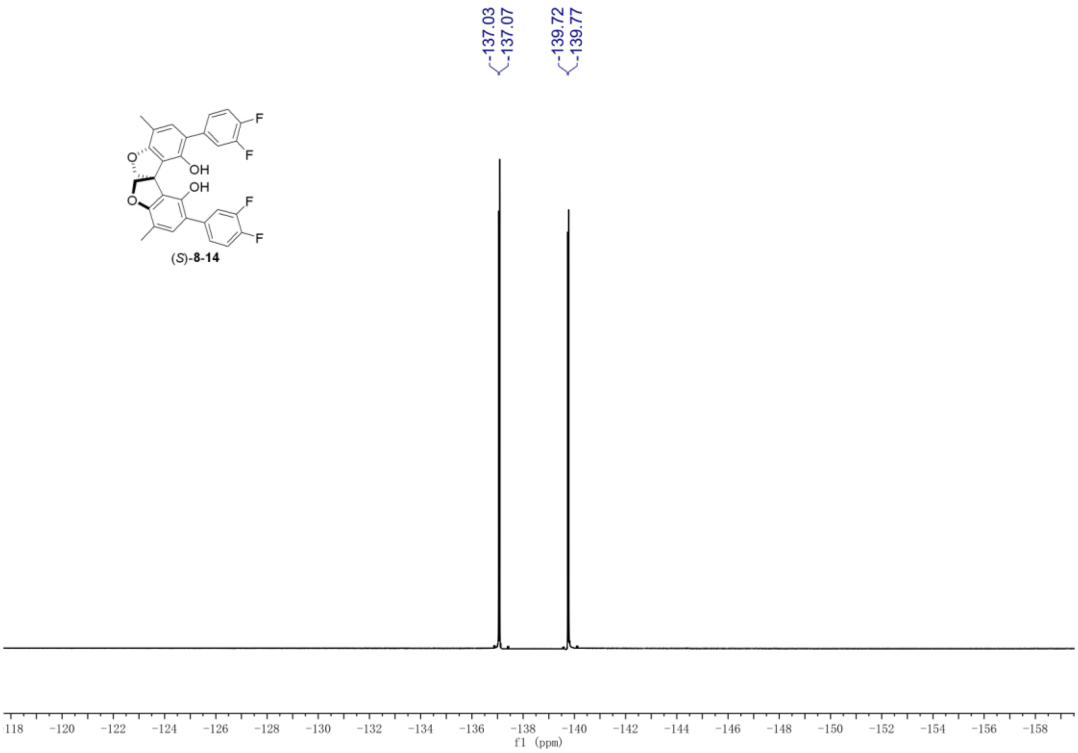

^{19}F NMR of (*S*)-**8-14** ($CDCl_3$)

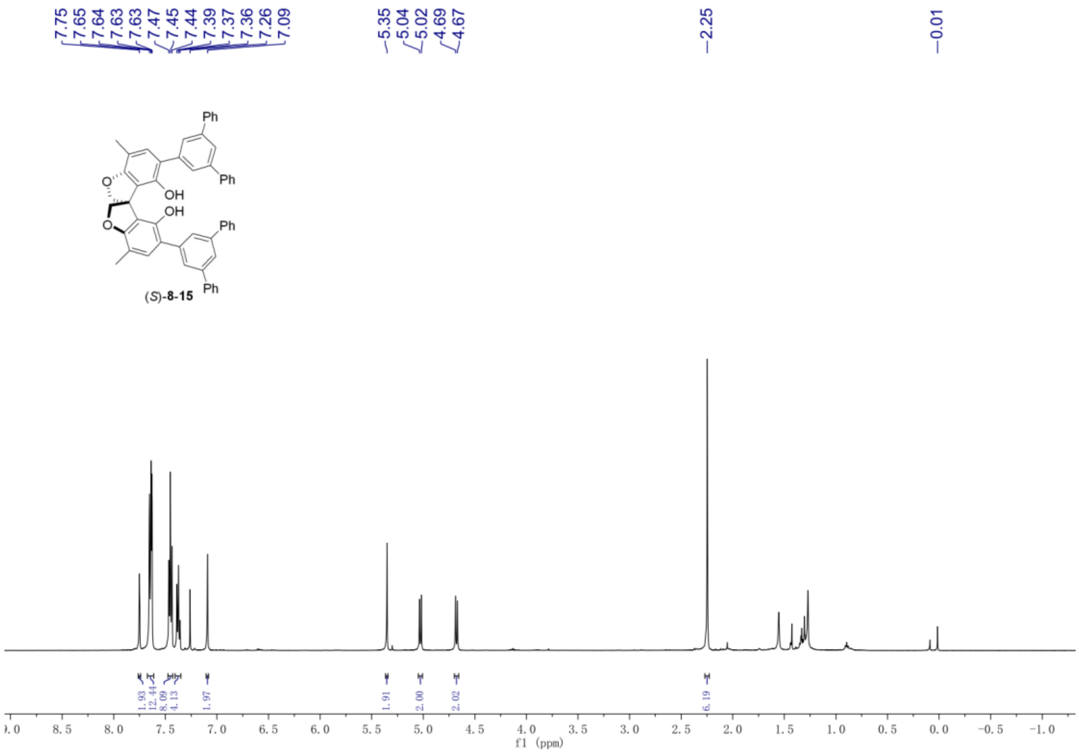

^{1}H NMR of (*S*)-**8-15** ($CDCl_3$)

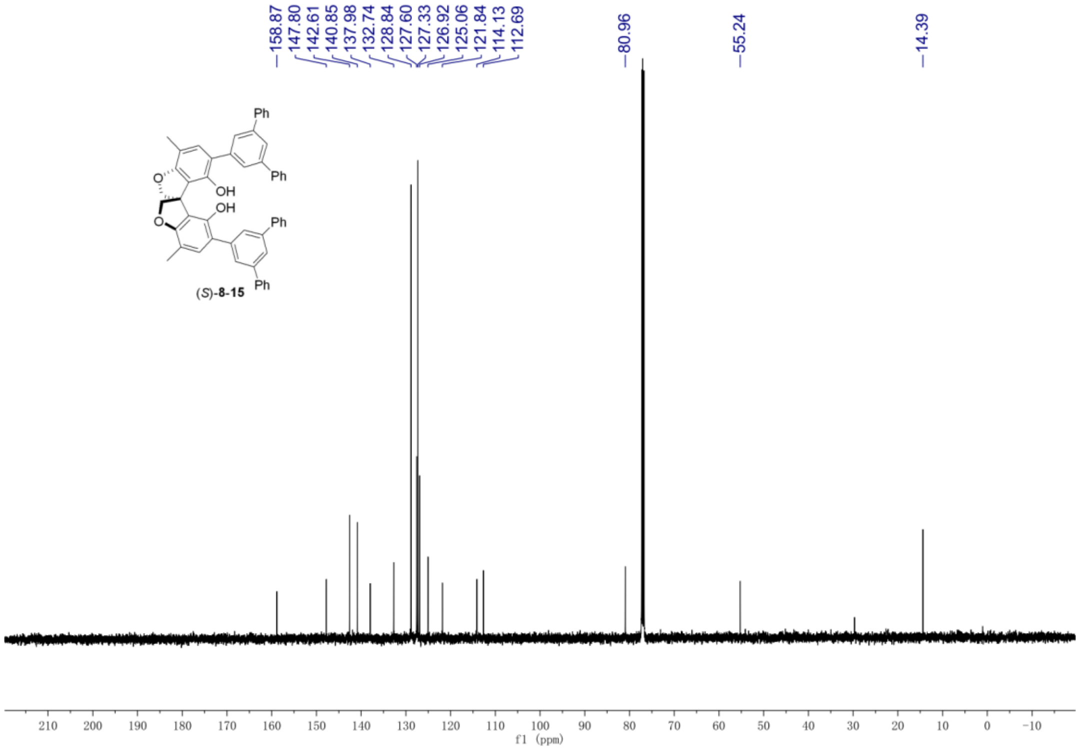

^{13}C NMR of (*S*)-**8-15** ($CDCl_3$)

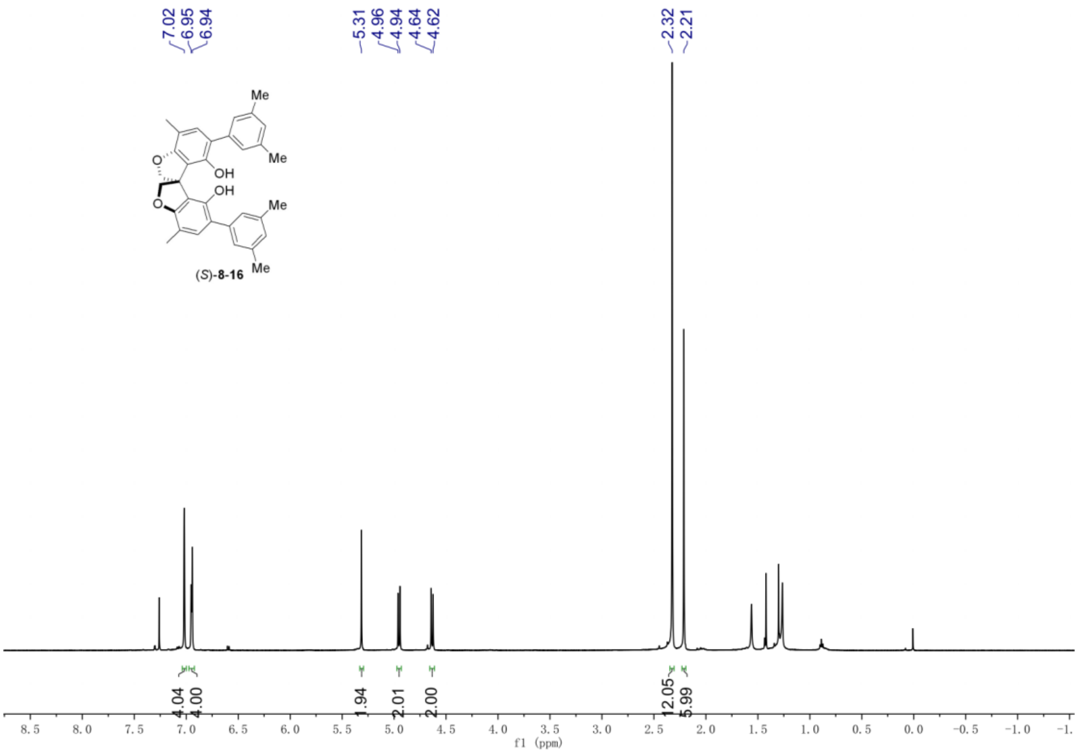

^{1}H NMR of (*S*)-**8-16** ($CDCl_3$)

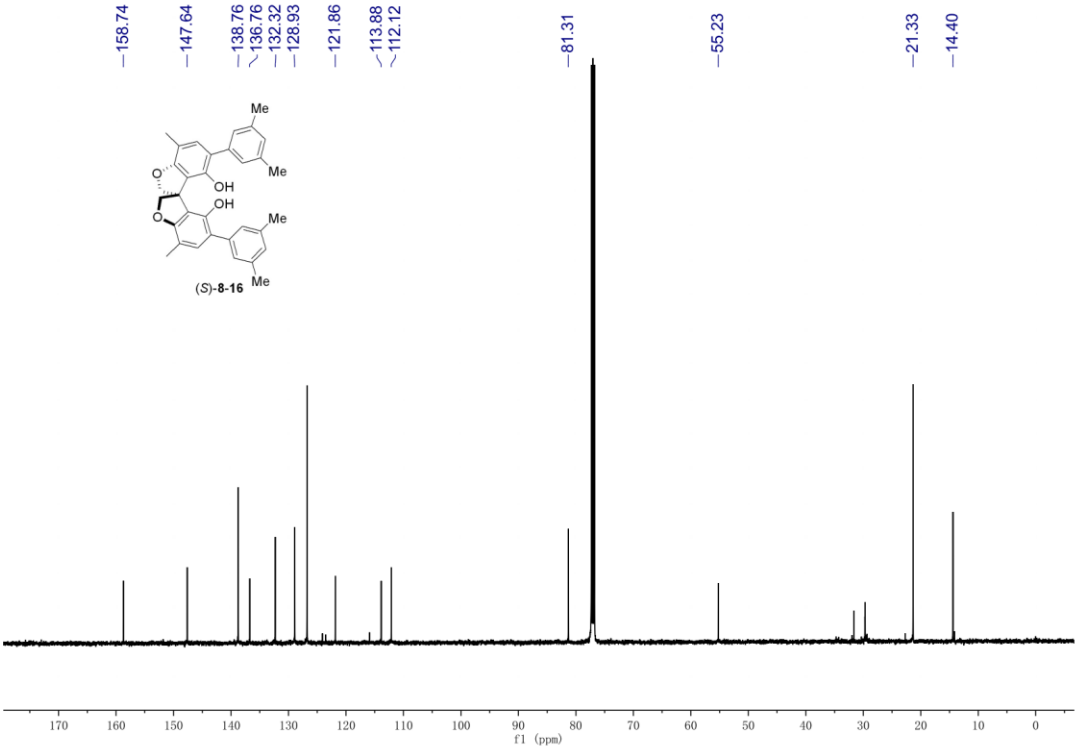

^{13}C NMR of (*S*)-**8-16** ($CDCl_3$)

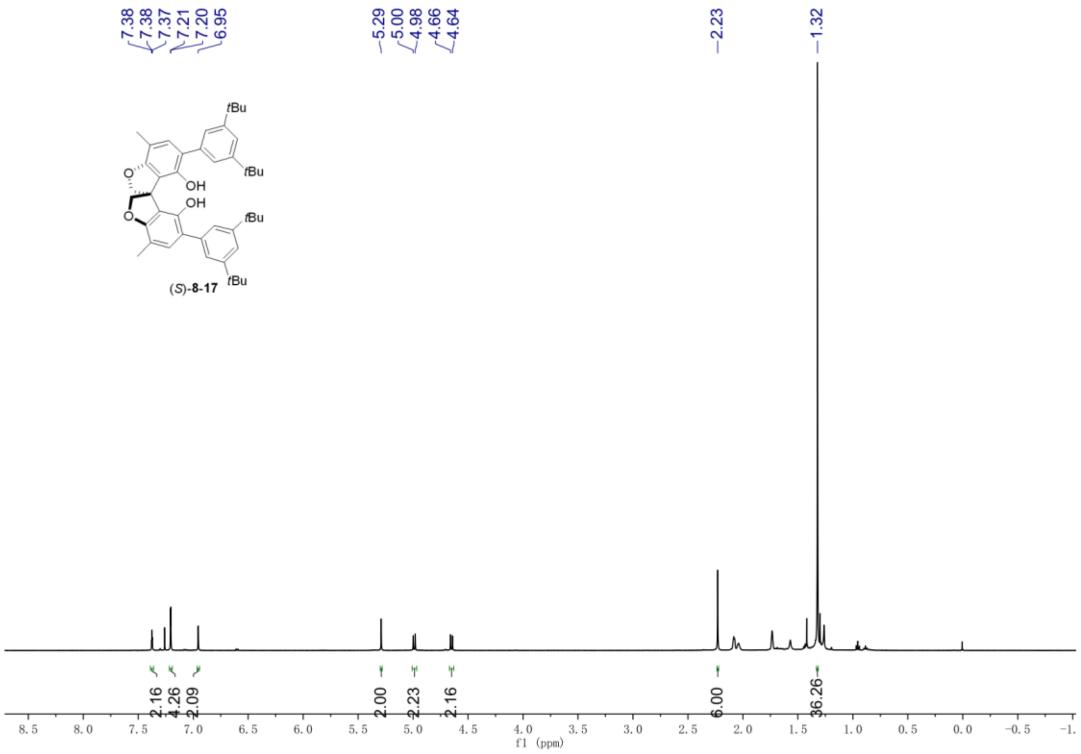

^{1}H NMR of (*S*)-**8-17** ($CDCl_3$)

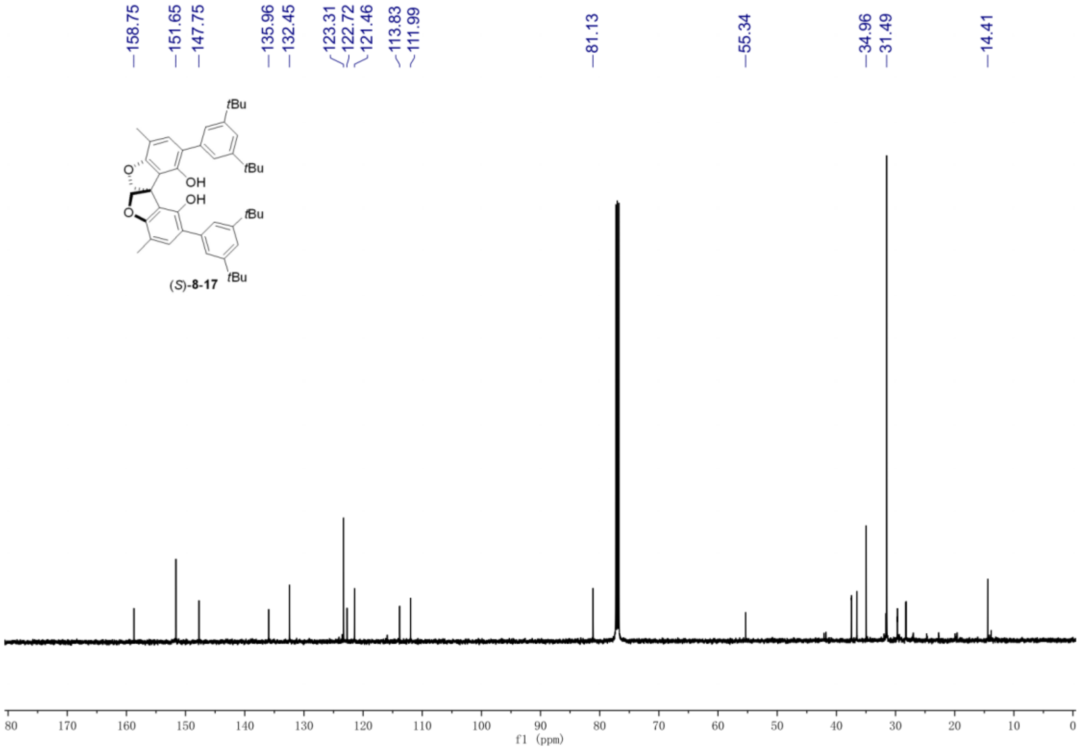

^{13}C NMR of (*S*)-**8-17** ($CDCl_3$)

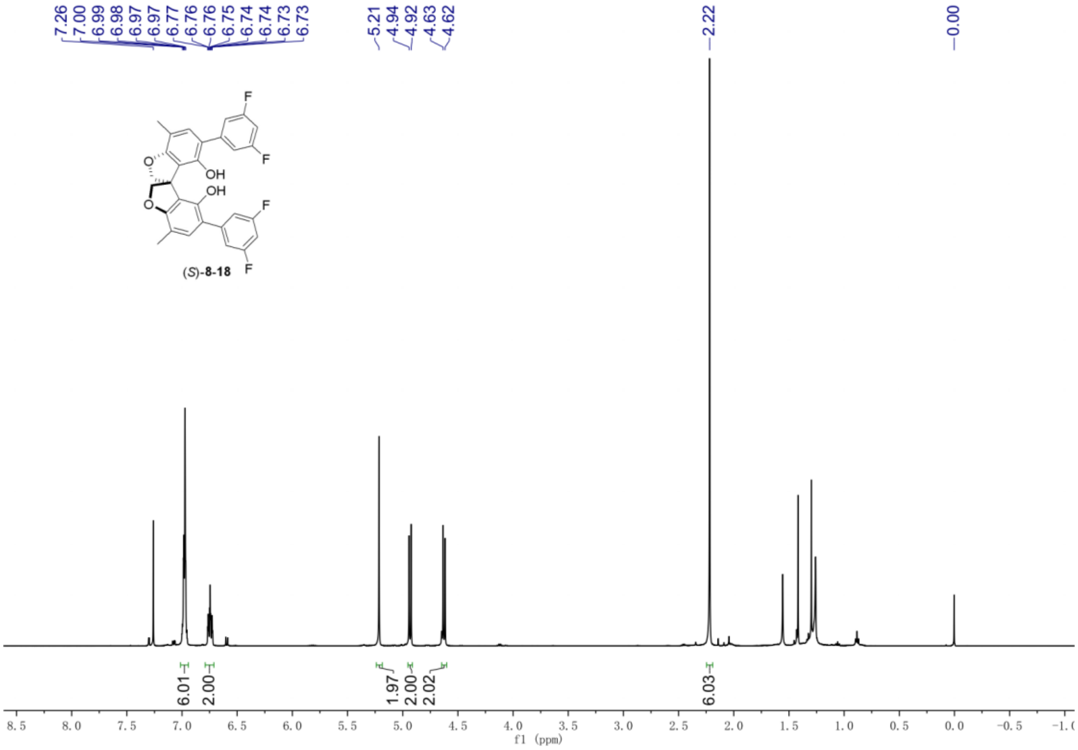

^{1}H NMR of (*S*)-**8-18** ($CDCl_3$)

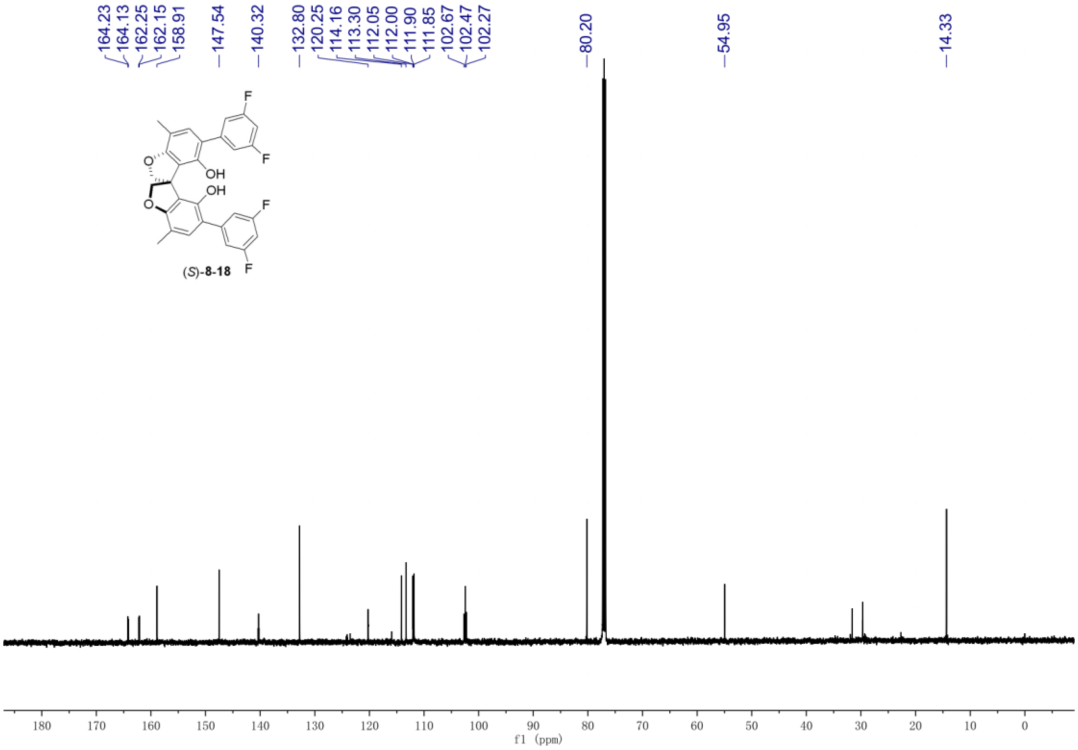

^{13}C NMR of (*S*)-**8-18** ($CDCl_3$)

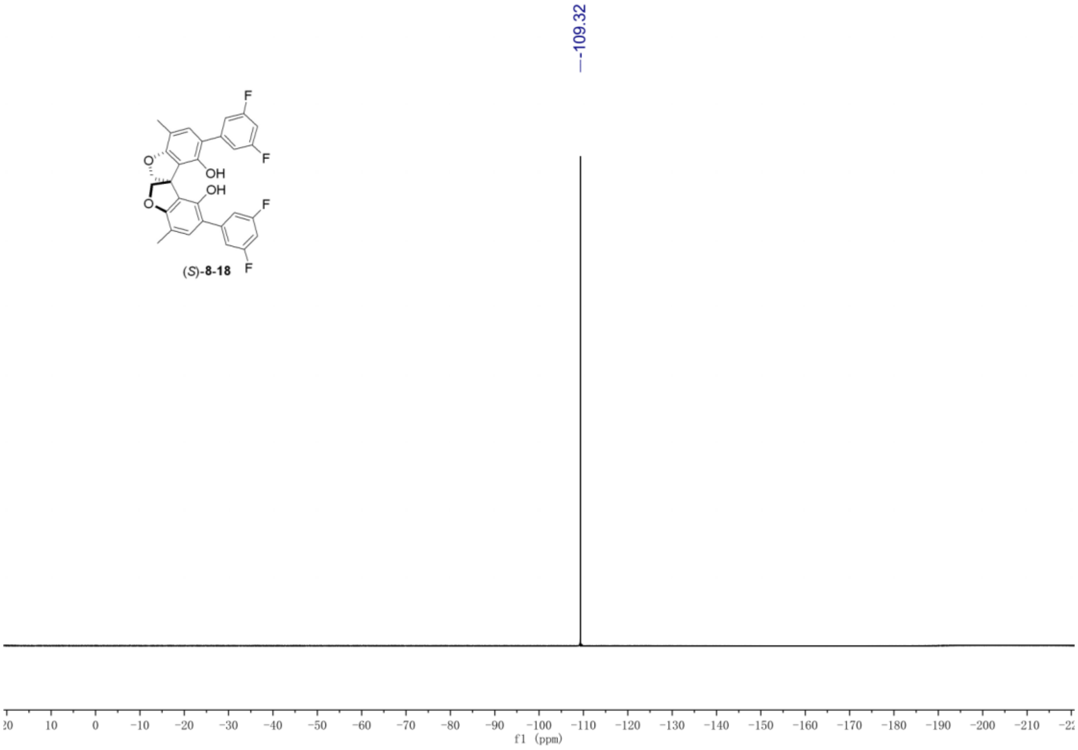

^{19}F NMR of (*S*)-**8-18** ($CDCl_3$)

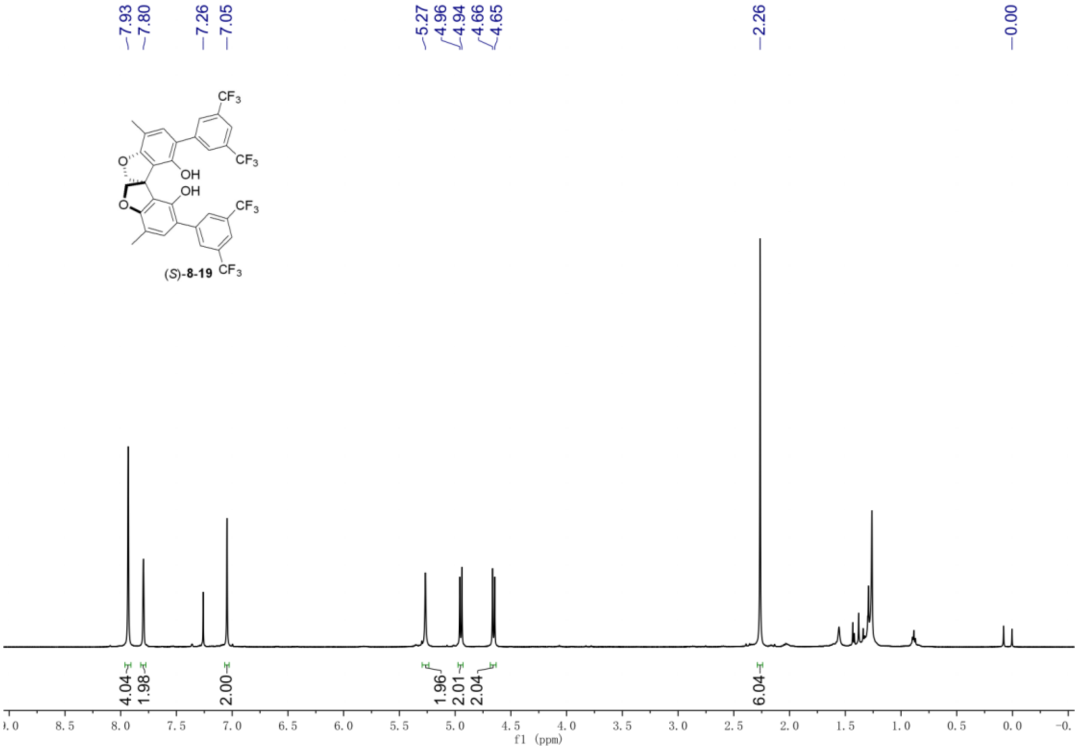

^{1}H NMR of (*S*)-**8-19** ($CDCl_3$)

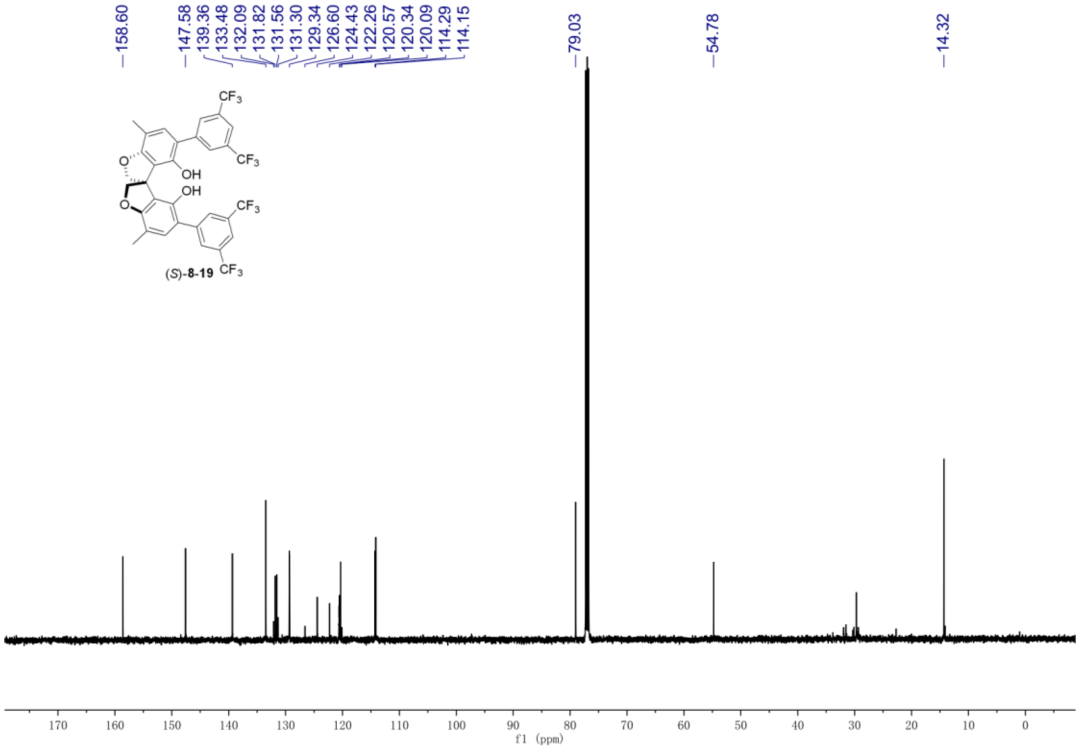

^{13}C NMR of (*S*)-**8-19** ($CDCl_3$)

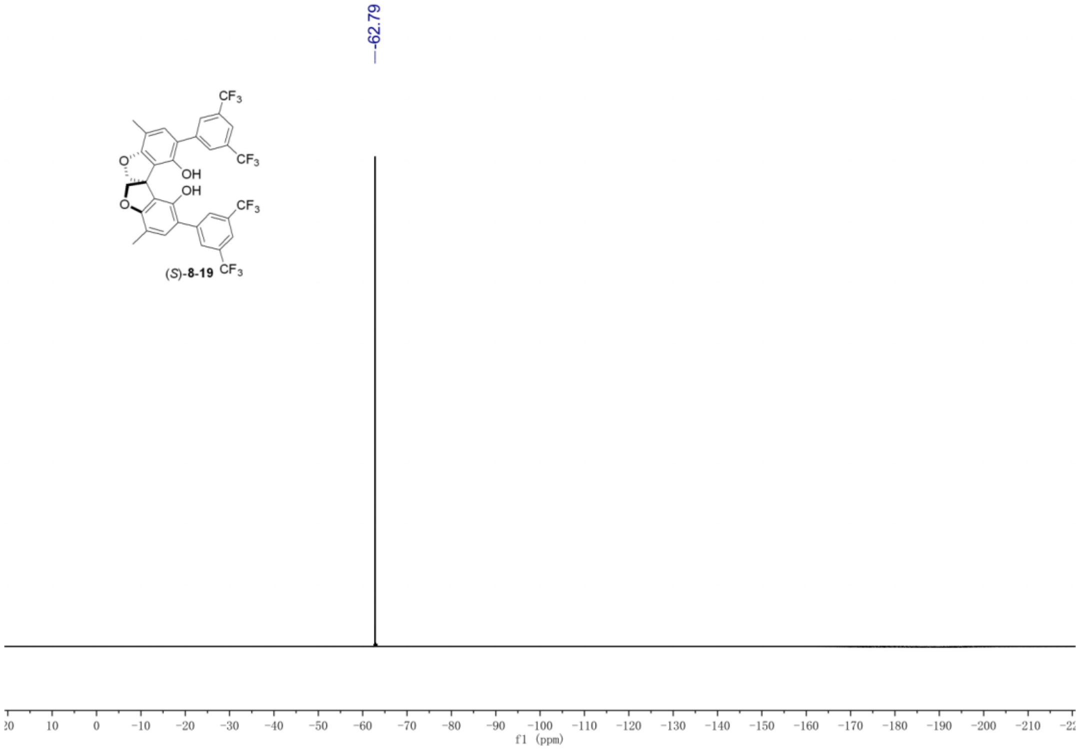

^{19}F NMR of (*S*)-**8-19** ($CDCl_3$)

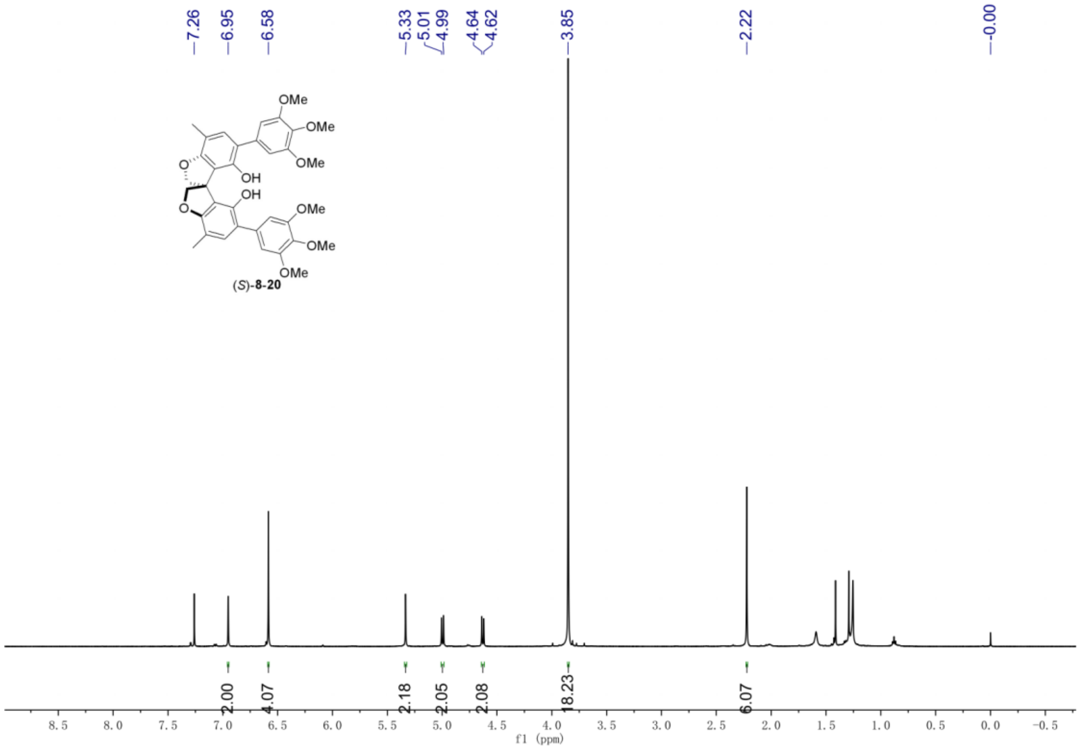

^{1}H NMR of (*S*)-**8-20** ($CDCl_3$)

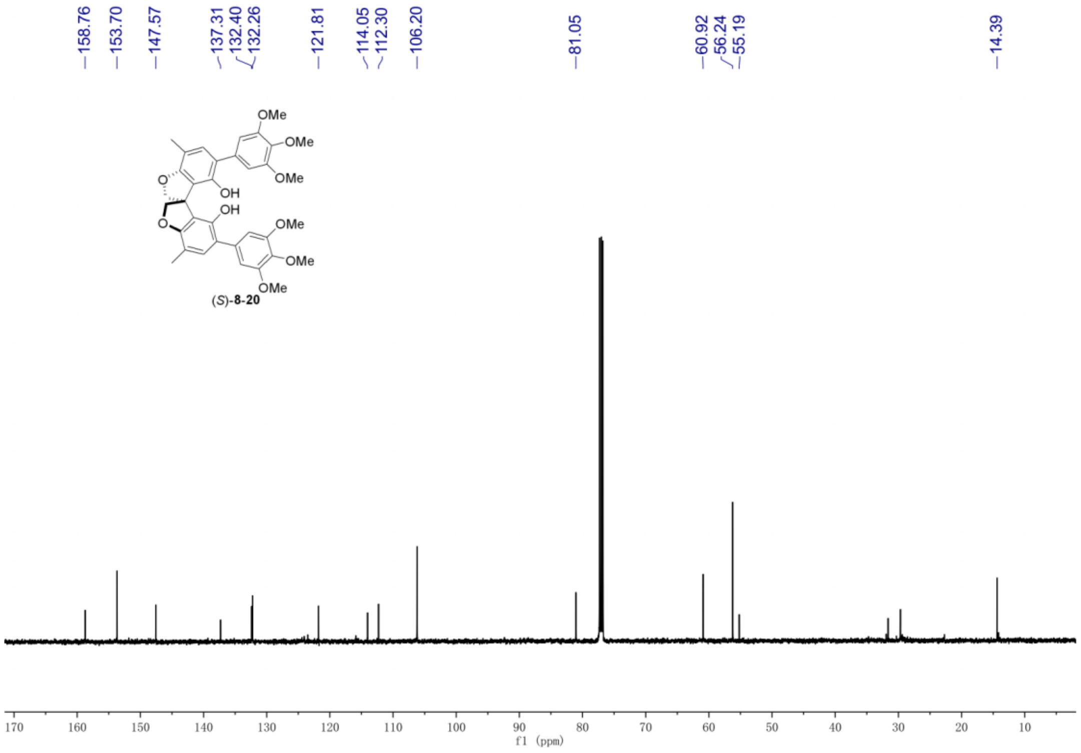

^{13}C NMR of (*S*)-**8-20** ($CDCl_3$)

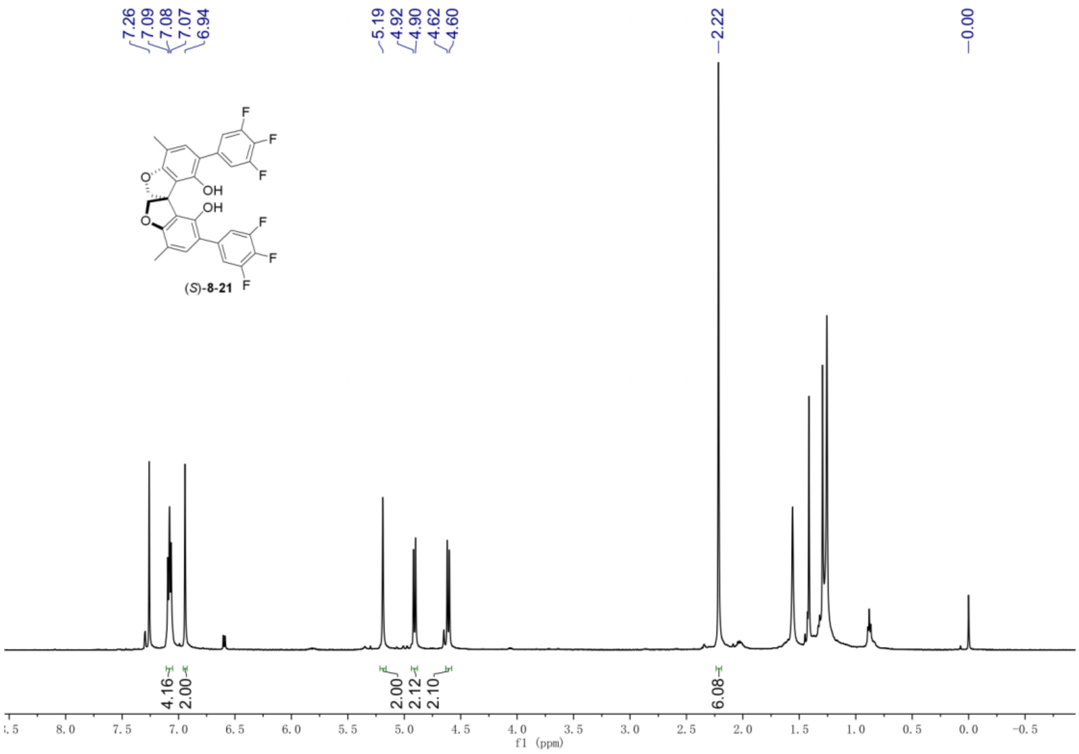

^{1}H NMR of (*S*)-**8-21** ($CDCl_3$)

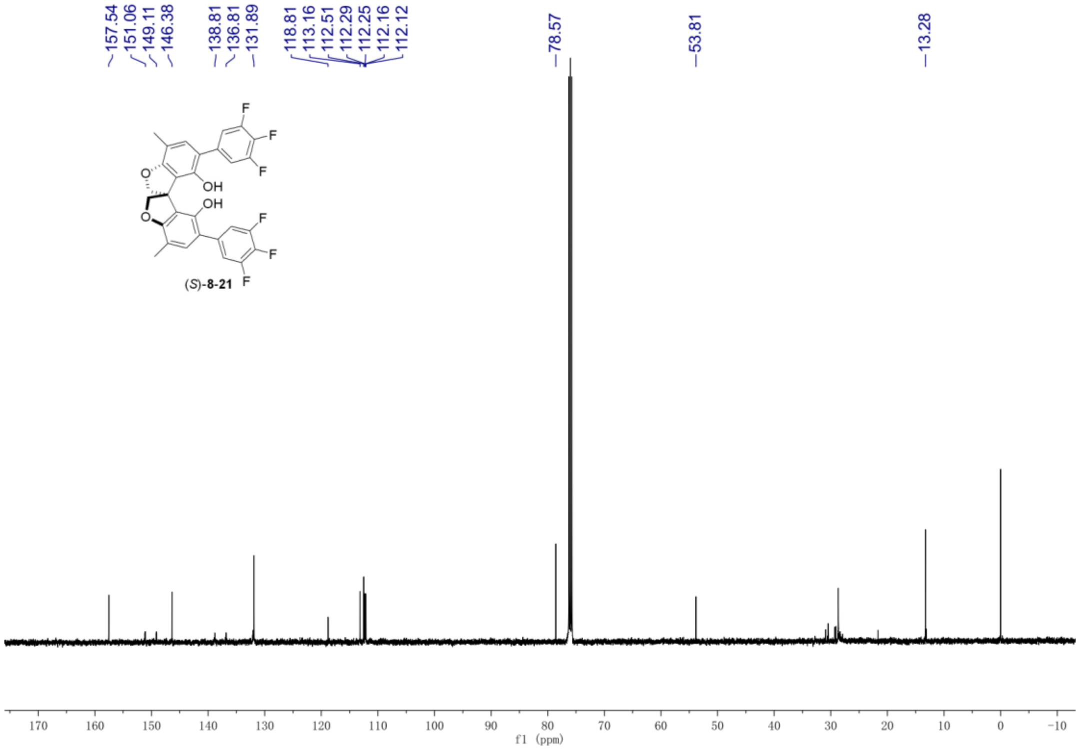

^{13}C NMR of (*S*)-**8-21** ($CDCl_3$)

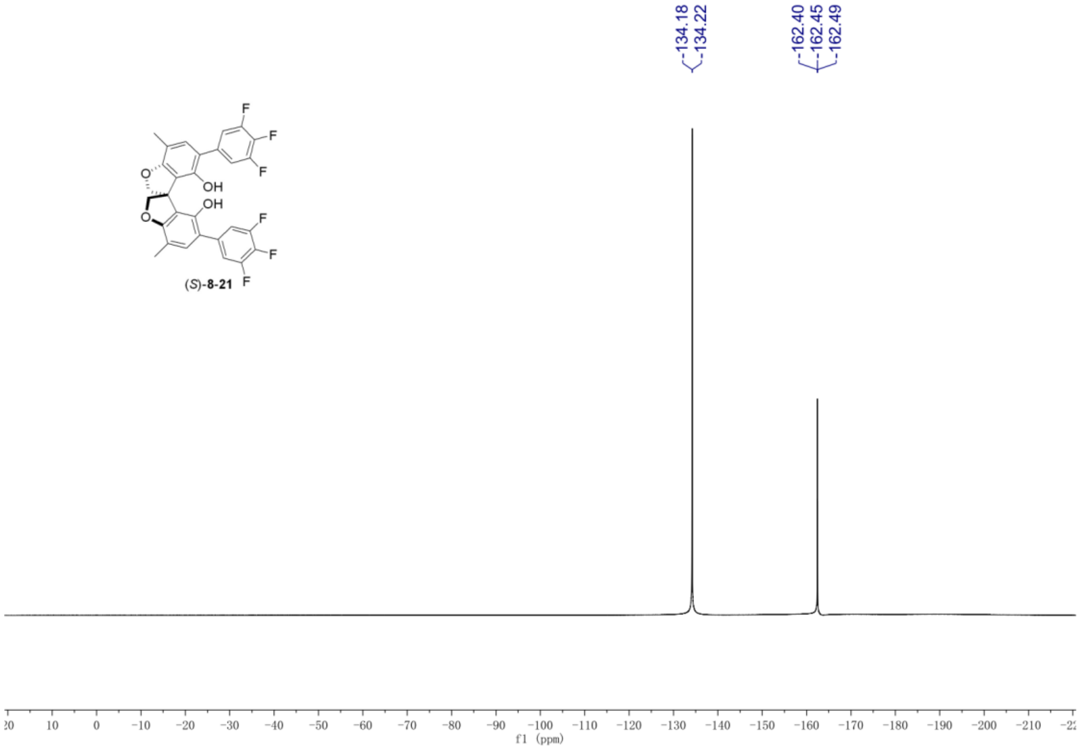

^{19}F NMR of (*S*)-**8-21** ($CDCl_3$)

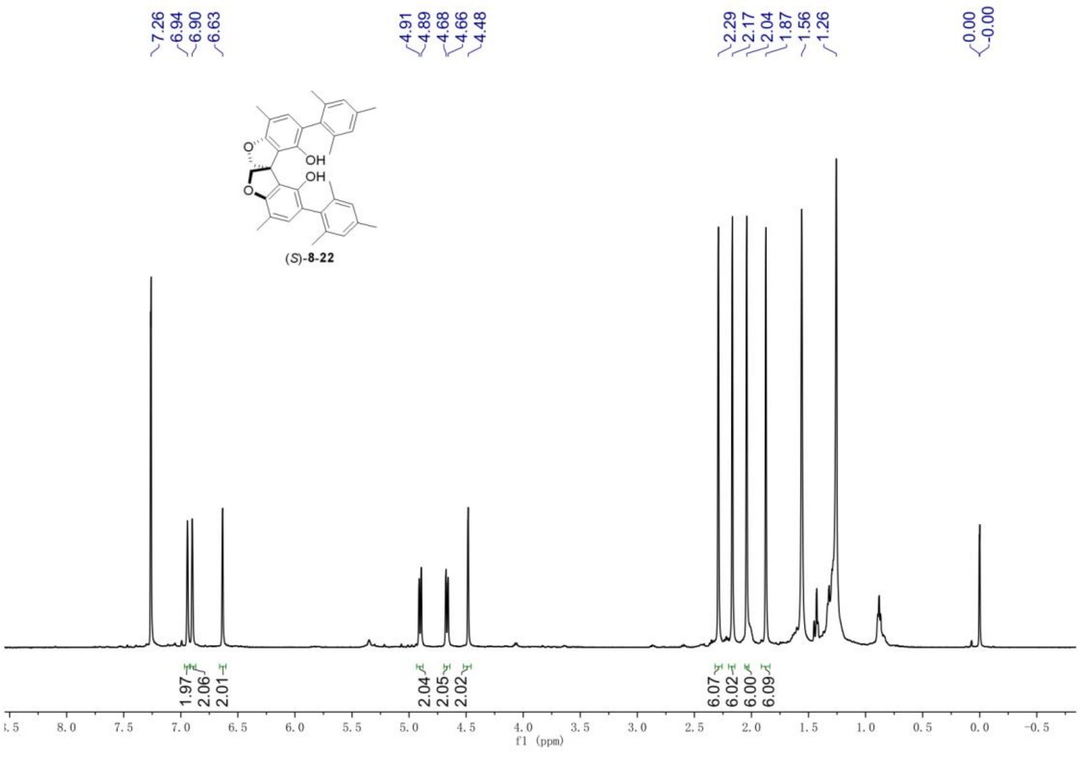

^{1}H NMR of (*S*)-**8-22** ($CDCl_3$)

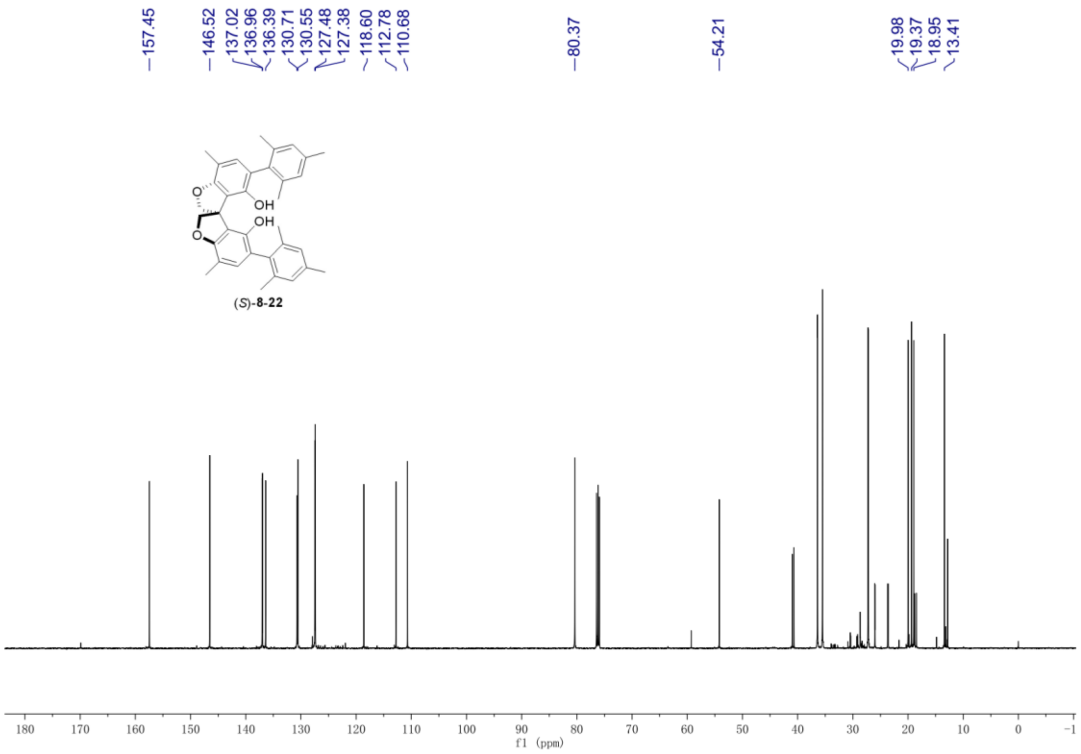

^{13}C NMR of (*S*)-**8-22** ($CDCl_3$)

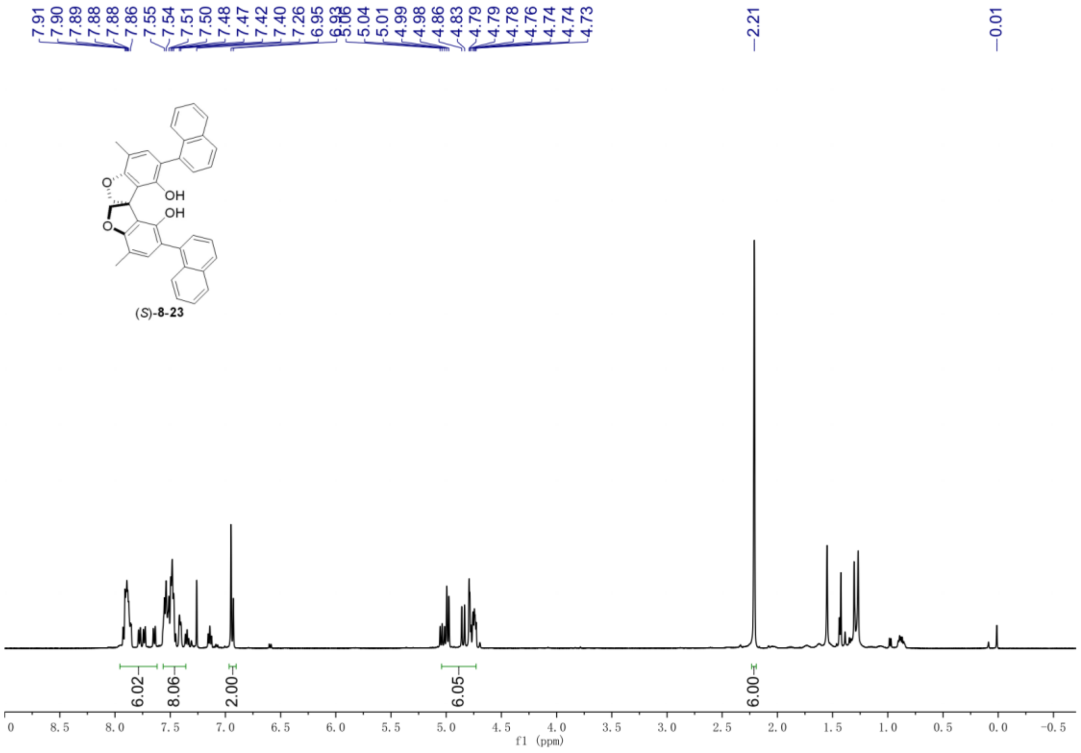

^{1}H NMR of (*S*)-**8-23** ($CDCl_3$)

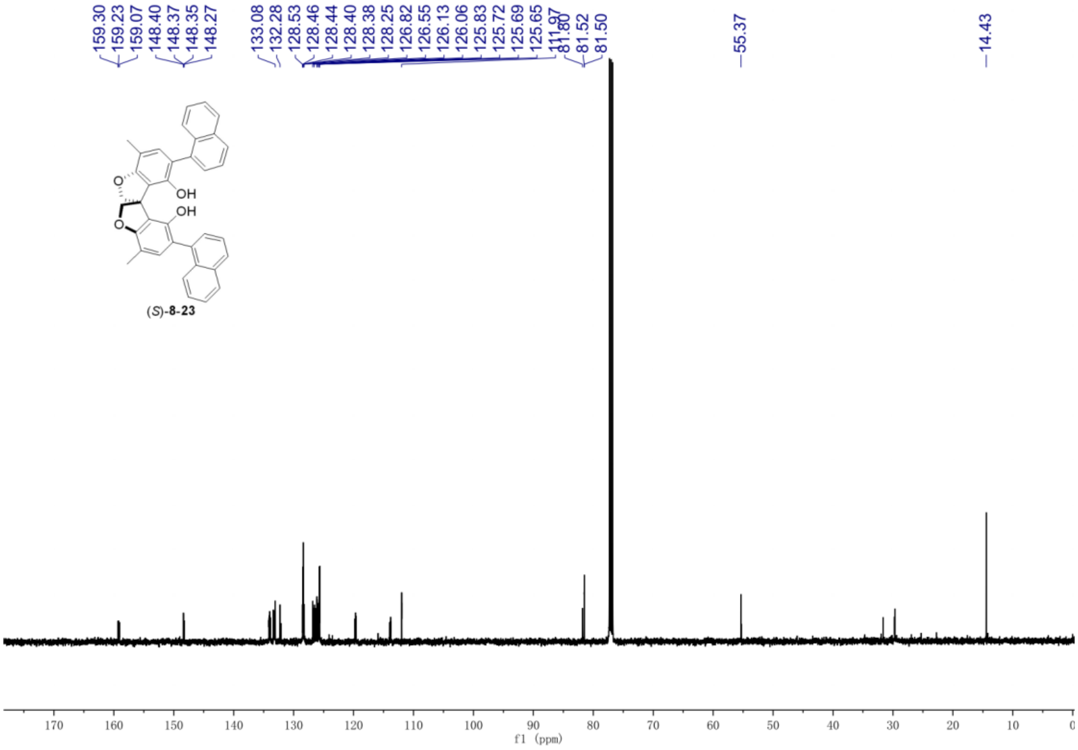

^{13}C NMR of (*S*)-**8-23** (CDCl$_3$)

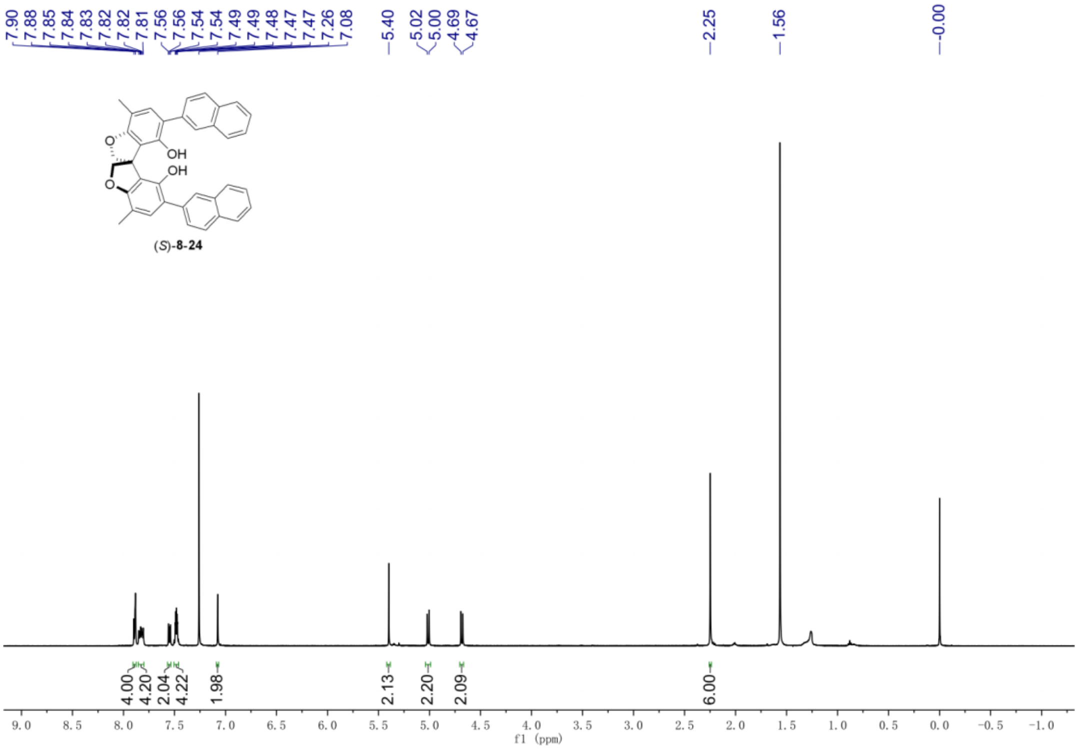

^{1}H NMR of (*S*)-**8-24** (CDCl$_3$)

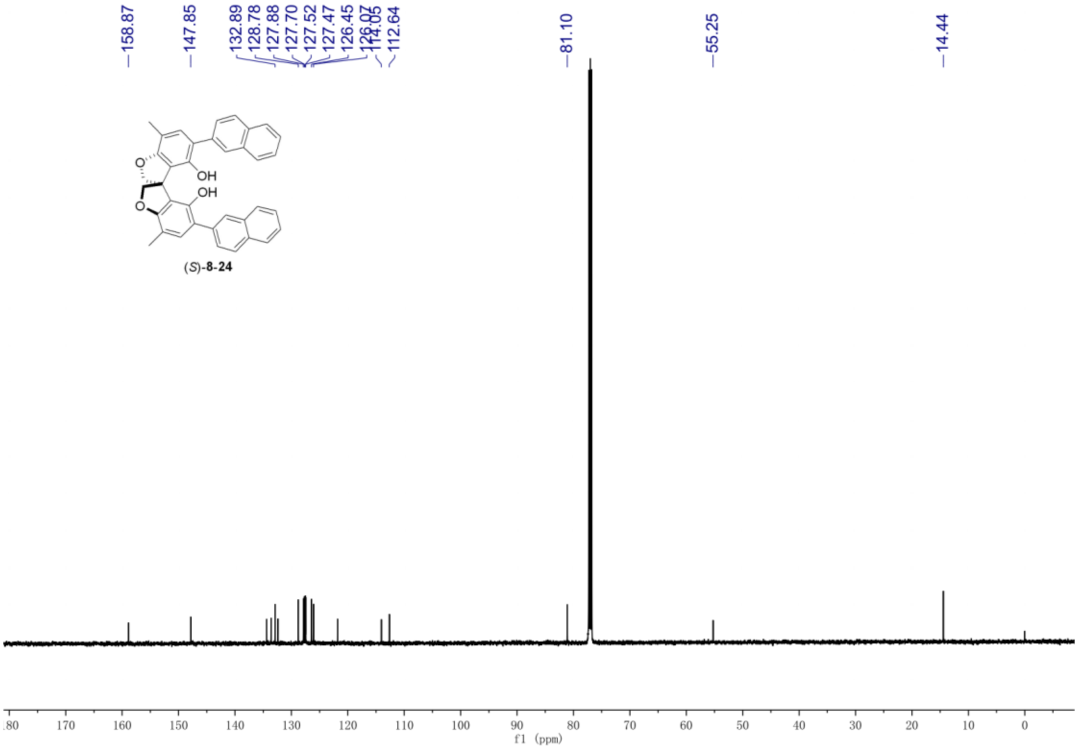

^{13}C NMR of (*S*)-**8-24** ($CDCl_3$)

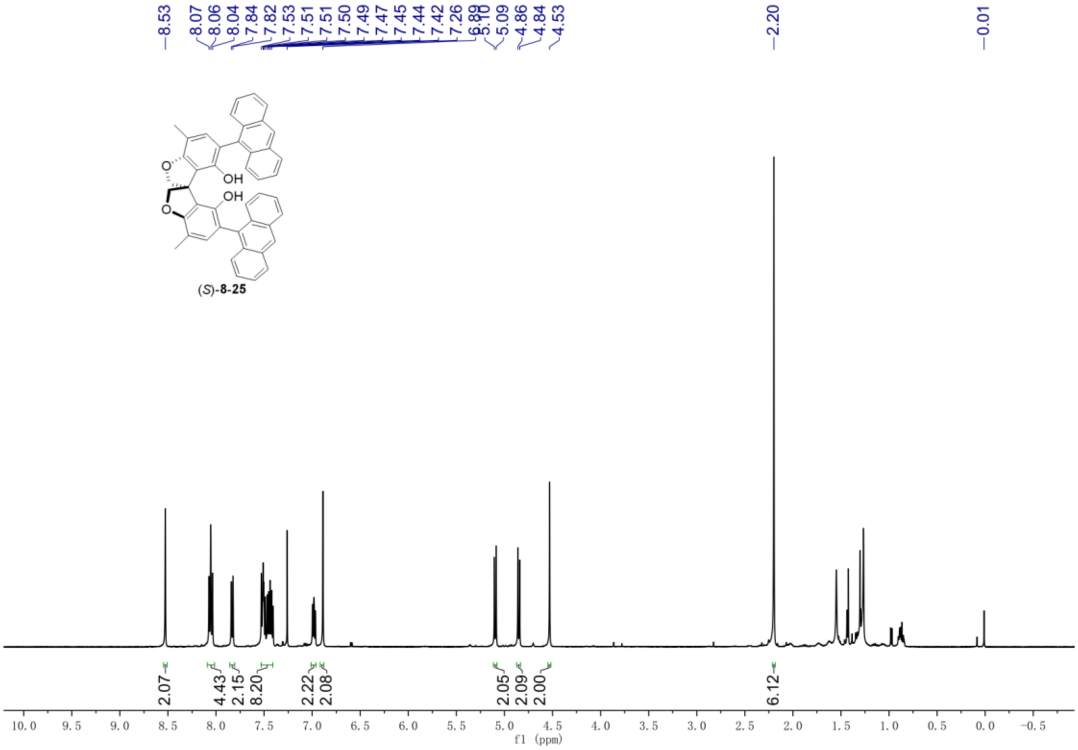

^{1}H NMR of (*S*)-**8-25** ($CDCl_3$)

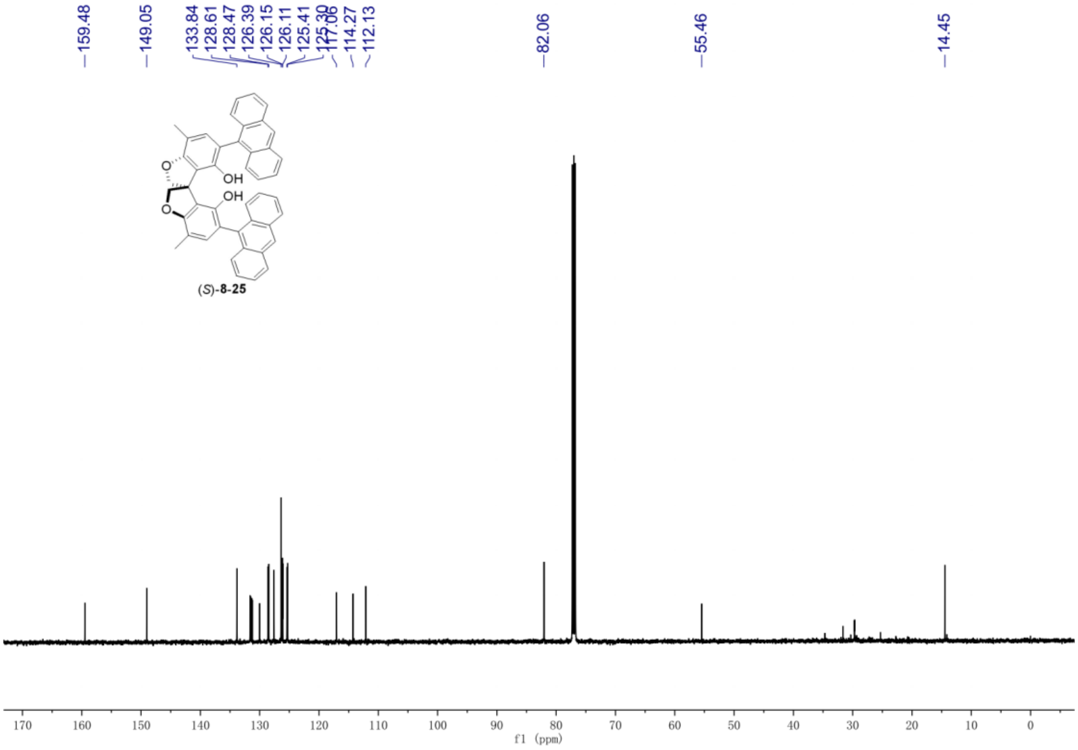

^{13}C NMR of (*S*)-**8-25** ($CDCl_3$)

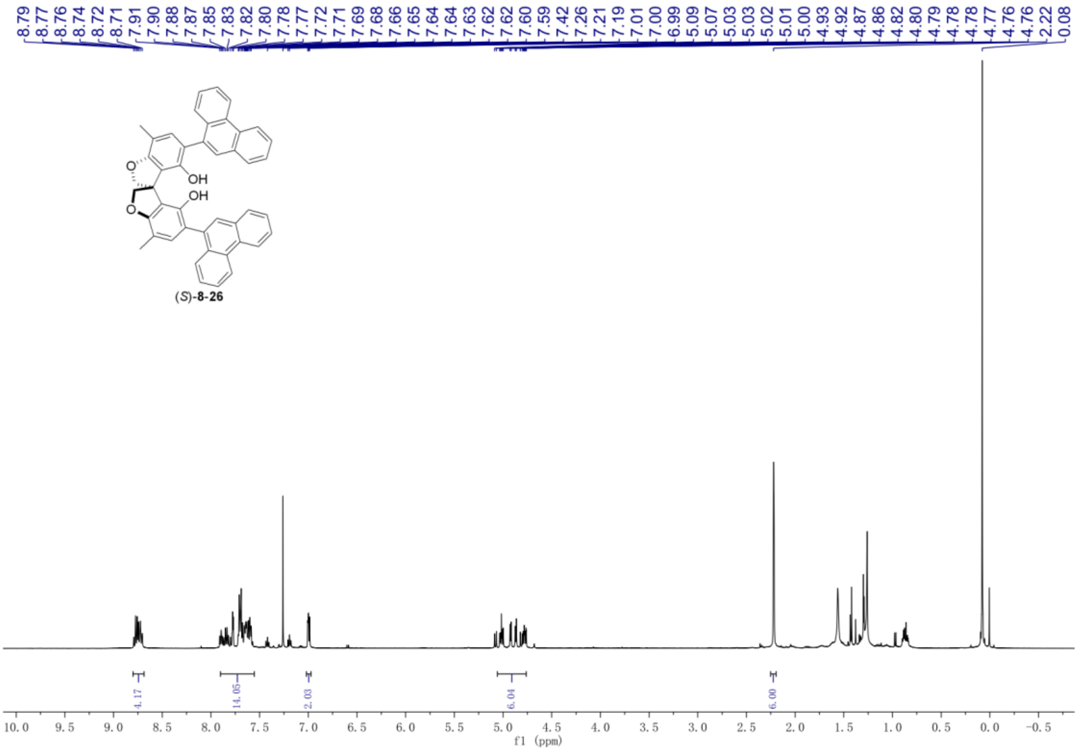

^{1}H NMR of (*S*)-**8-26** ($CDCl_3$)

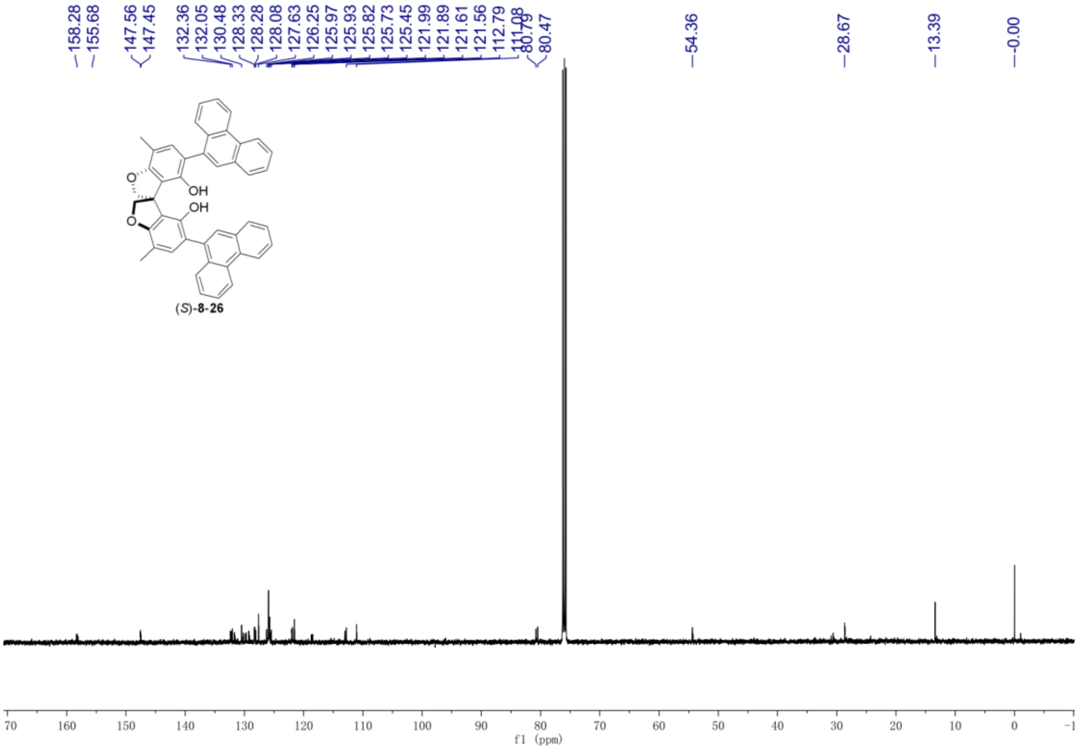

^{13}C NMR of (*S*)-**8-26** (CDCl$_3$)

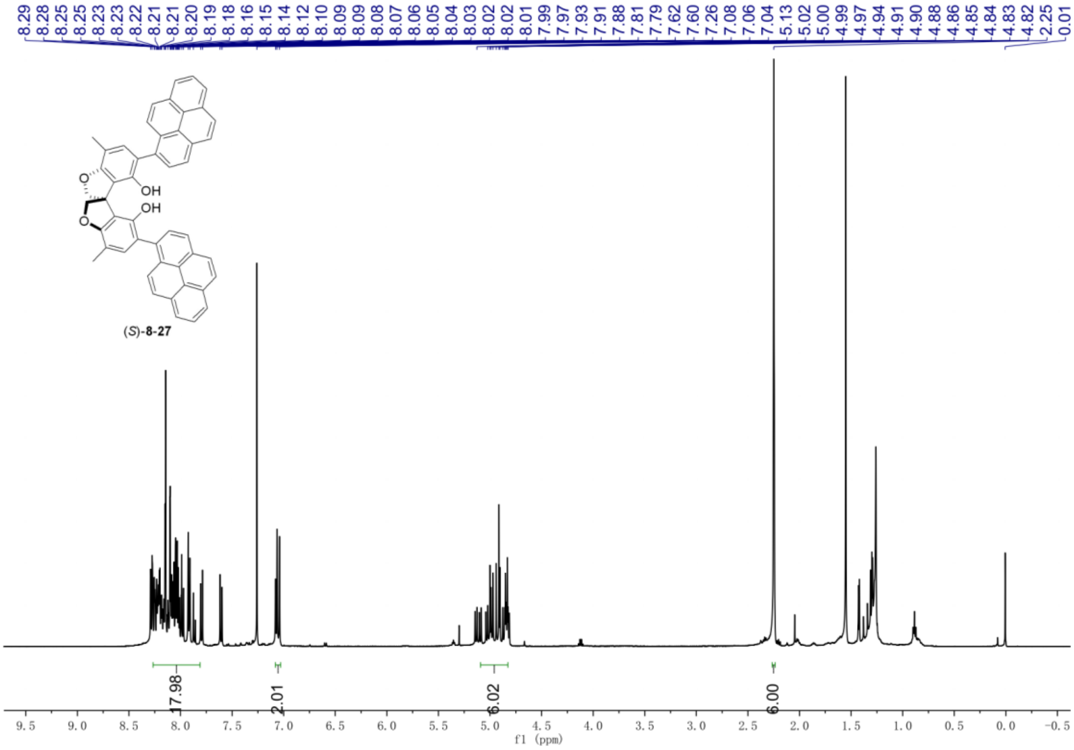

^{1}H NMR of (*S*)-**8-27** (CDCl$_3$)

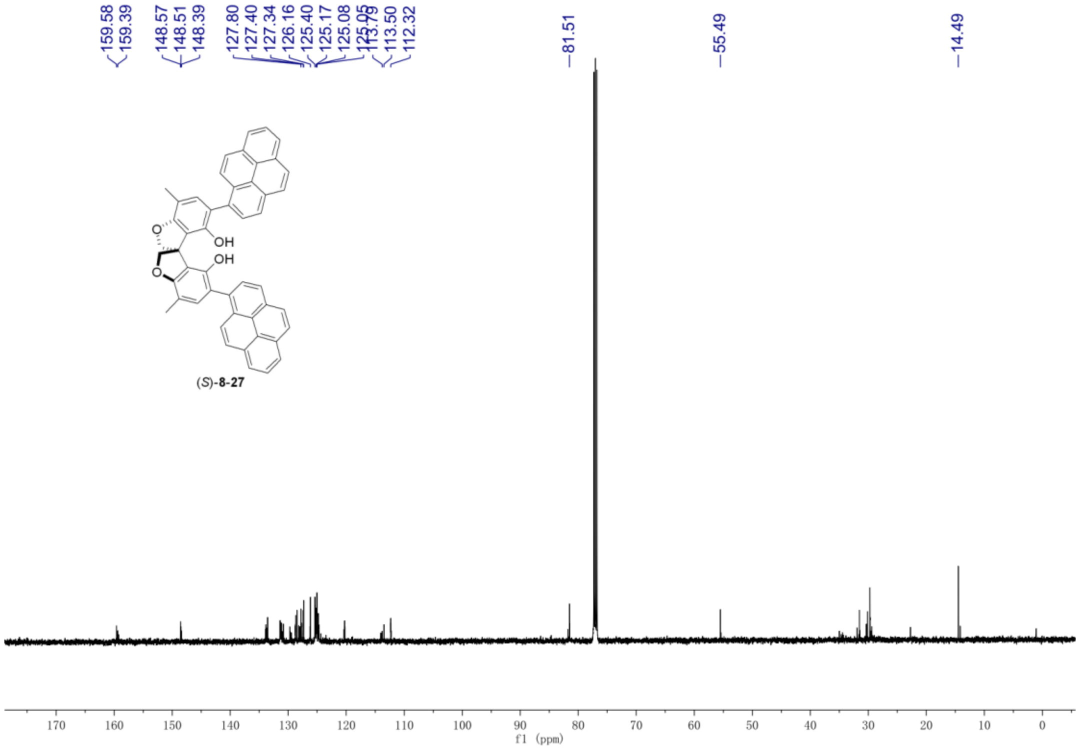

^{13}C NMR of (*S*)-**8-27** ($CDCl_3$)

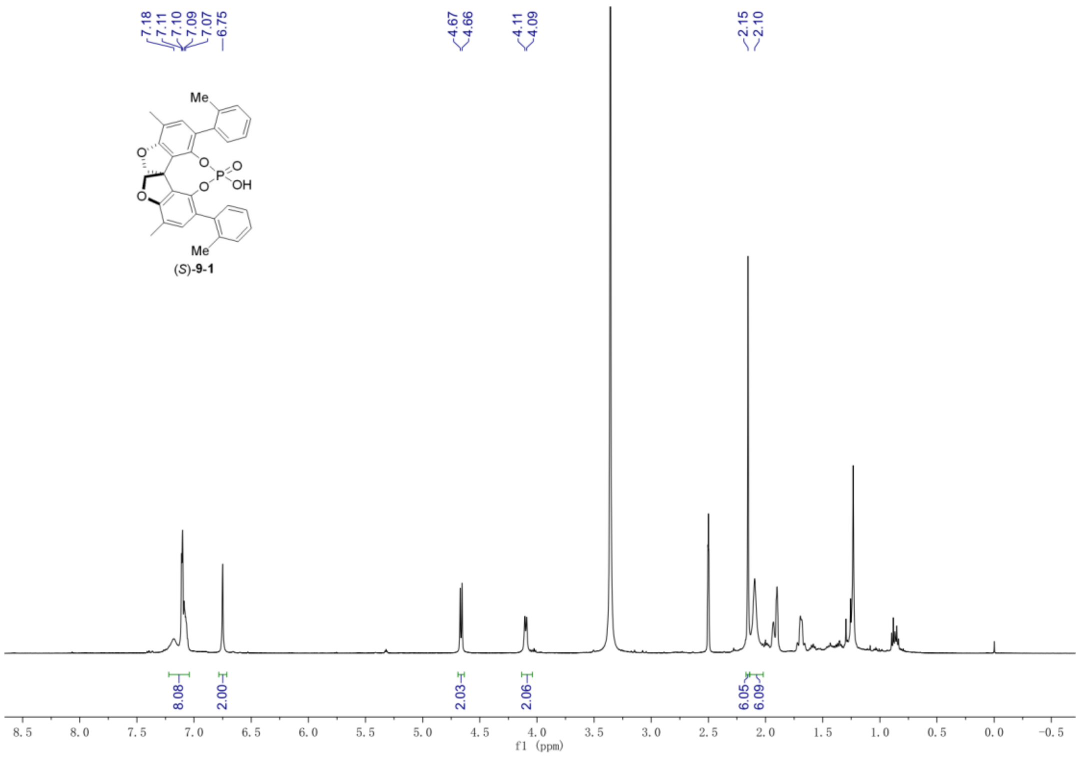

^{1}H NMR of (*S*)-**9-1** (DMSO-d_6)

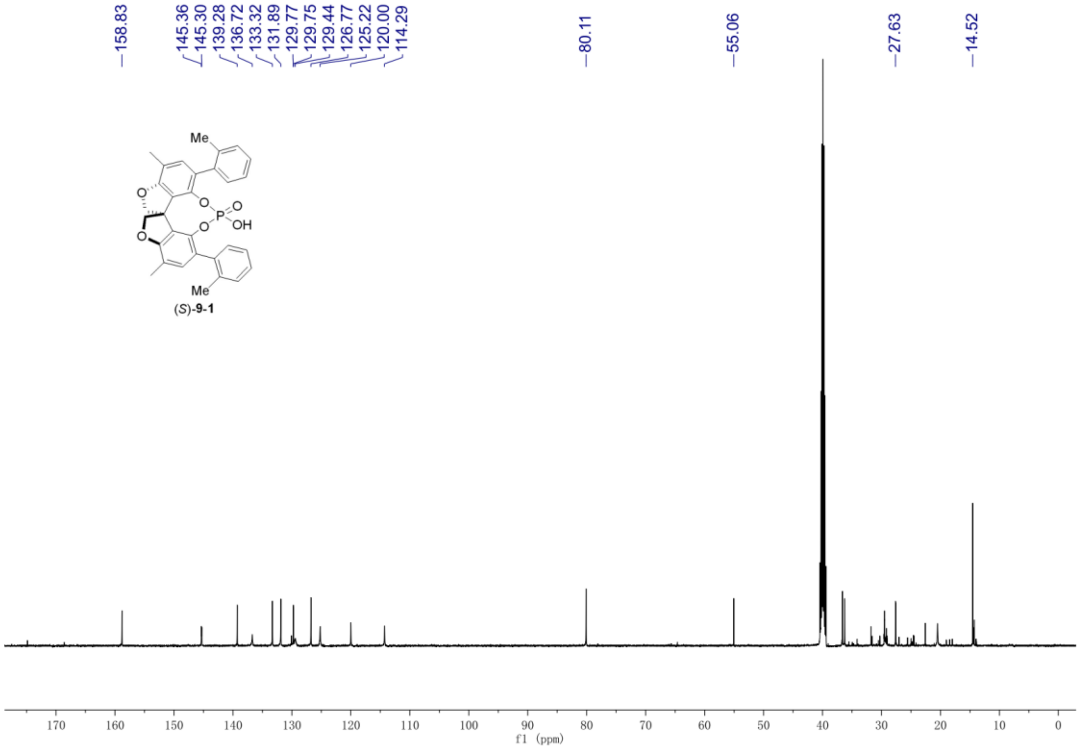

^{13}C NMR of (*S*)-**9-1** (DMSO-d_6)

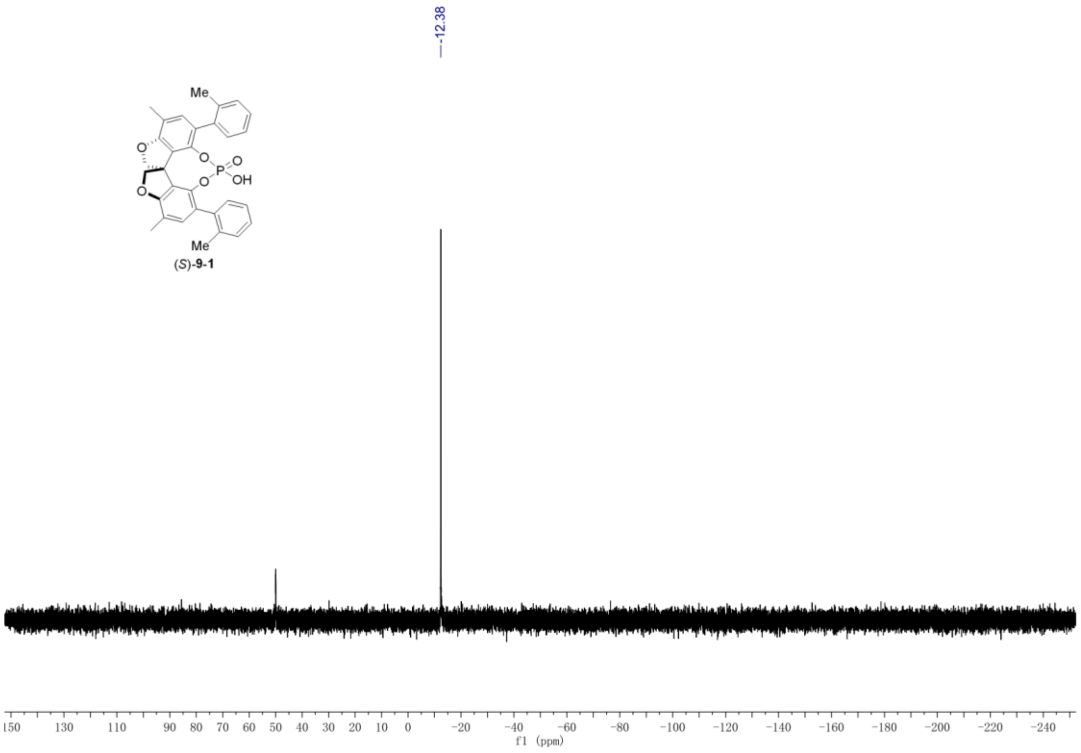

^{31}P NMR of (*S*)-**9-1** (DMSO-d_6)

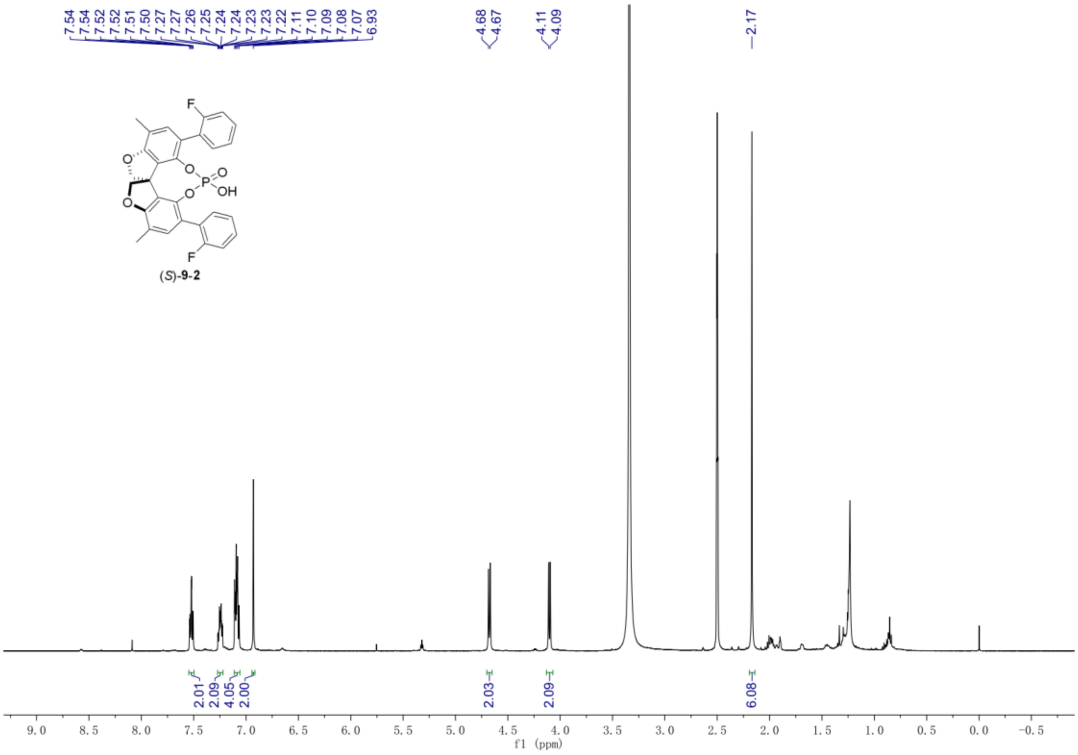

^{1}H NMR of (*S*)-**9-2** (DMSO-d_6)

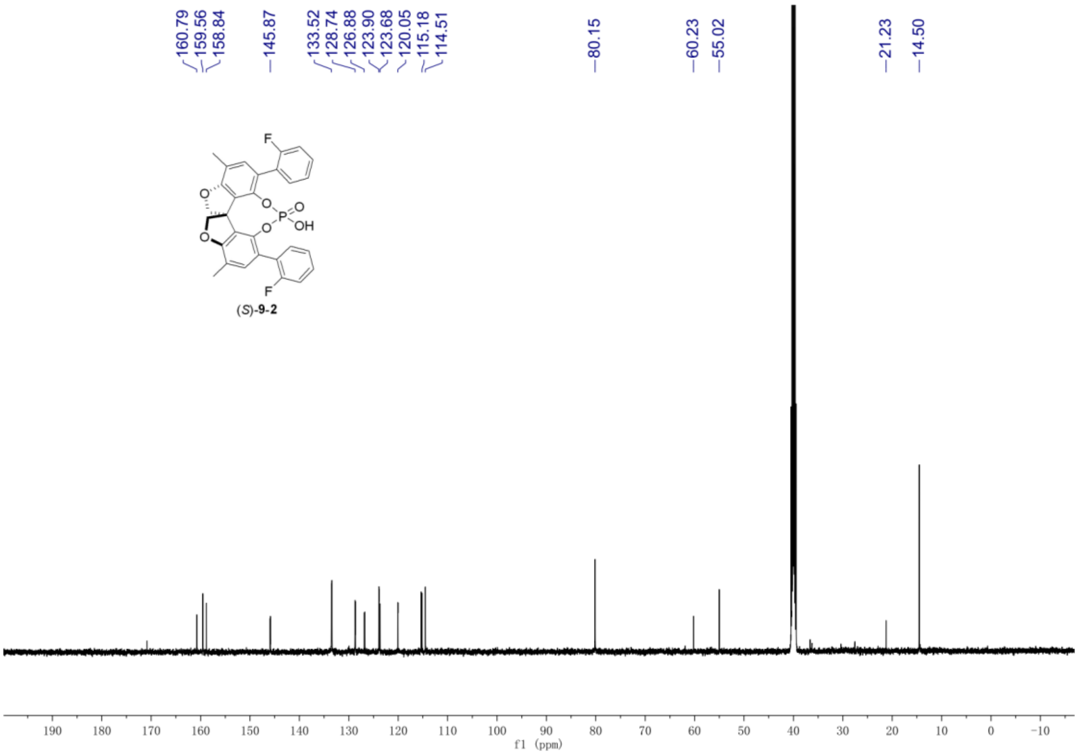

^{13}C NMR of (*S*)-**9-2** (DMSO-d_6)

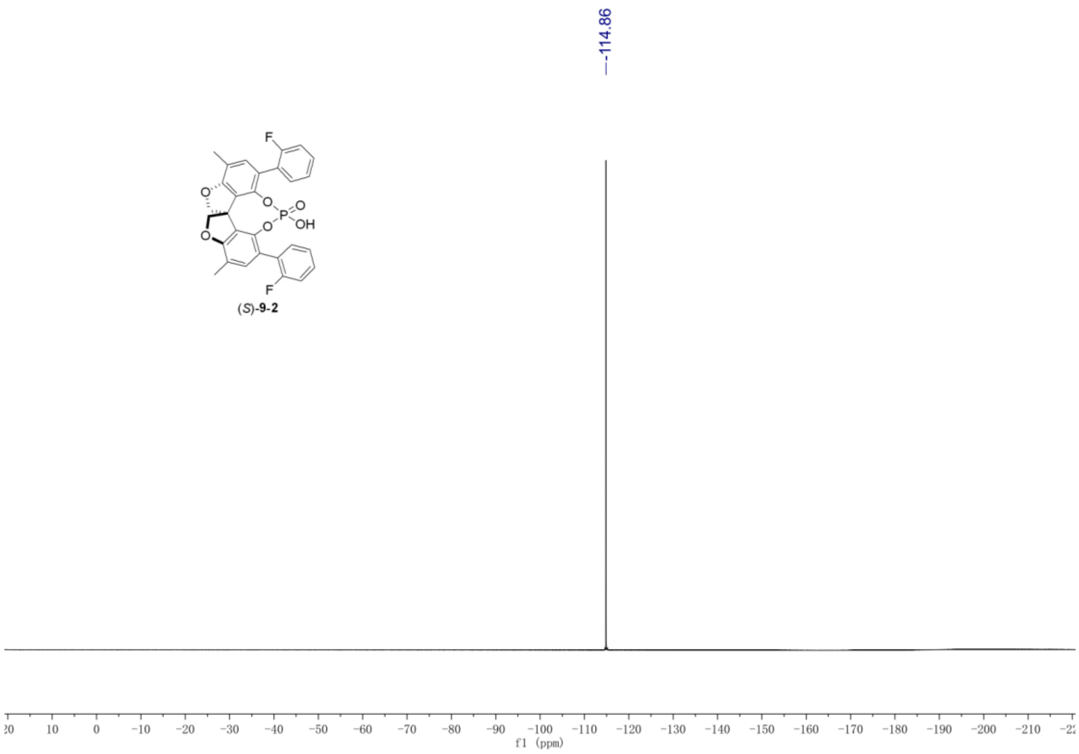

^{19}F NMR of (*S*)-**9-2** (DMSO-d_6)

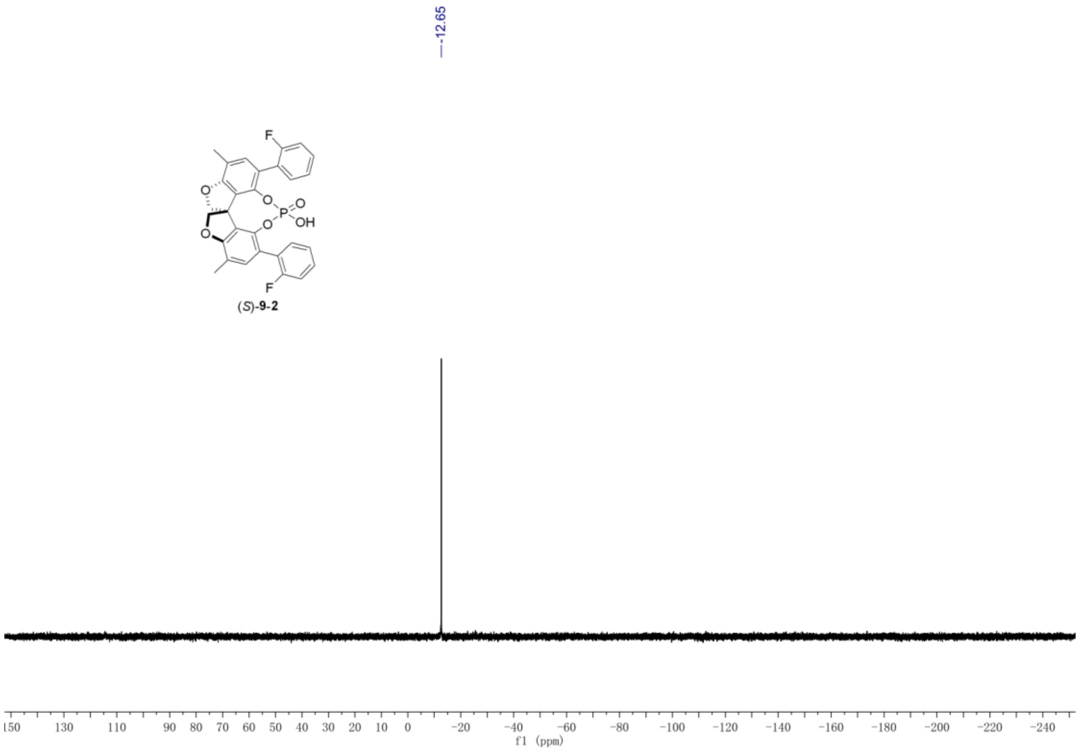

^{31}P NMR of (*S*)-**9-2** (DMSO-d_6)

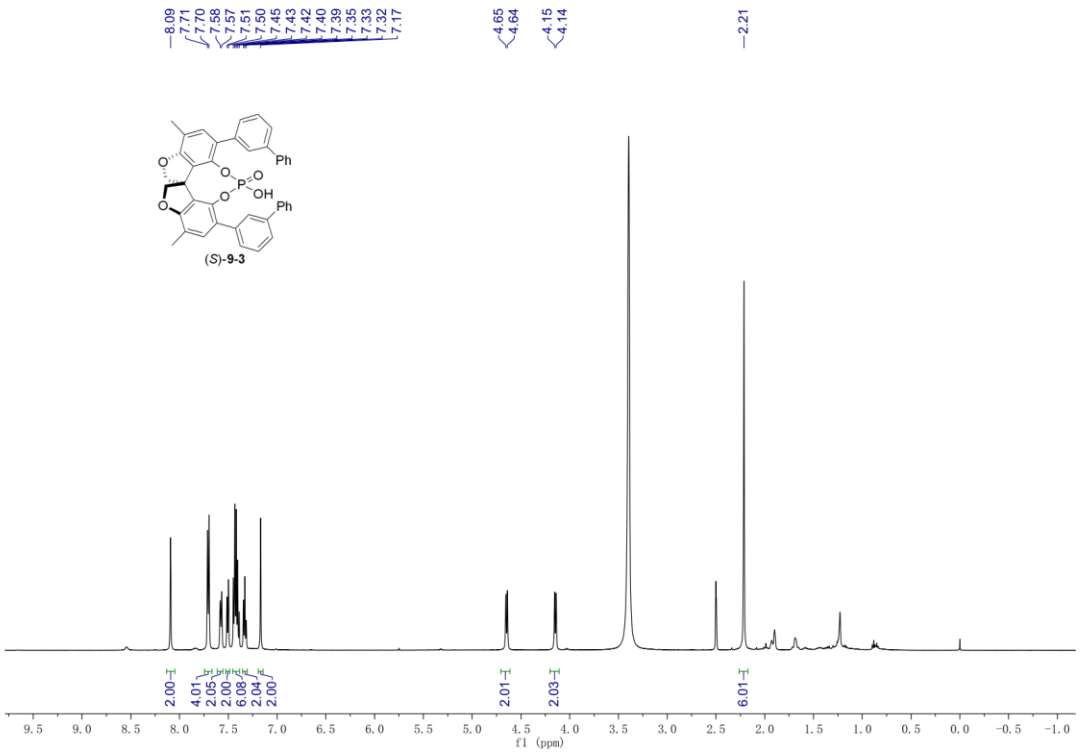

^{1}H NMR of (*S*)-**9-3** (DMSO-*d*$_6$)

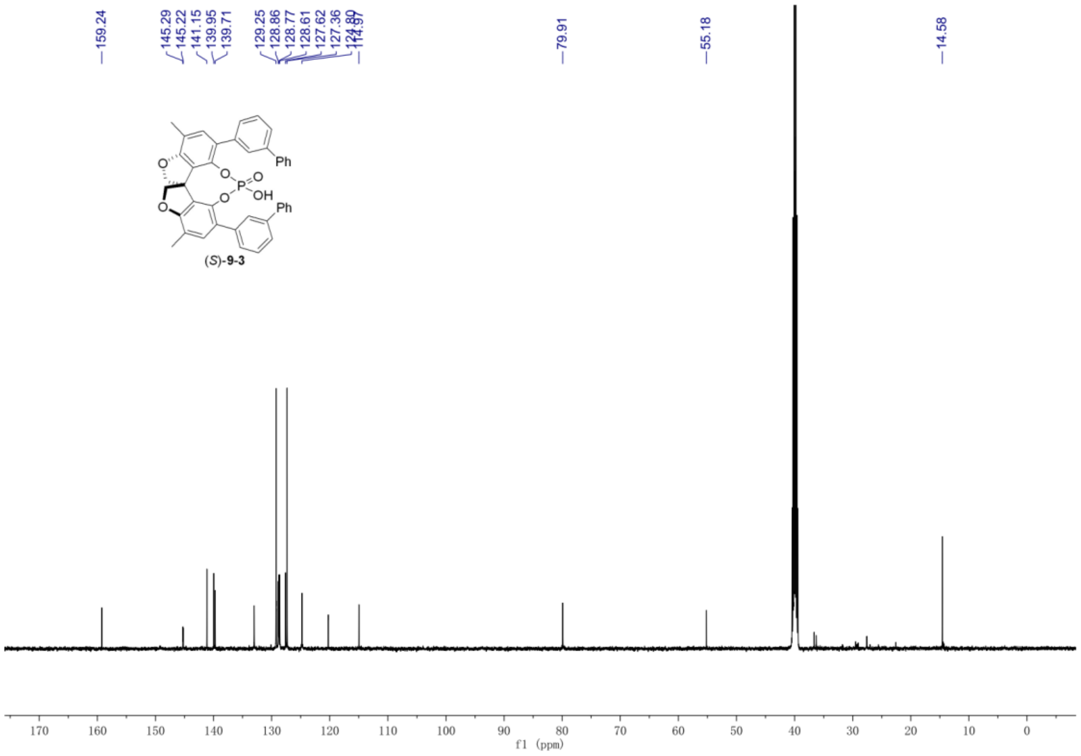

^{13}C NMR of (*S*)-**9-3** (DMSO-*d*$_6$)

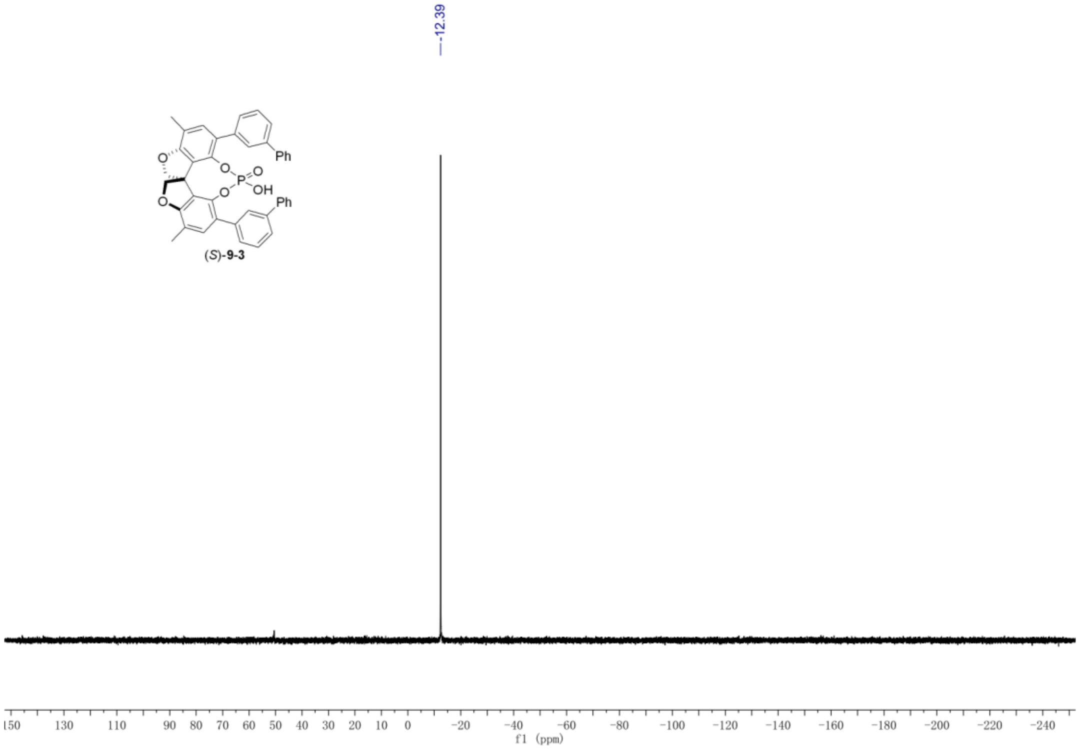

^{31}P NMR of (*S*)-**9-3** (DMSO-d_6)

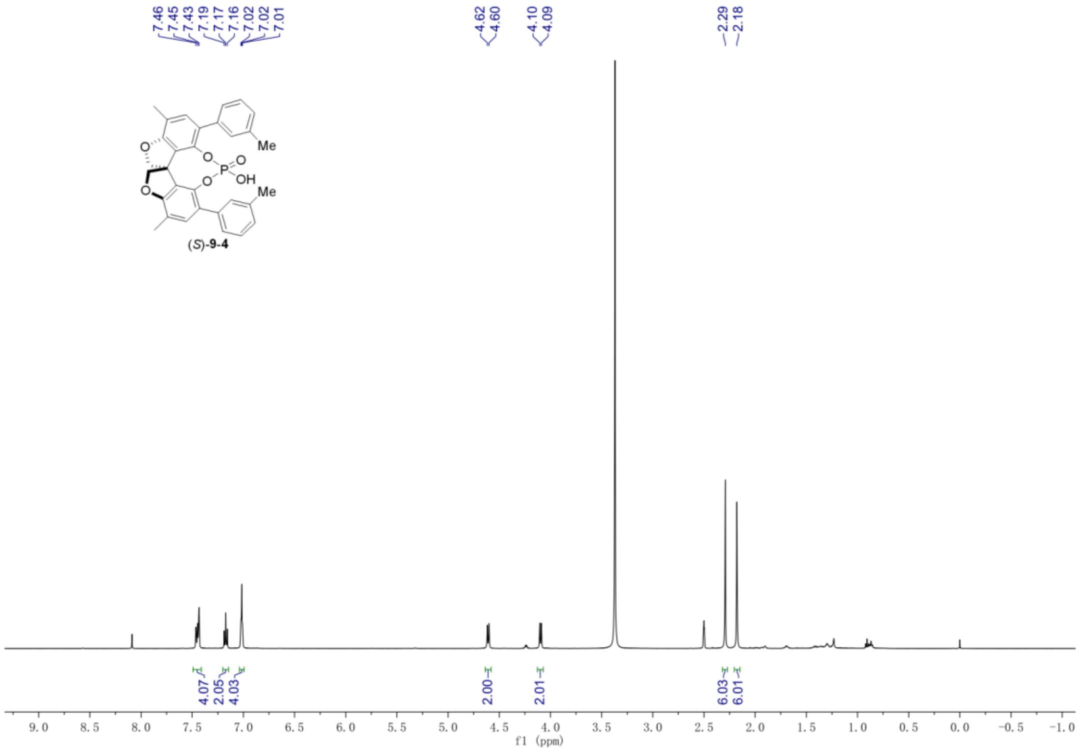

^{1}H NMR of (*S*)-**9-4** (DMSO-d_6)

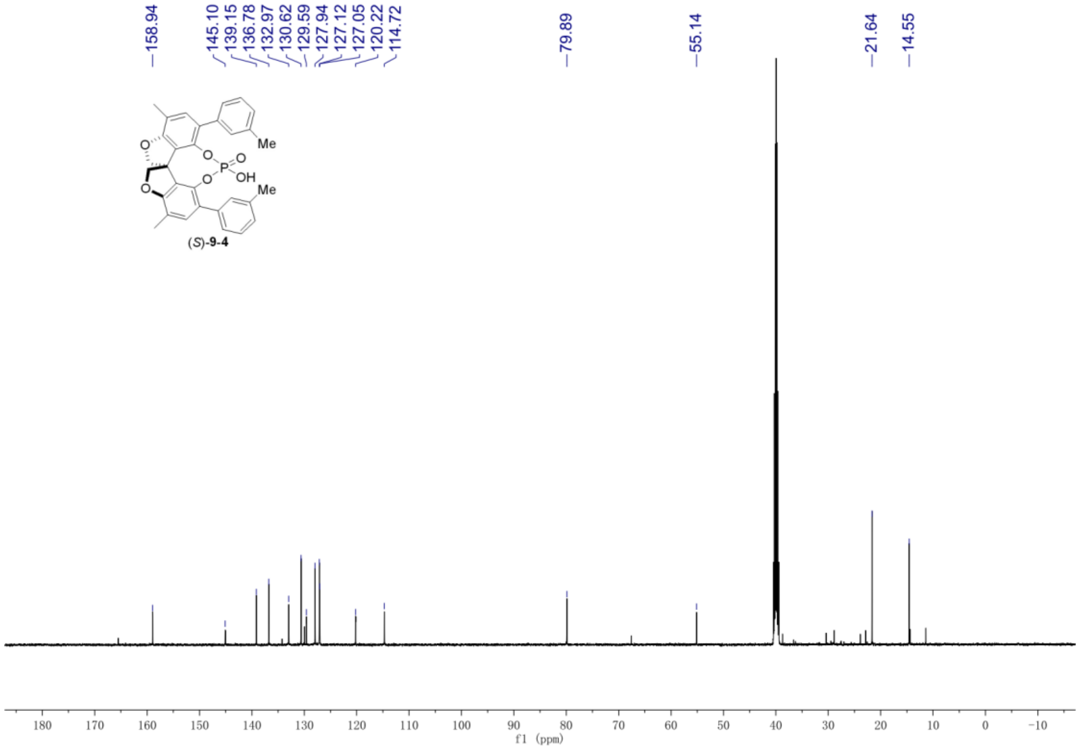

^{13}C NMR of (*S*)-**9-4** (DMSO-d_6)

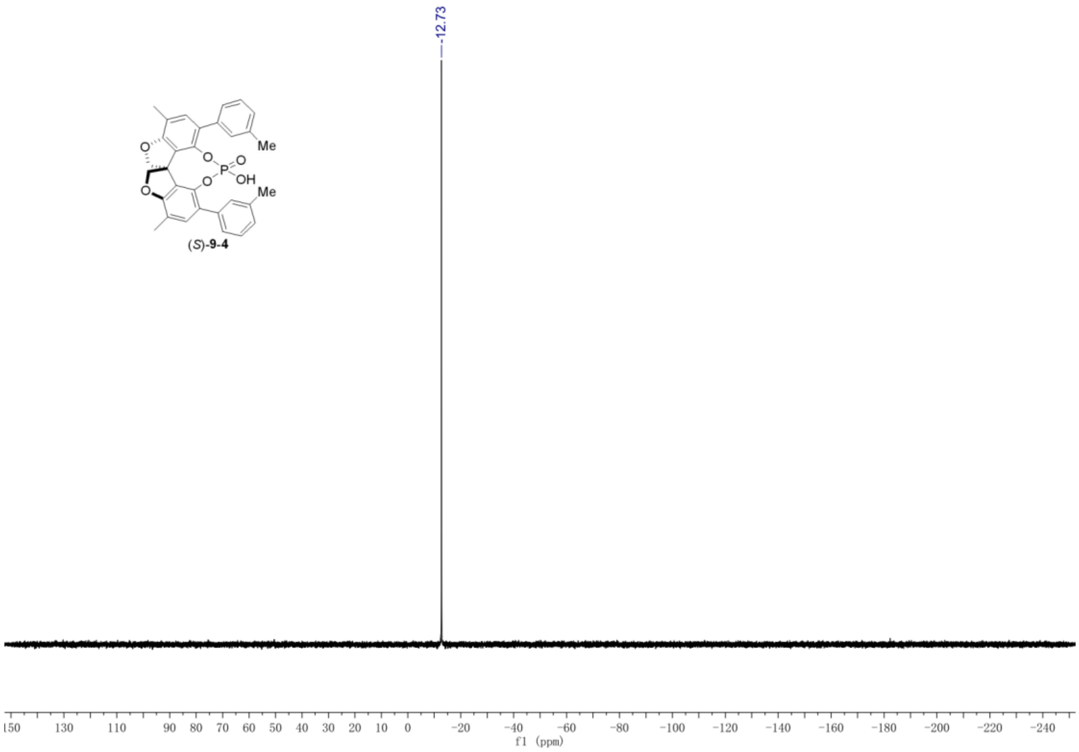

^{31}P NMR of (*S*)-**9-4** (DMSO-d_6)

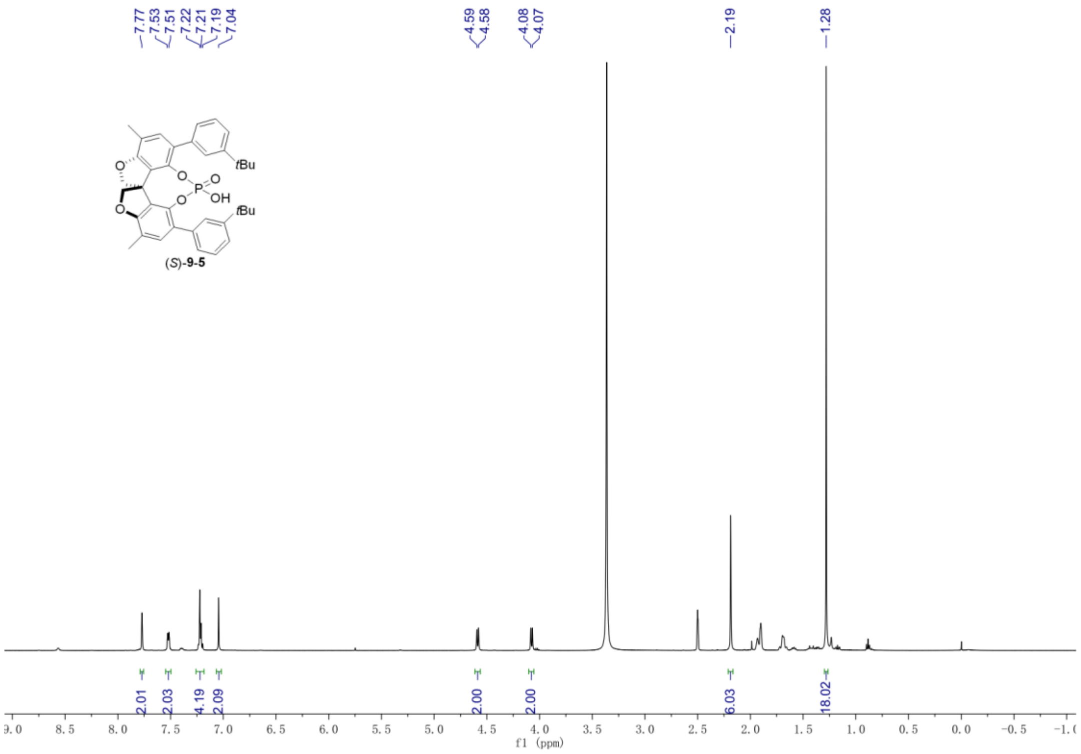

^{1}H NMR of (*S*)-**9-5** (DMSO-d_6)

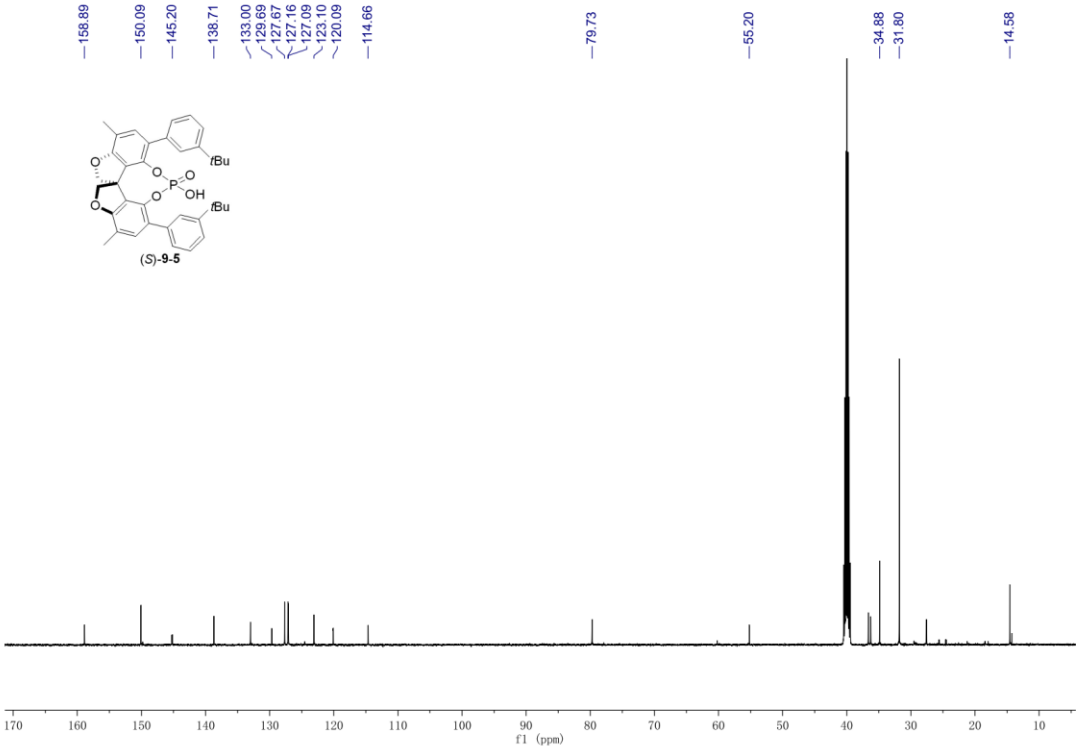

^{13}C NMR of (*S*)-**9-5** (DMSO-d_6)

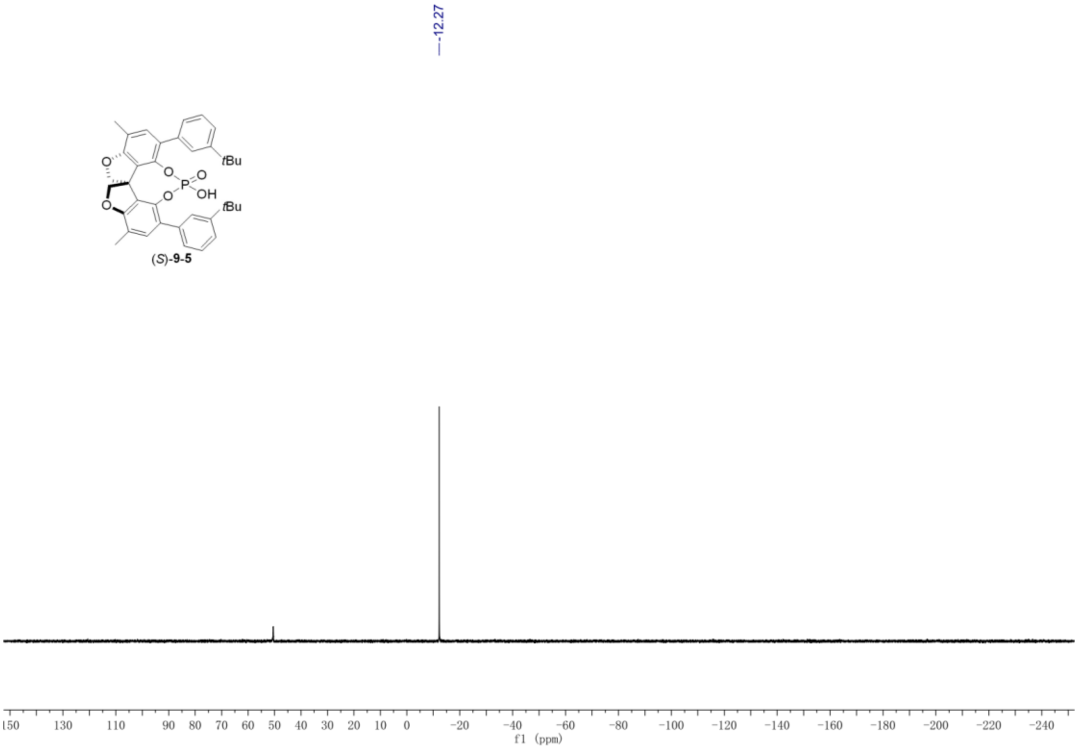

^{31}P NMR of (*S*)-**9-5** (DMSO-d_6)

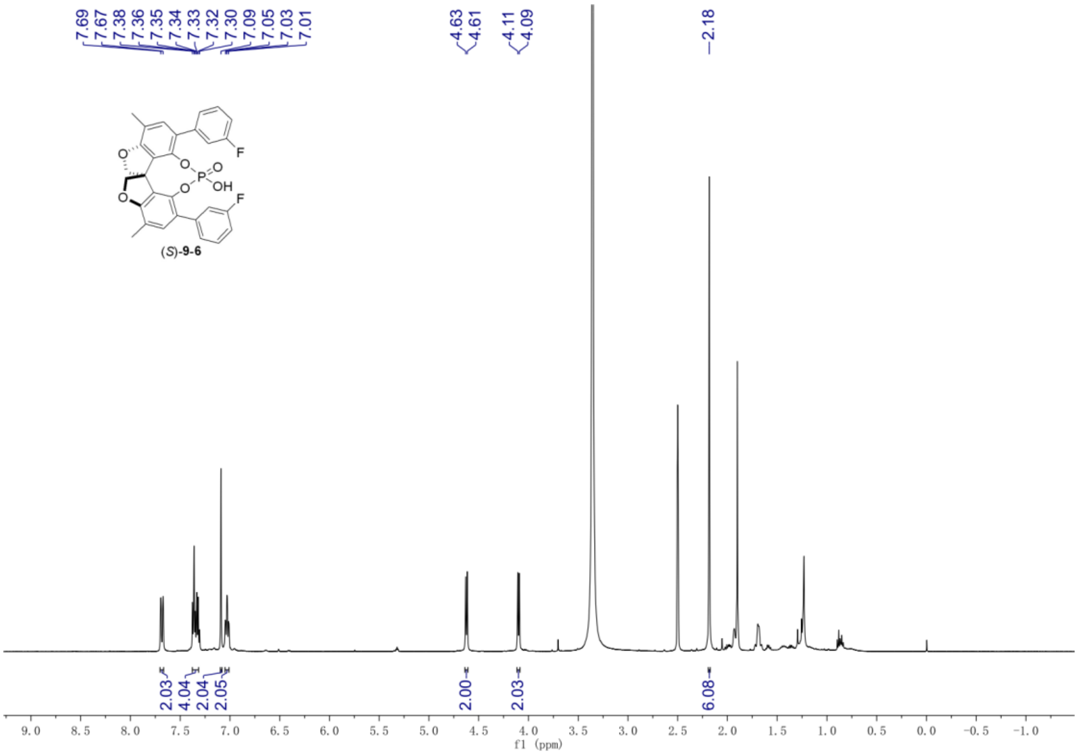

^{1}H NMR of (*S*)-**9-6** (DMSO-d_6)

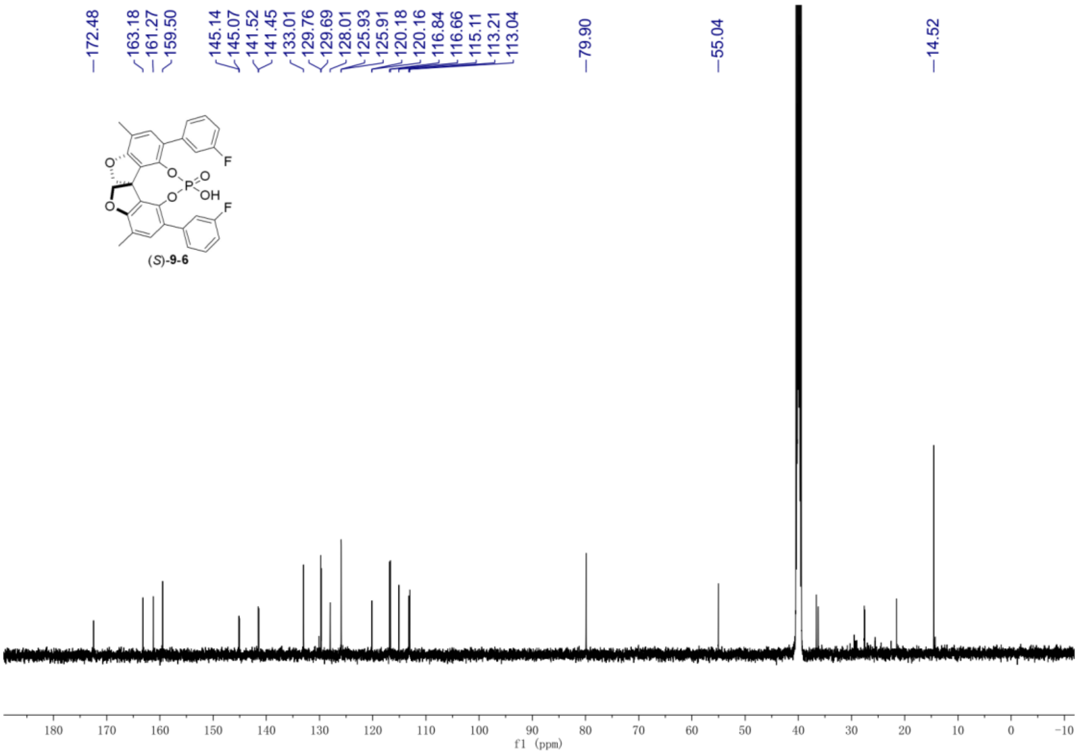

^{13}C NMR of (*S*)-**9-6** (DMSO-*d*$_6$)

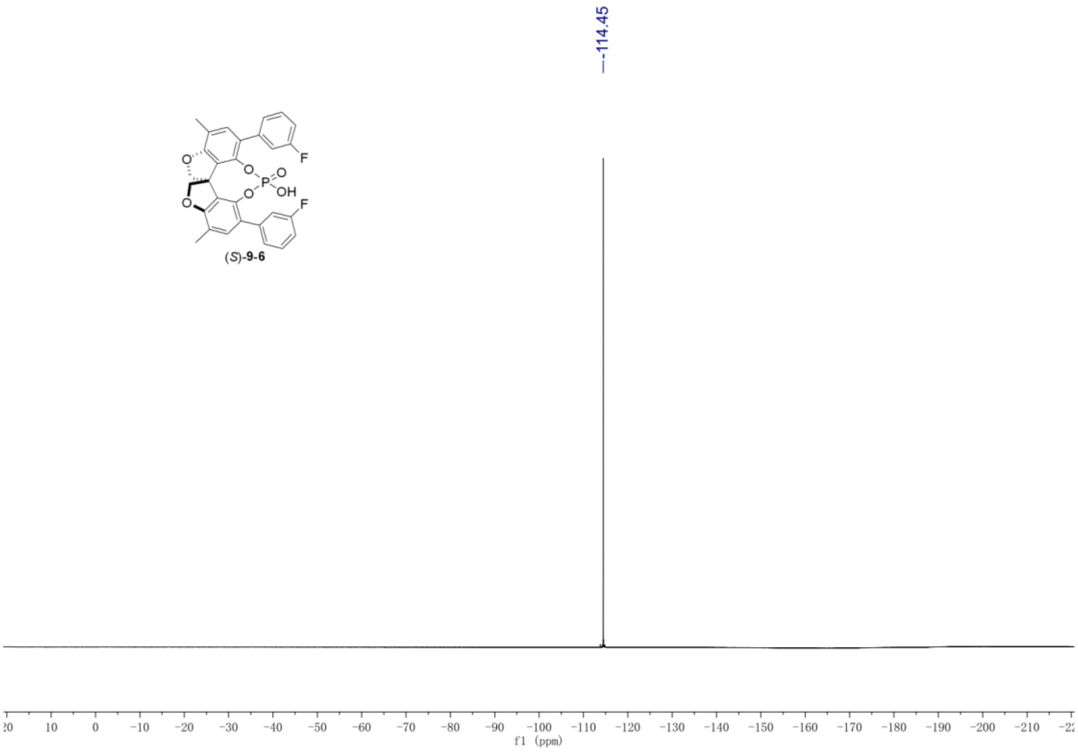

^{19}F NMR of (*S*)-**9-6** (DMSO-*d*$_6$)

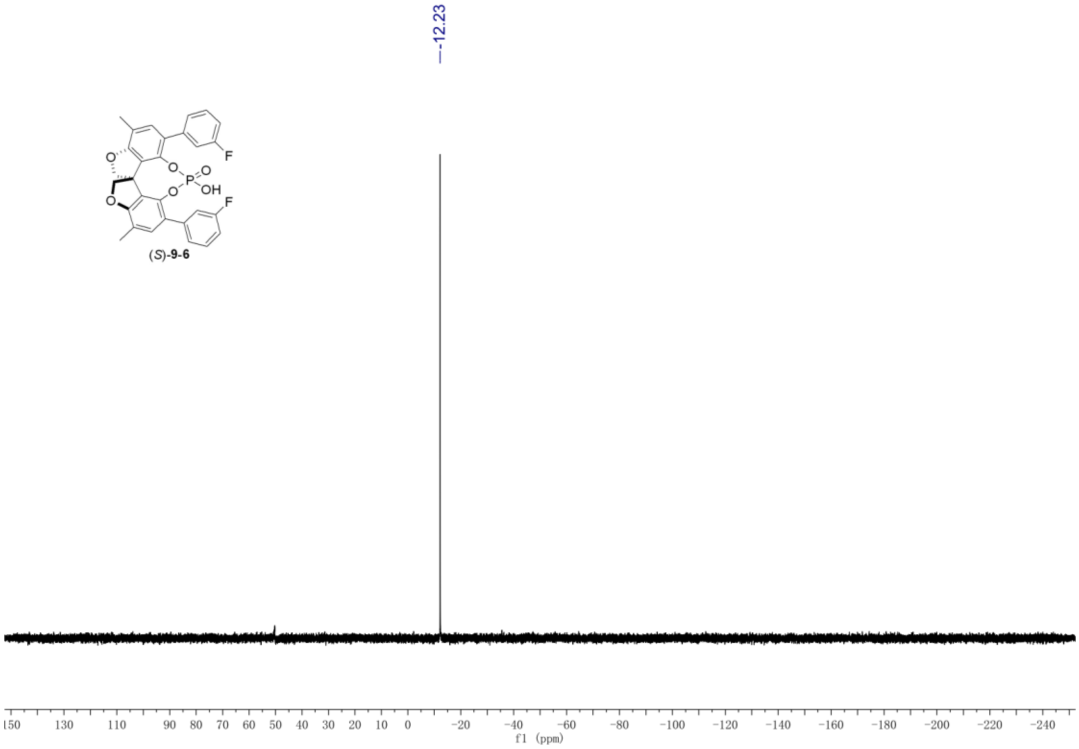

^{31}P NMR of (*S*)-**9-6** (DMSO-d_6)

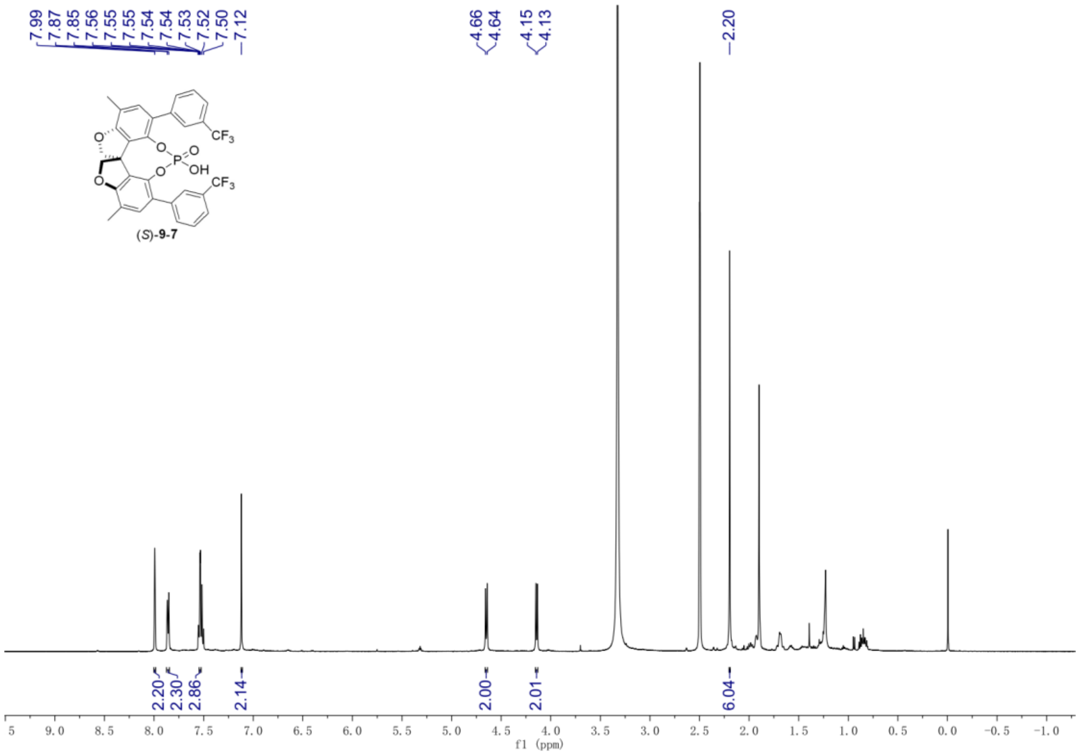

^{1}H NMR of (*S*)-**9-7** (DMSO-d_6)

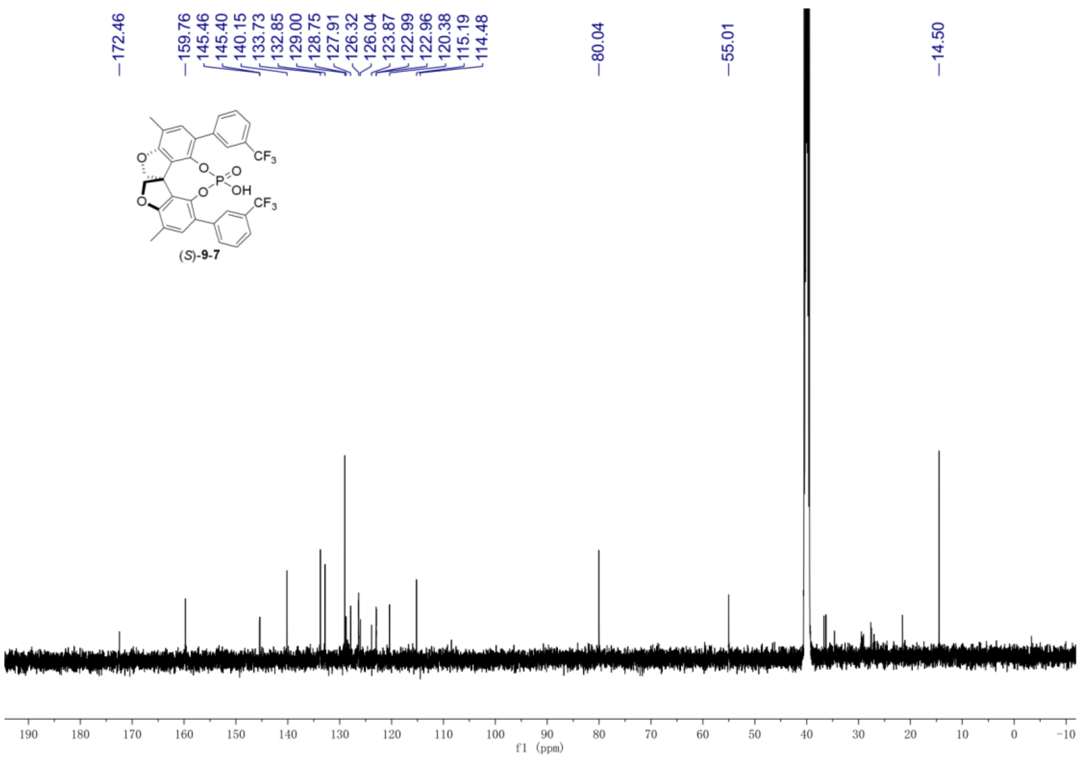

^{13}C NMR of (*S*)-**9-7** (DMSO-d_6)

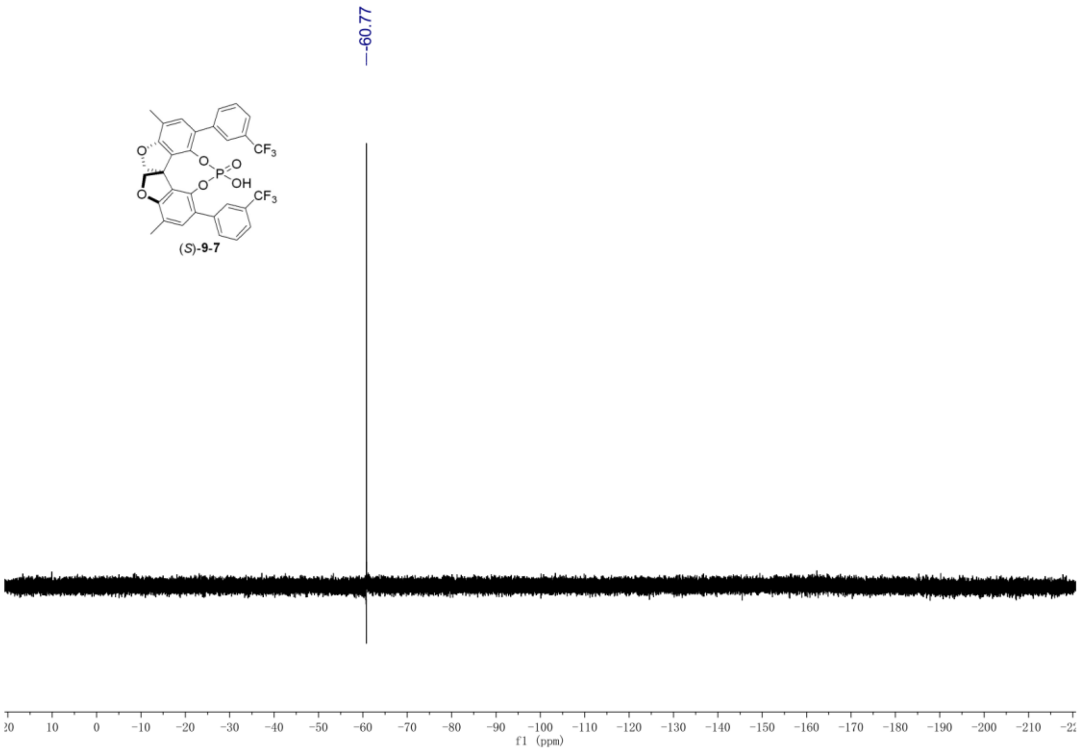

^{19}F NMR of (*S*)-**9-7** (DMSO-d_6)

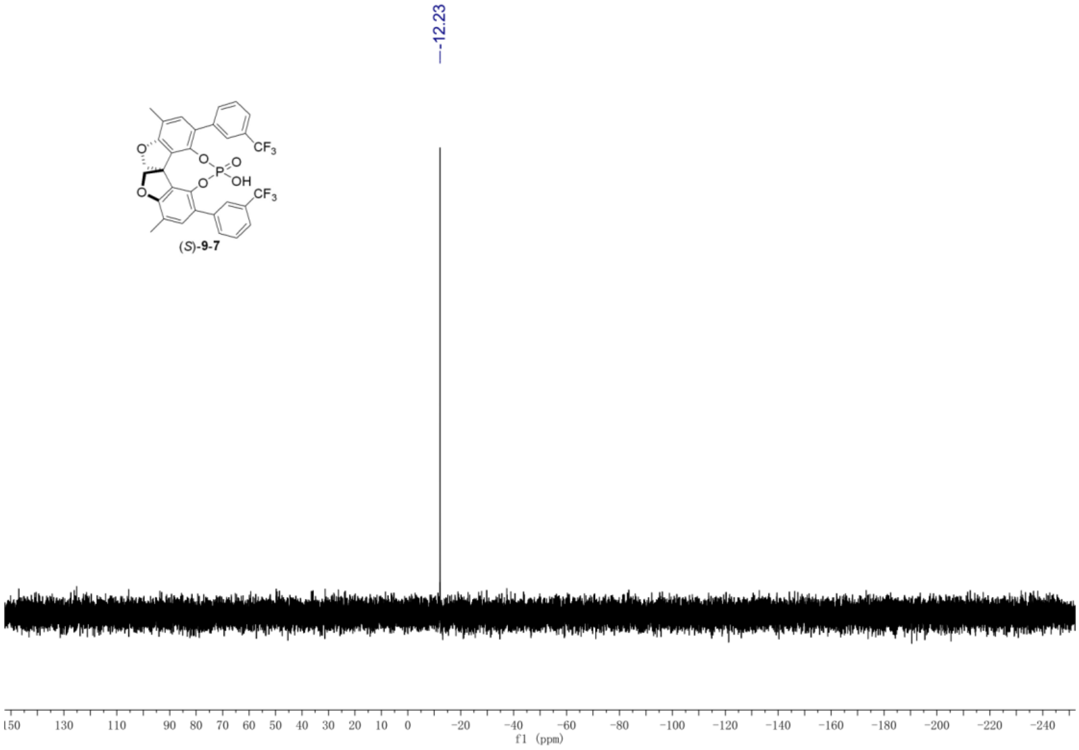

^{31}P NMR of (S)-**9-7** (DMSO-d_6)

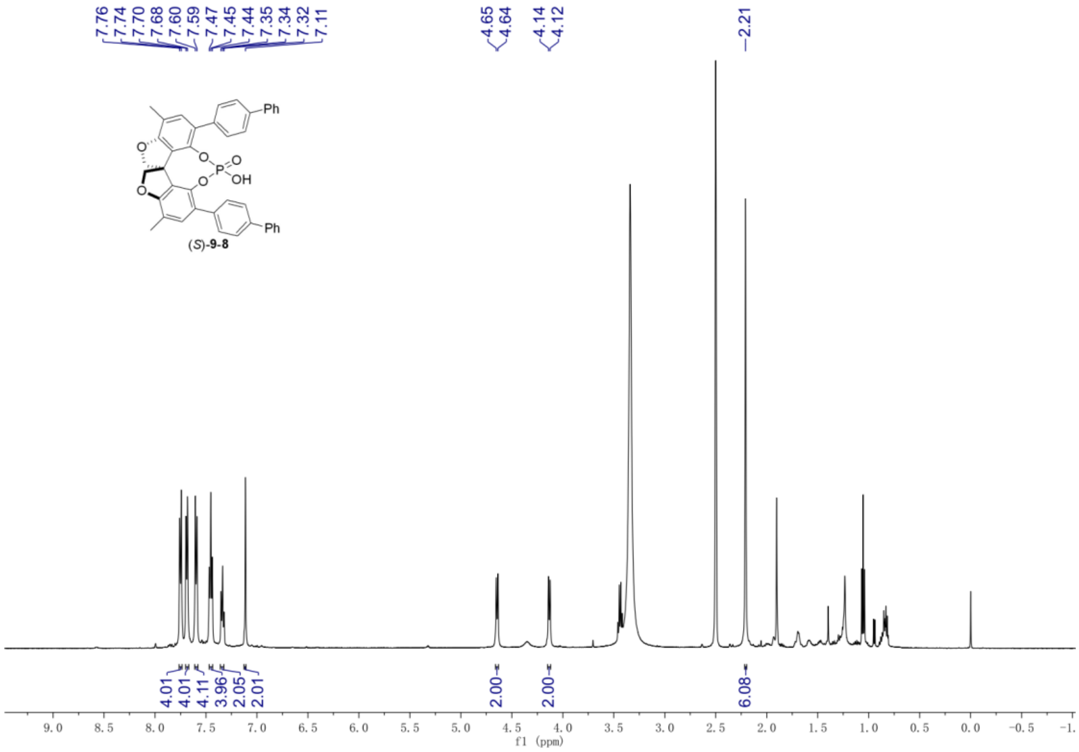

^{1}H NMR of (S)-**9-8** (DMSO-d_6)

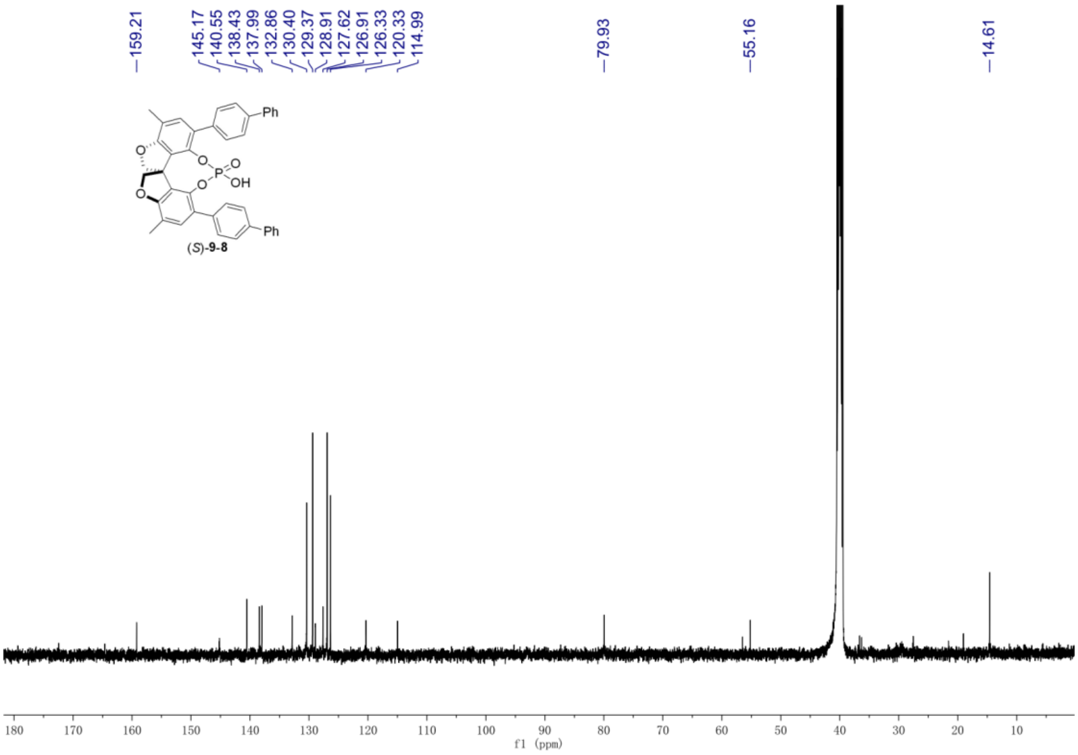

^{13}C NMR of (*S*)-**9-8** (DMSO-d_6)

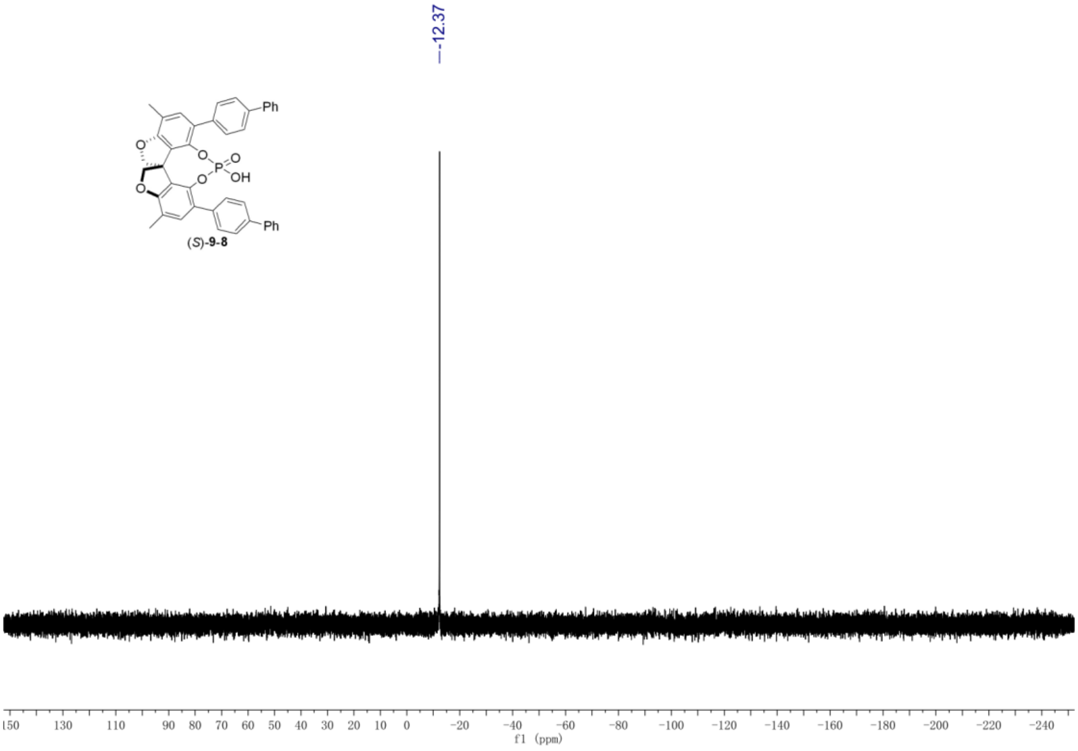

^{31}P NMR of (*S*)-**9-8** (DMSO-d_6)

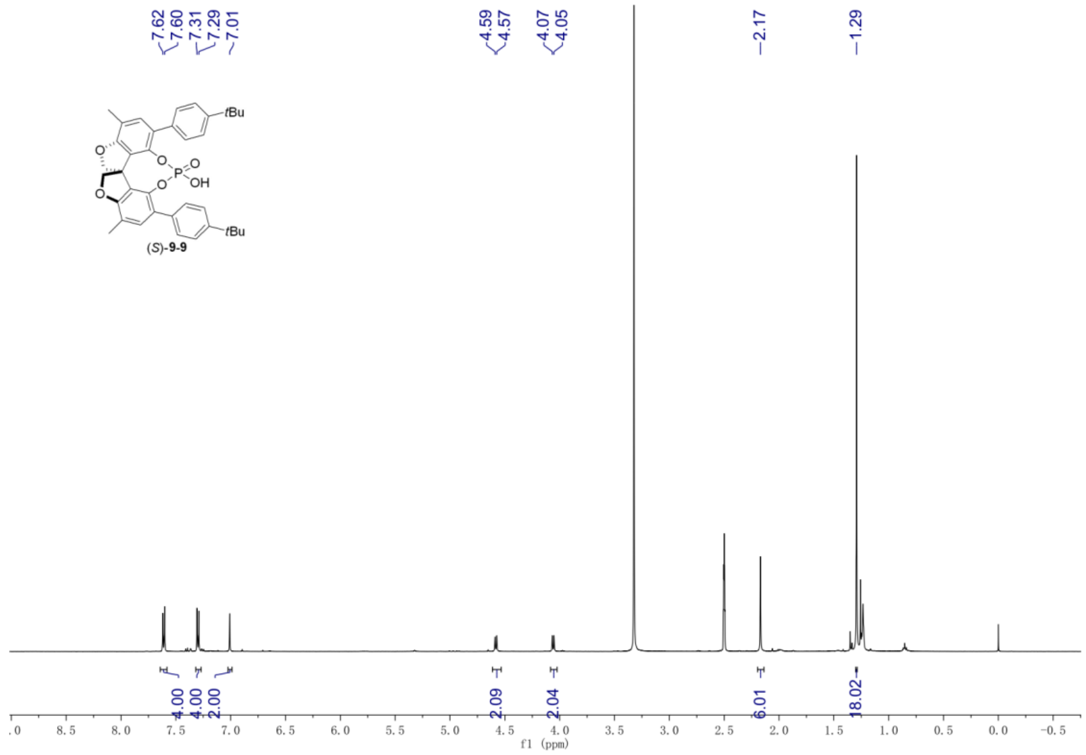

^{1}H NMR of (*S*)-**9-9** (DMSO-d_6)

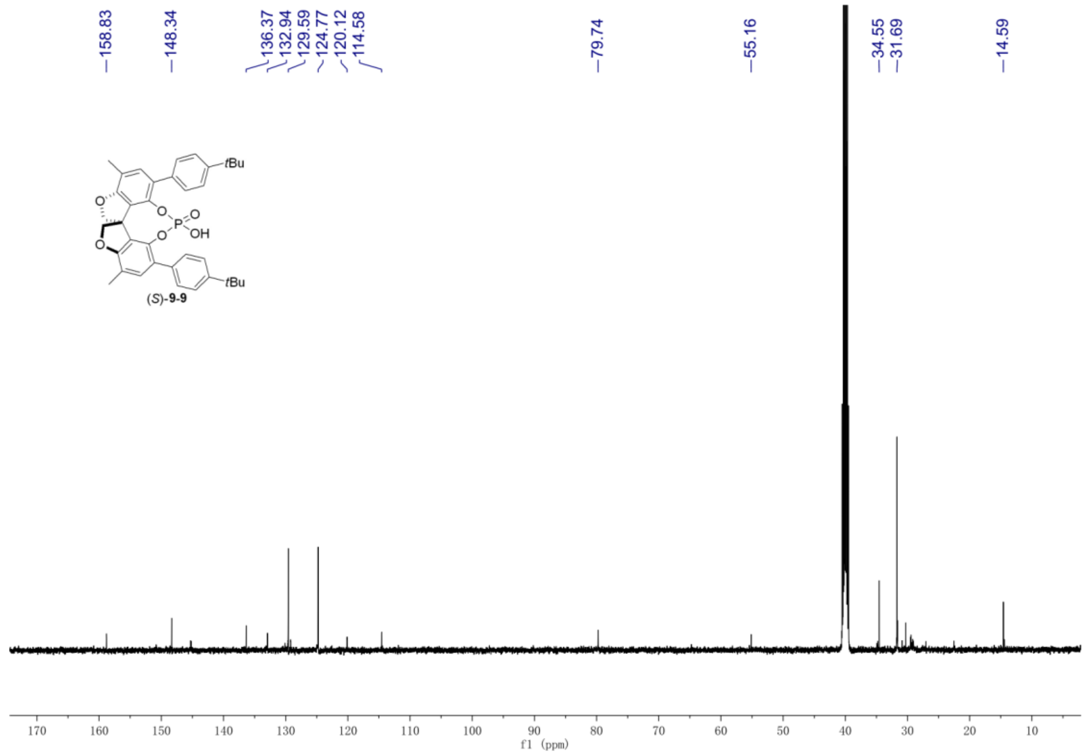

^{13}C NMR of (*S*)-**9-9** (DMSO-d_6)

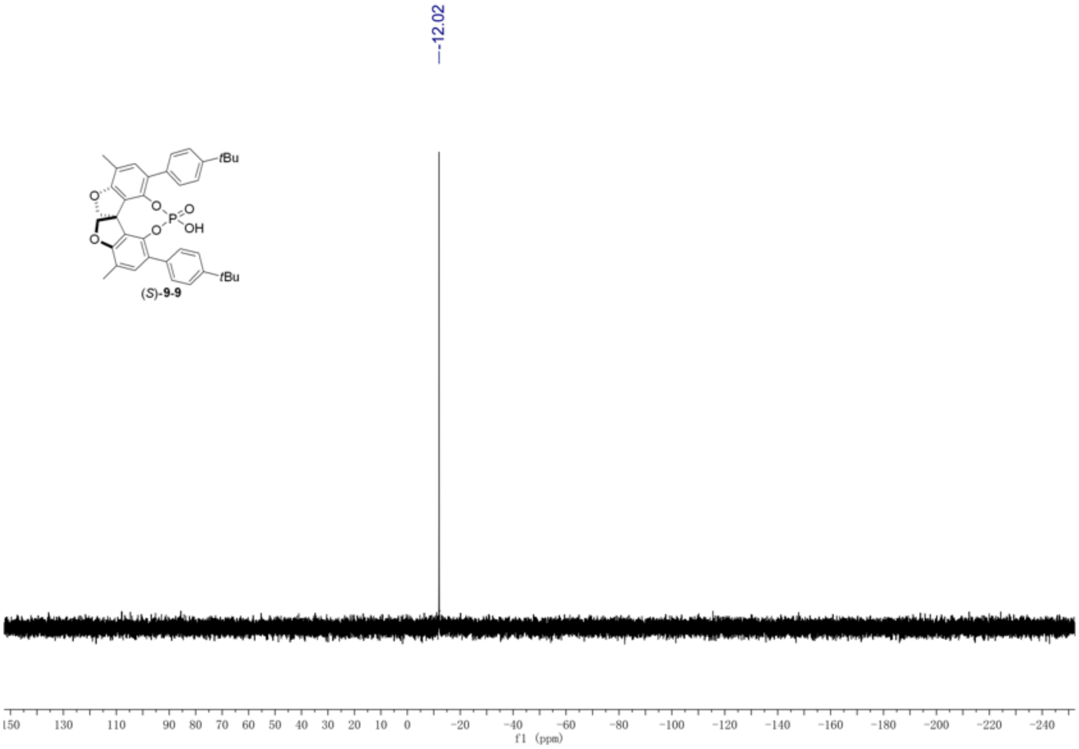

^{31}P NMR of (*S*)-**9-9** (DMSO-d_6)

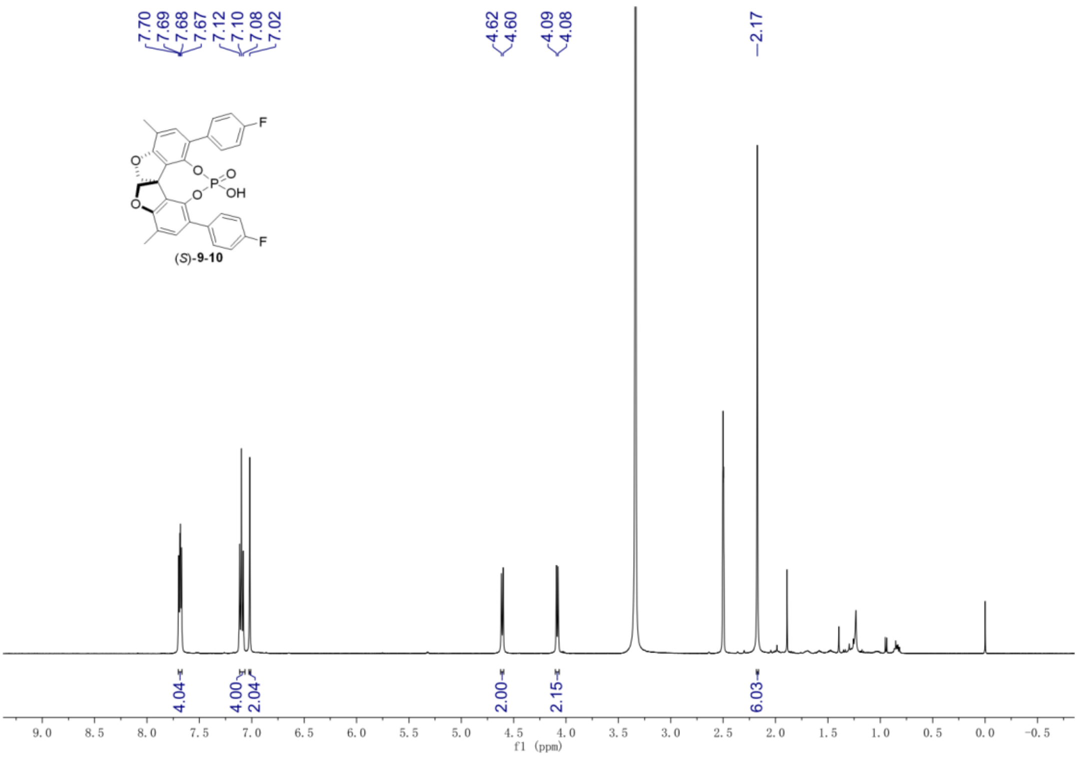

^{1}H NMR of (*S*)-**9-10** (DMSO-d_6)

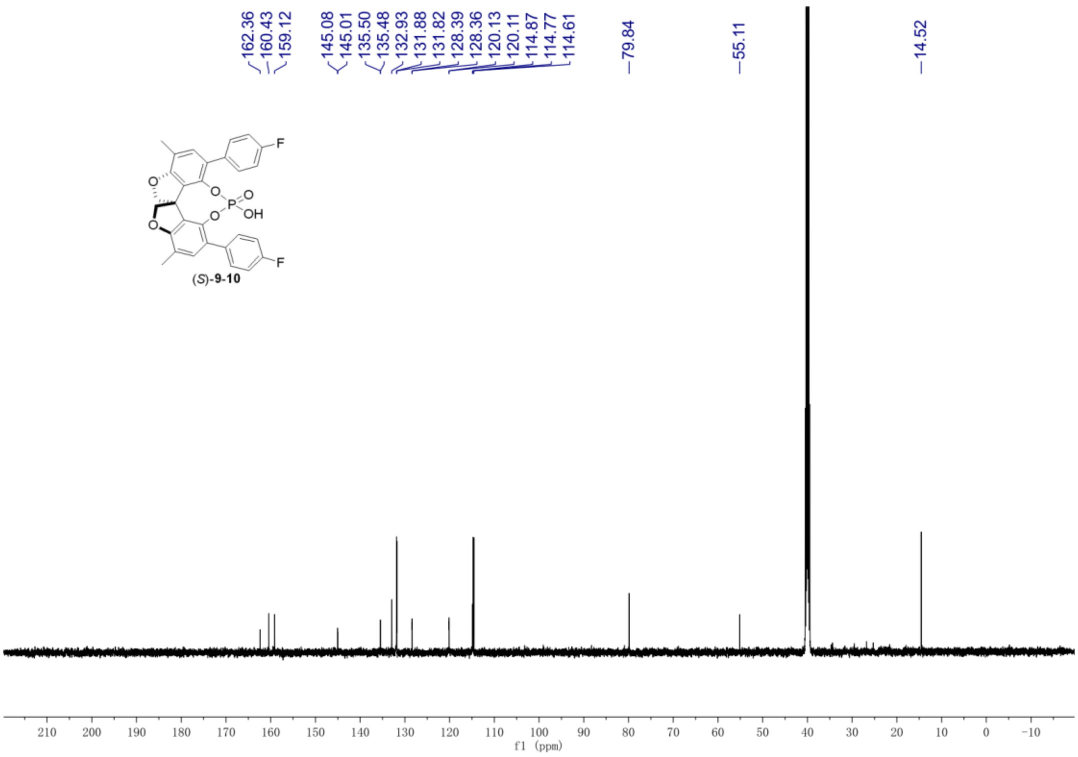

^{13}C NMR of (*S*)-**9-10** (DMSO-*d*$_6$)

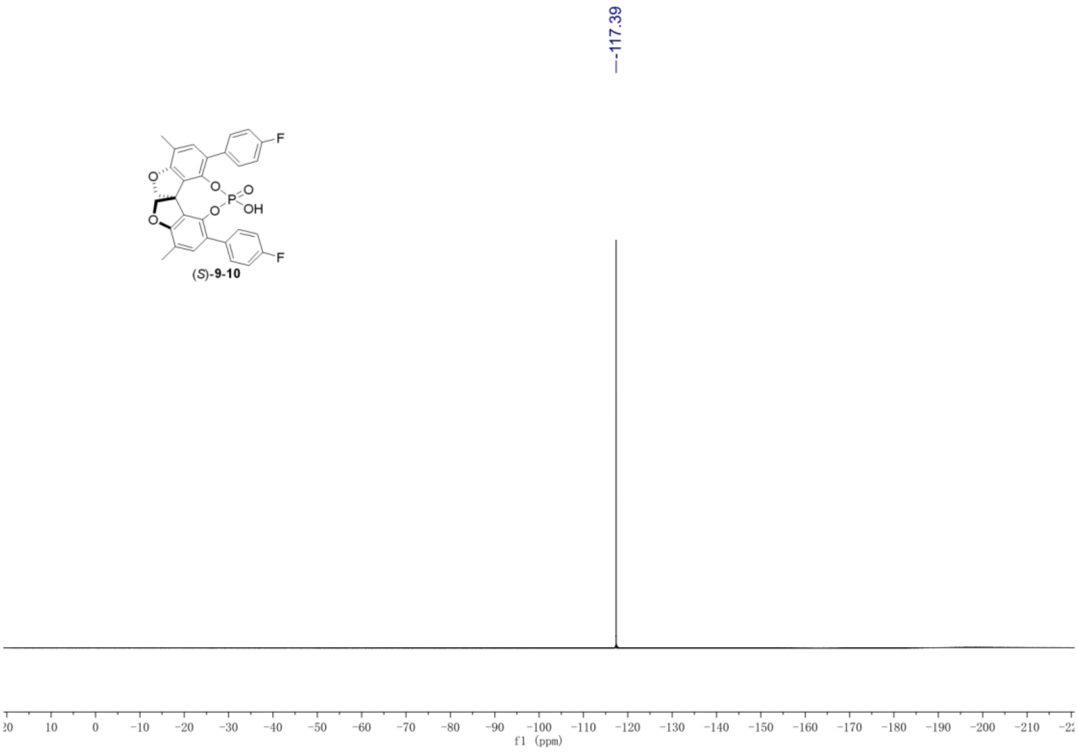

^{19}F NMR of (*S*)-**9-10** (DMSO-*d*$_6$)

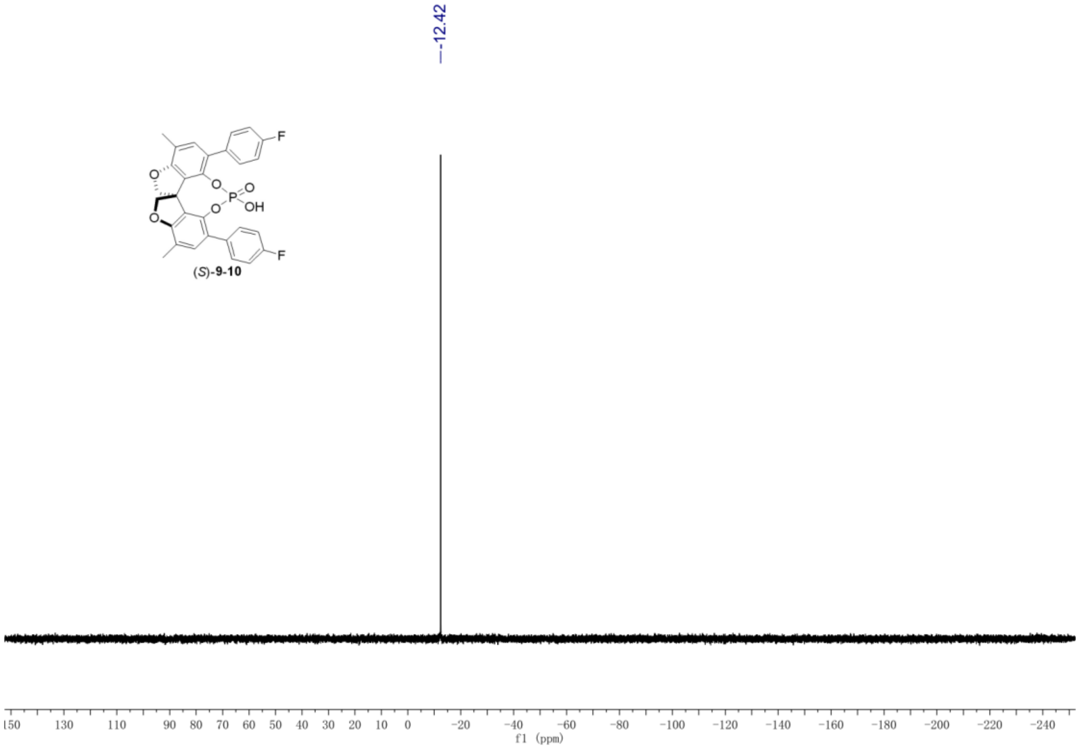

^{31}P NMR of (*S*)-**9-10** (DMSO-d_6)

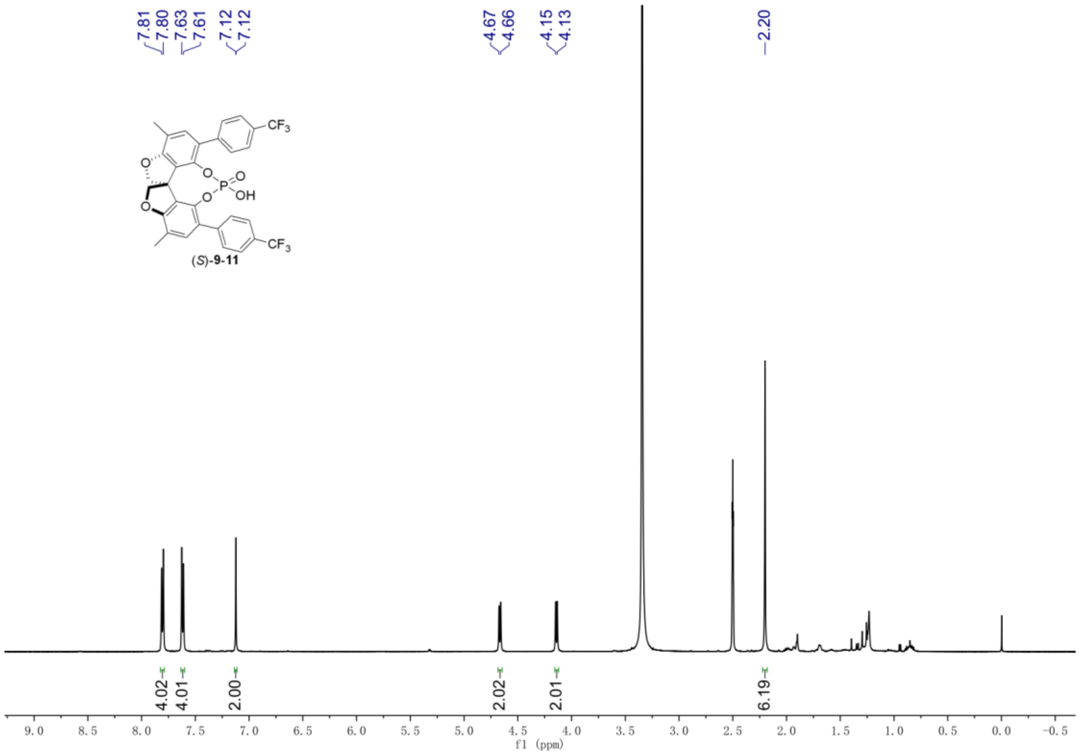

^{1}H NMR of (*S*)-**9-11** (DMSO-d_6)

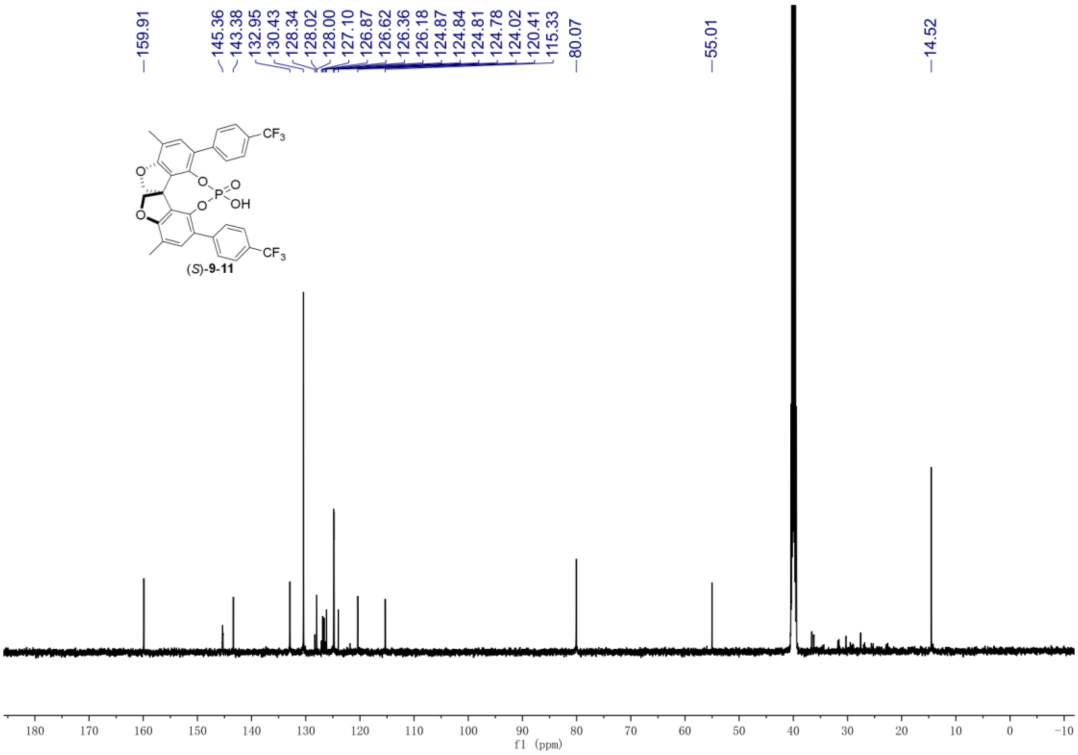

^{13}C NMR of (*S*)-**9-11** (DMSO-d_6)

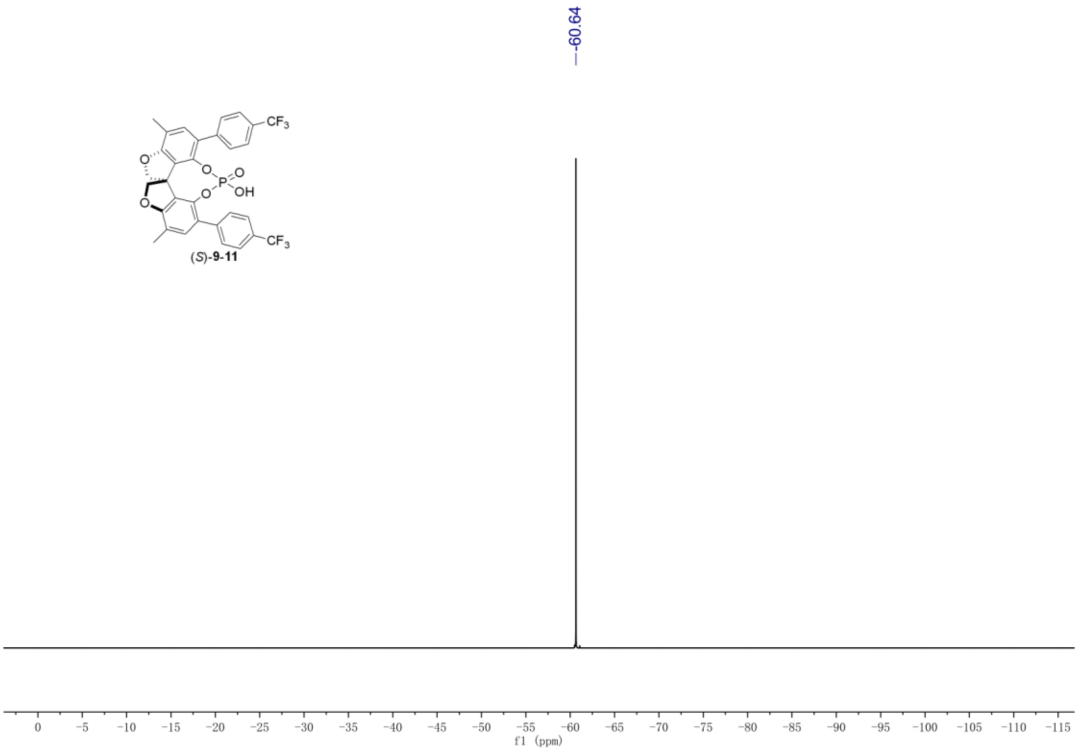

^{19}F NMR of (*S*)-**9-11** (DMSO-d_6)

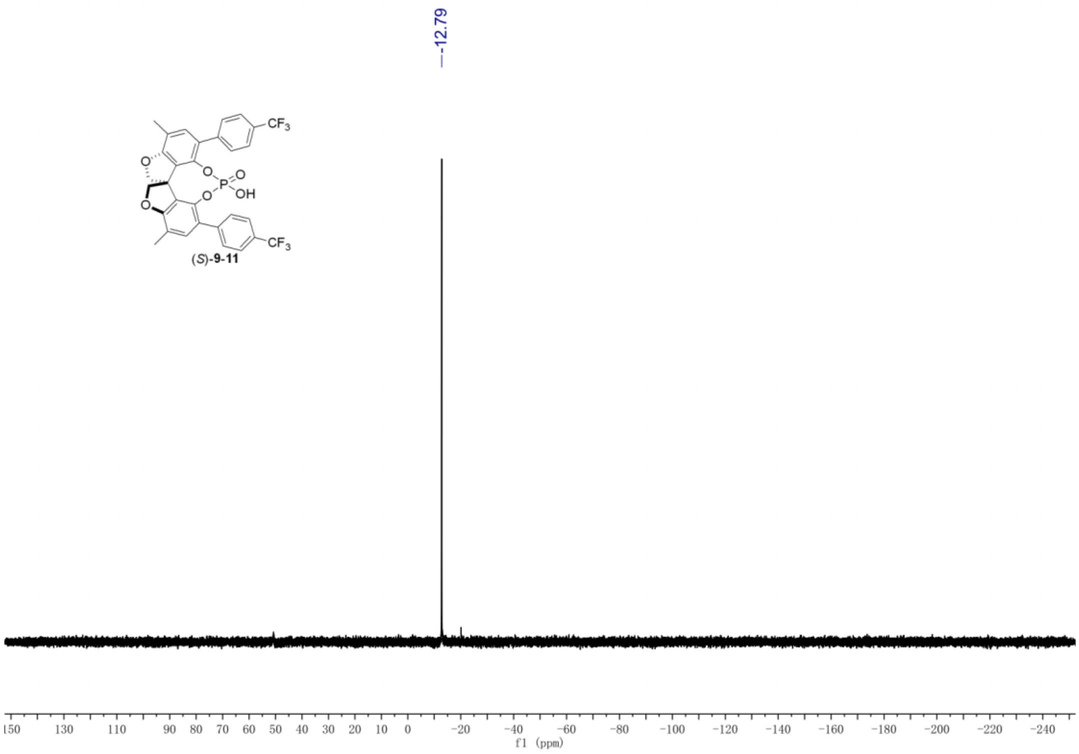

^{31}P NMR of (*S*)-**9-11** (DMSO-d_6)

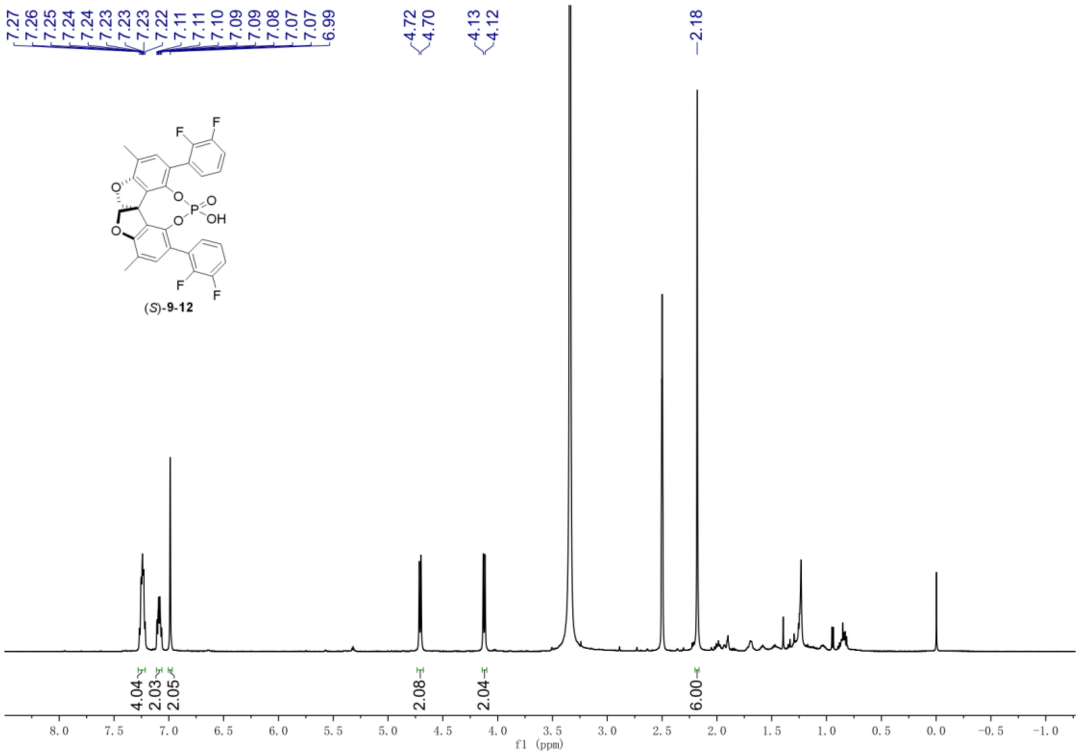

^{1}H NMR of (*S*)-**9-12** (DMSO-d_6)

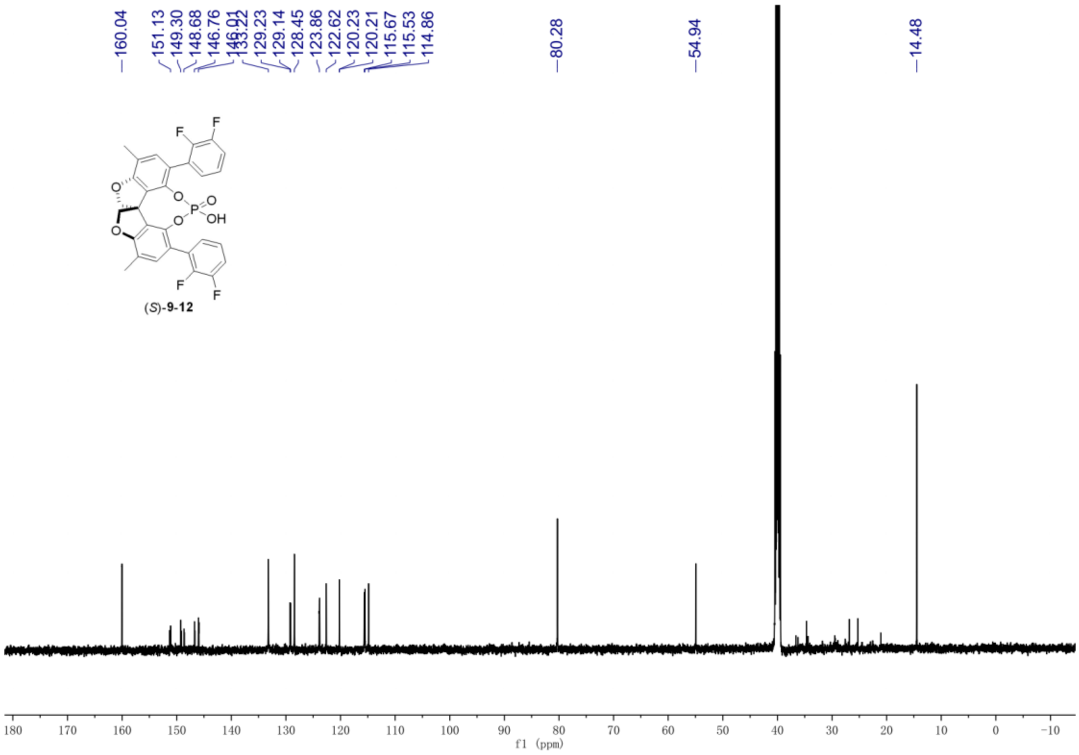

^{13}C NMR of (*S*)-**9-12** (DMSO-d_6)

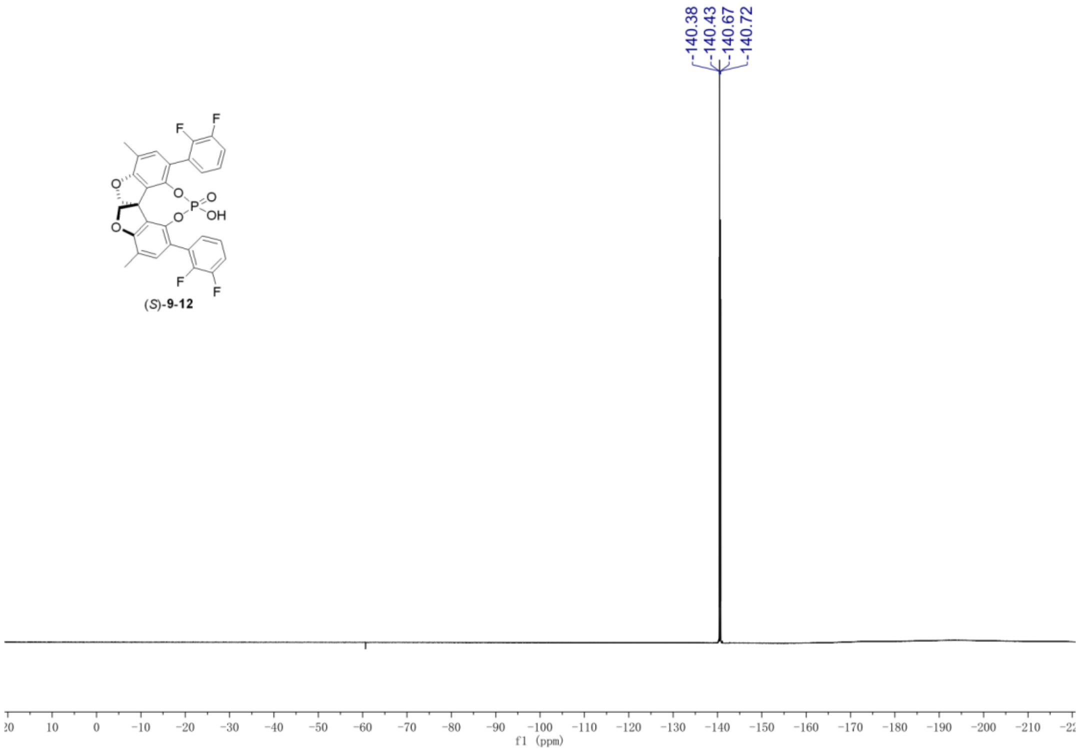

^{19}F NMR of (*S*)-**9-12** (DMSO-d_6)

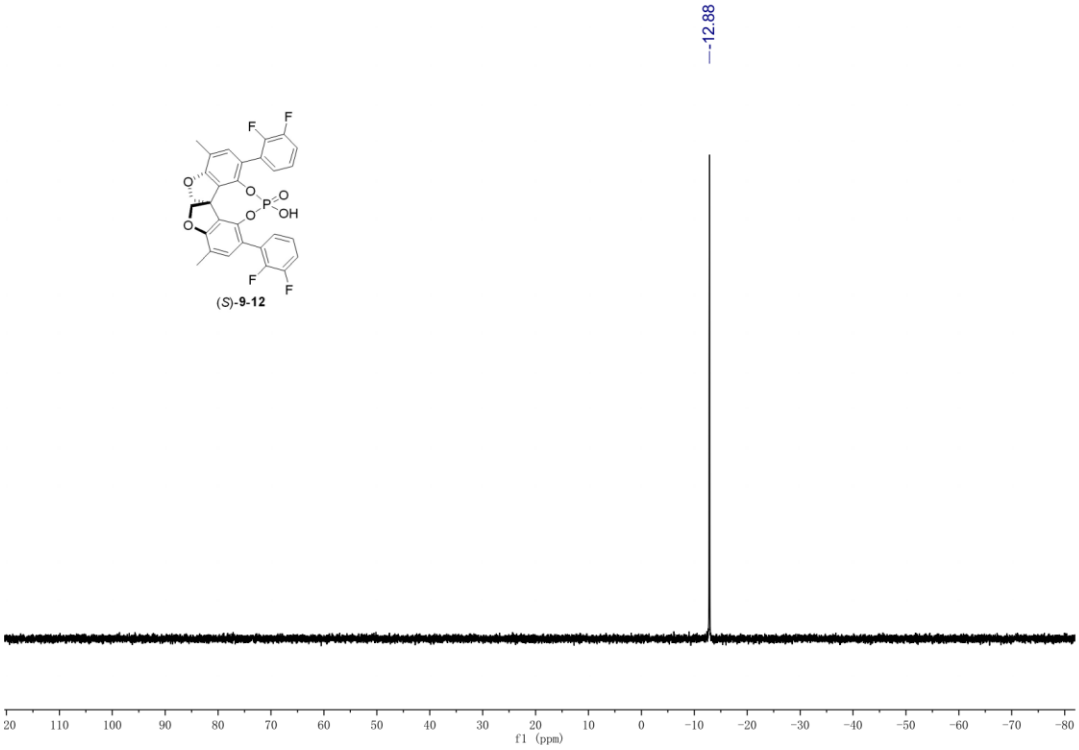

^{31}P NMR of (*S*)-**9-12** (DMSO-d_6)

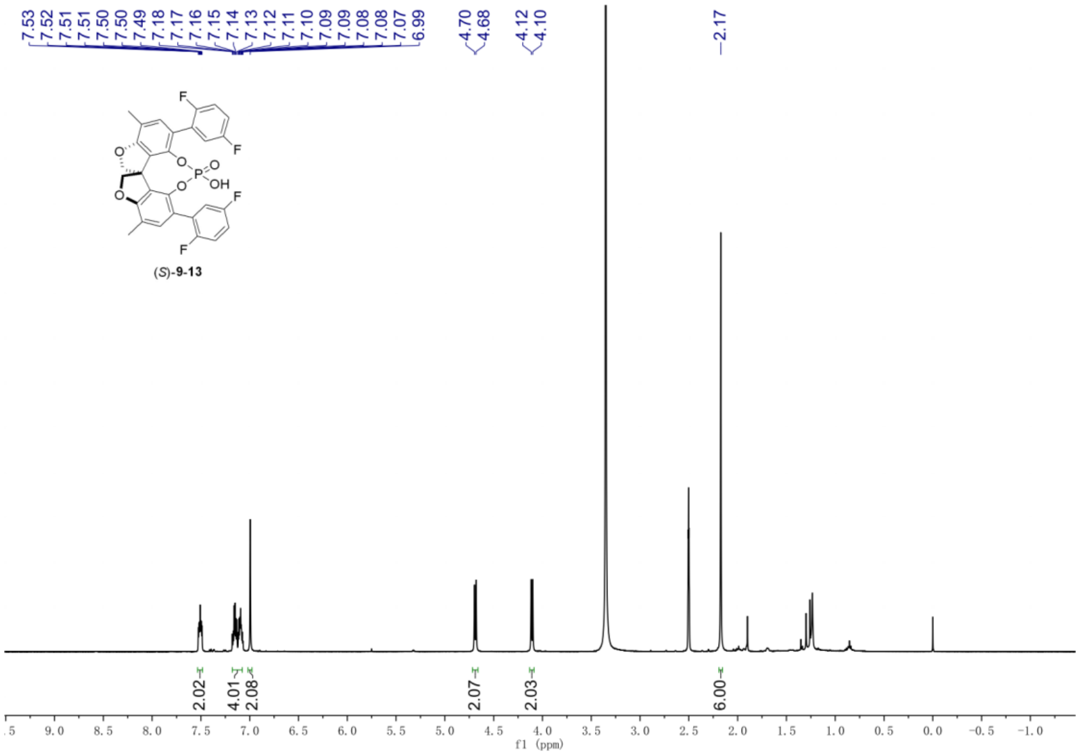

^{1}H NMR of (*S*)-**9-13** (DMSO-d_6)

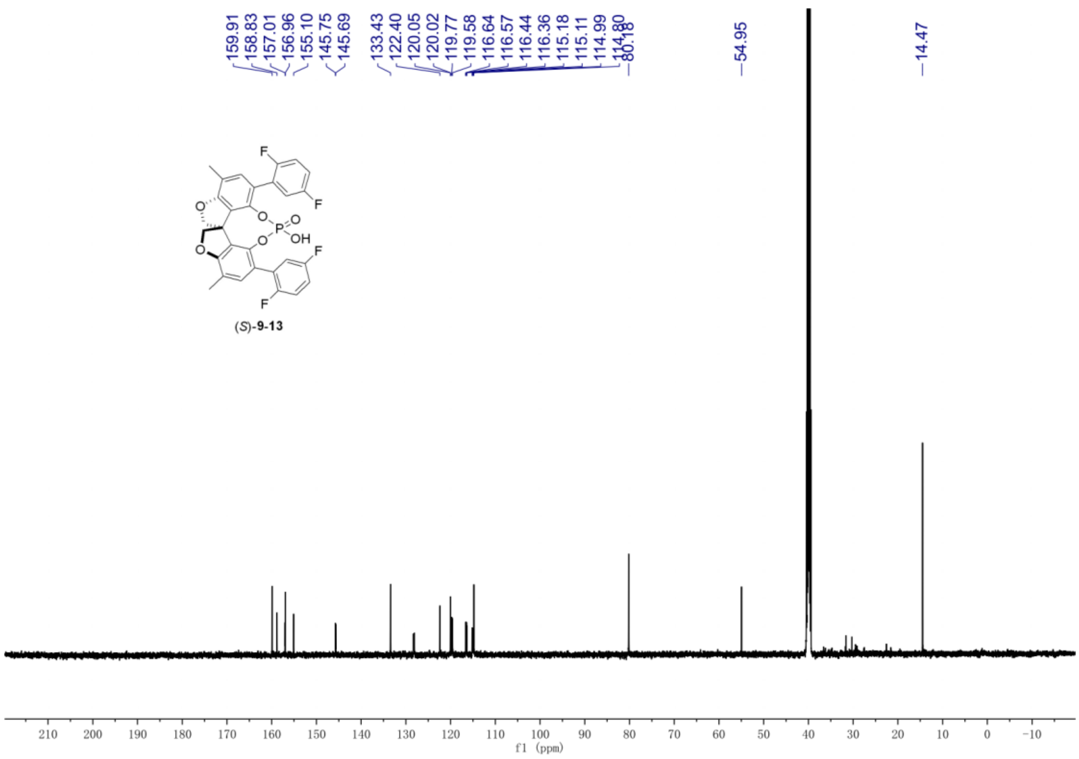

^{13}C NMR of (*S*)-**9-13** (DMSO-d_6)

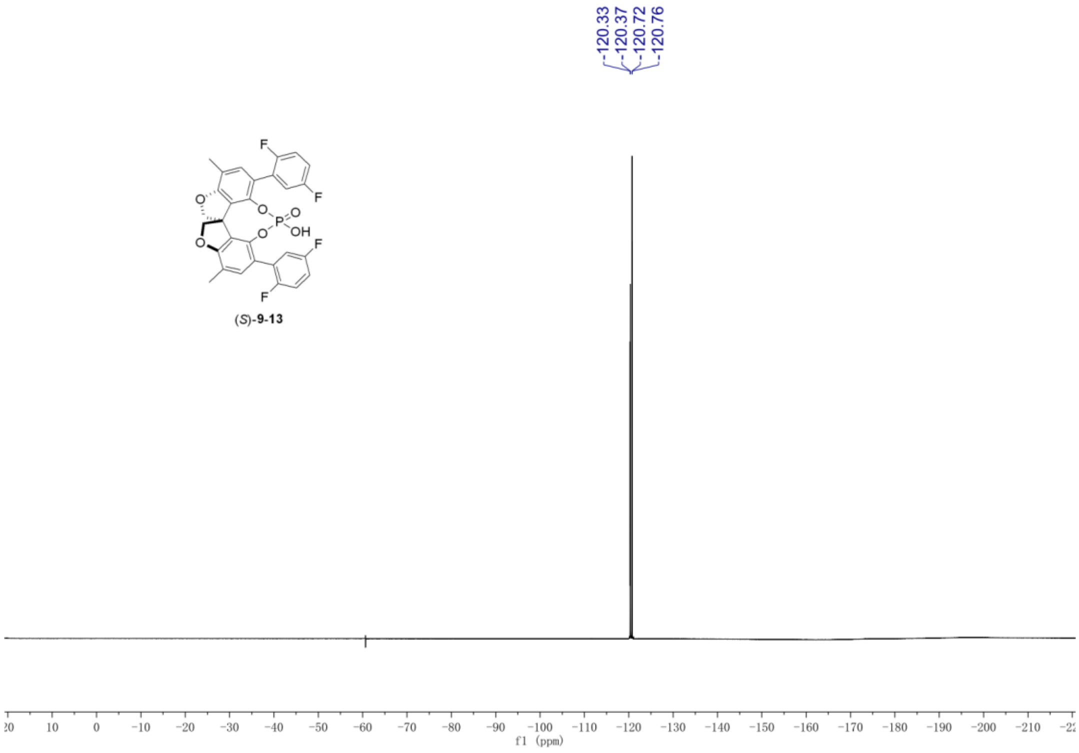

^{19}F NMR of (*S*)-**9-13** (DMSO-d_6)

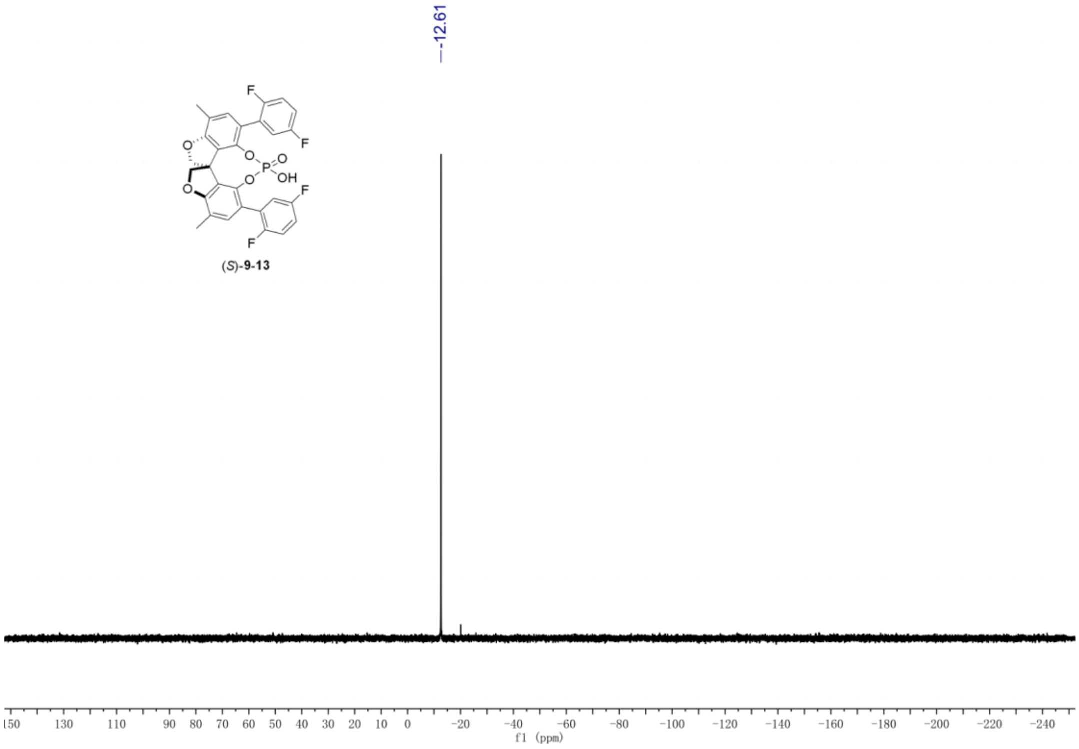

^{31}P NMR of (*S*)-**9-13** (DMSO-d_6)

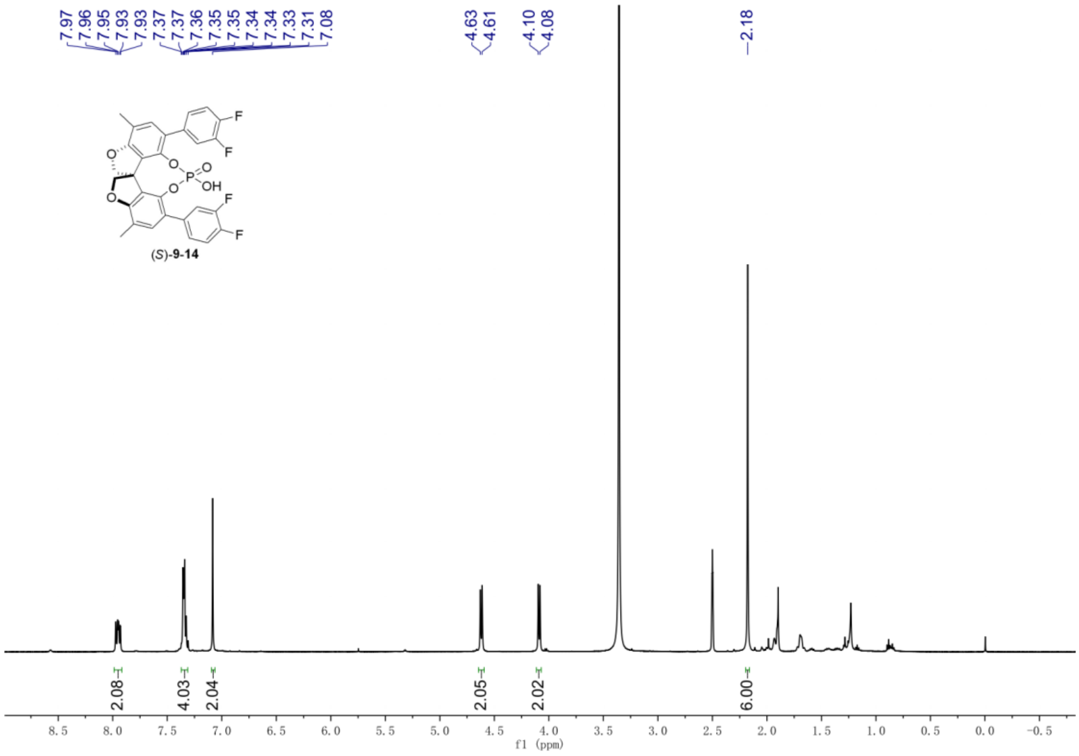

^{1}H NMR of (*S*)-**9-14** (DMSO-d_6)

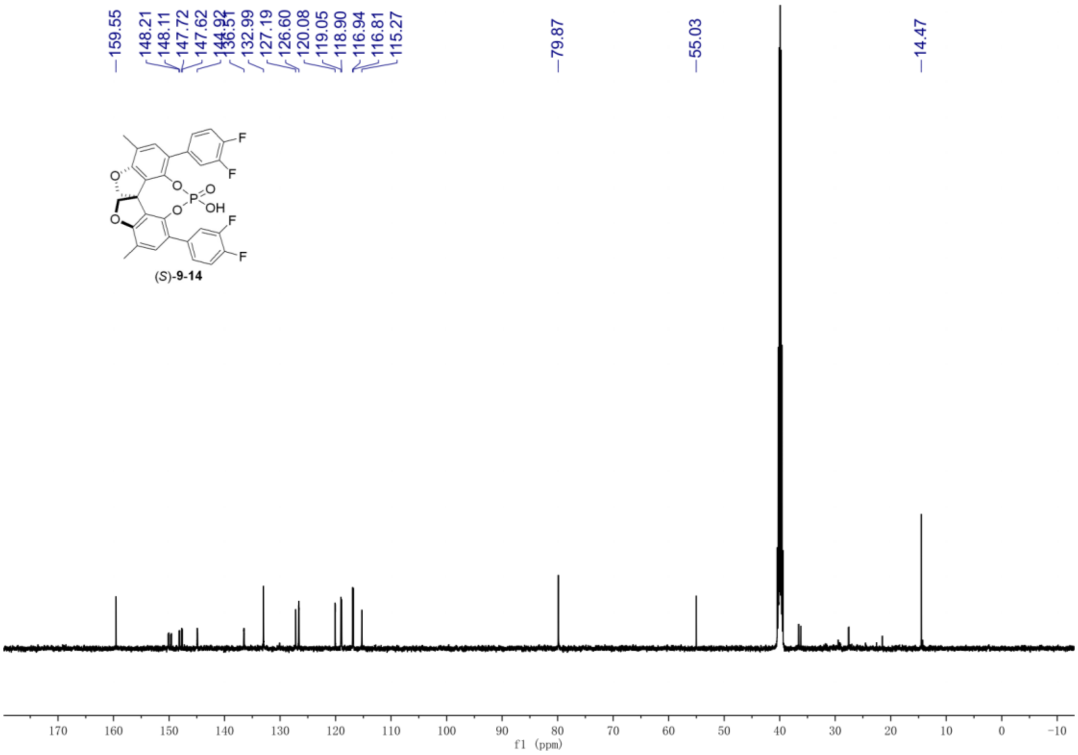

^{13}C NMR of (*S*)-**9-14** (DMSO-d_6)

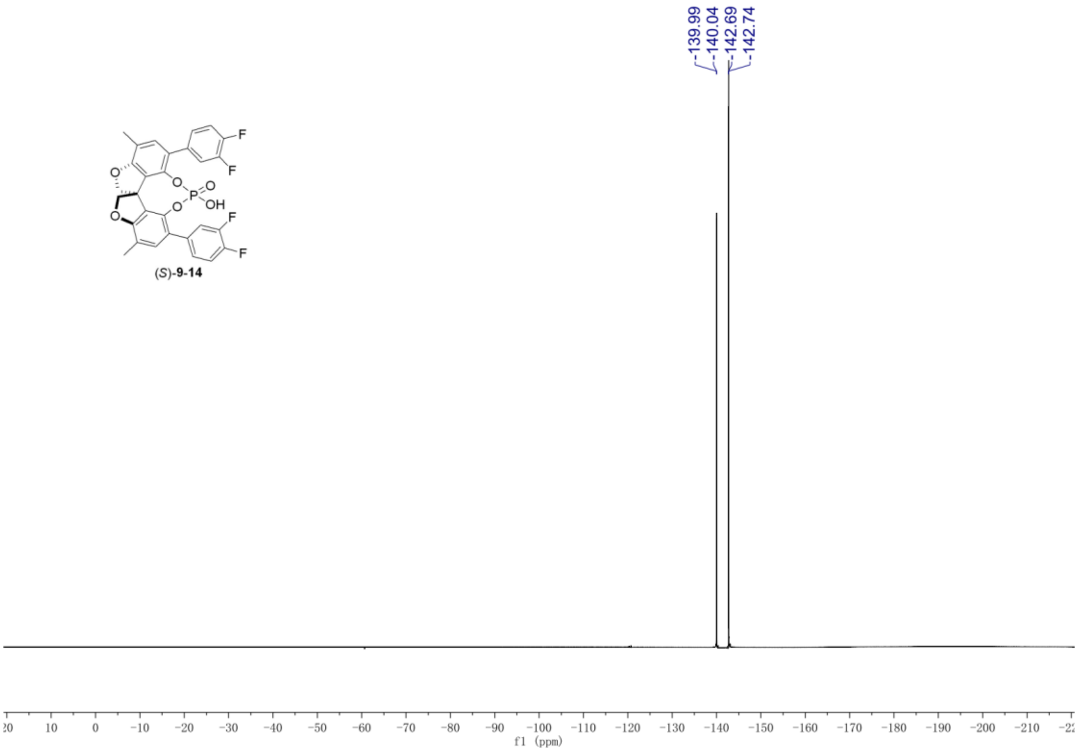

^{19}F NMR of (*S*)-**9-14** (DMSO-d_6)

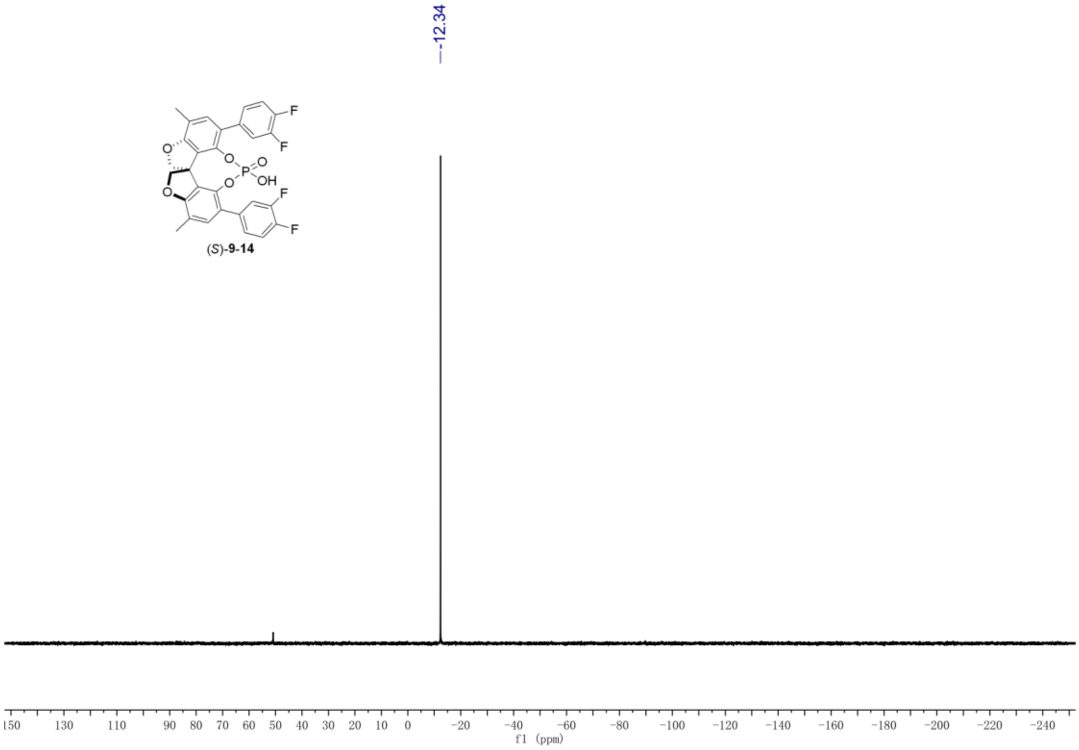

^{31}P NMR of (*S*)-**9-14** (DMSO-*d*$_6$)

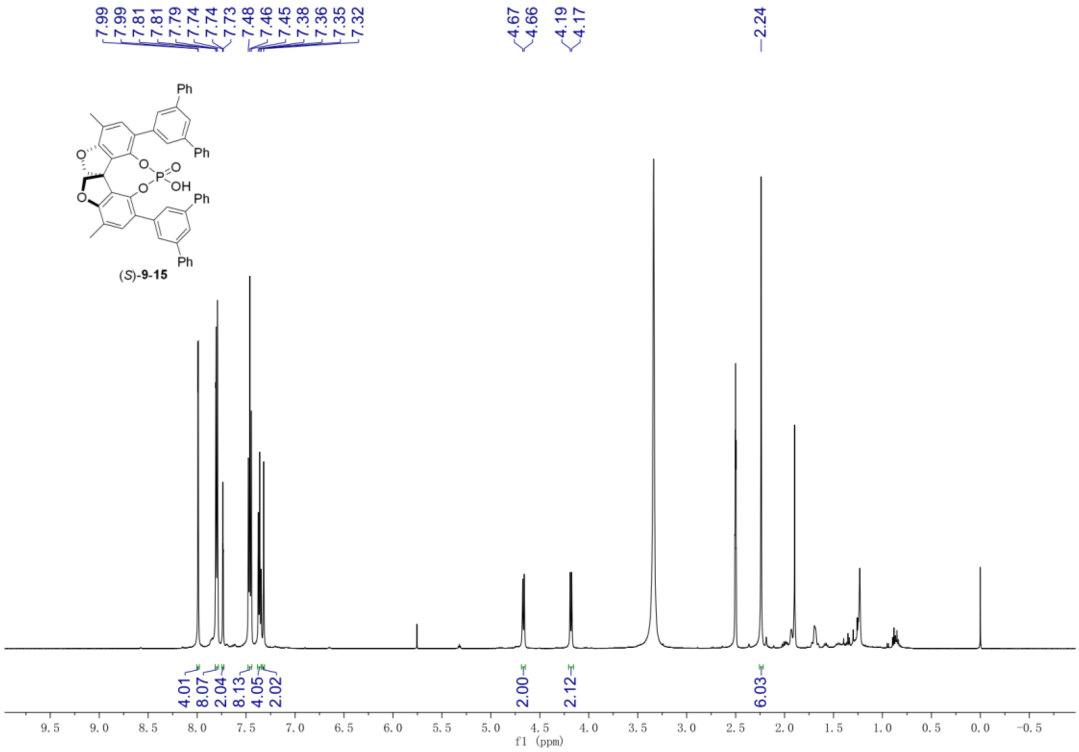

^{1}H NMR of (*S*)-**9-15** (DMSO-*d*$_6$)

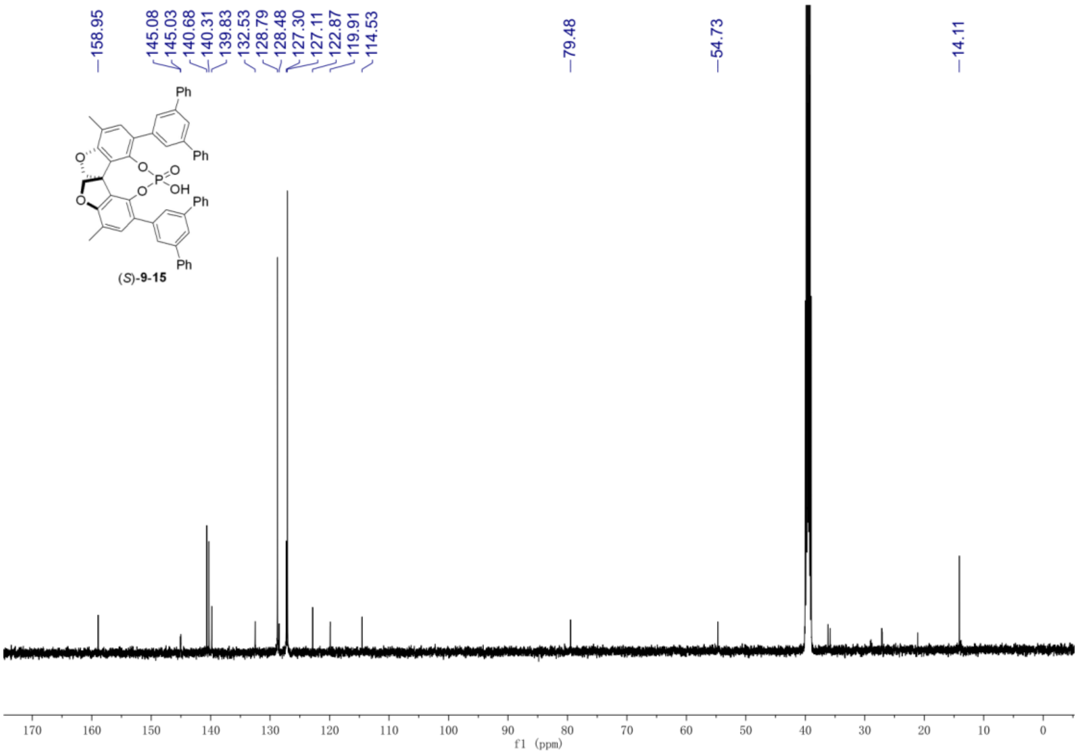

^{13}C NMR of (*S*)-**9-15** (DMSO-*d*$_6$)

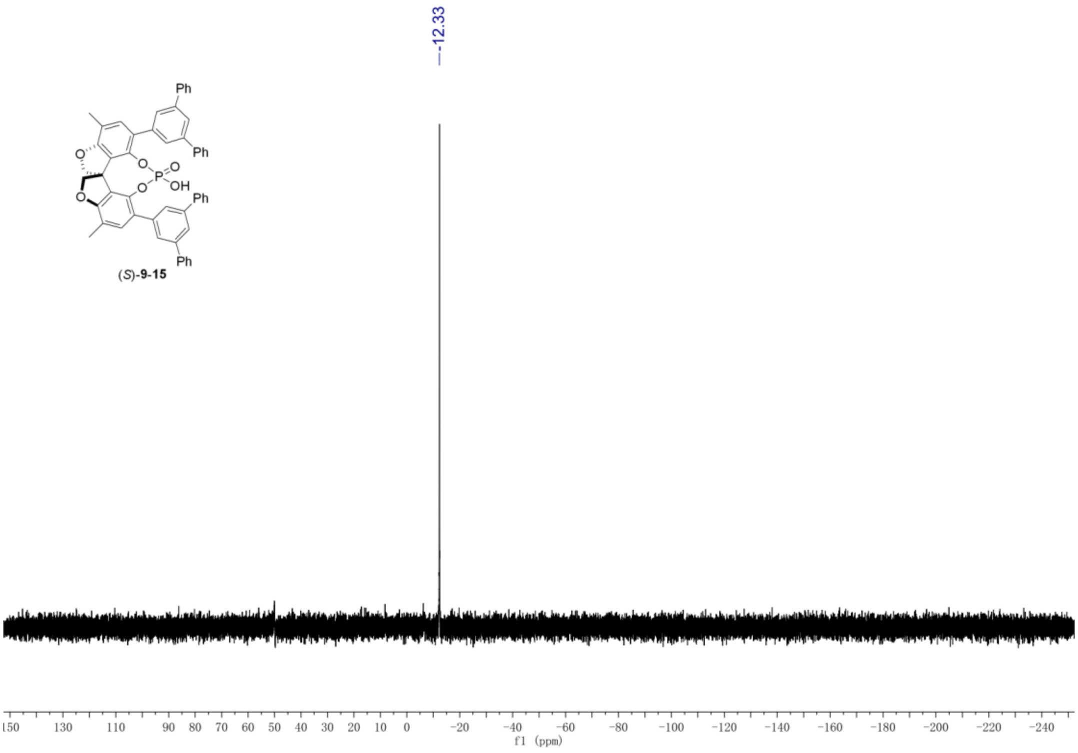

^{31}P NMR of (*S*)-**9-15** (DMSO-*d*$_6$)

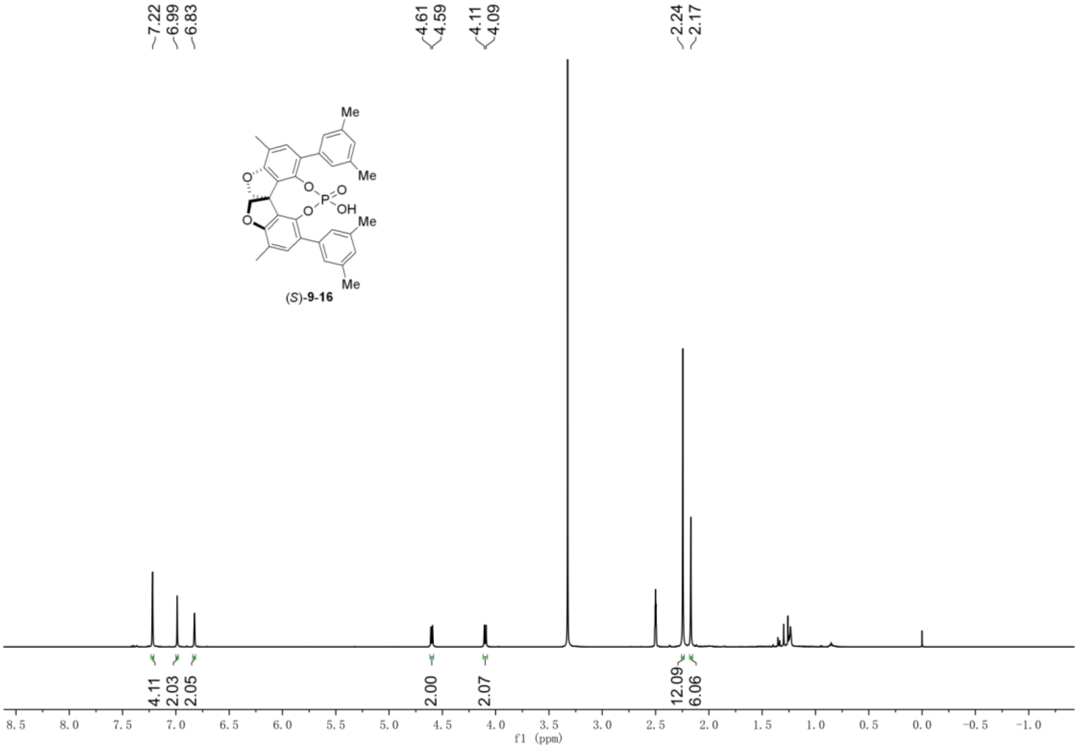

^{1}H NMR of (*S*)-**9-16** (DMSO-d_6)

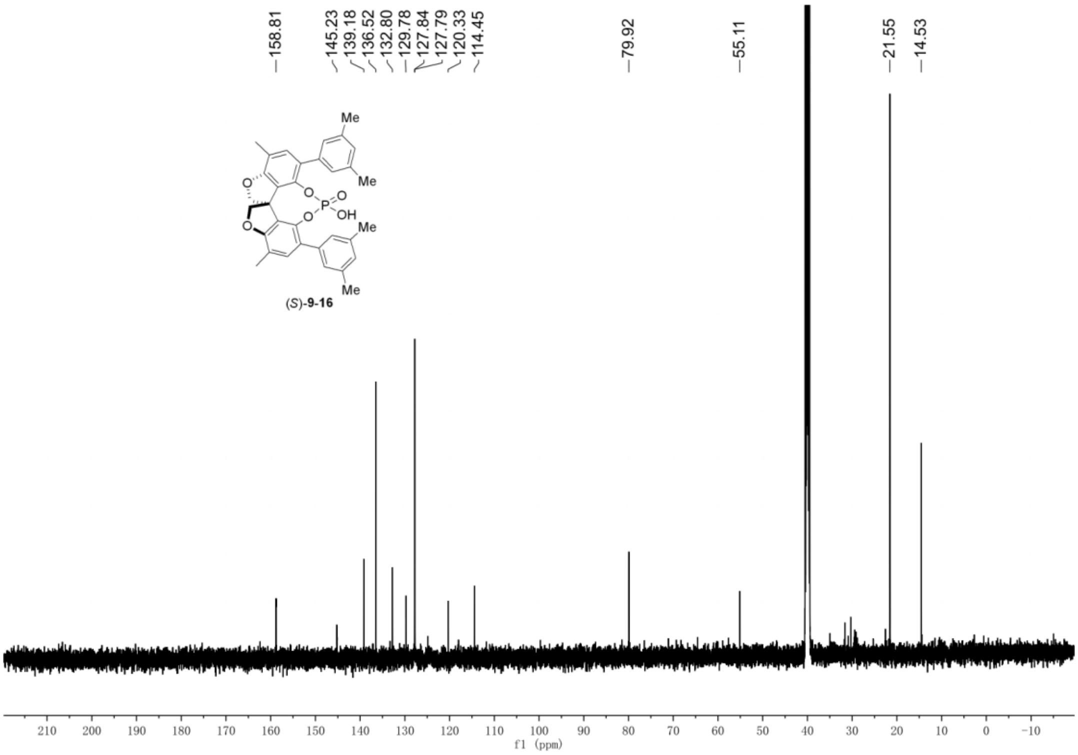

^{13}C NMR of (*S*)-**9-16** (DMSO-d_6)

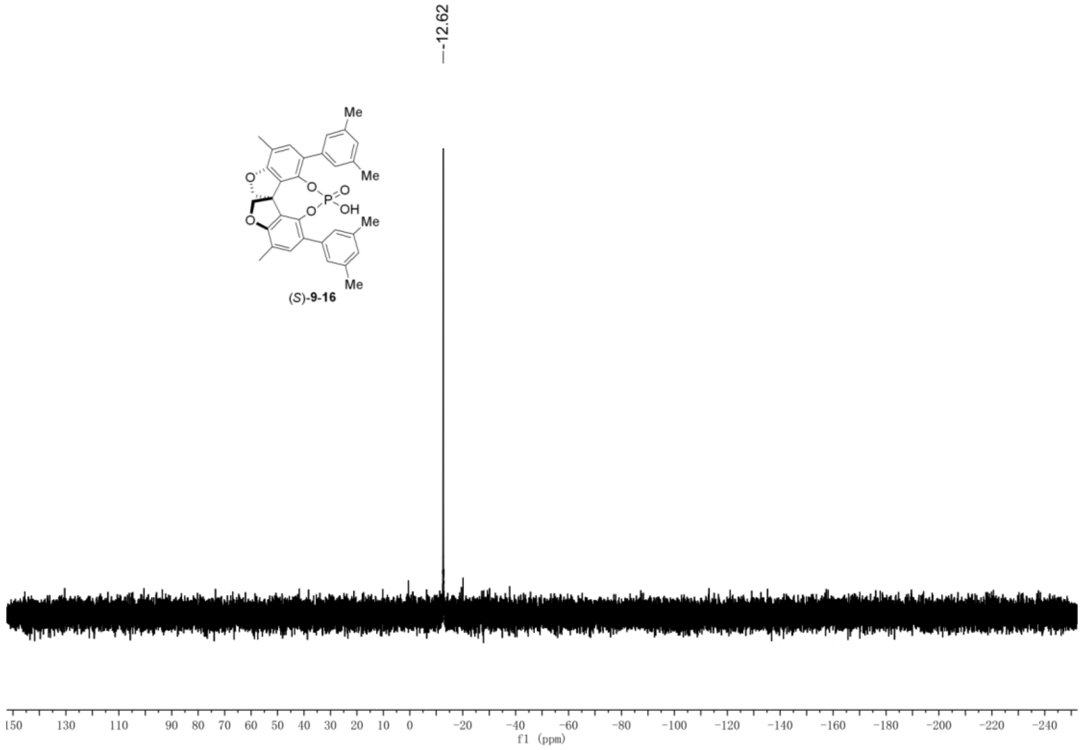

^{31}P NMR of (*S*)-**9-16** (DMSO-d_6)

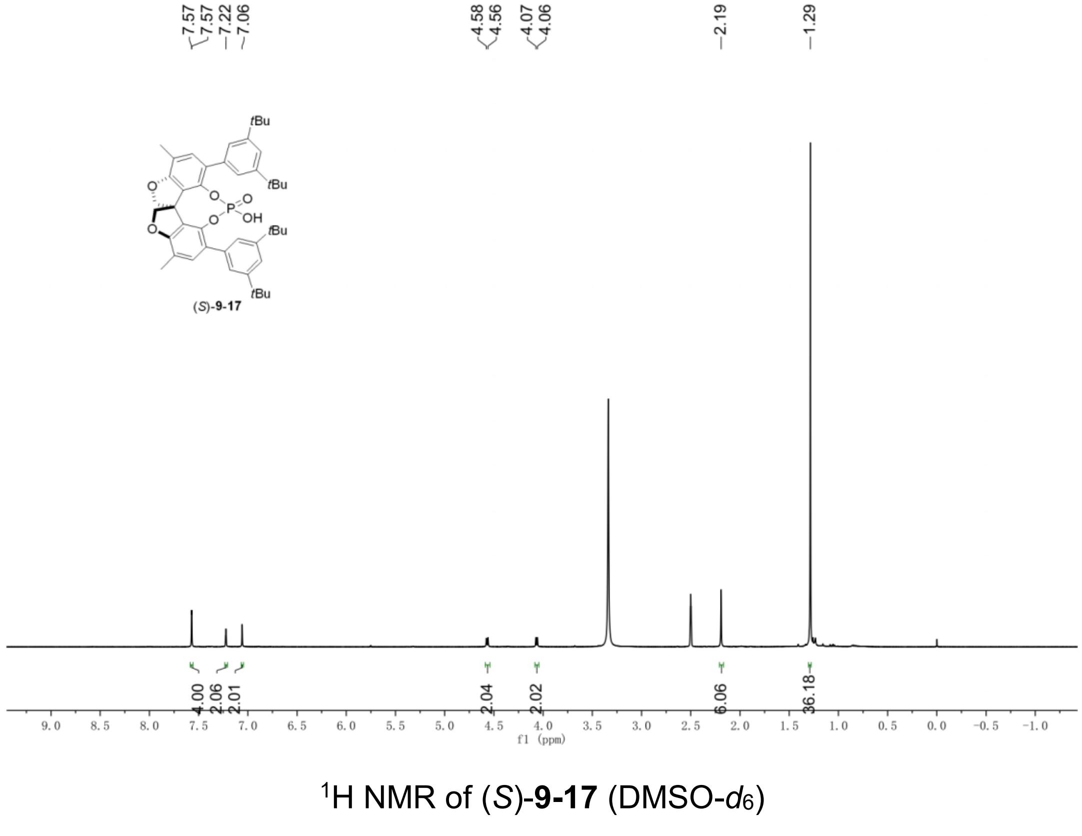

^{1}H NMR of (*S*)-**9-17** (DMSO-d_6)

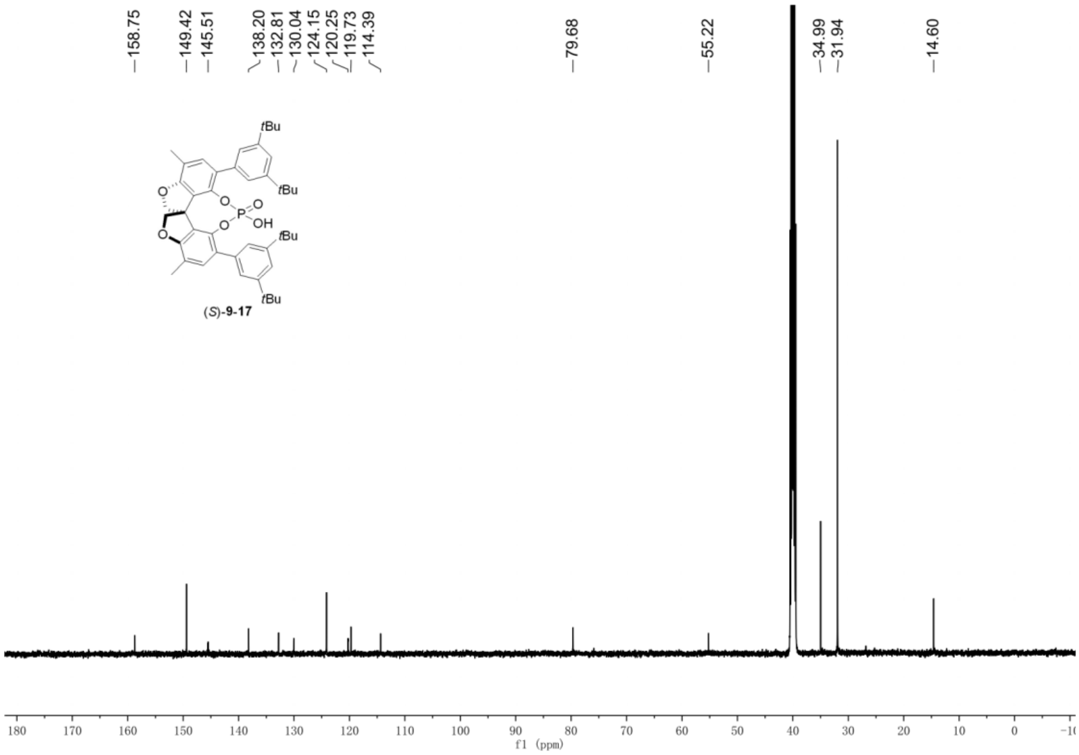

^{13}C NMR of (*S*)-**9-17** (DMSO-*d*$_6$)

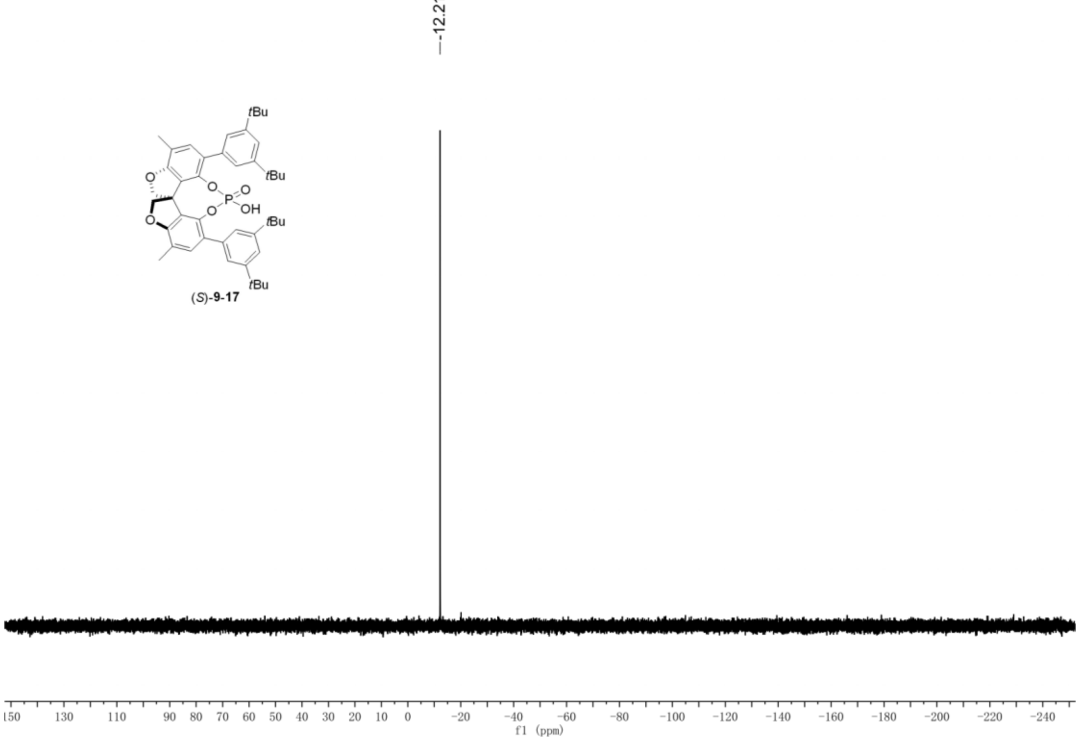

^{31}P NMR of (*S*)-**9-17** (DMSO-*d*$_6$)

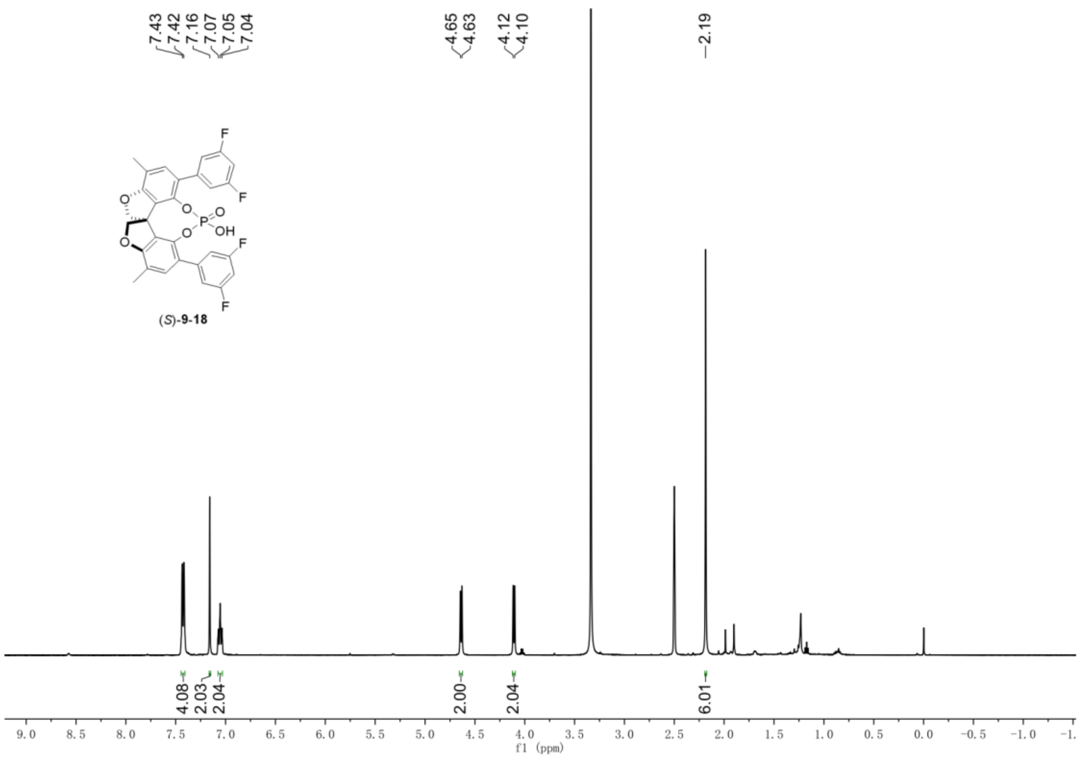

^{1}H NMR of (*S*)-**9-18** (DMSO-d_6)

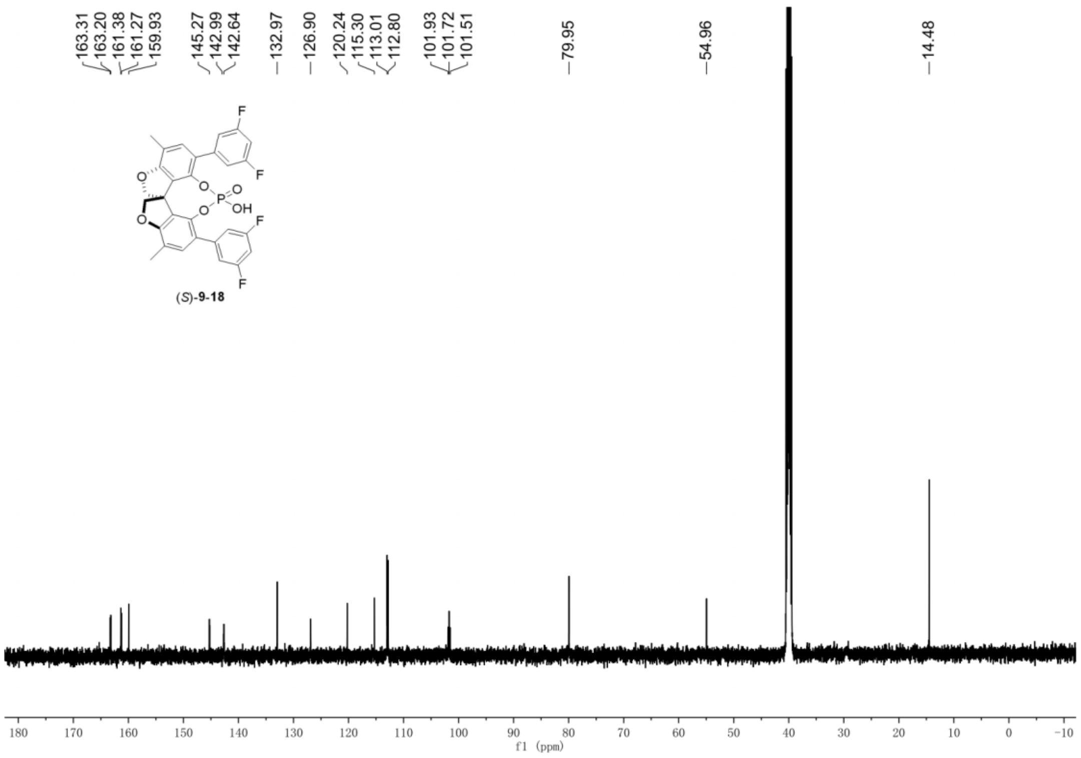

^{13}C NMR of (*S*)-**9-18** (DMSO-d_6)

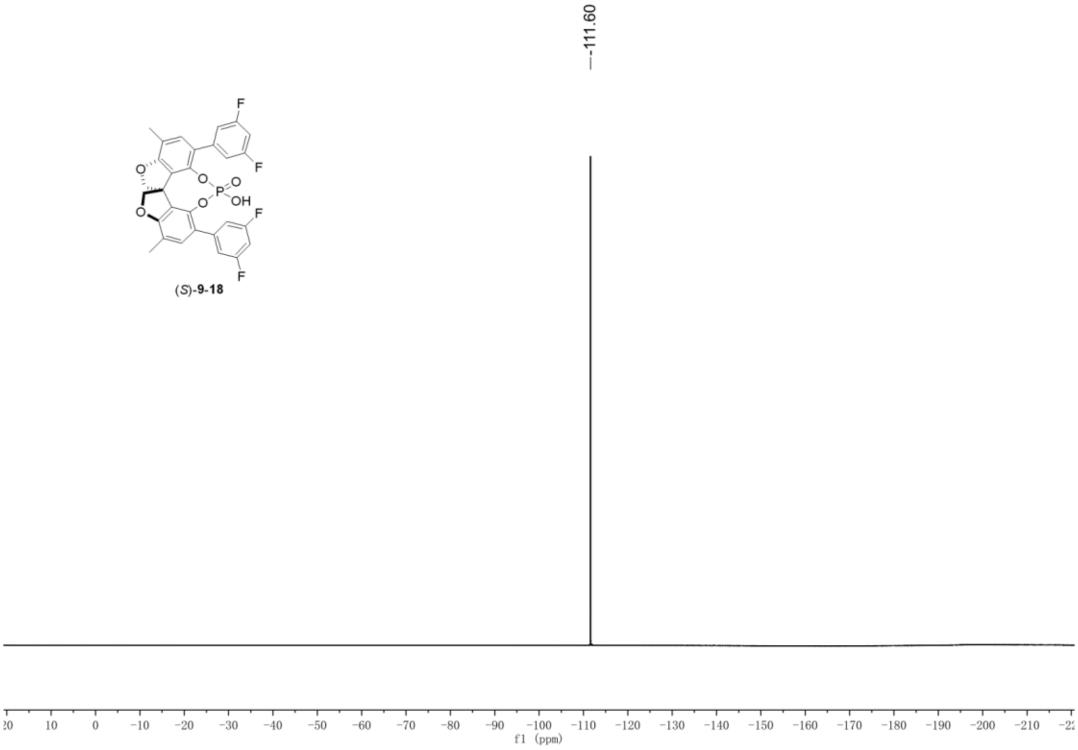

^{19}F NMR of (*S*)-**9-18** (DMSO-d_6)

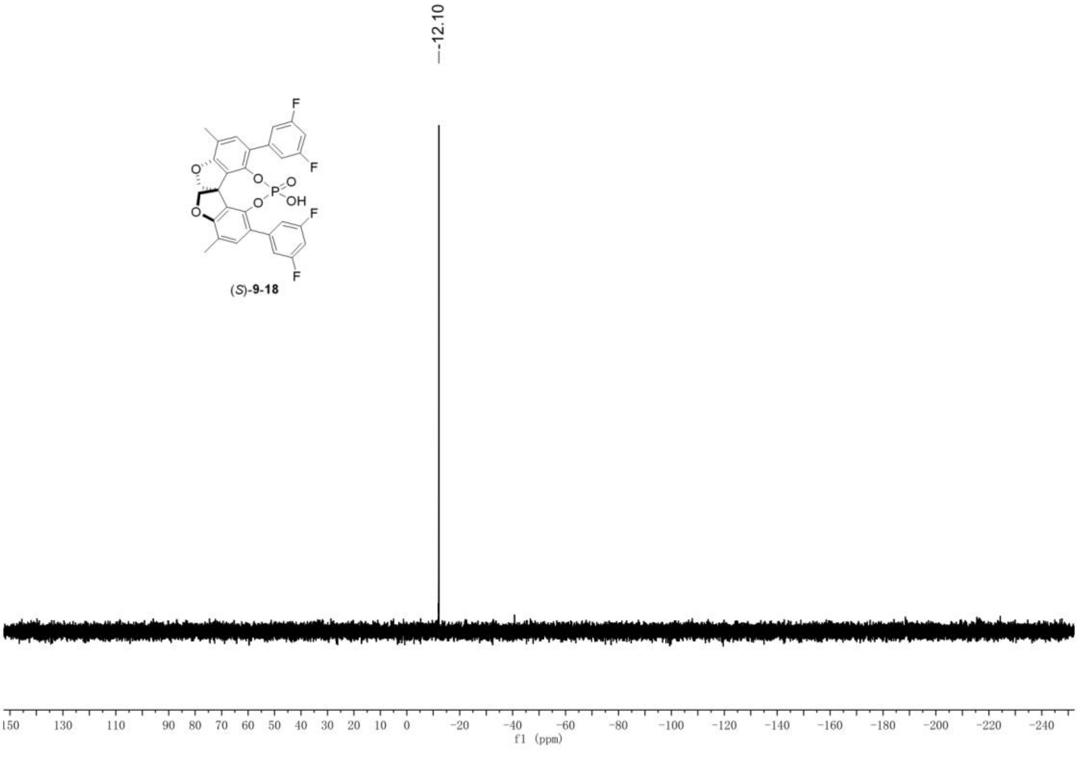

^{31}P NMR of (*S*)-**9-18** (DMSO-d_6)

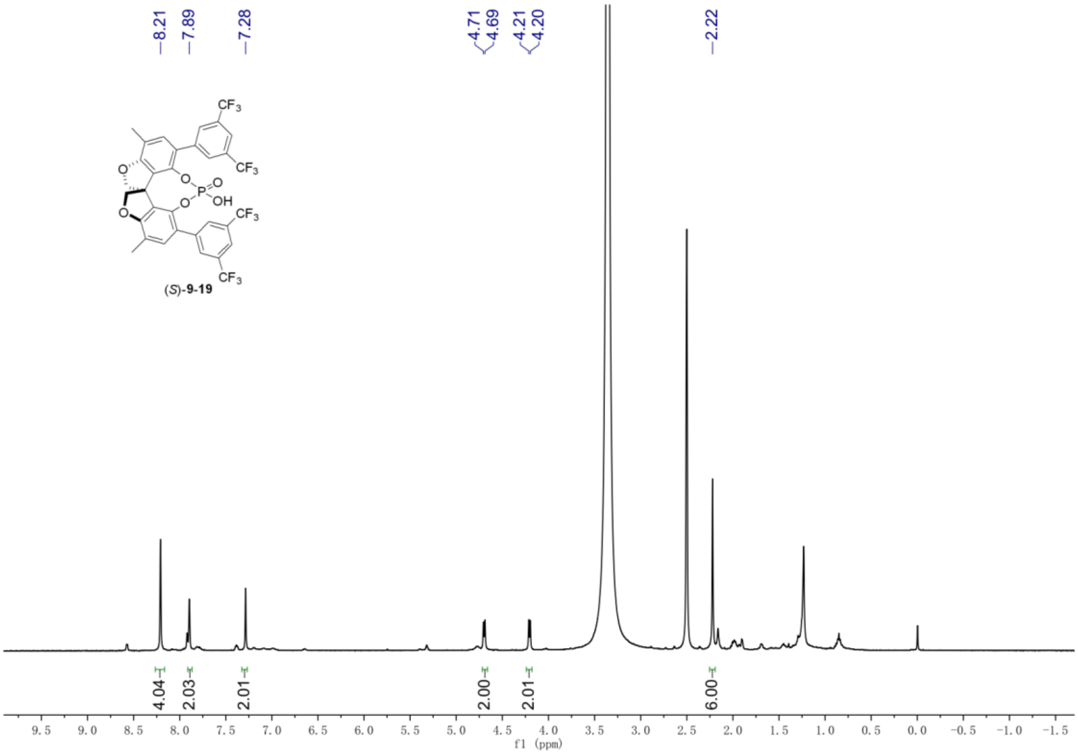

^{1}H NMR of (*S*)-**9-19** (DMSO-d_6)

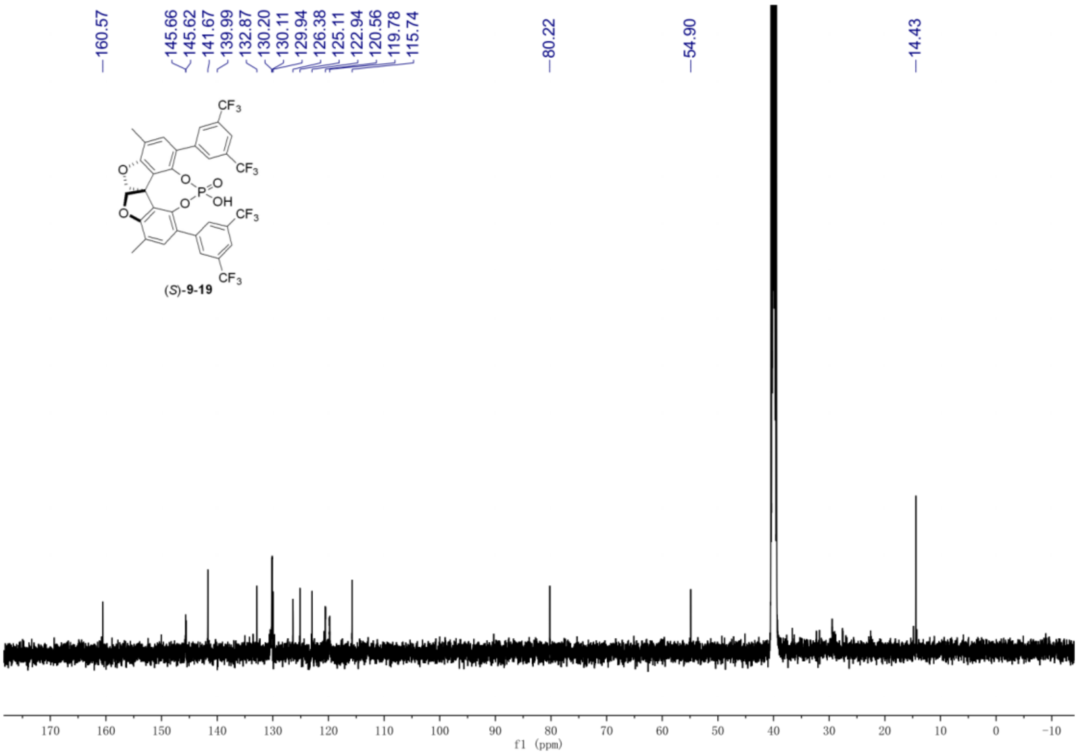

^{13}C NMR of (*S*)-**9-19** (DMSO-d_6)

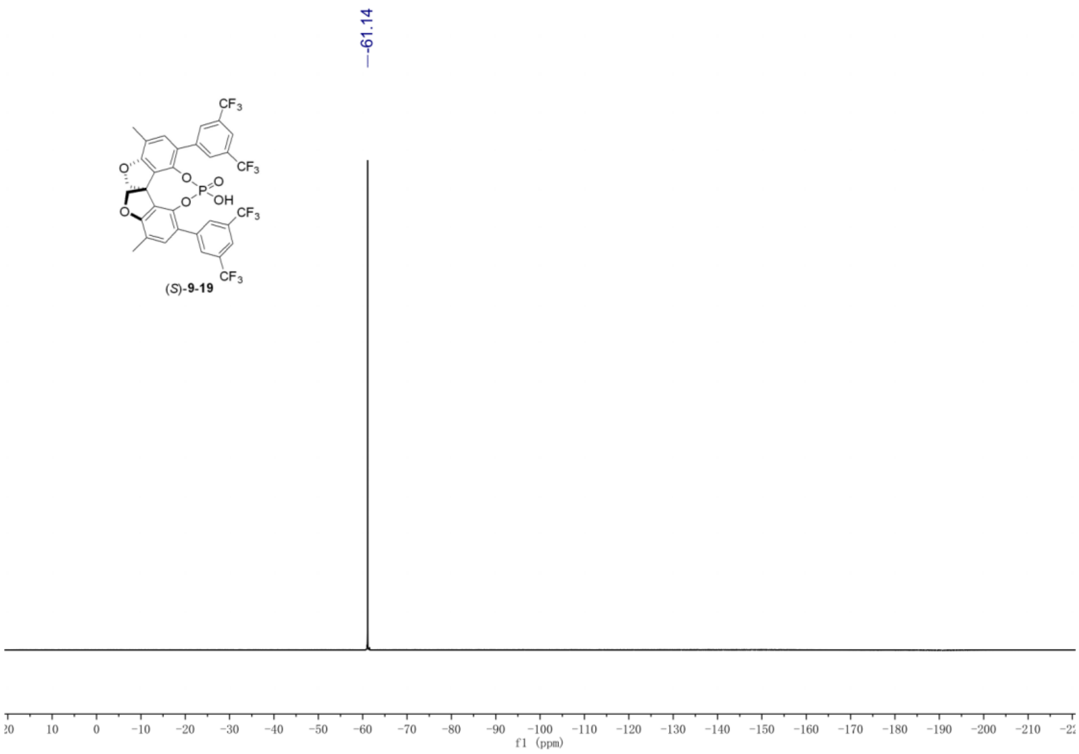

^{19}F NMR of (*S*)-**9-19** (DMSO-*d*$_6$)

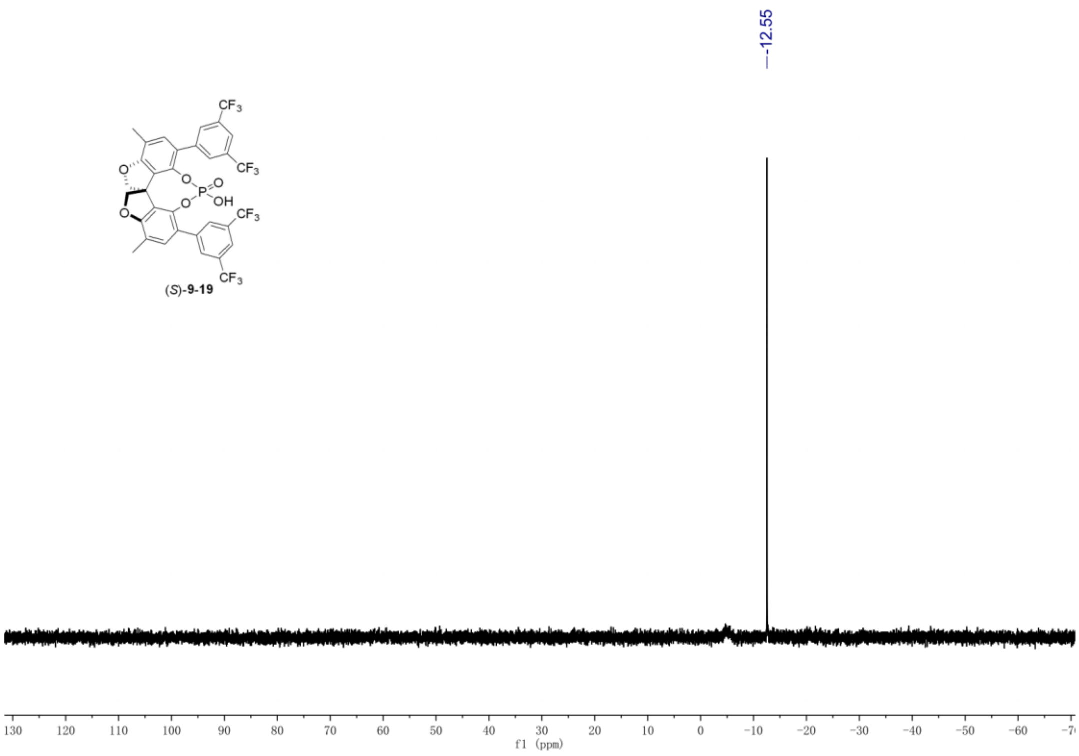

^{31}P NMR of (*S*)-**9-19** (DMSO-*d*$_6$)

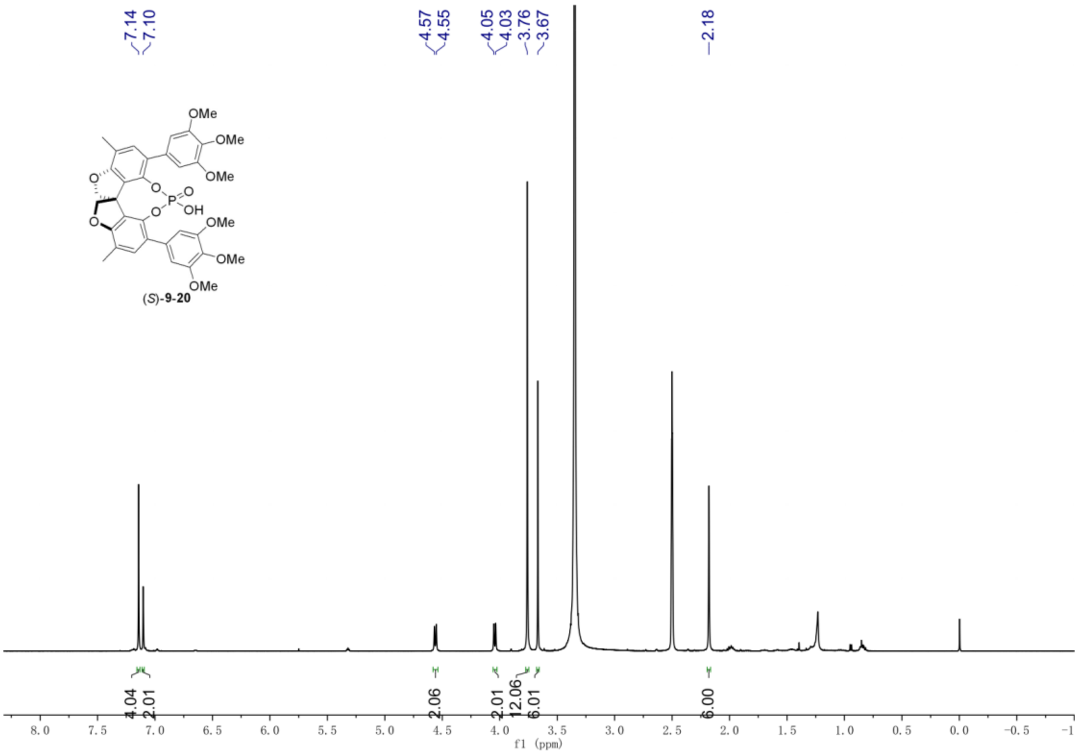

^{1}H NMR of (*S*)-**9-20** (DMSO-d_6)

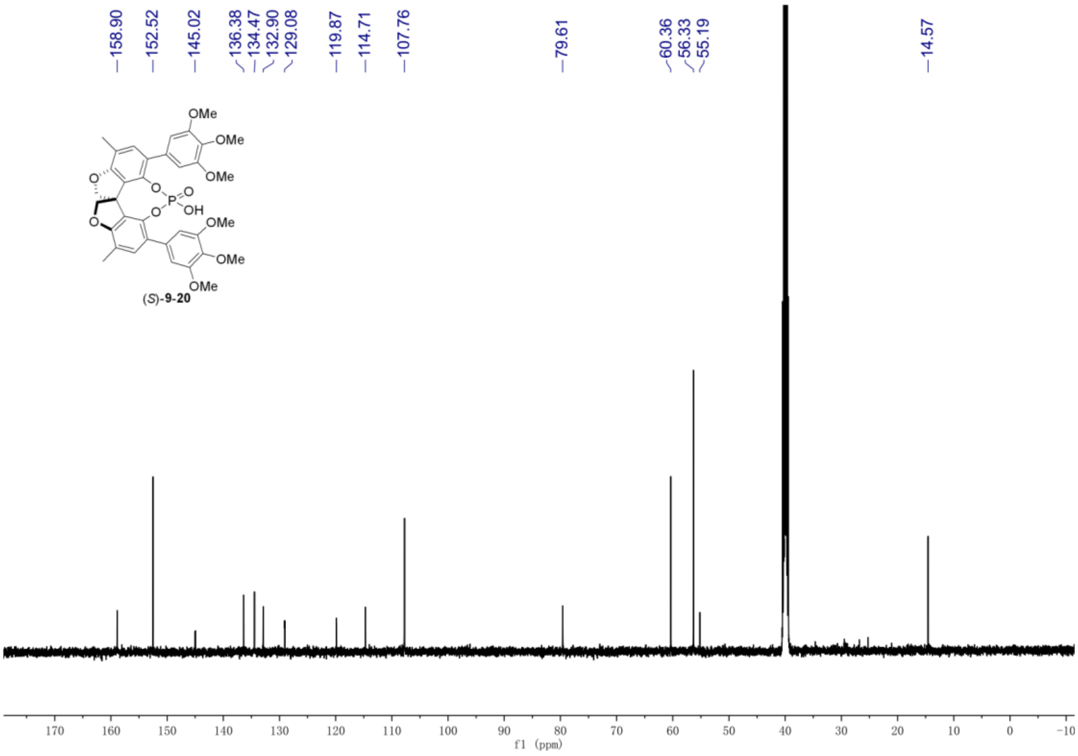

^{13}C NMR of (*S*)-**9-20** (DMSO-d_6)

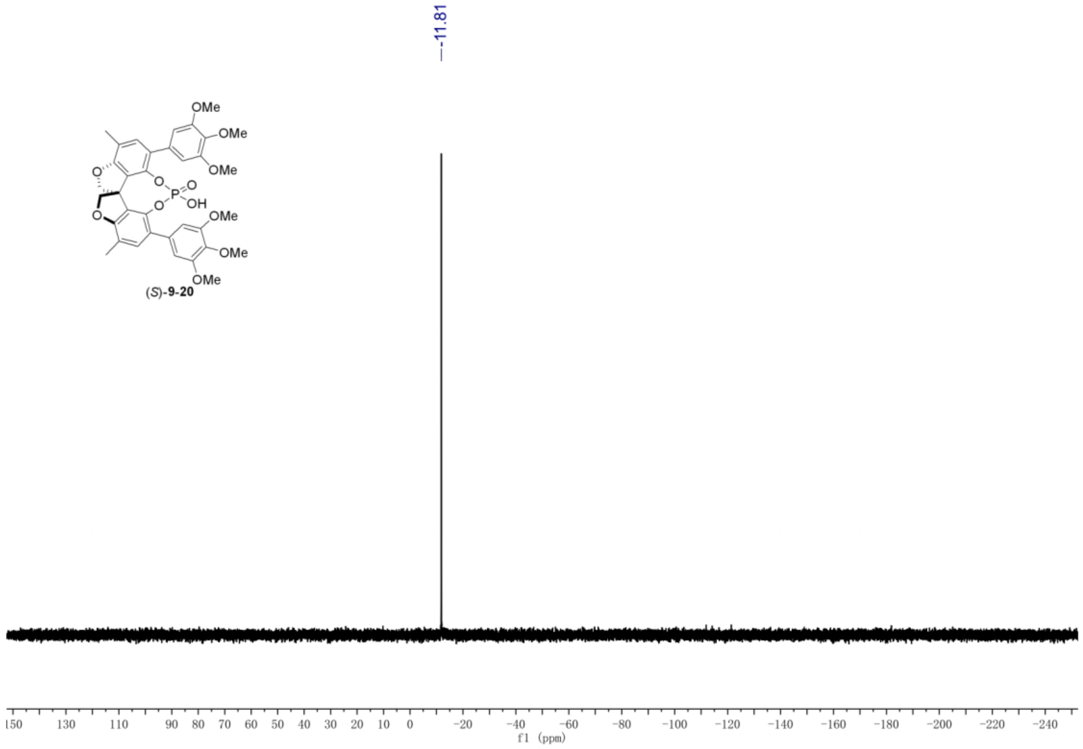

^{31}P NMR of (*S*)-**9-20** (DMSO-d_6)

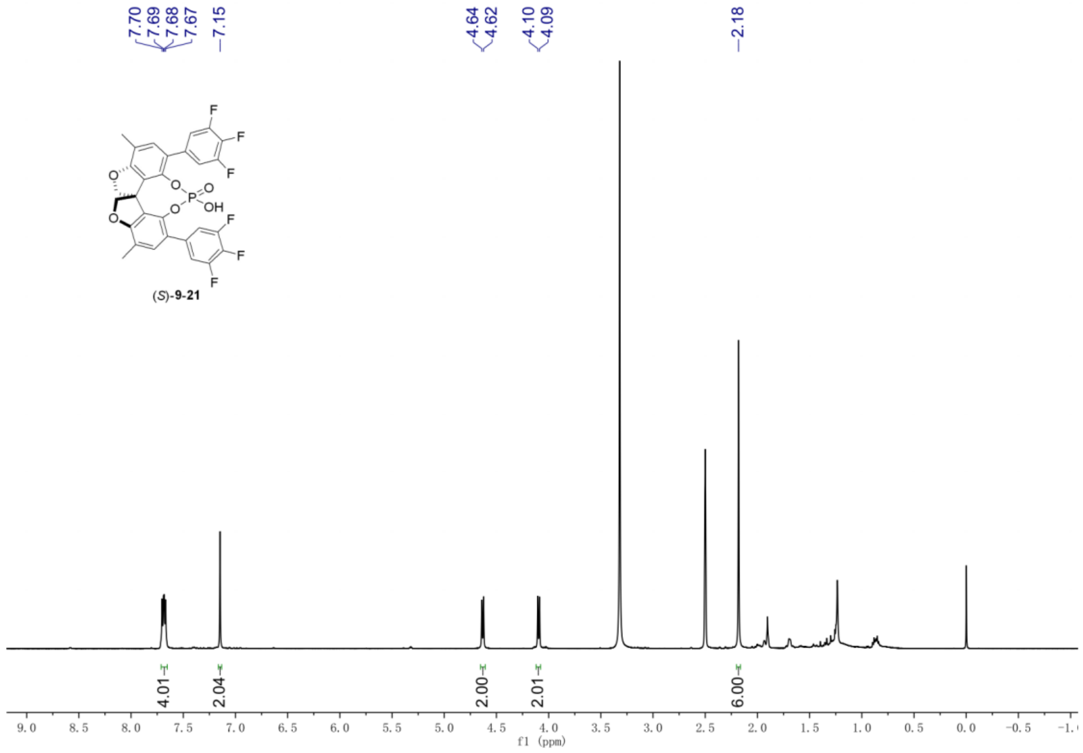

^{1}H NMR of (*S*)-**9-21** (DMSO-d_6)

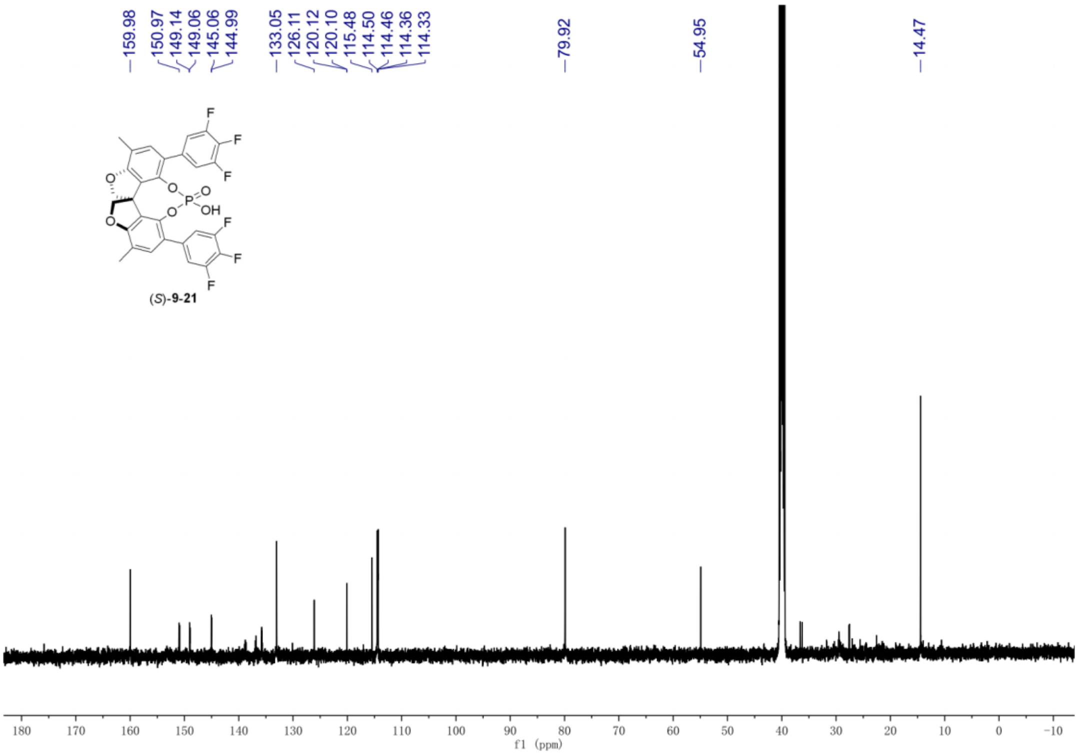

^{13}C NMR of (*S*)-**9-21** (DMSO-*d*$_6$)

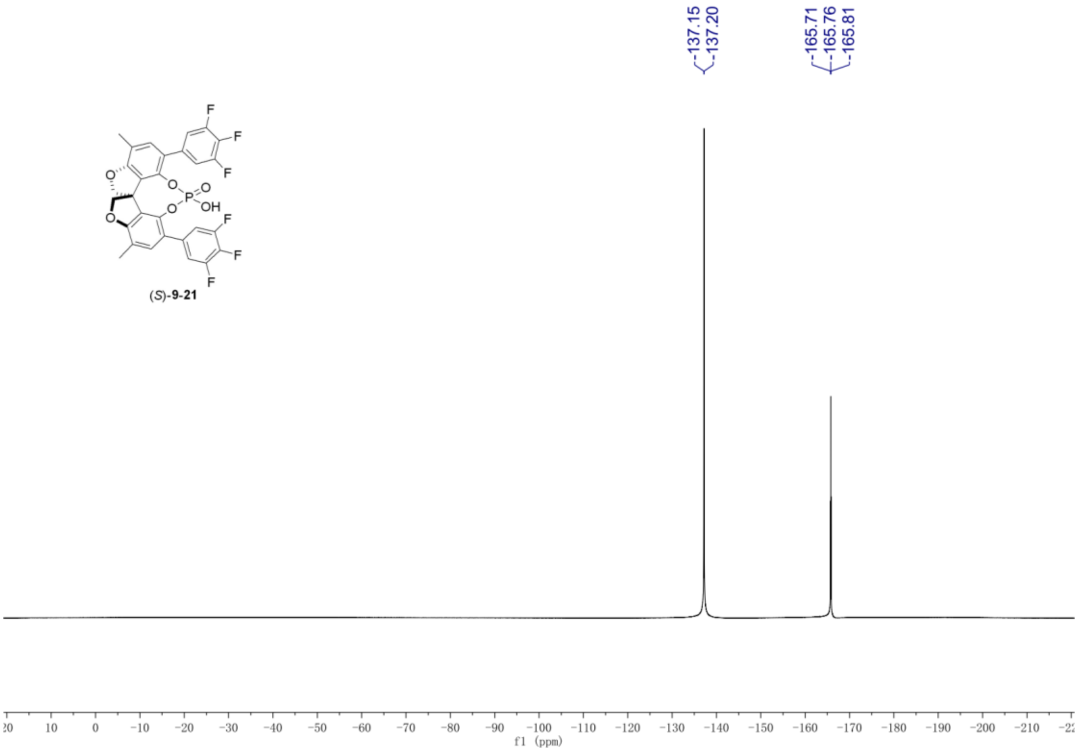

^{19}F NMR of (*S*)-**9-21** (DMSO-*d*$_6$)

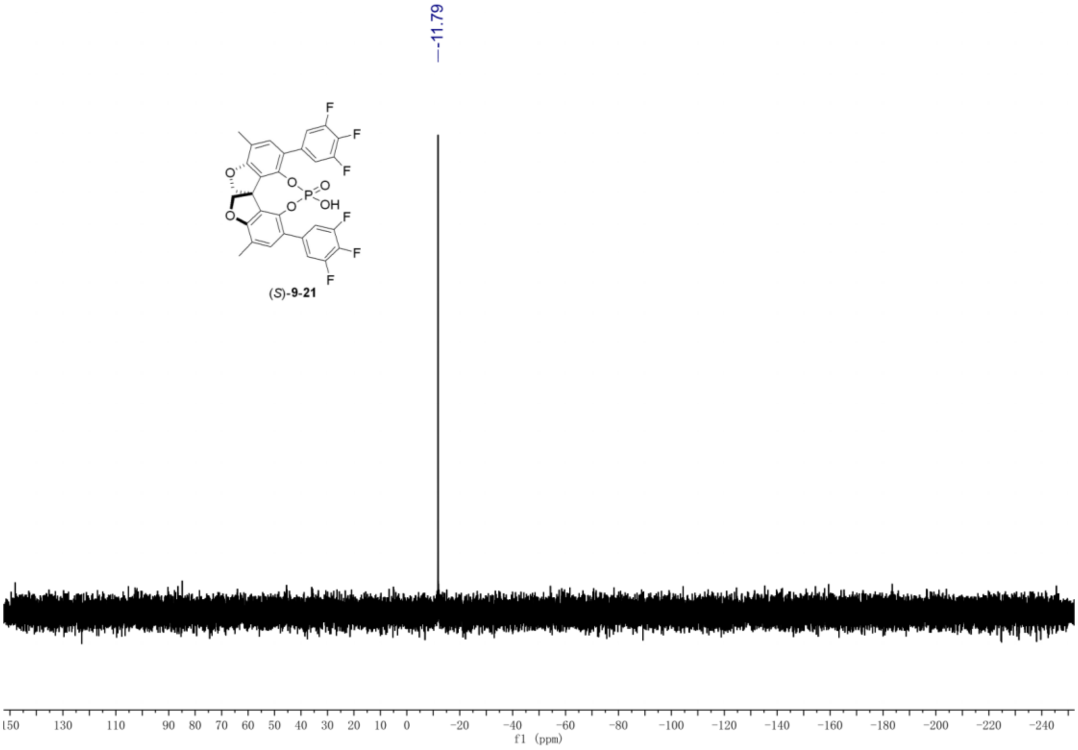

^{31}P NMR of (*S*)-**9-21** (DMSO-d_6)

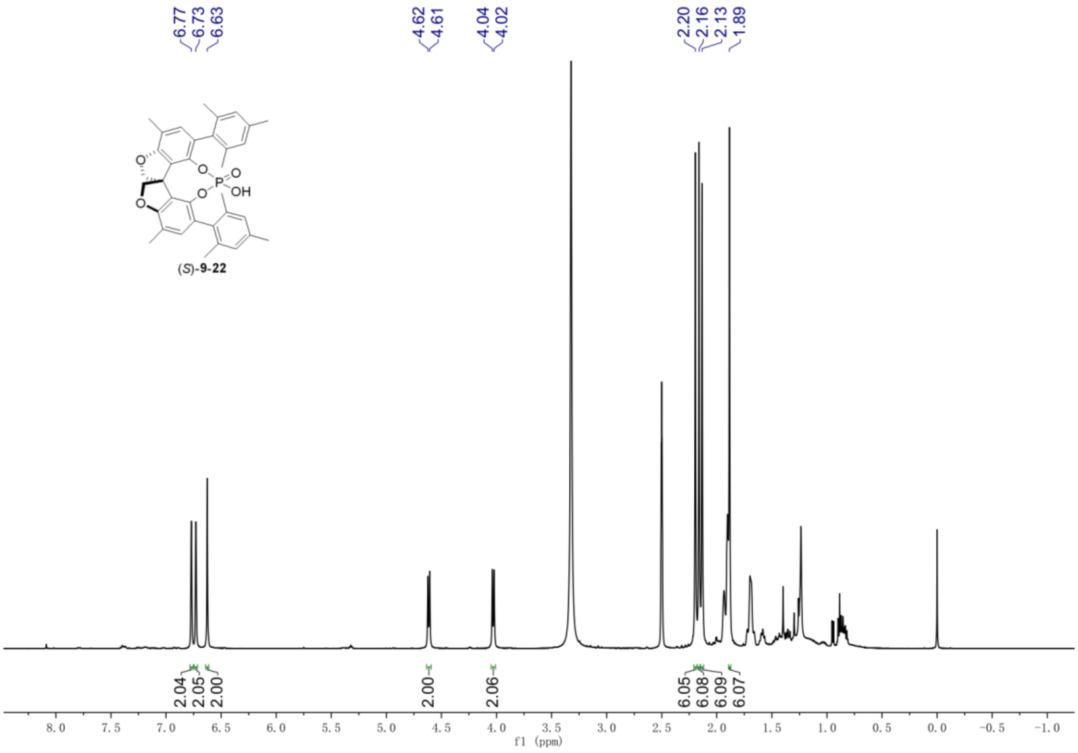

^{1}H NMR of (*S*)-**9-22** (DMSO-d_6)

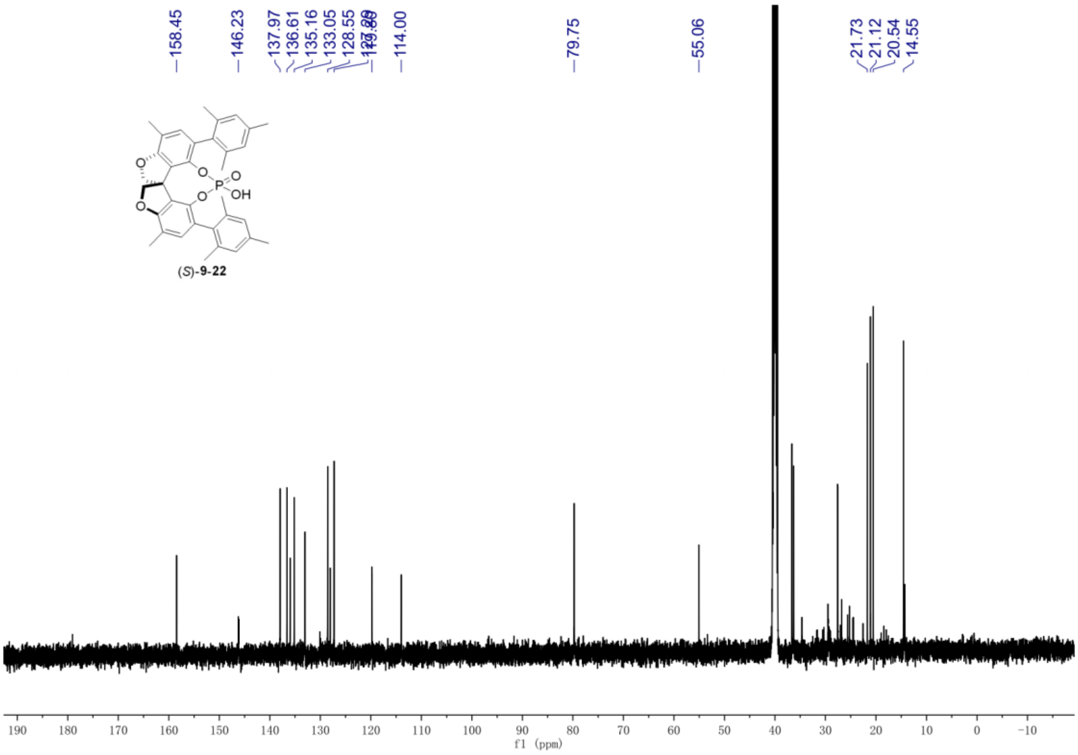

^{13}C NMR of (*S*)-**9-22** (DMSO-*d*6)

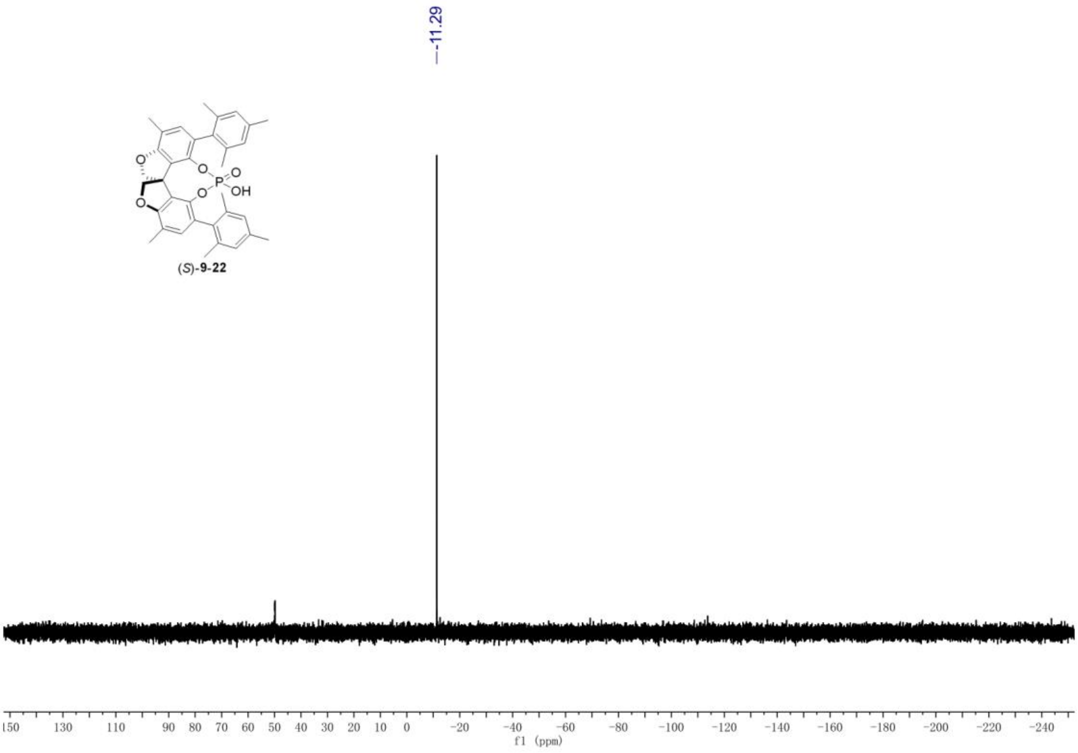

^{31}P NMR of (*S*)-**9-22** (DMSO-*d*6)

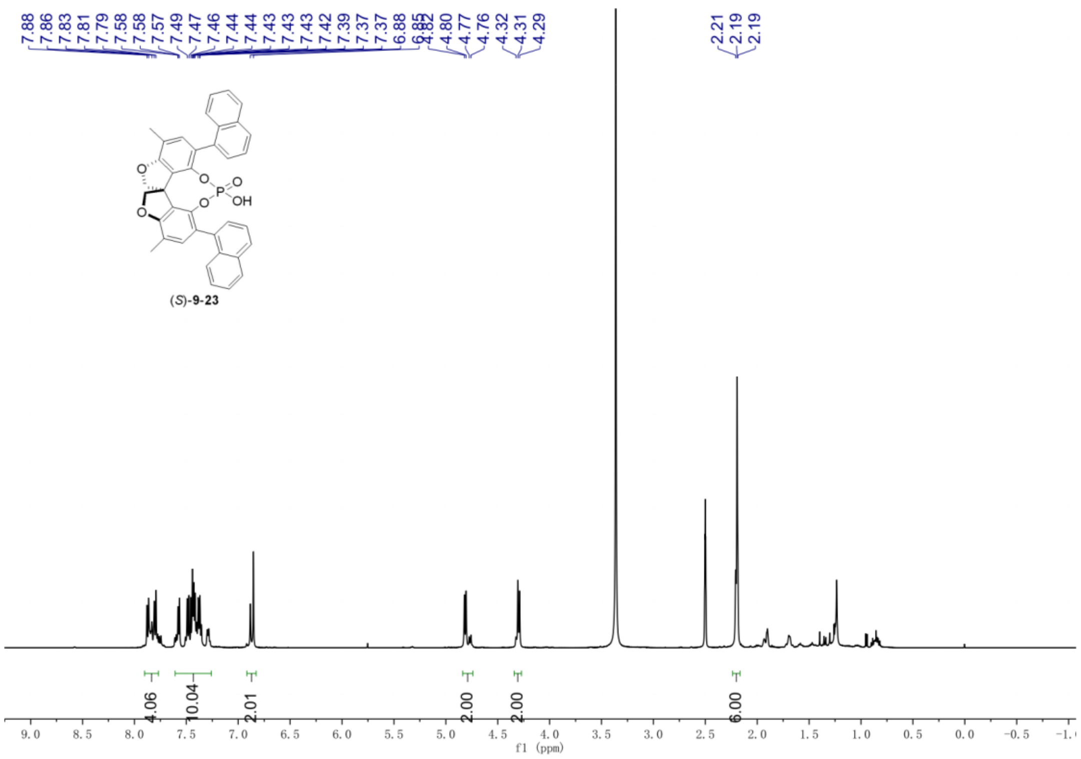

^{1}H NMR of (*S*)-**9-23** (DMSO-d_6)

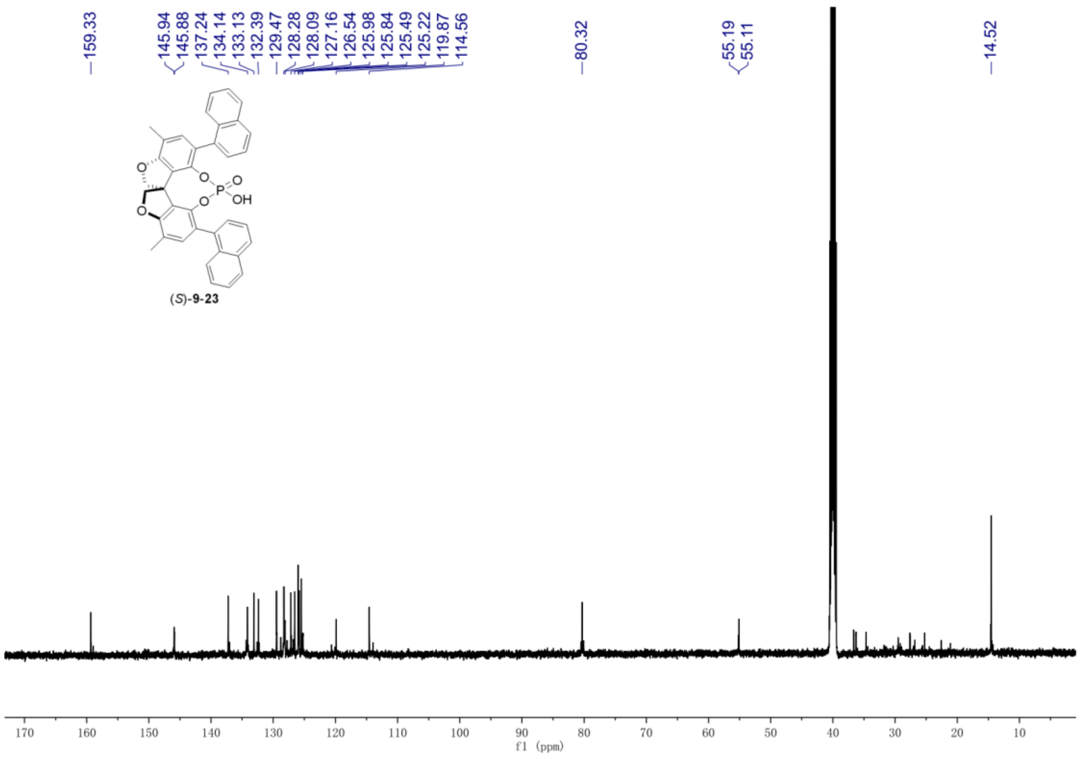

^{13}C NMR of (*S*)-**9-23** (DMSO-d_6)

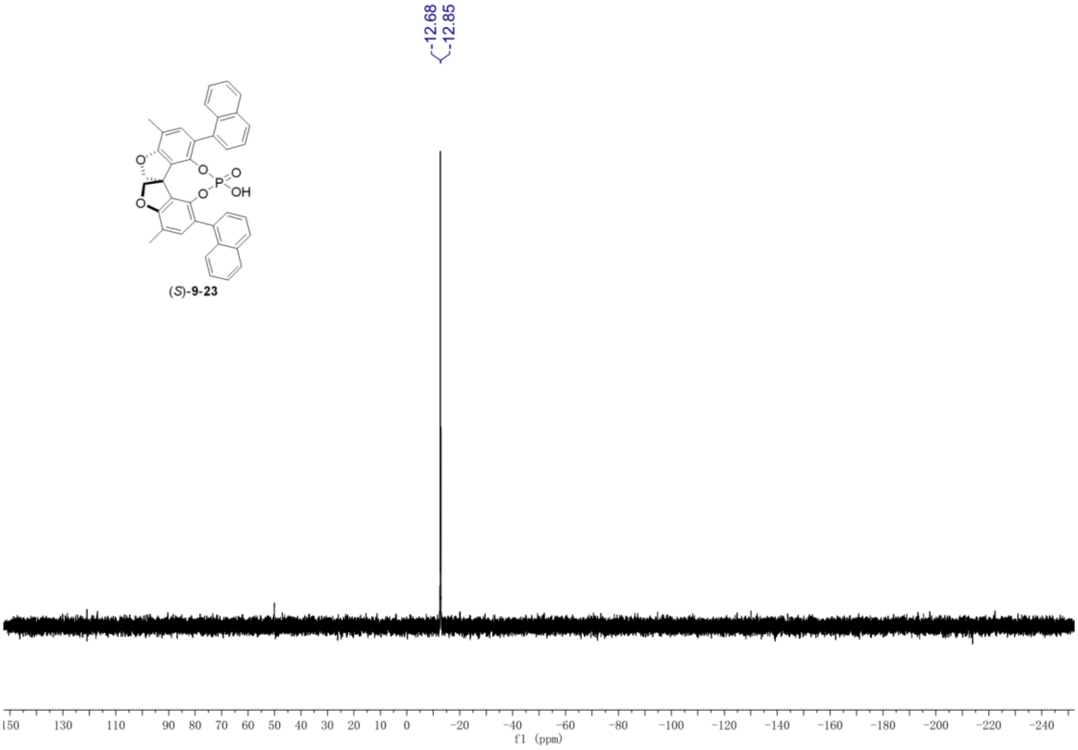

^{31}P NMR of (*S*)-**9-23** (DMSO-d_6)

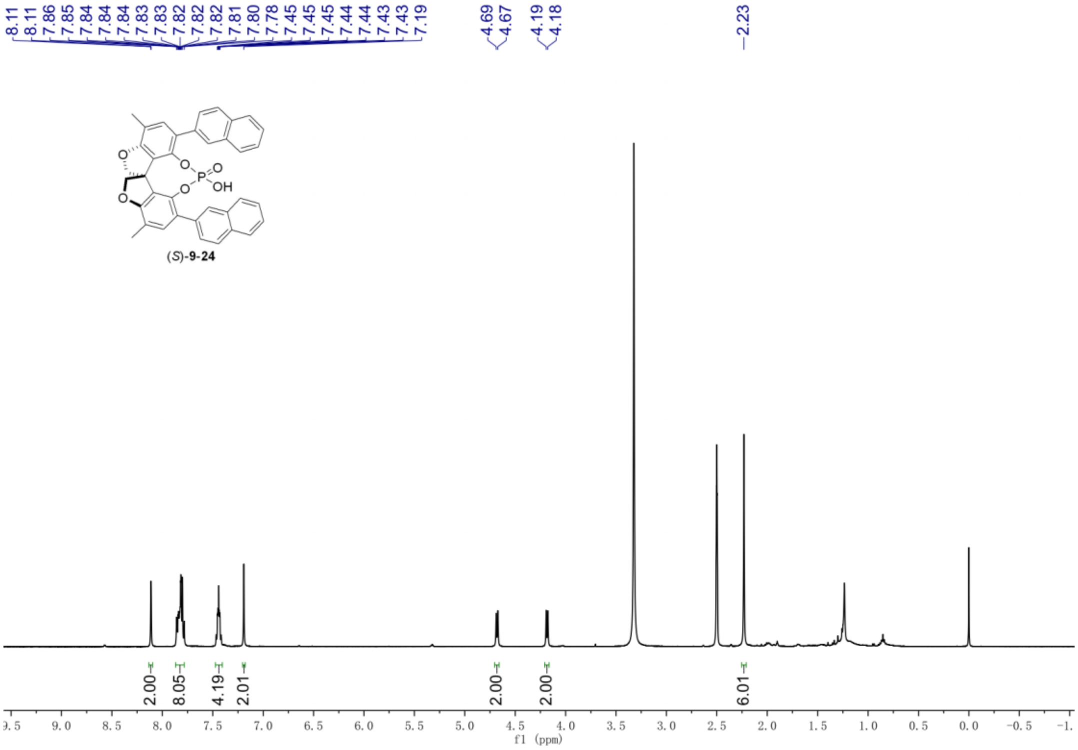

^{1}H NMR of (*S*)-**9-24** (DMSO-d_6)

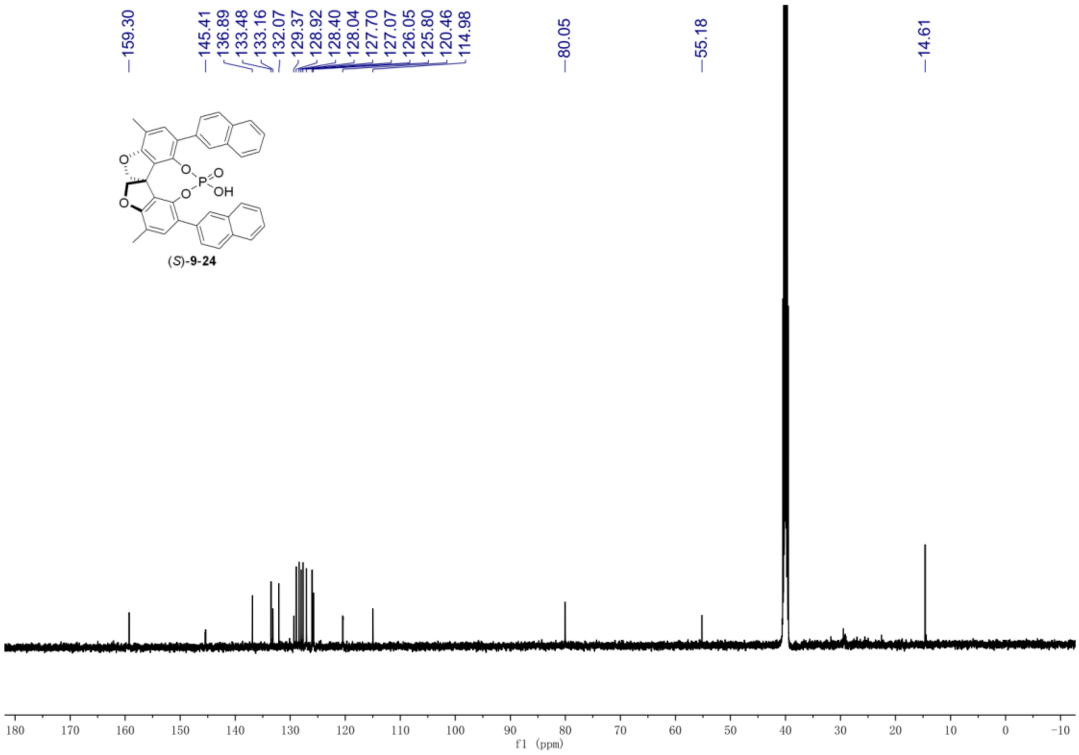

^{13}C NMR of (*S*)-**9-24** (DMSO-d_6)

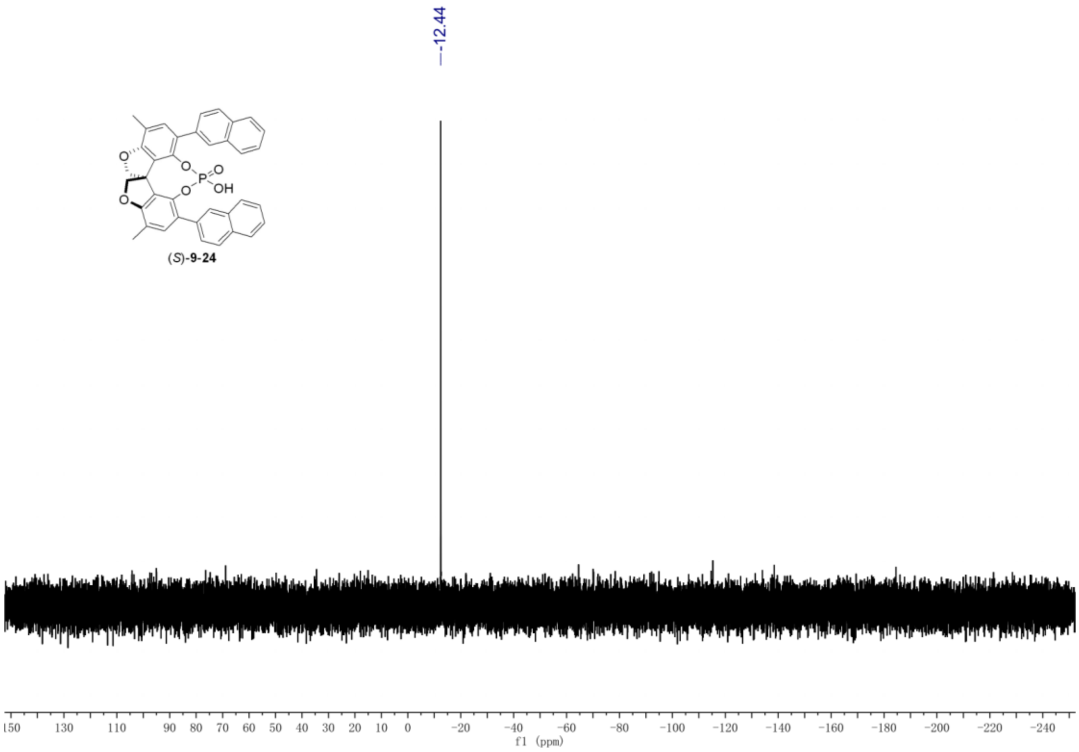

^{31}P NMR of (*S*)-**9-24** (DMSO-d_6)

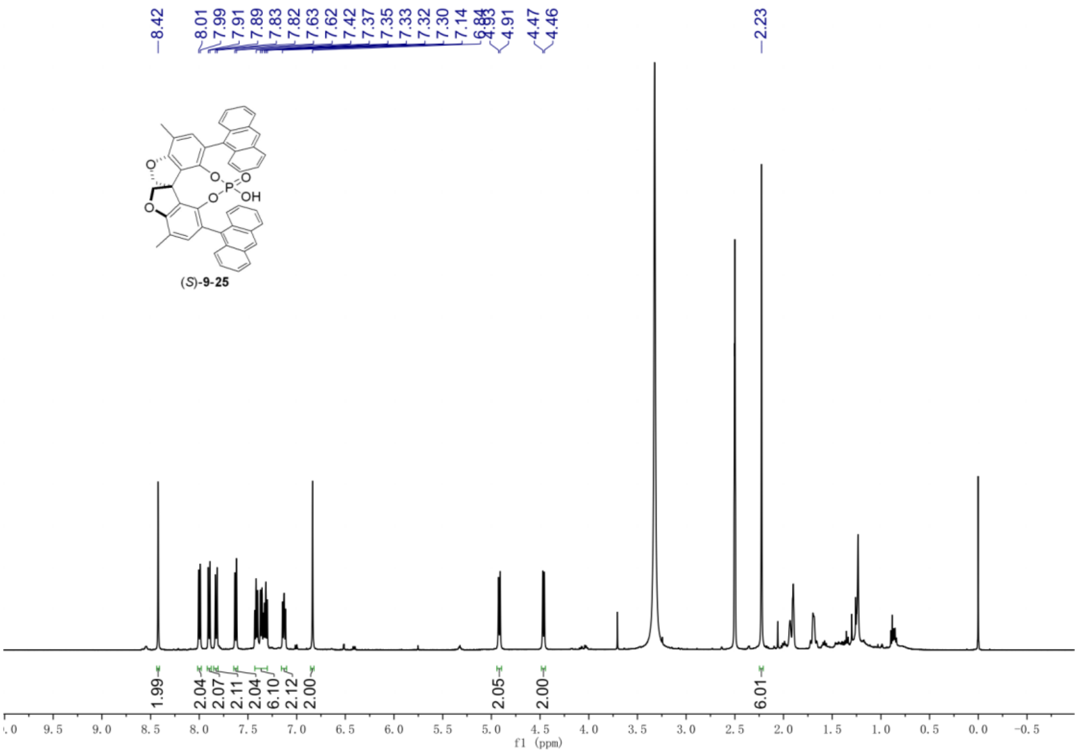

^{1}H NMR of (*S*)-**9-25** (DMSO-d_6)

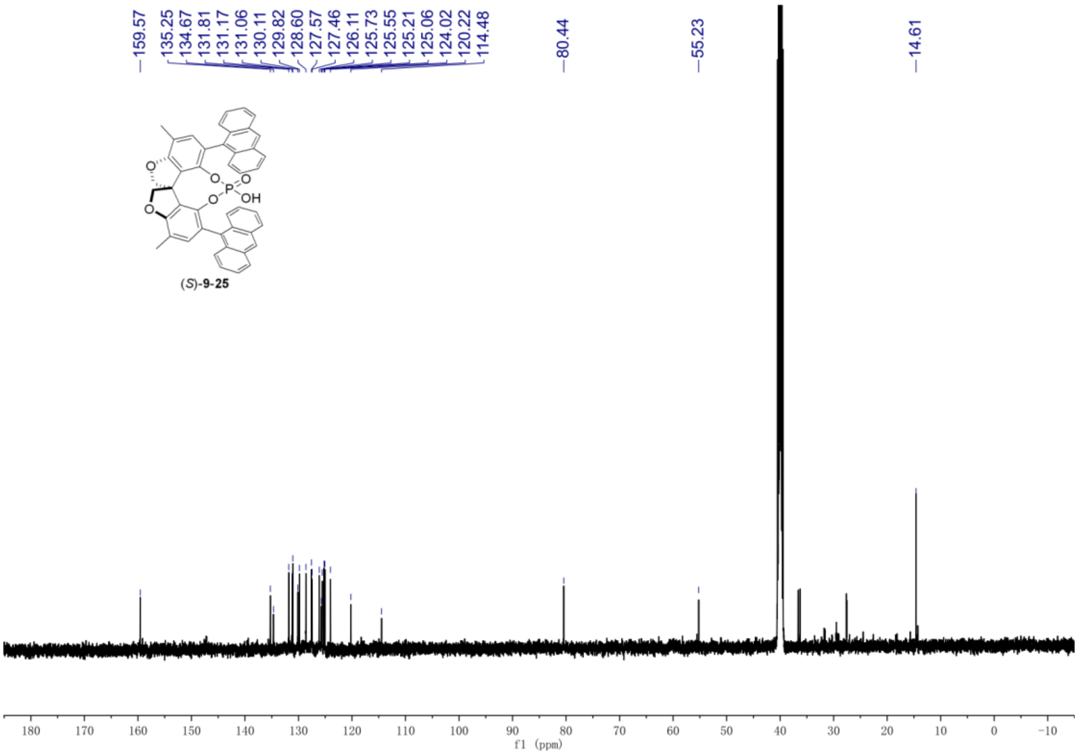

^{13}C NMR of (*S*)-**9-25** (DMSO-d_6)

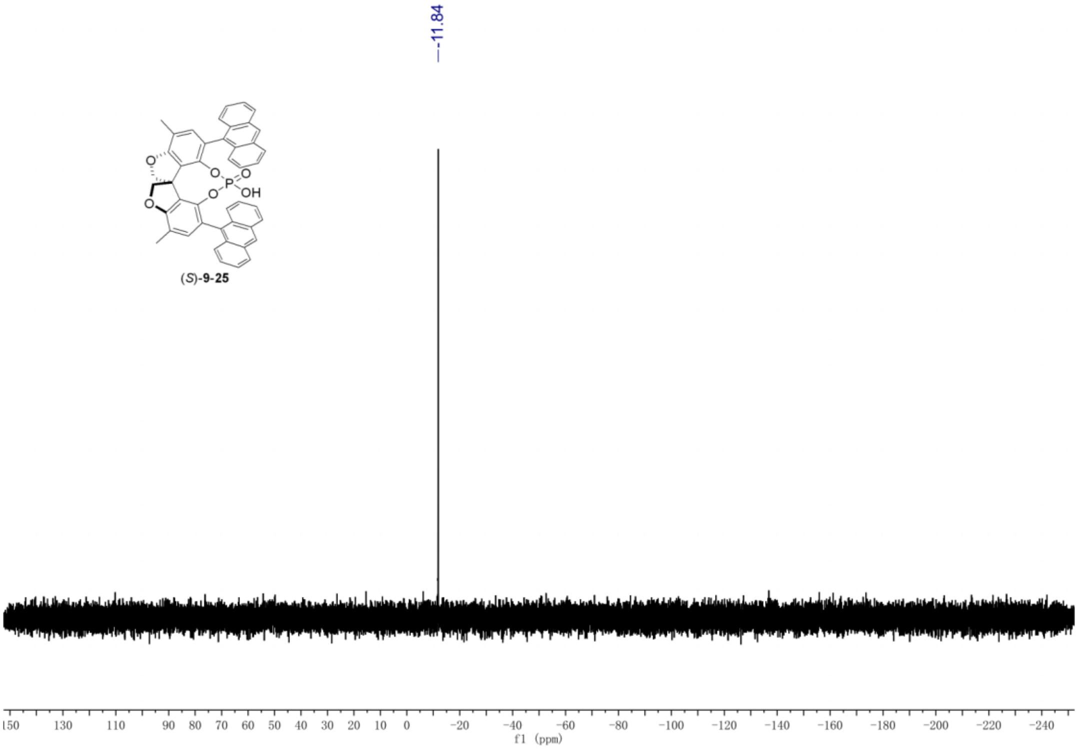

^{31}P NMR of (*S*)-**9-25** (DMSO-d_6)

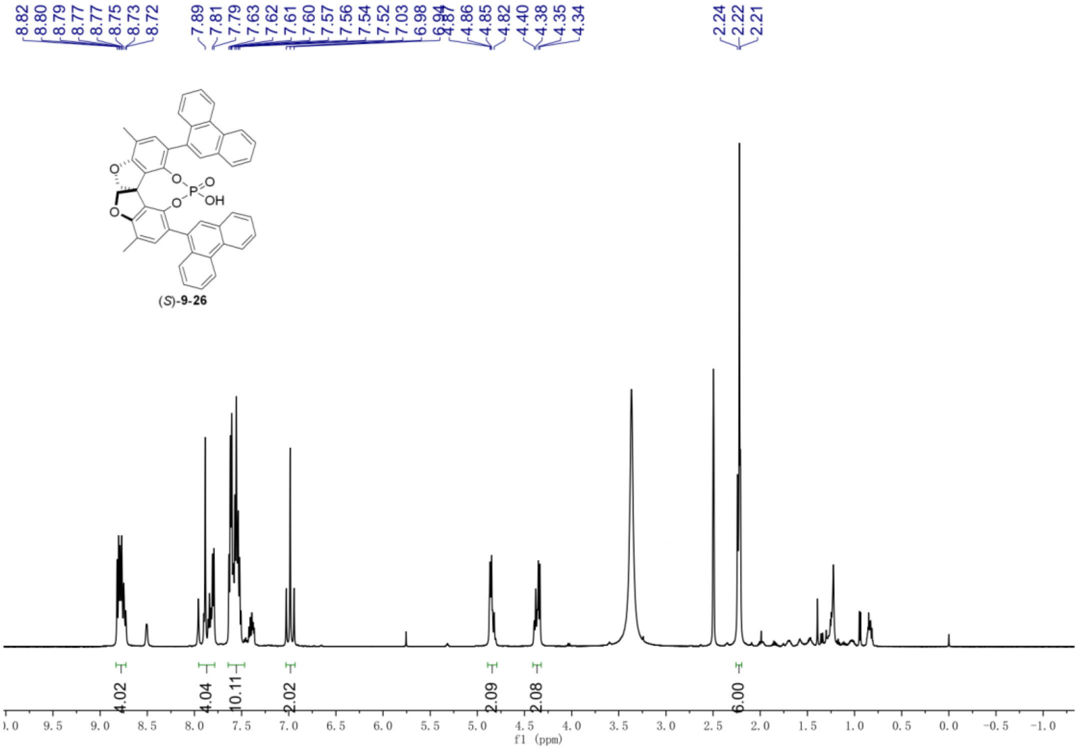

^{1}H NMR of (*S*)-**9-26** (DMSO-d_6)

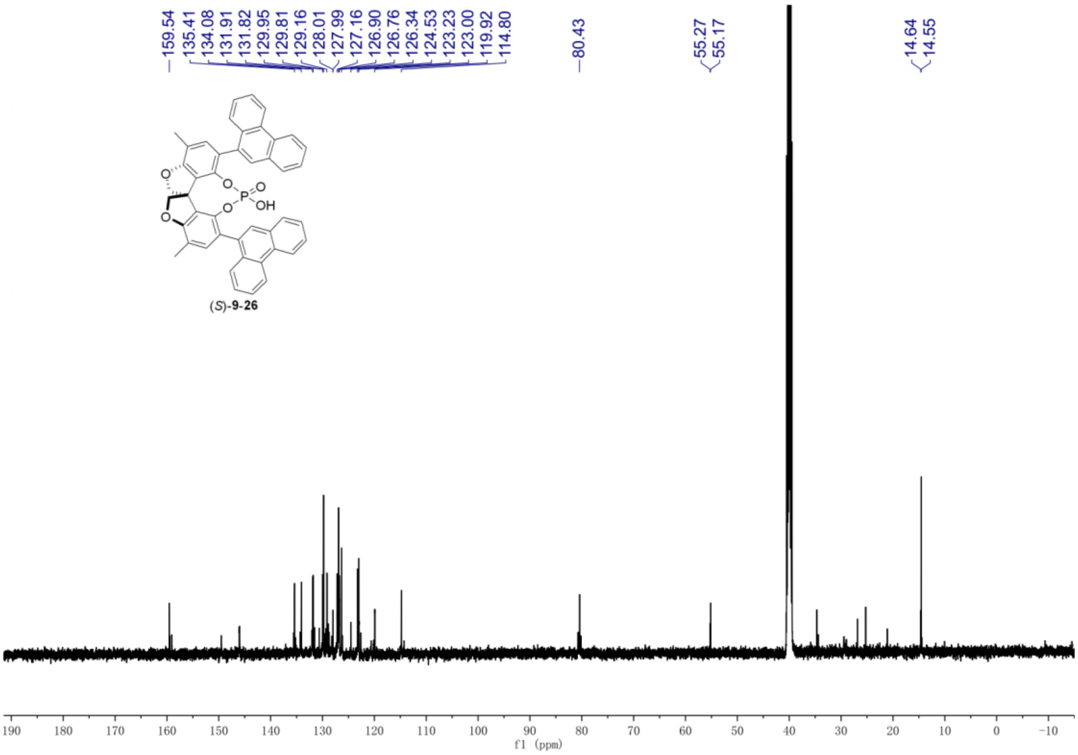

^{13}C NMR of (*S*)-**9-26** (DMSO-d_6)

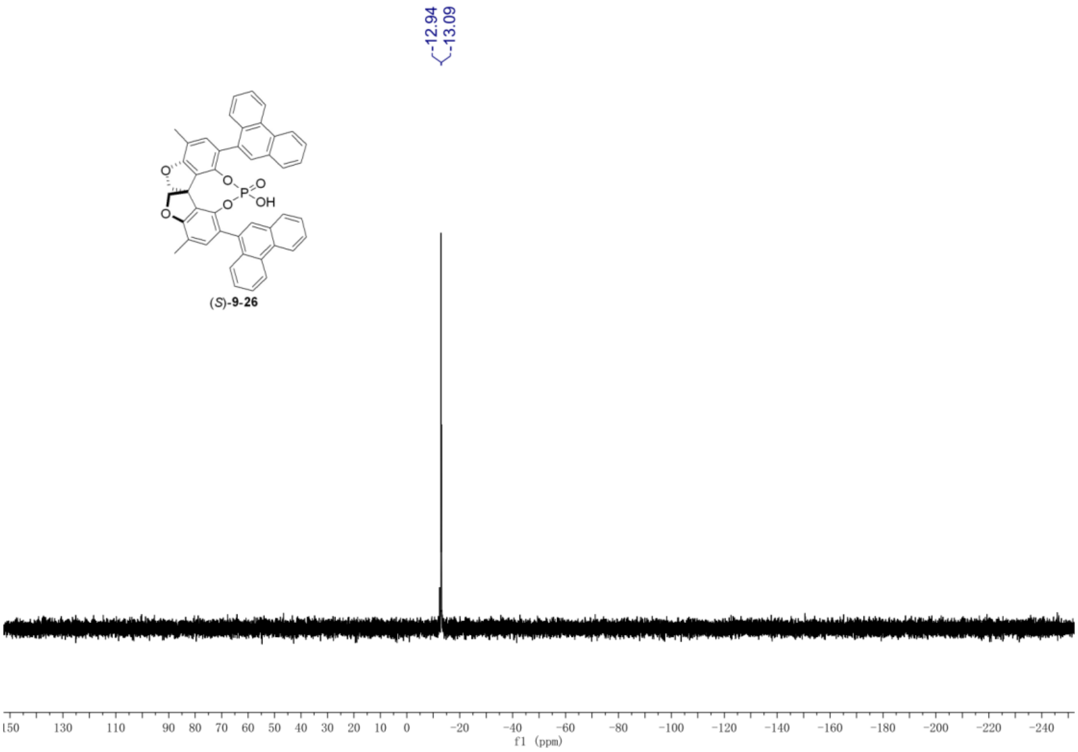

^{31}P NMR of (*S*)-**9-26** (DMSO-d_6)

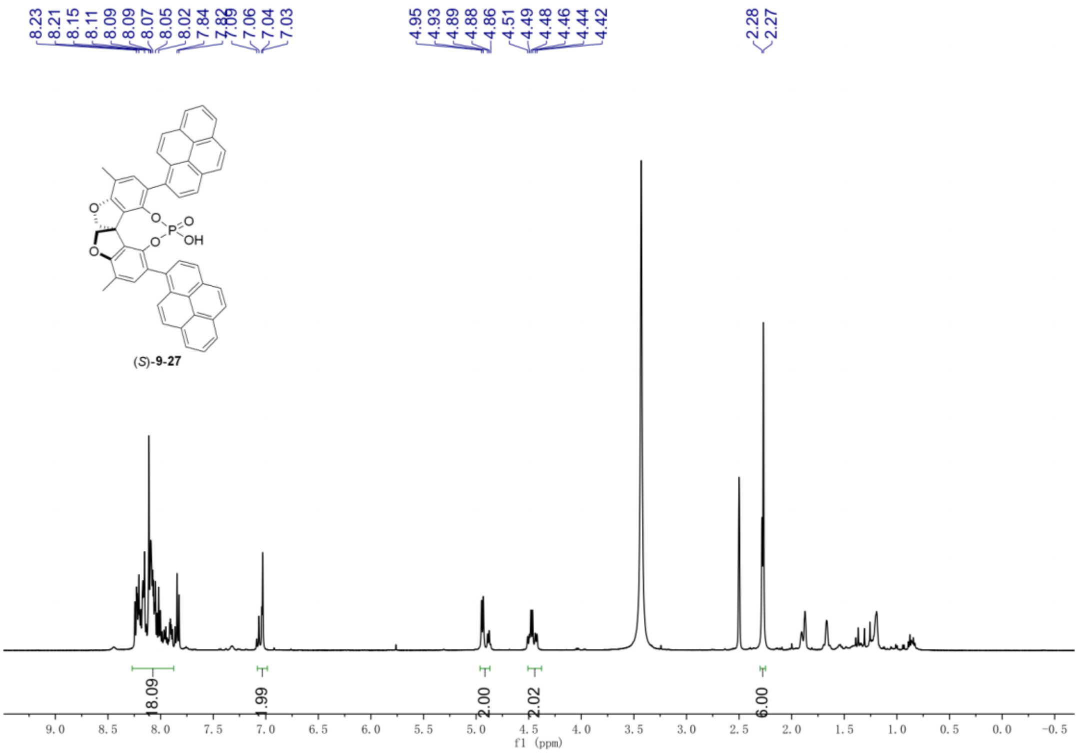

^{1}H NMR of (*S*)-**9-27** (DMSO-*d*$_6$)

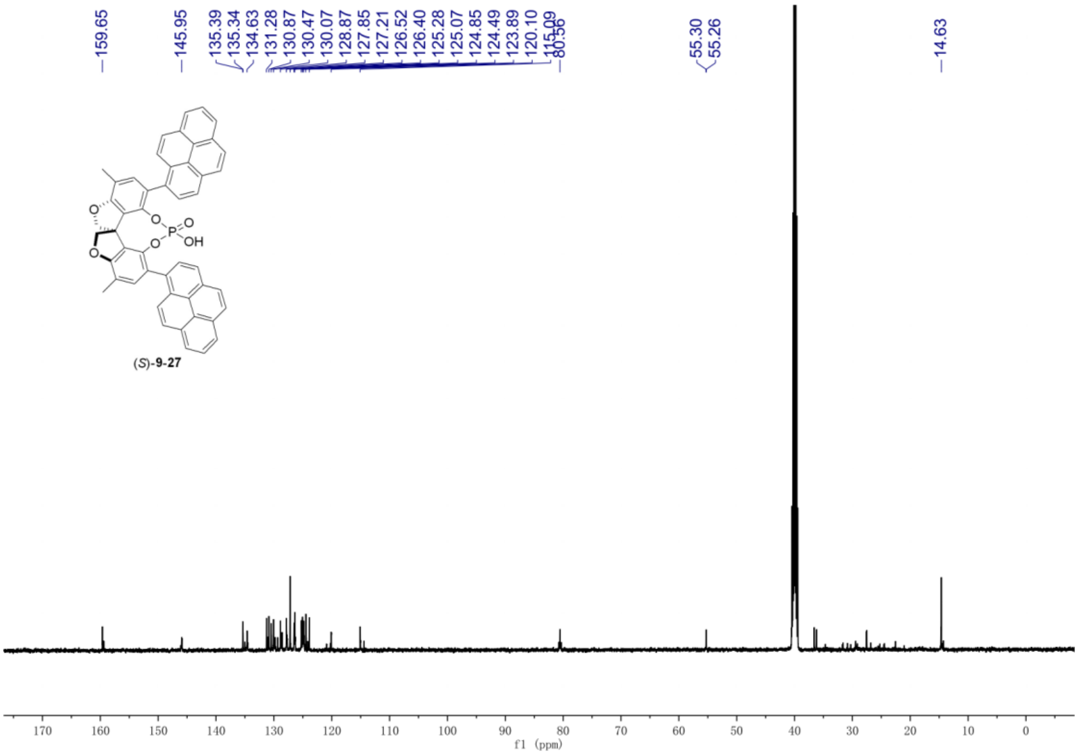

^{13}C NMR of (*S*)-**9-27** (DMSO-*d*$_6$)

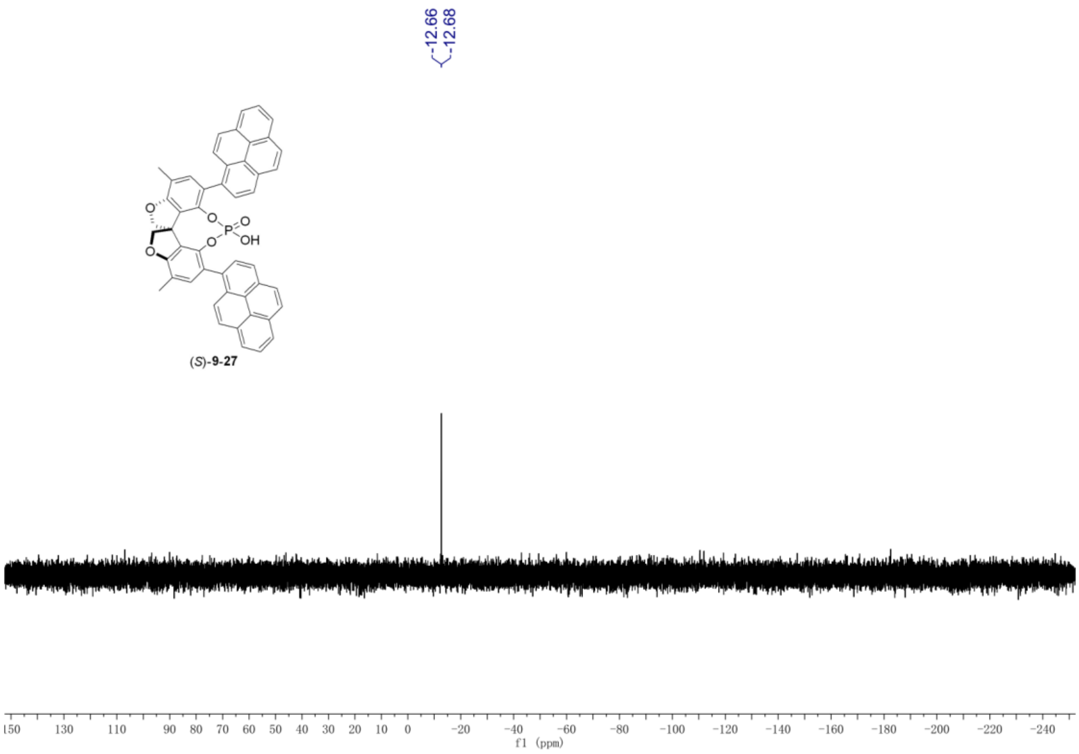

^{31}P NMR of (*S*)-**9-27** (DMSO-d_6)

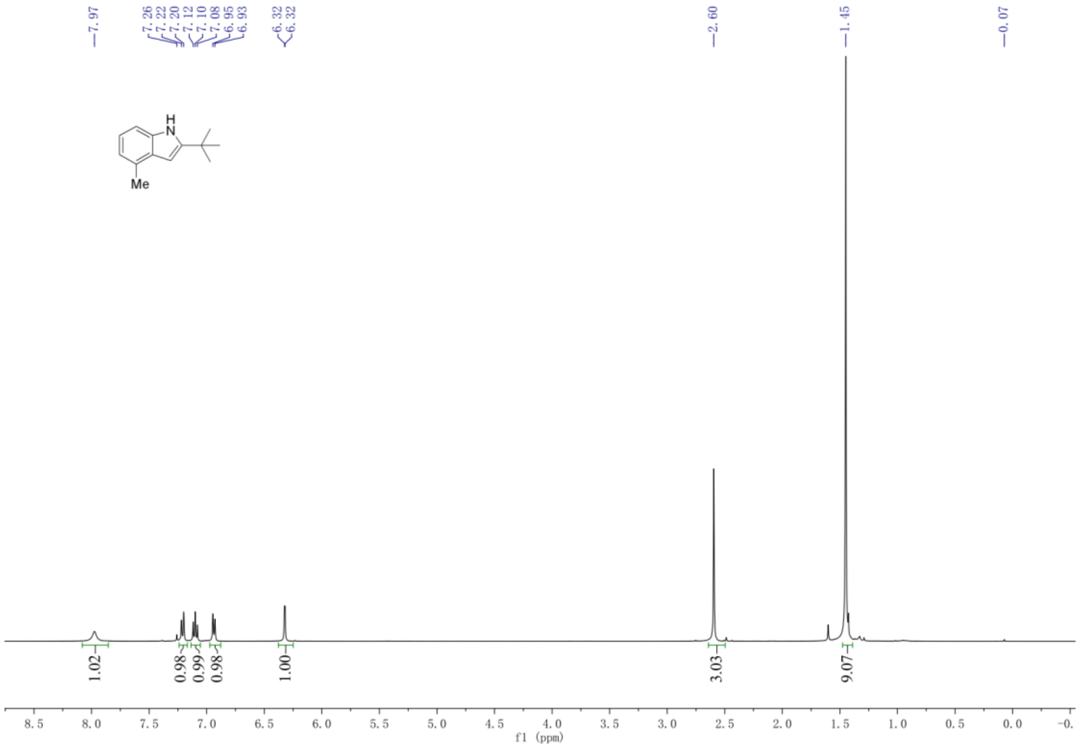

^{1}H NMR of **11-1** ($CDCl_3$)

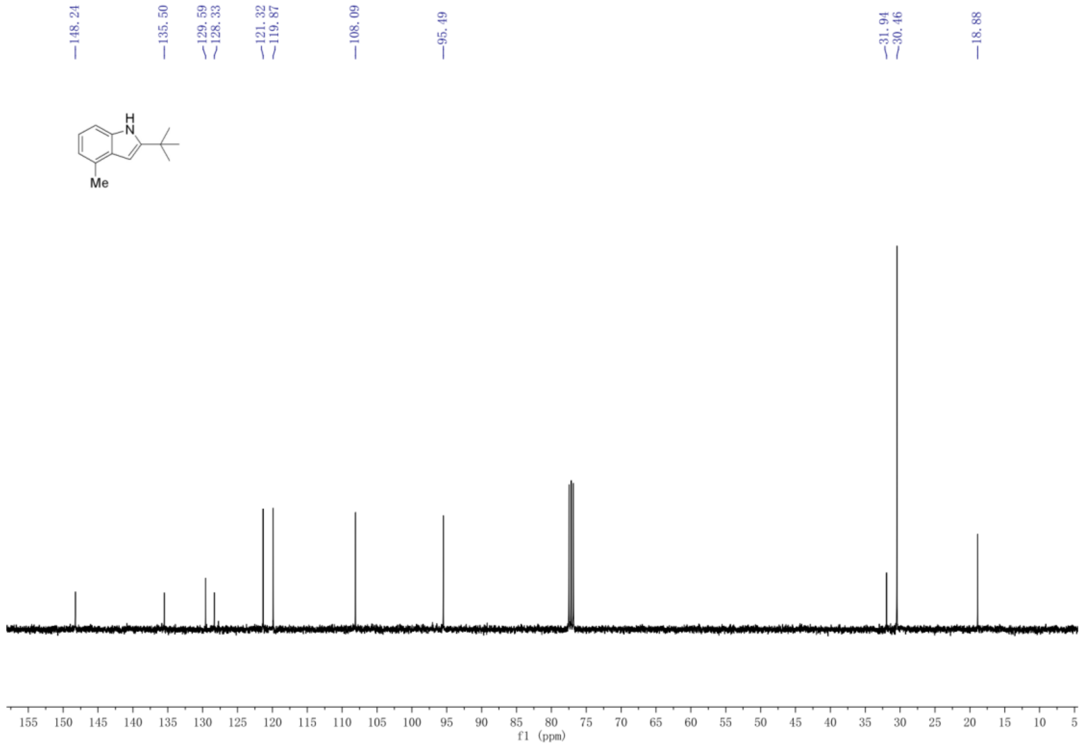

^{13}C NMR of **11-1** ($CDCl_3$)

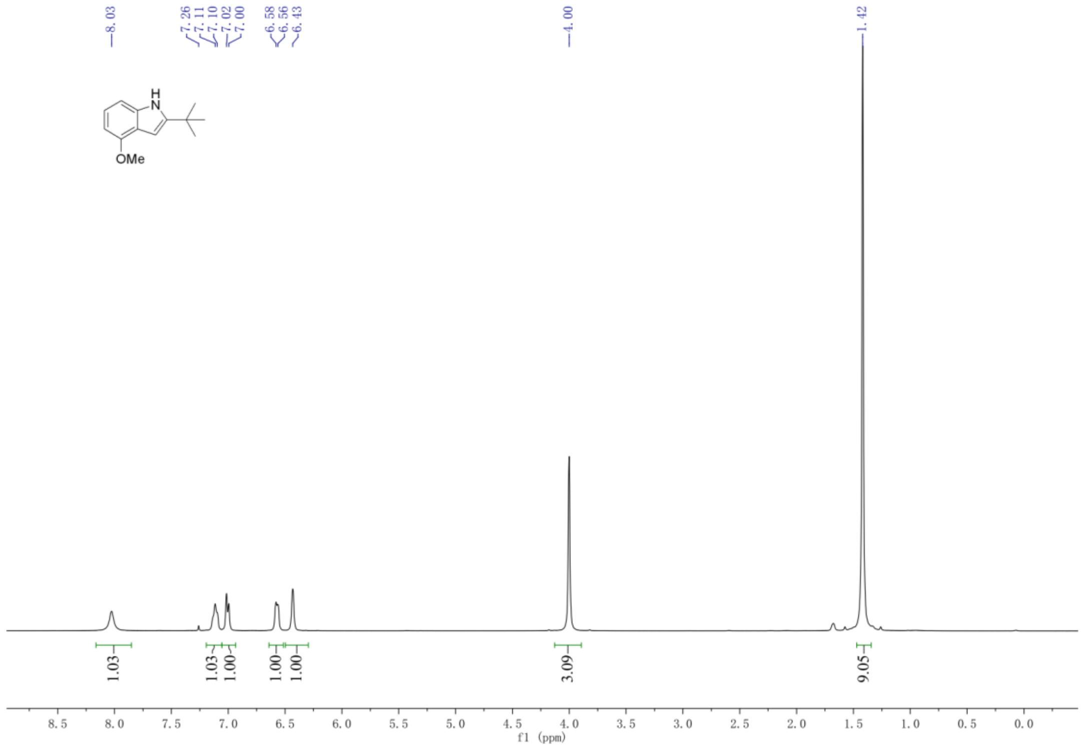

^{1}H NMR of **11-2** ($CDCl_3$)

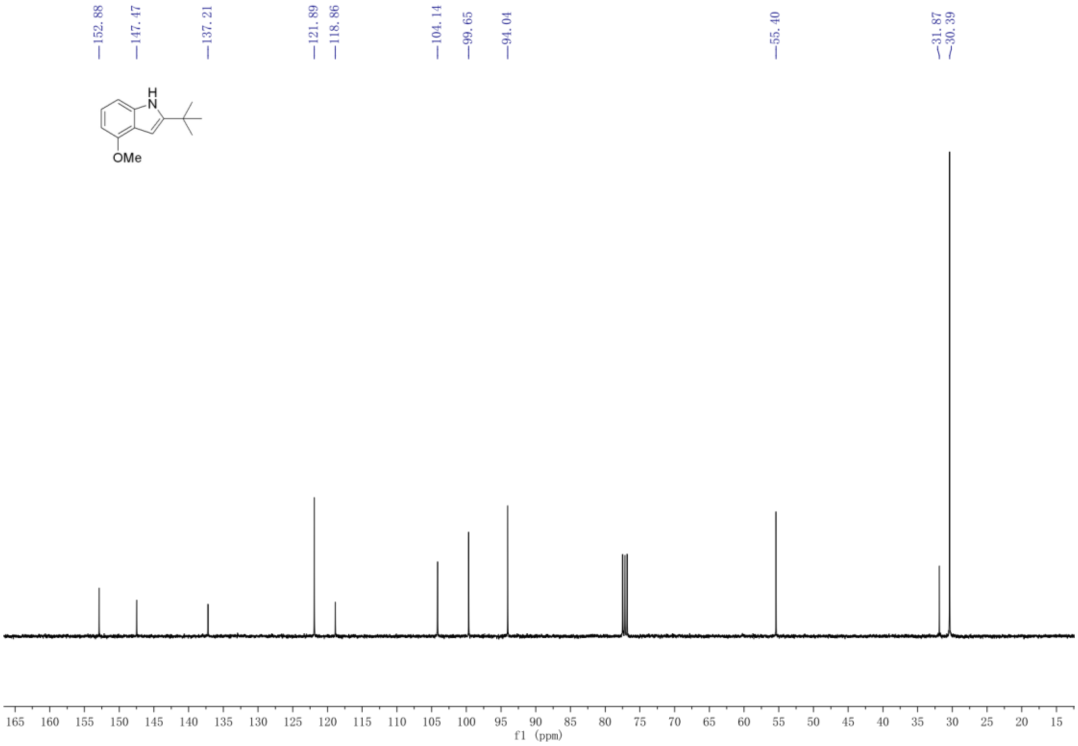

^{13}C NMR of **11-2** ($CDCl_3$)

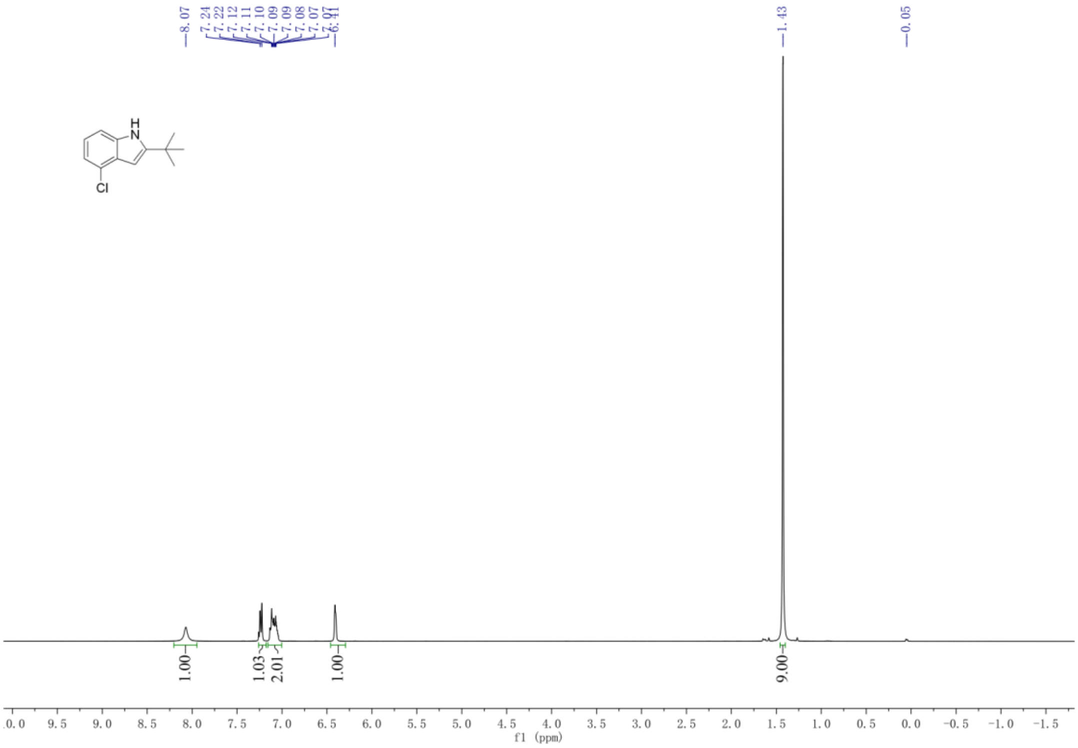

^{1}H NMR of **11-3** ($CDCl_3$)

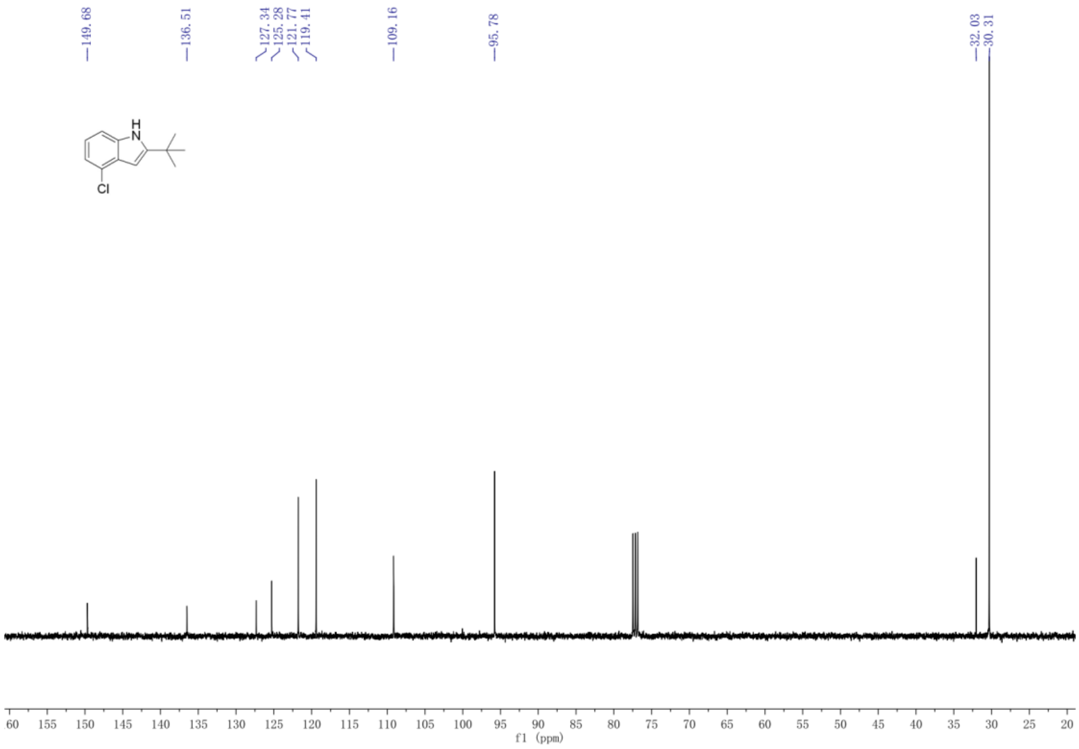

^{13}C NMR of **11-3** ($CDCl_3$)

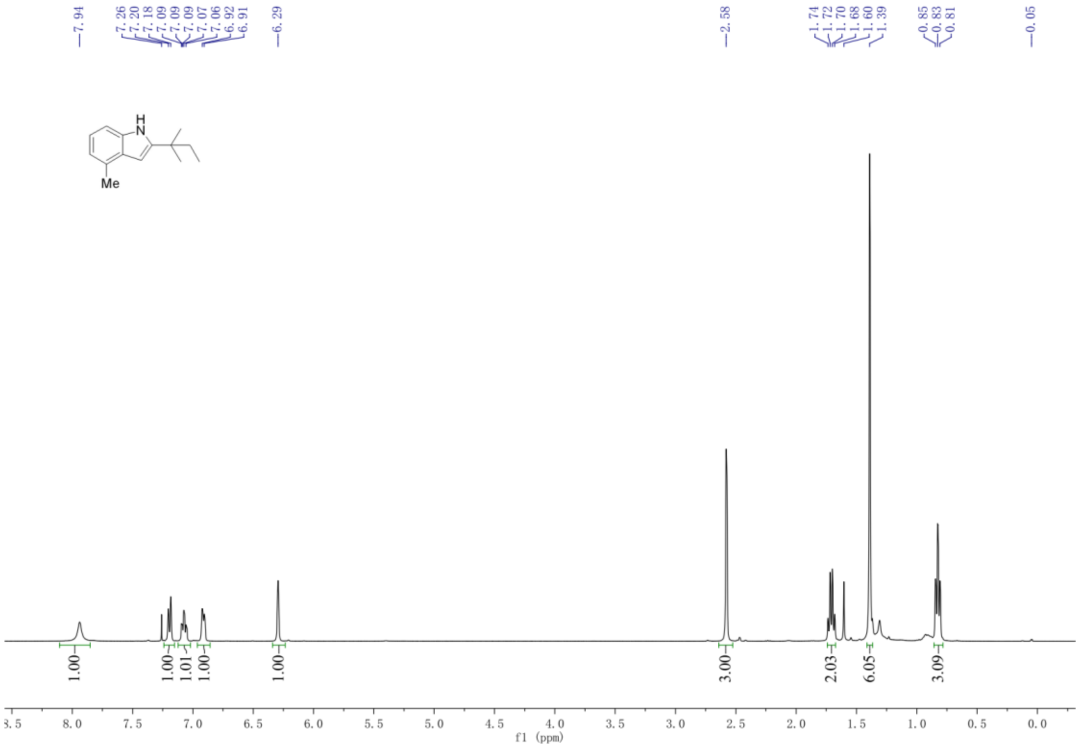

^{1}H NMR of **11-4** ($CDCl_3$)

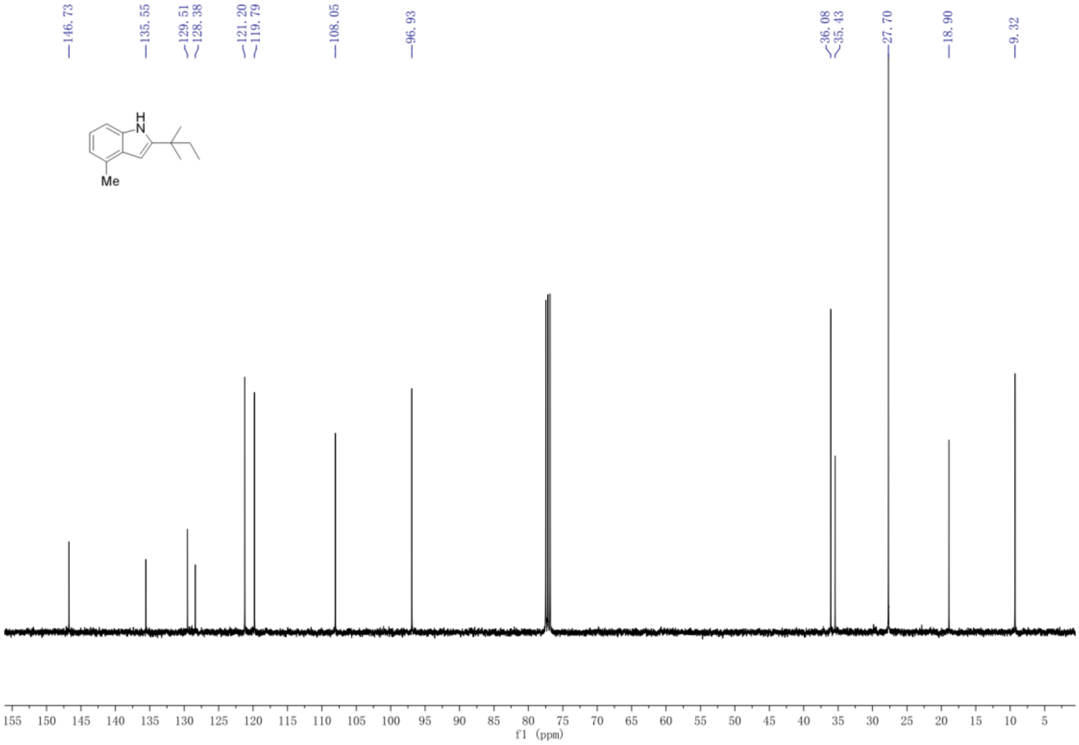

^{13}C NMR of **11-4** ($CDCl_3$)

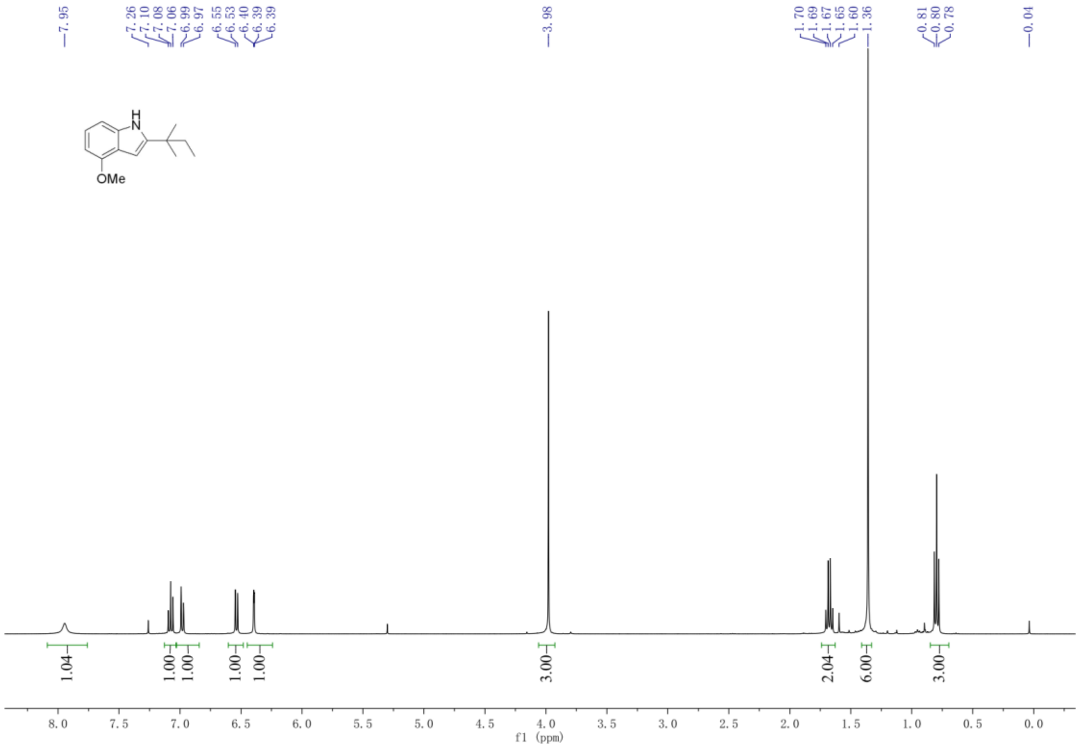

^{1}H NMR of **11-5** ($CDCl_3$)

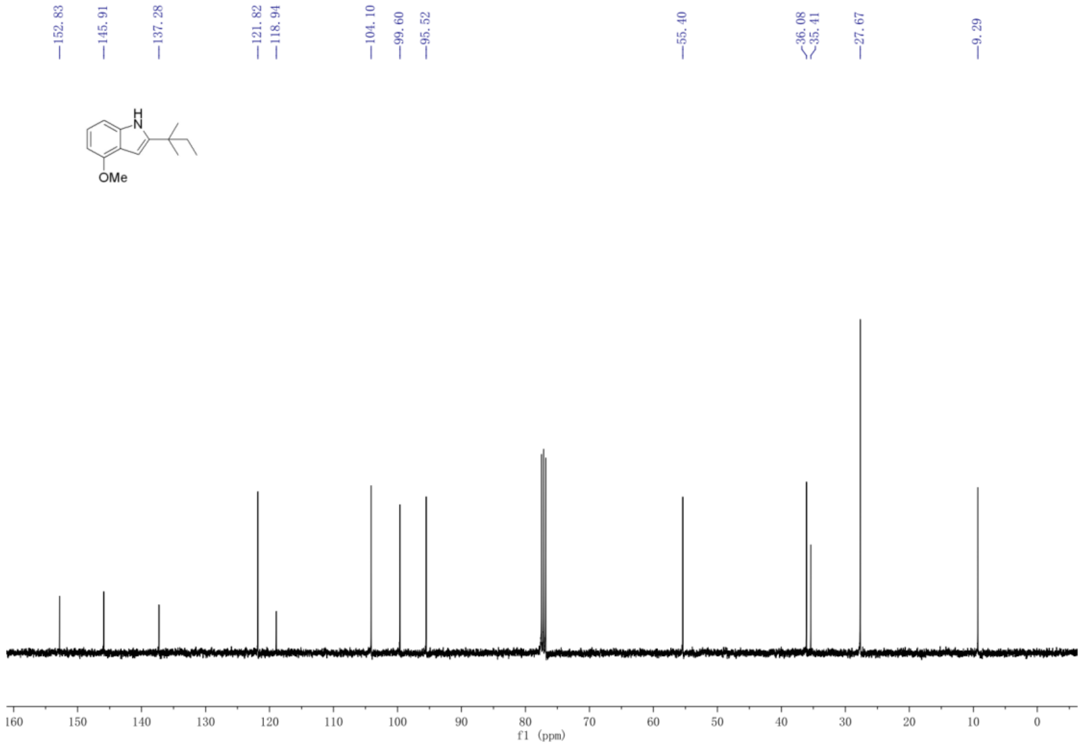

^{13}C NMR of **11-5** ($CDCl_3$)

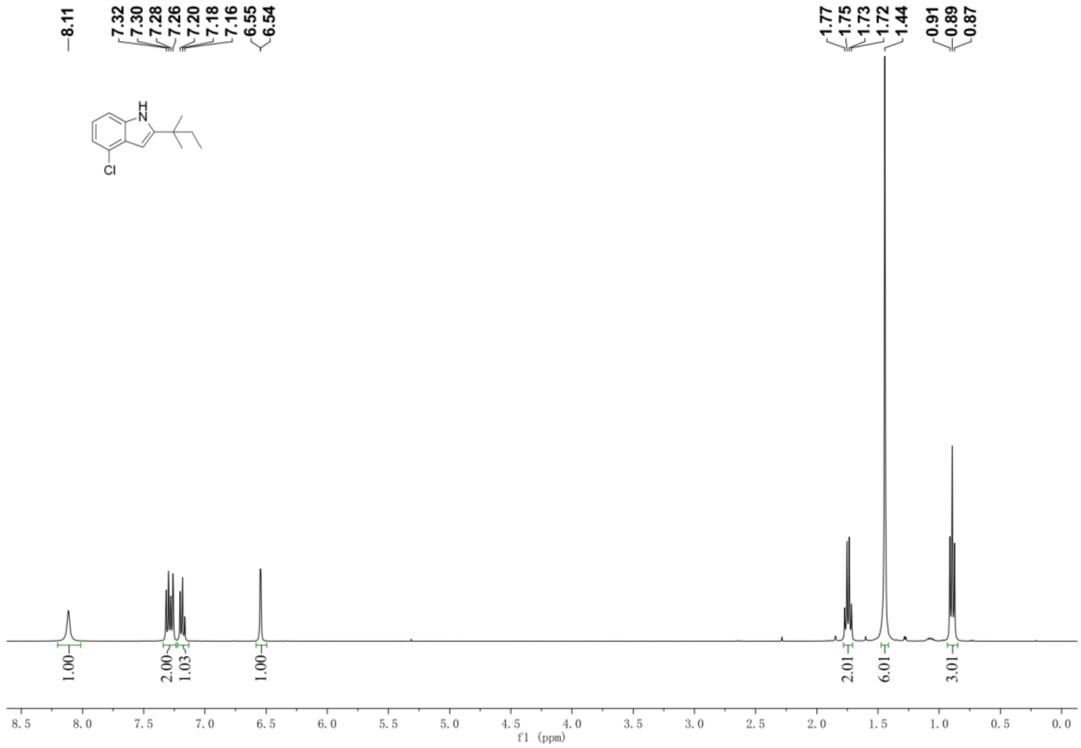

^{1}H NMR of **11-6** ($CDCl_3$)

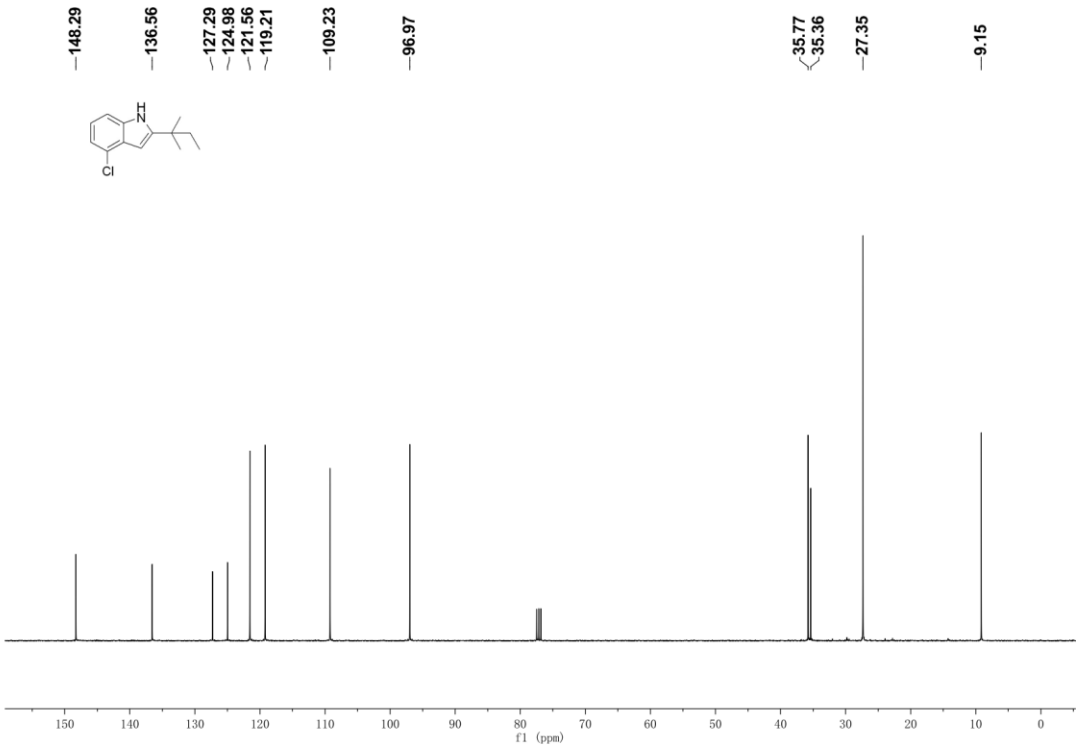

^{13}C NMR of **11-6** ($CDCl_3$)

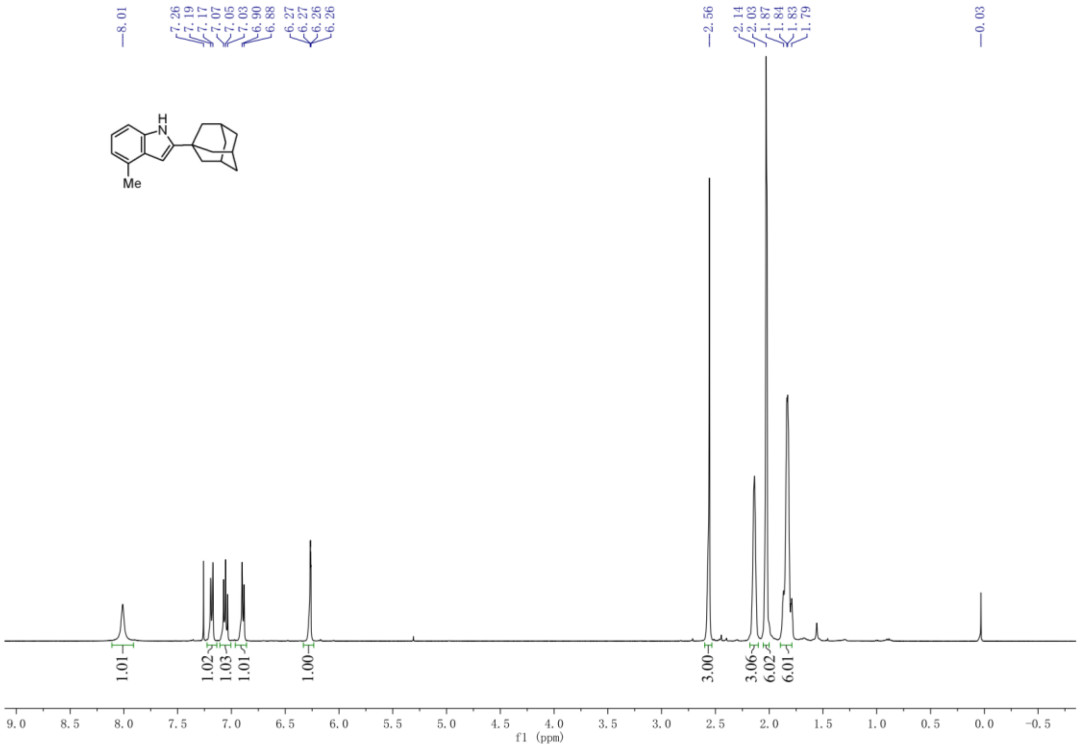

^{1}H NMR of **11-7** ($CDCl_3$)

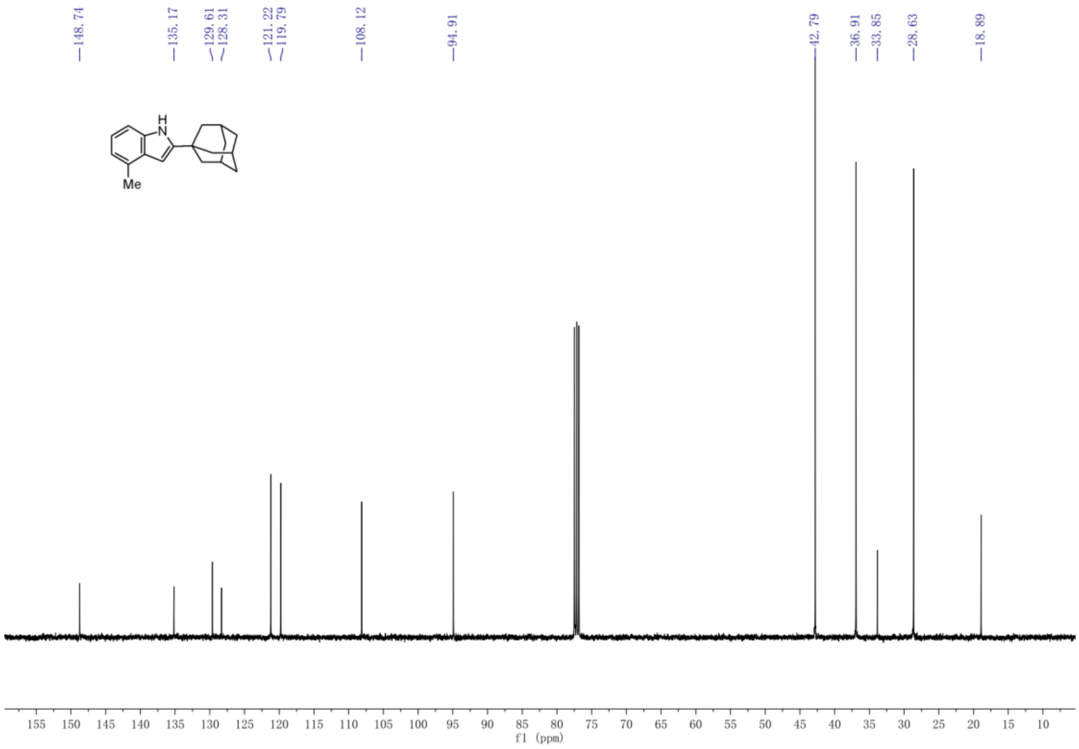

^{13}C NMR of **11-7** ($CDCl_3$)

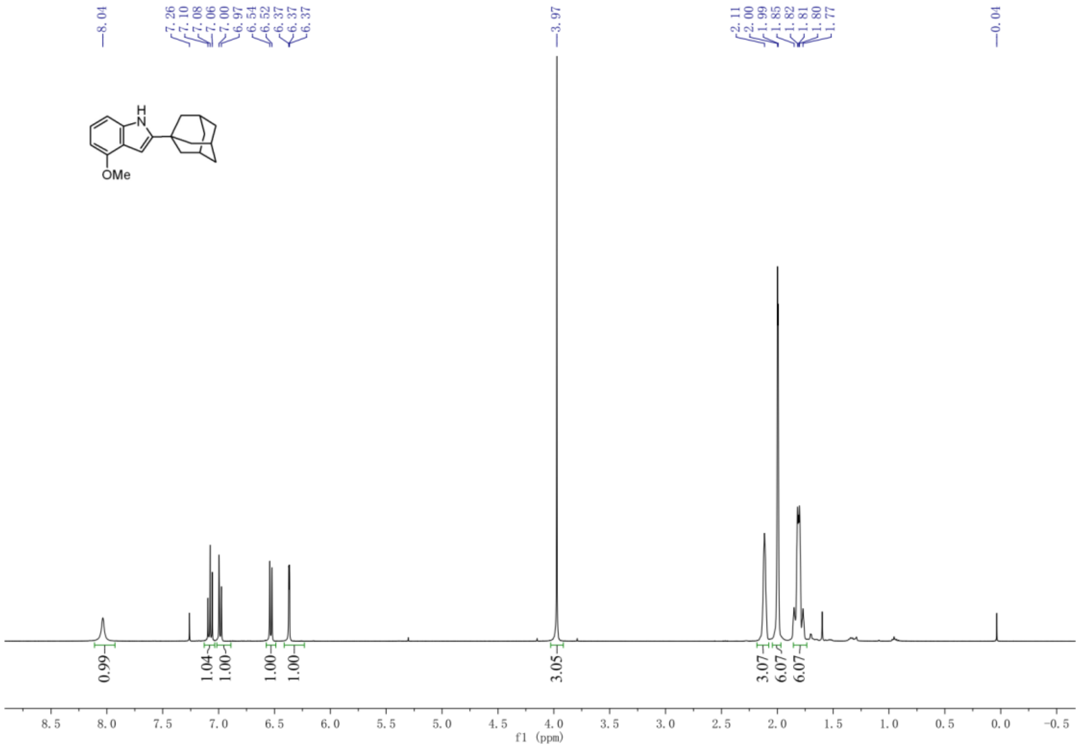

^{1}H NMR of **11-8** ($CDCl_3$)

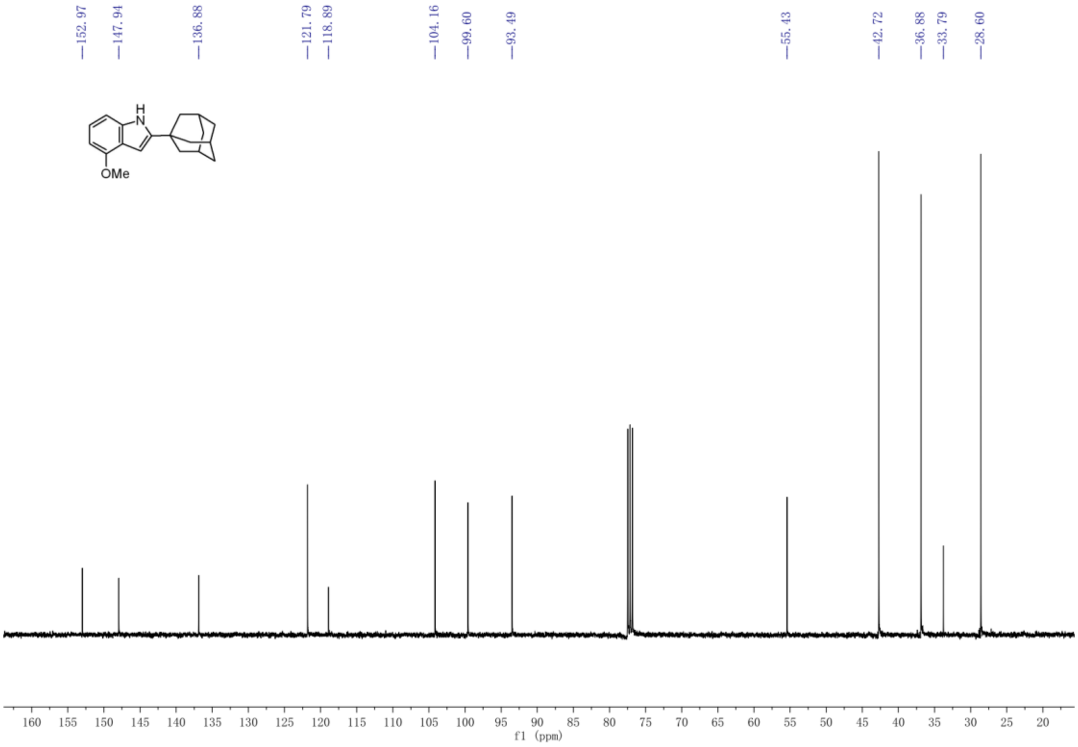

^{13}C NMR of **11-8** ($CDCl_3$)

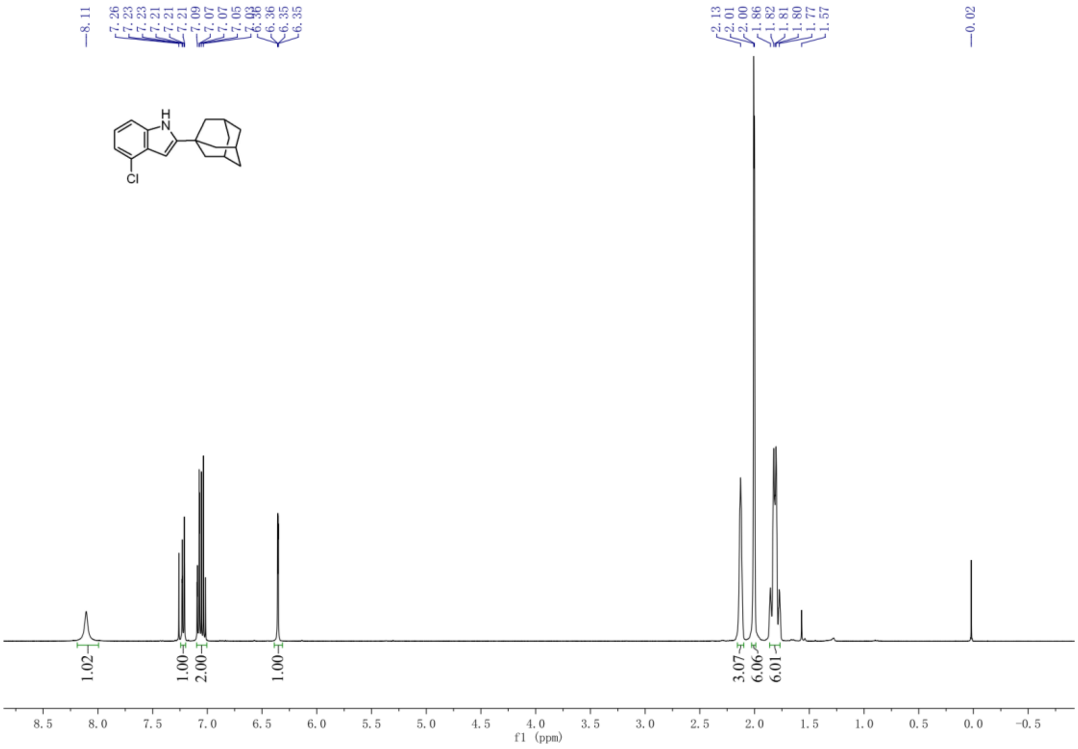

^{1}H NMR of **11-9** ($CDCl_3$)

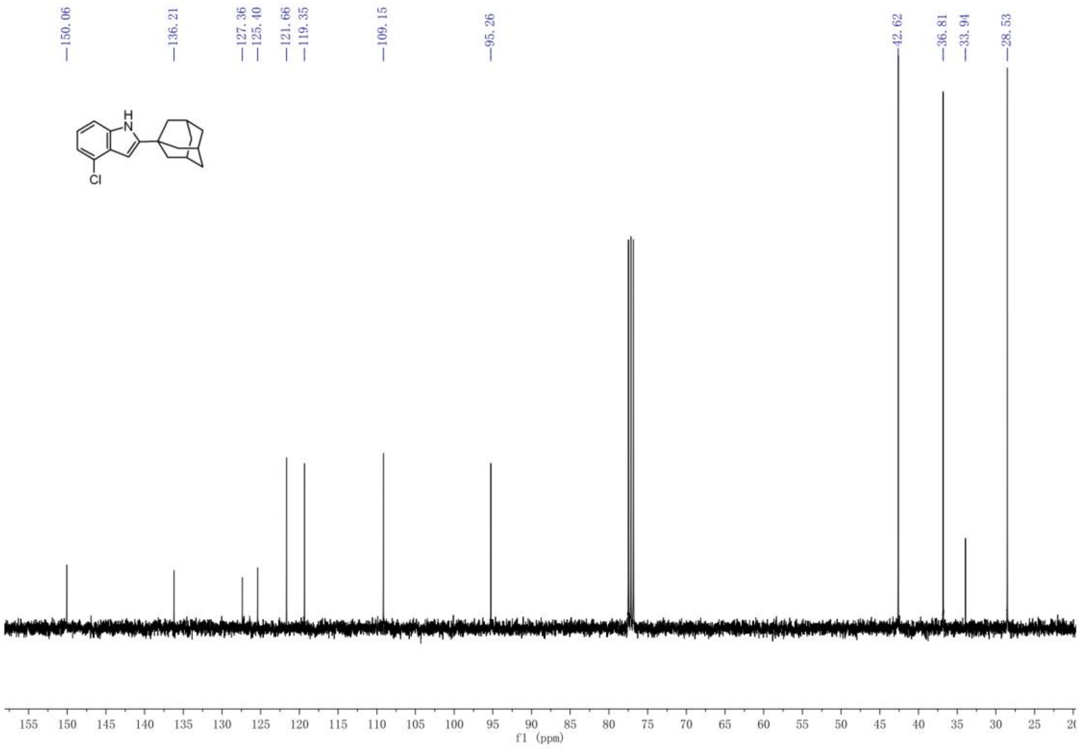

^{13}C NMR of **11-9** ($CDCl_3$)

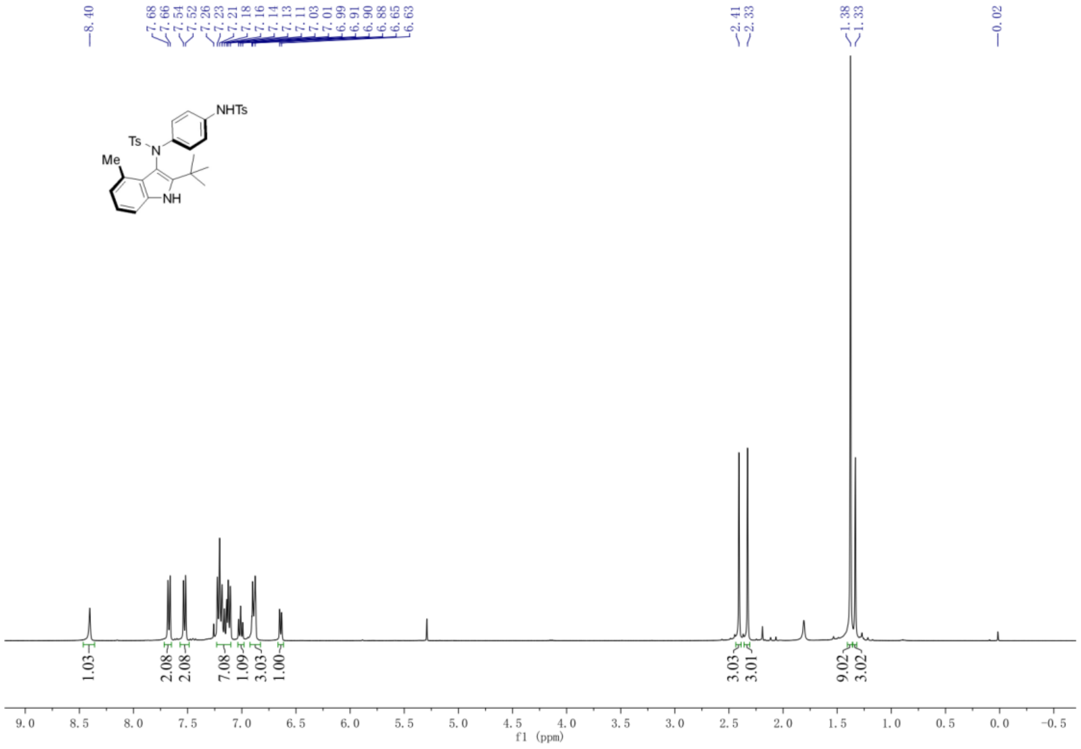

^{1}H NMR of **12-1** ($CDCl_3$)

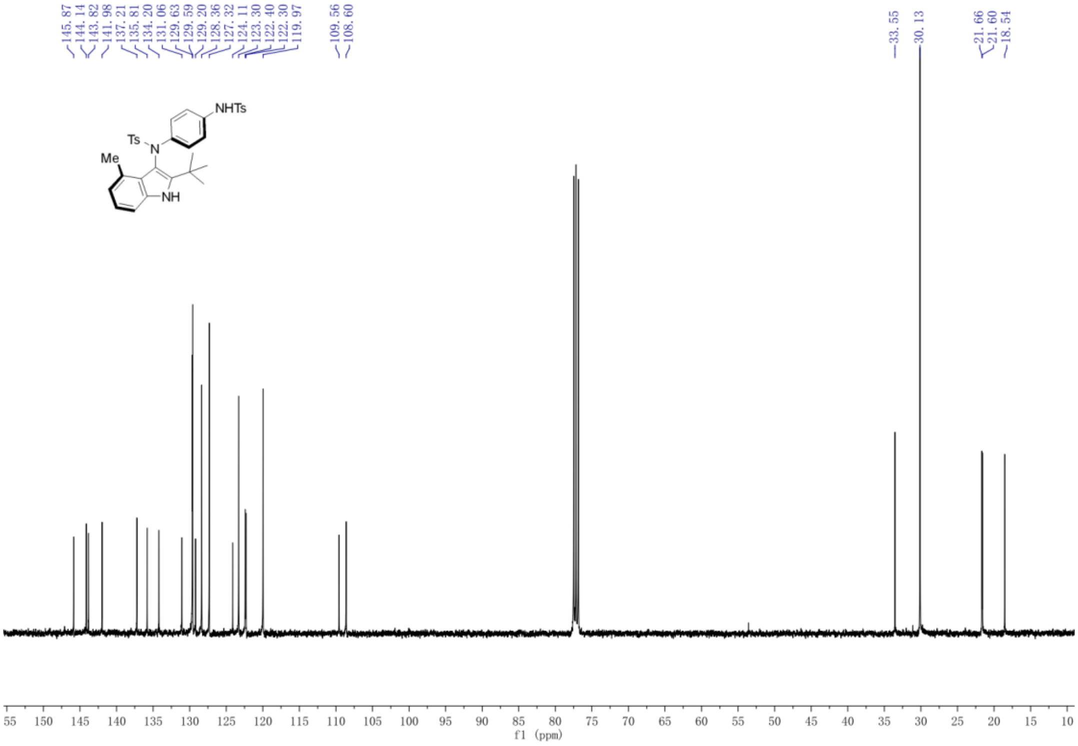

^{13}C NMR of **12-1** ($CDCl_3$)

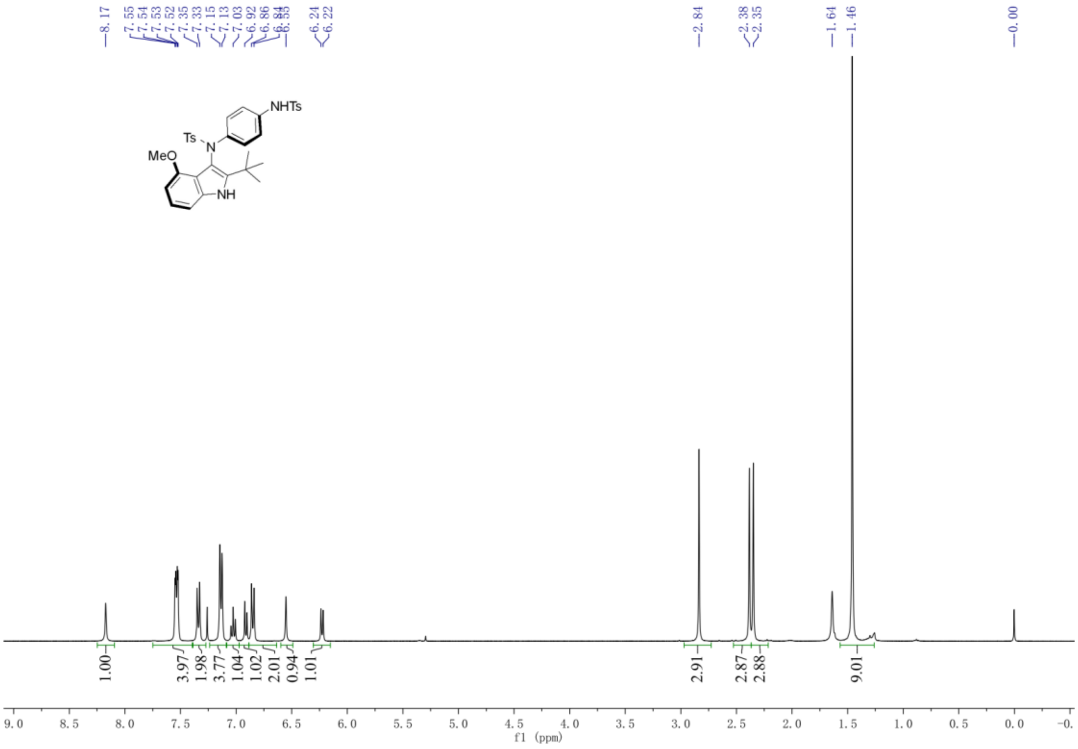

^{1}H NMR of **12-2** ($CDCl_3$)

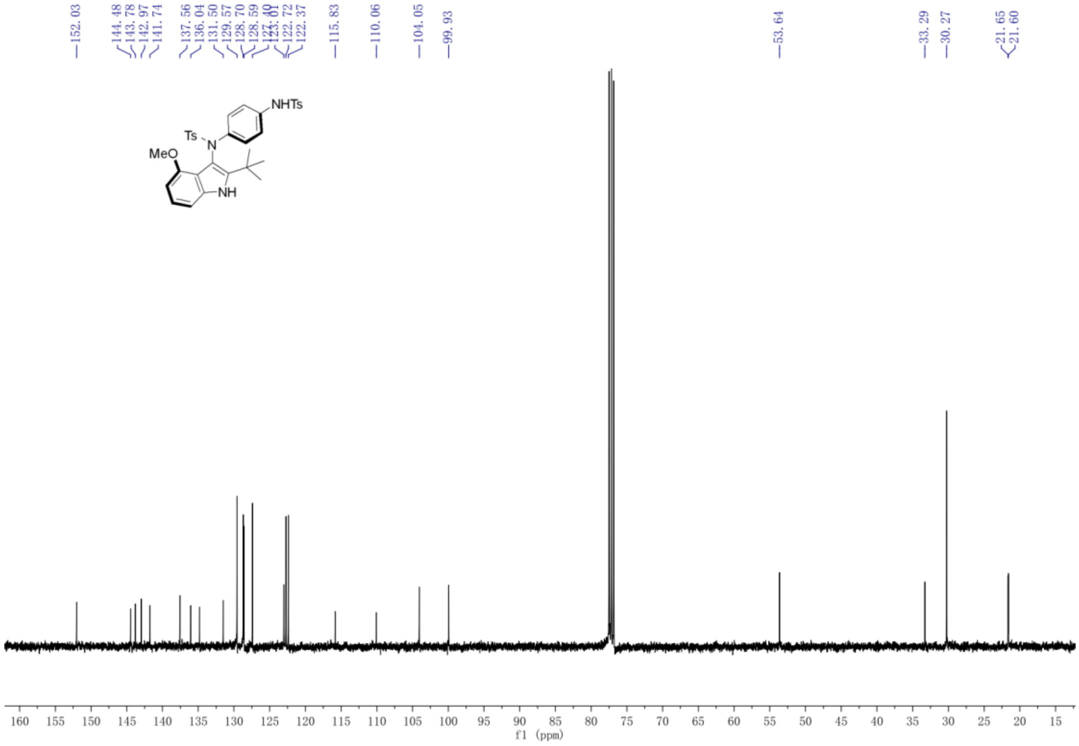

^{13}C NMR of **12-2** ($CDCl_3$)

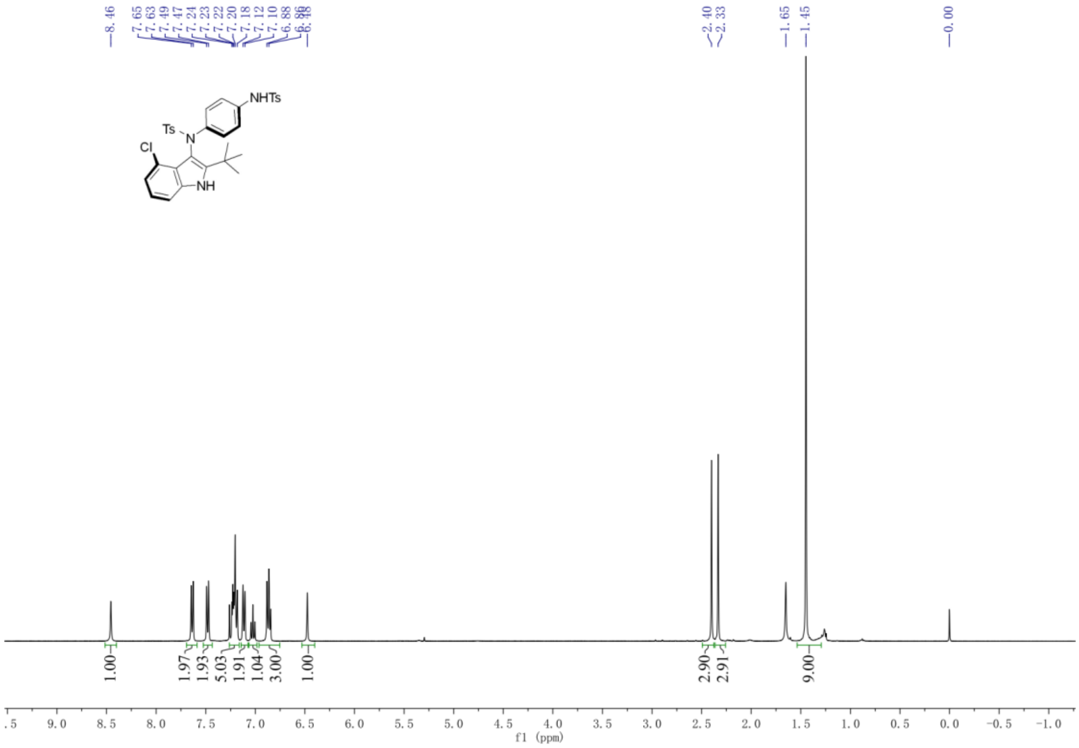

^{1}H NMR of **12-3** ($CDCl_3$)

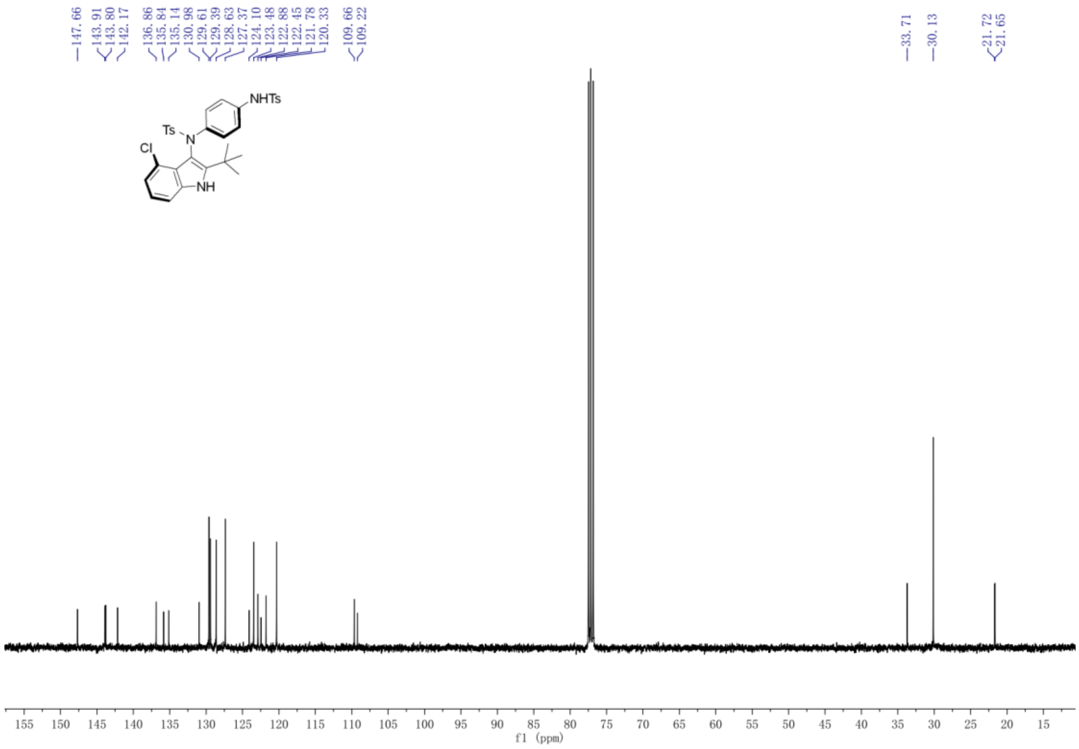

^{13}C NMR of **12-3** ($CDCl_3$)

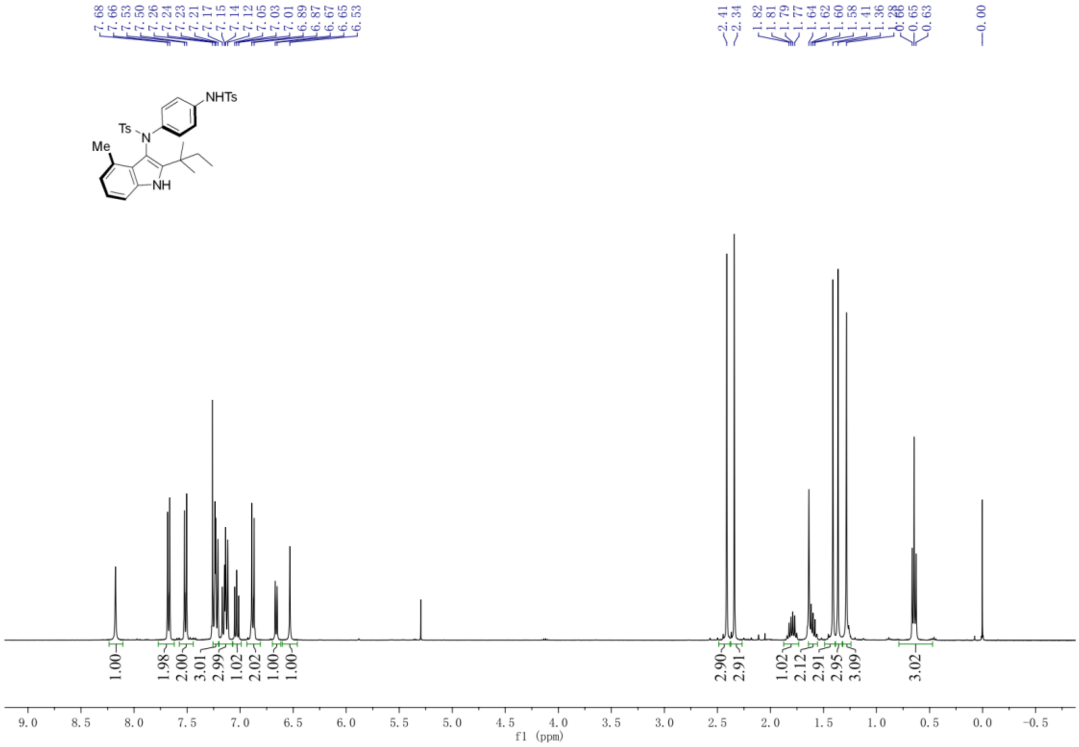

^{1}H NMR of **12-4** ($CDCl_3$)

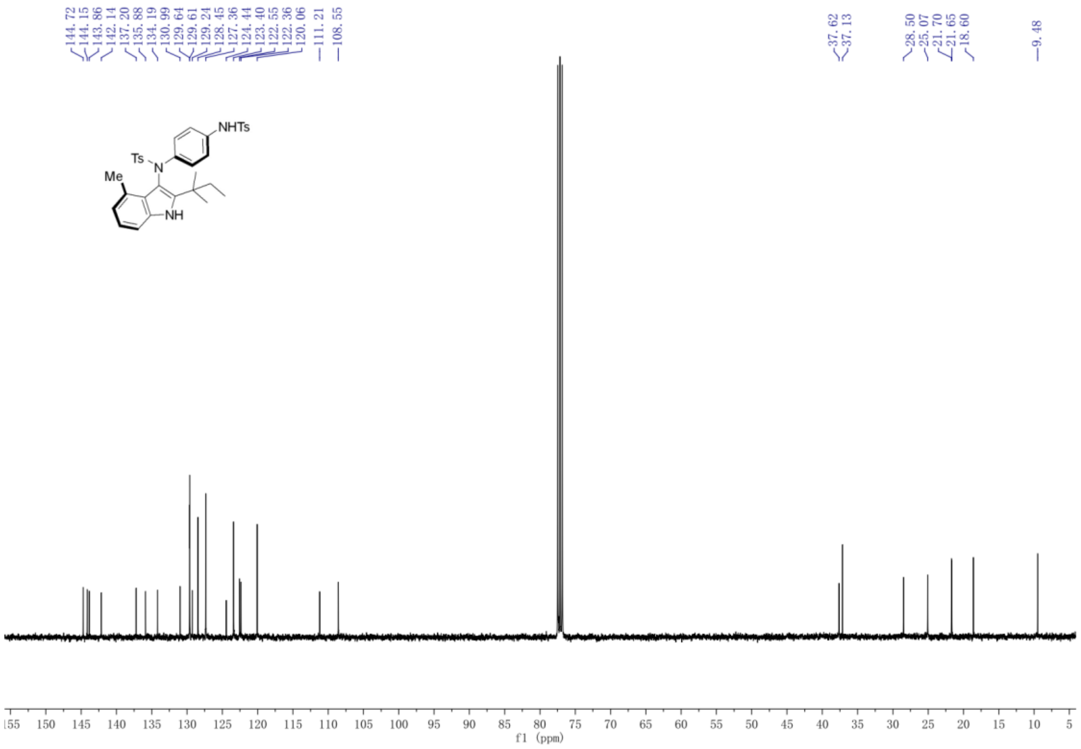

^{13}C NMR of **12-4** ($CDCl_3$)

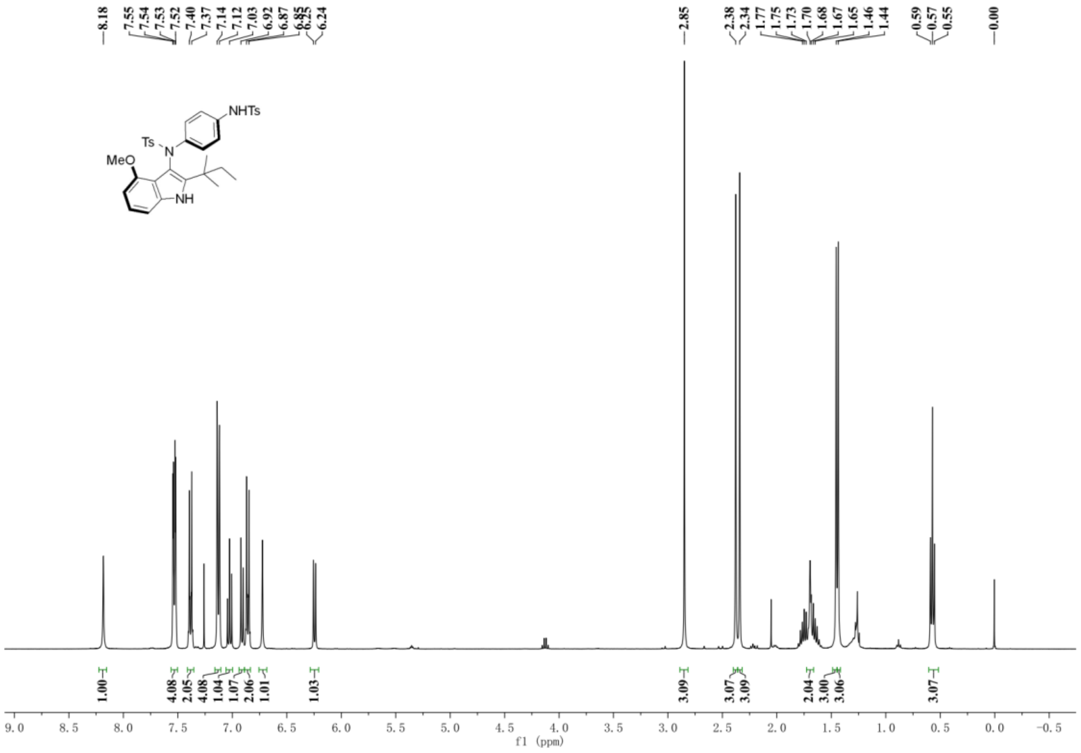

^{1}H NMR of **12-5** ($CDCl_3$)

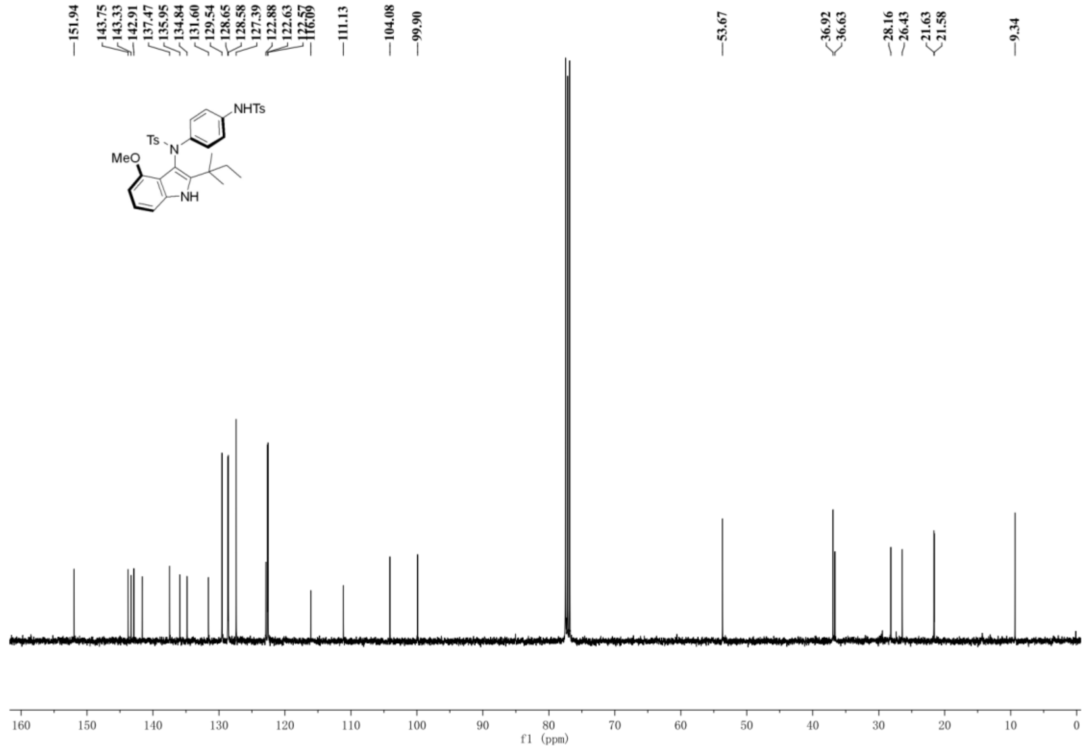

^{13}C NMR of **12-5** ($CDCl_3$)

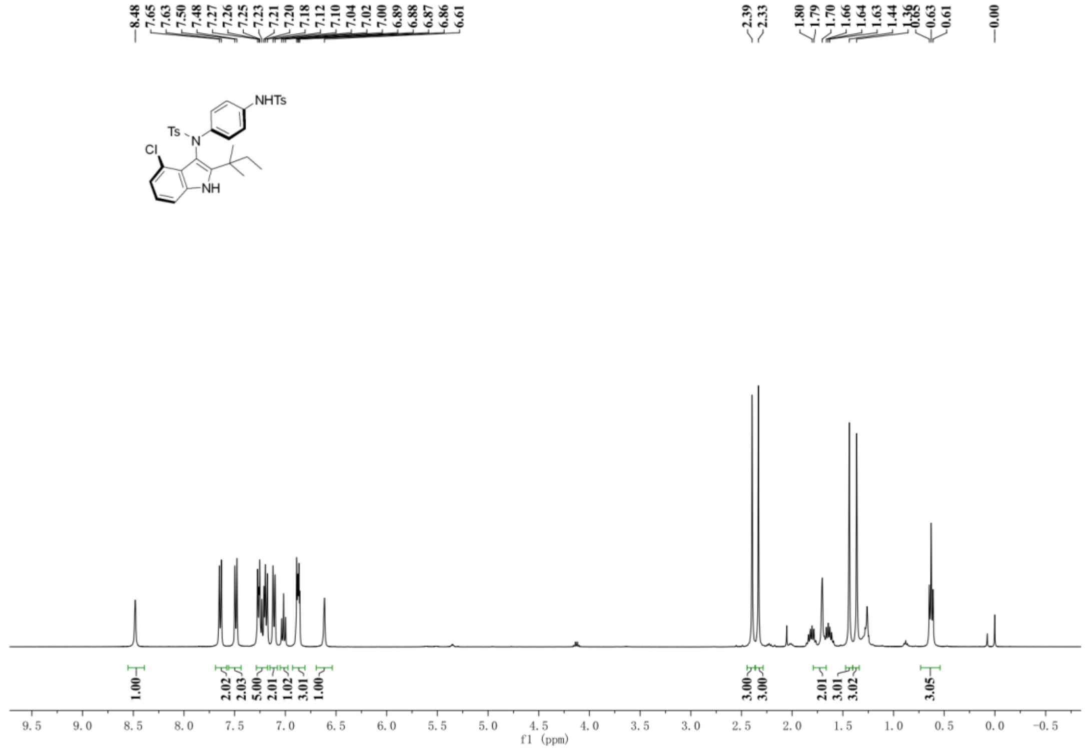

^{1}H NMR of **12-6** ($CDCl_3$)

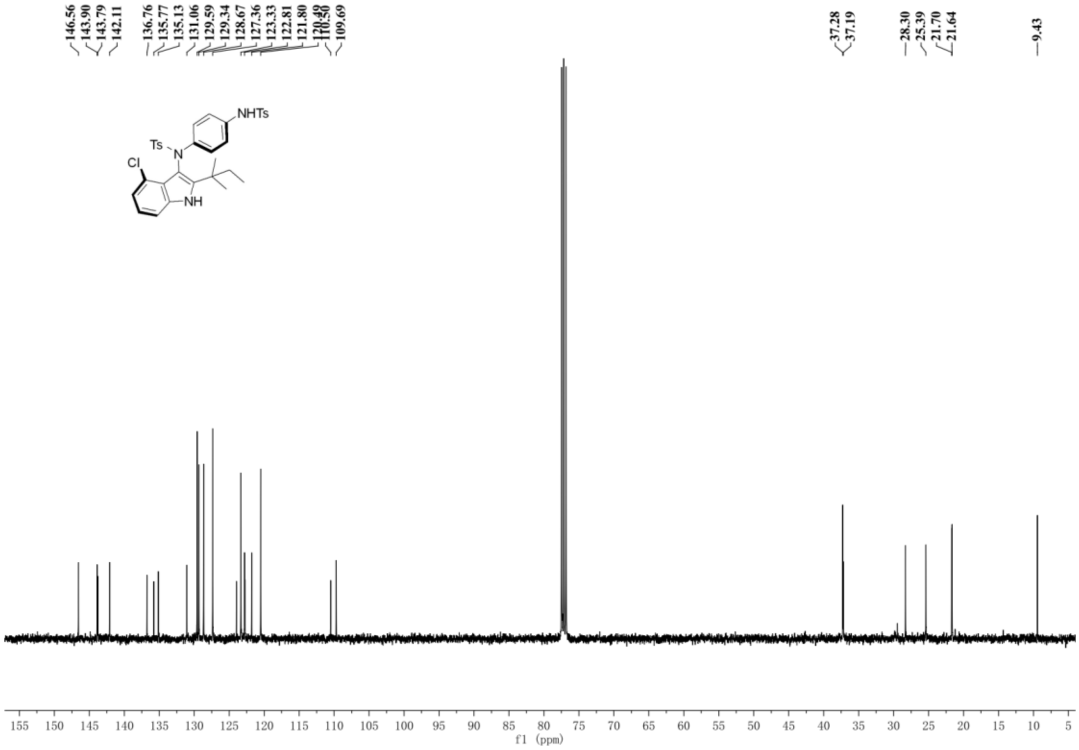

^{13}C NMR of **12-6** ($CDCl_3$)

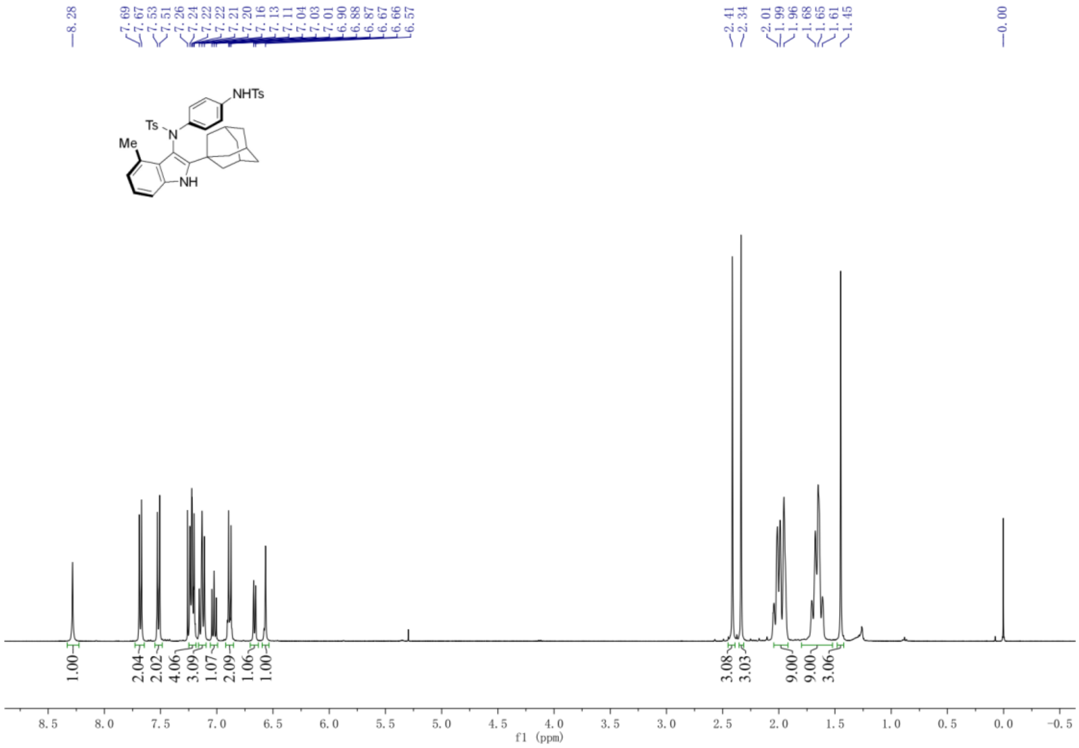

^{1}H NMR of **12-7** ($CDCl_3$)

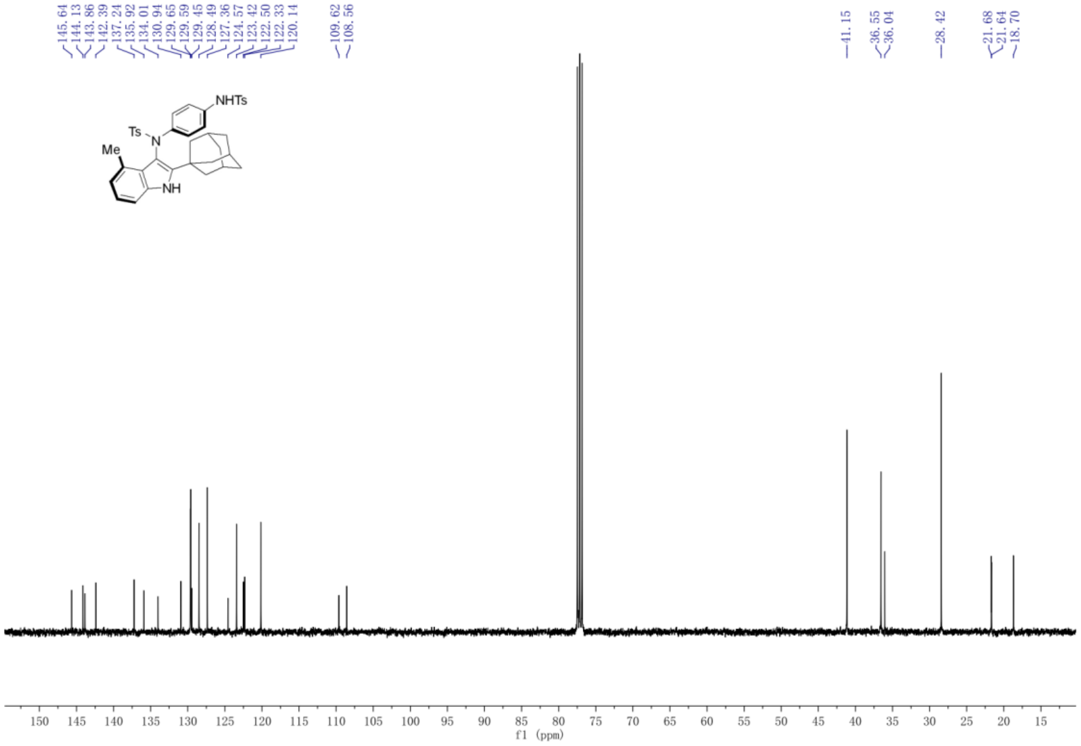

^{13}C NMR of **12-7** ($CDCl_3$)

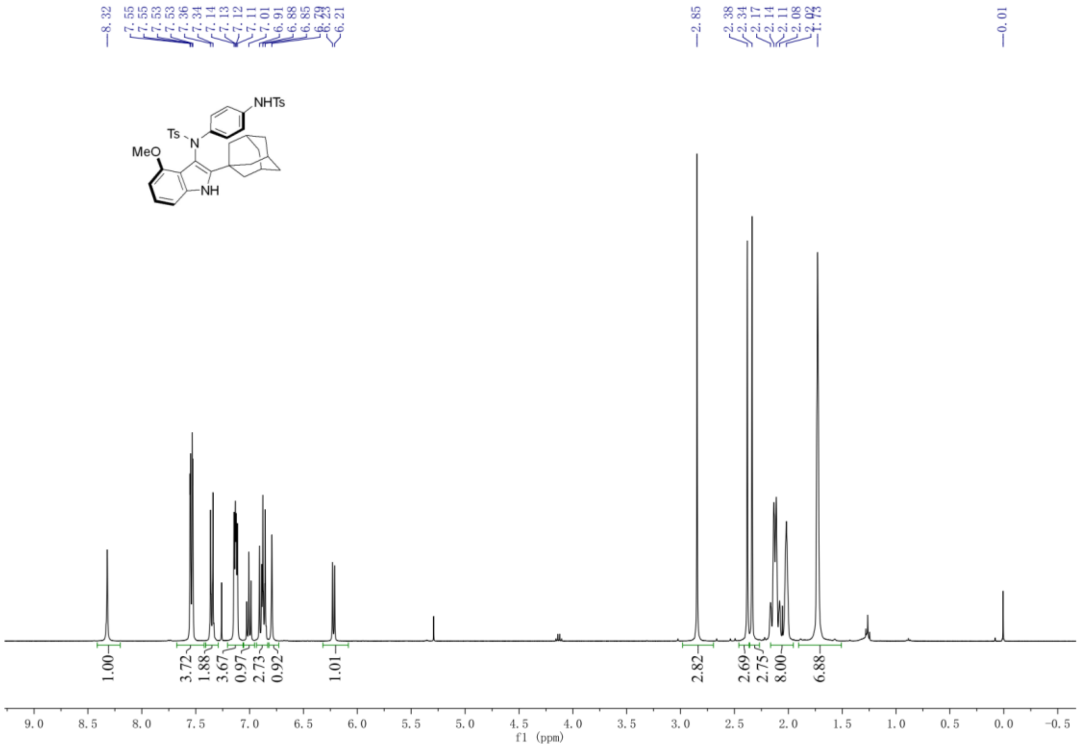

^{1}H NMR of **12-8** ($CDCl_3$)

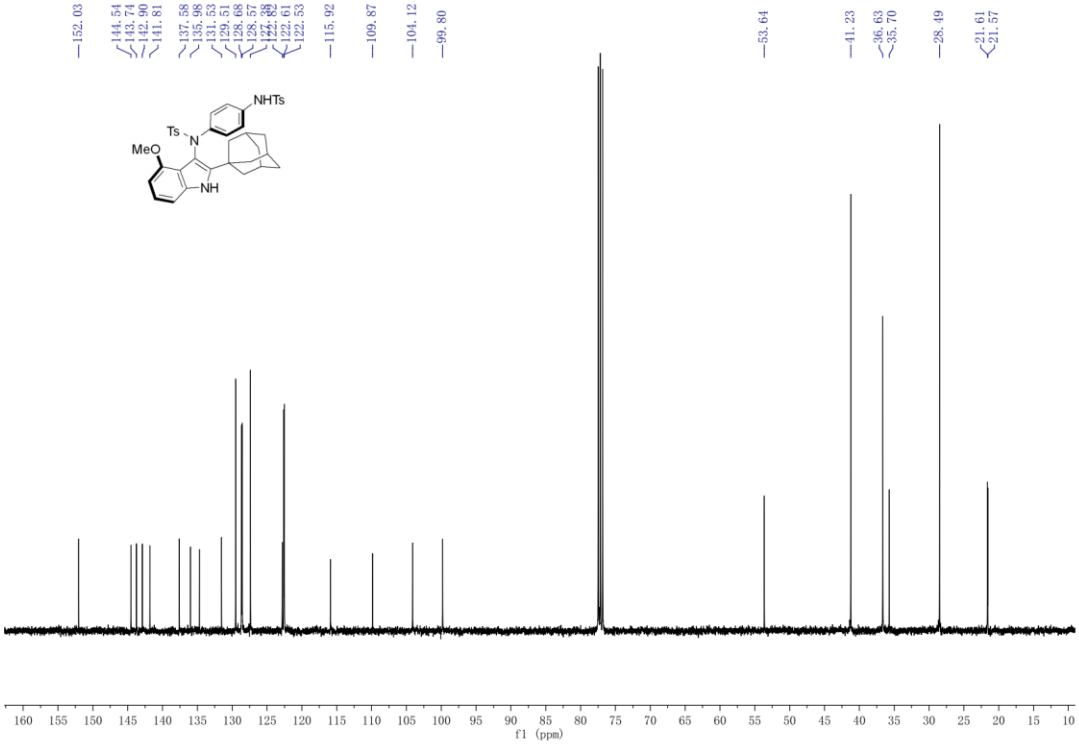

^{13}C NMR of **12-8** ($CDCl_3$)

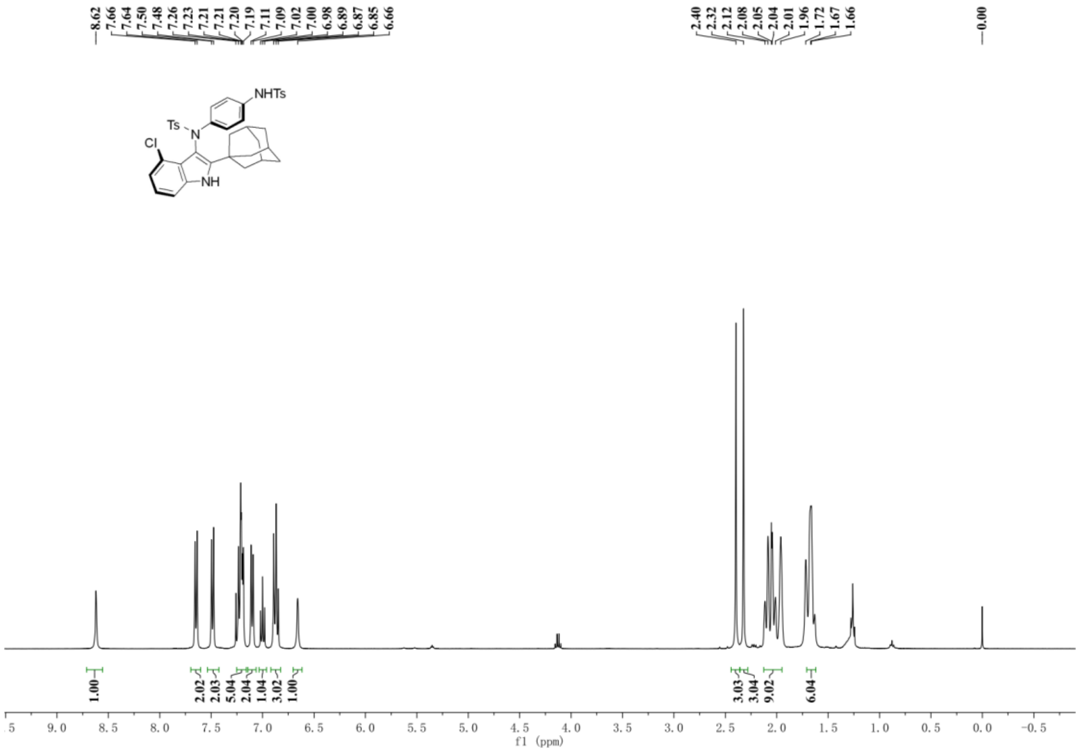

^{1}H NMR of **12-9** ($CDCl_3$)

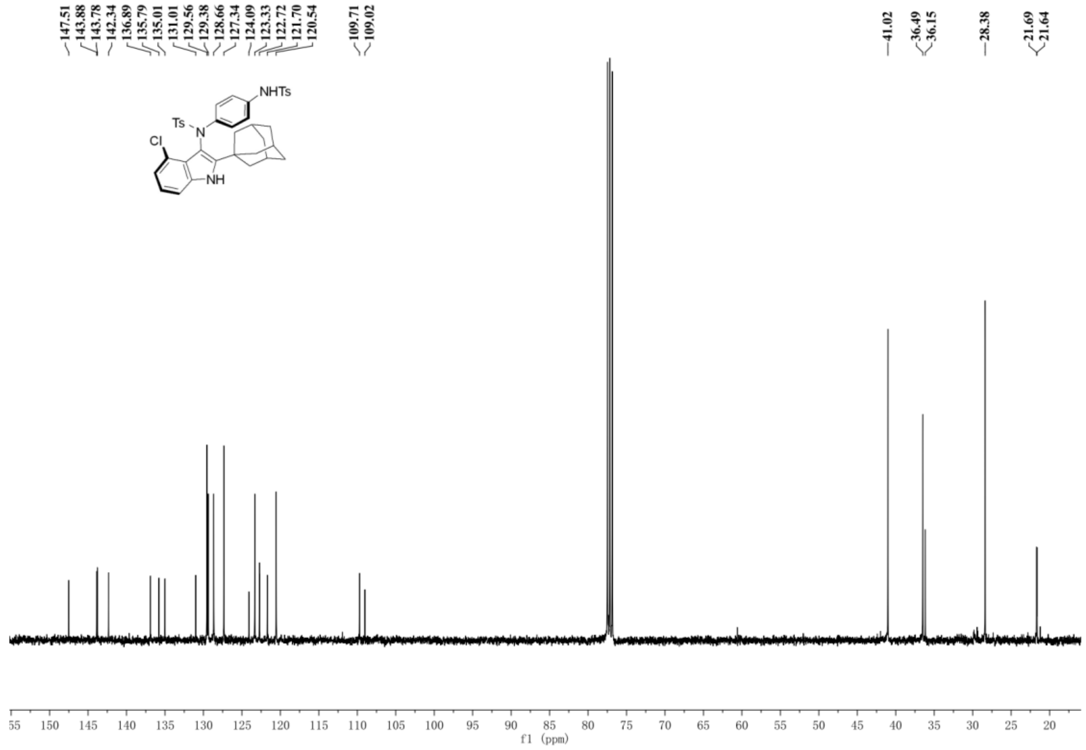

^{13}C NMR of **12-9** ($CDCl_3$)

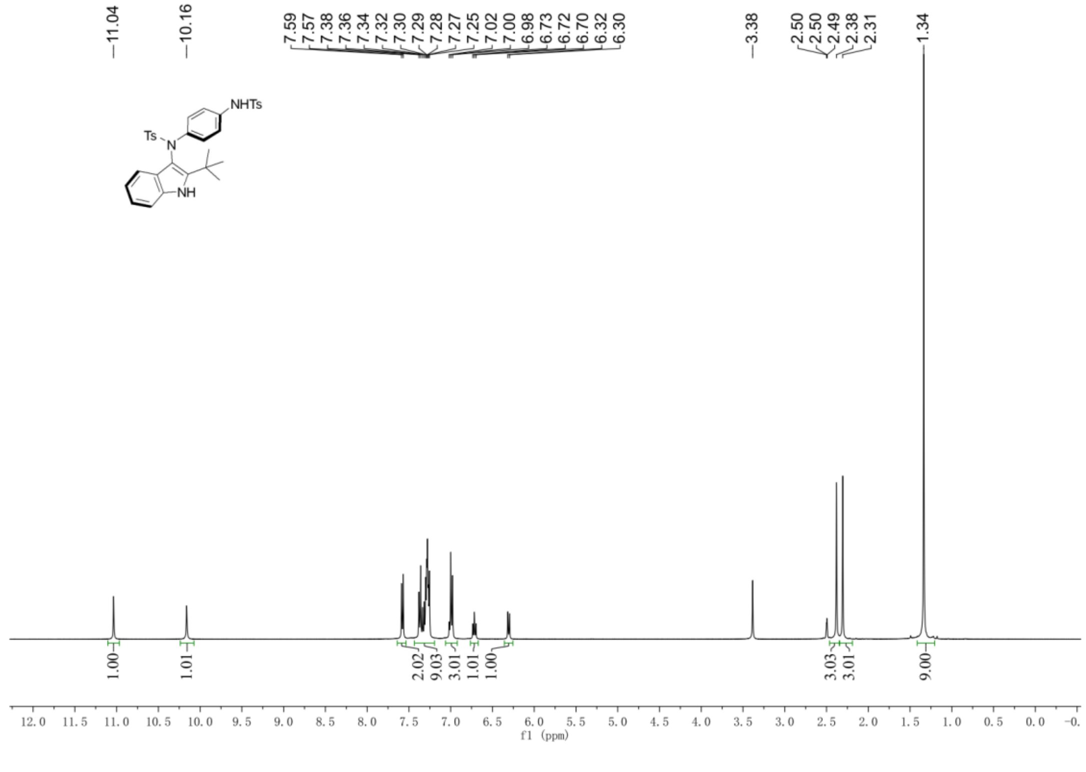

^{1}H NMR of **12-10** (DMSO-d_6)

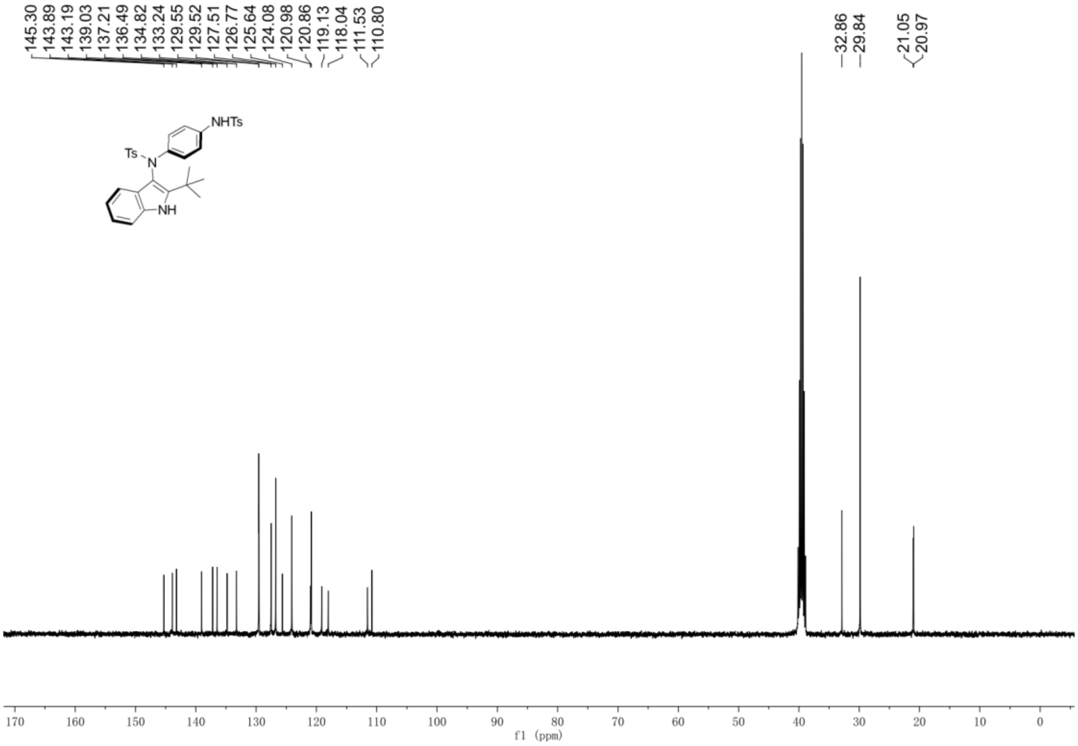

^{13}C NMR of **12-10** (DMSO-d_6)

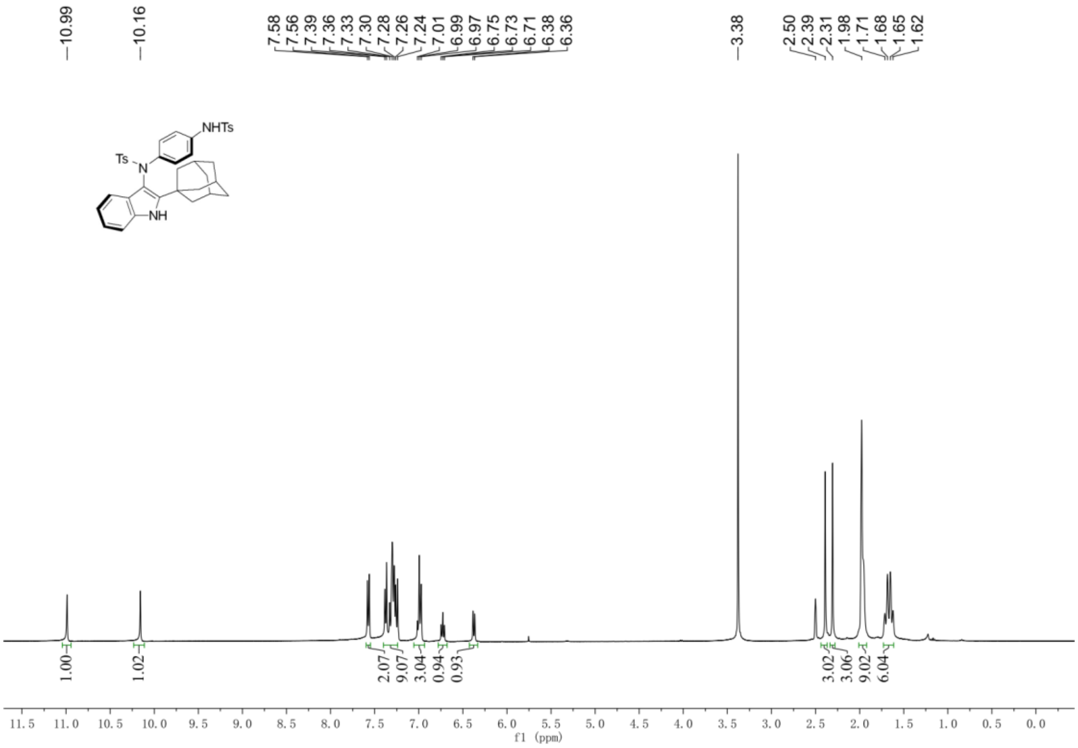

^{1}H NMR of **12-11** (DMSO-d_6)

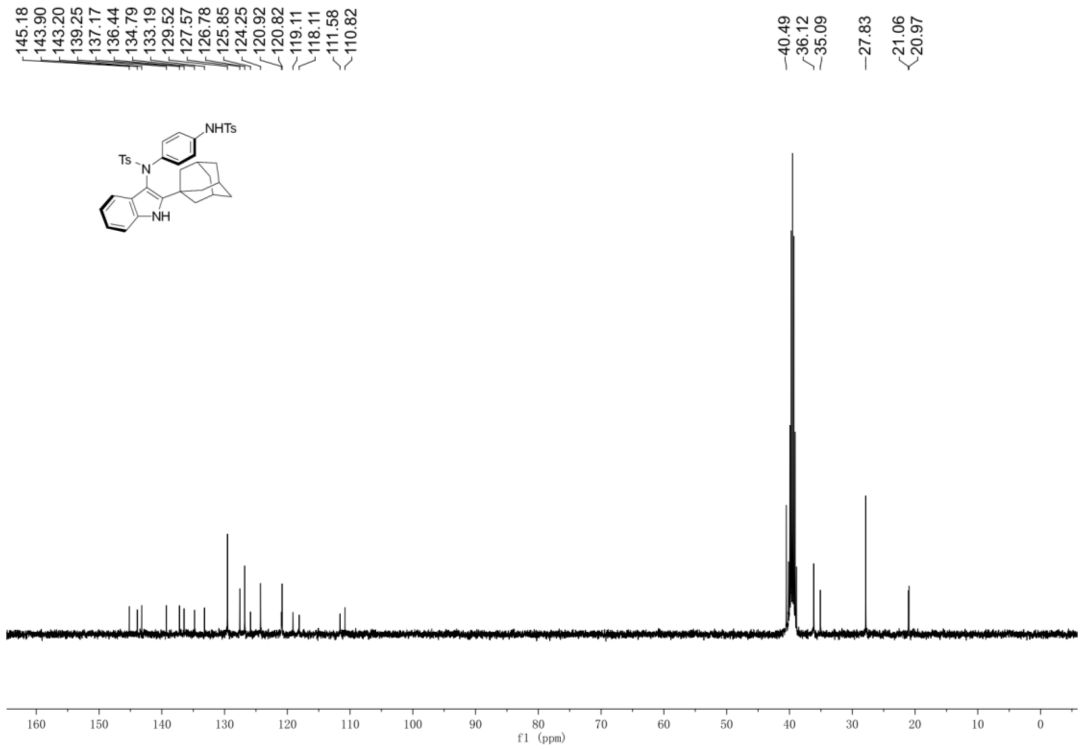

^{13}C NMR of **12-11** (DMSO-d_6)
